# Prespecified dental mesenchymal cells for the making of a tooth

**DOI:** 10.1038/s41368-025-00391-7

**Published:** 2025-10-09

**Authors:** Eun-Jung Kim, Hyun-Yi Kim, Suyeon Lee, Junsu Kim, Shujin Li, Anish Ashok Adpaikar, Thantrira Porntaveetus, Senthil Kumar Baskaran, Jong-Min Lee, Han-Sung Jung

**Affiliations:** 1 Division in Anatomy and Developmental Biology, Department of Oral Biology, Taste Research Center, Oral Science Research Center, BK21 FOUR Project, Yonsei University College of Dentistry, Seoul, Korea; 2 NGeneS Inc., Ansan-si, Korea; 3 Center of Excellence in Precision Medicine and Digital Health, Geriatric Dentistry and Special Patients Care, Department of Physiology, Faculty of Dentistry, Chulalongkorn University, Bangkok, Thailand

**Keywords:** Developmental biology, Anatomy

## Abstract

Positional information plays a crucial role in embryonic pattern formation, yet its role in tooth development remains unexplored. In this study, we investigated the regional specification of lingual and buccal dental mesenchyme during tooth development. Tooth germs at the cap stage were dissected from mouse mandibles, and their lingual and buccal mesenchymal regions were separated for bulk RNA sequencing. Gene ontology analysis revealed that odontogenesis, pattern specification, and proliferation-related genes were enriched in the lingual mesenchyme, whereas stem cell development, mesenchymal differentiation, neural crest differentiation, and regeneration-related genes were predominant in the buccal mesenchyme. Reaggregation experiments using Wnt1^creERT/+^; R26R^tdT/+^ and WT mouse models demonstrated that lingual mesenchyme contributes to tooth formation, while buccal mesenchyme primarily supports surrounding tissues. Furthermore, only the lingual part of tooth germs exhibited odontogenic potential when cultured in vitro and transplanted under the kidney capsule. Bulk RNA transcriptomic analysis further validated the regional specification of the lingual and buccal mesenchyme. These findings provide novel insights into the molecular basis of positional information in tooth development and pattern formation.

## Introduction

Tooth development is a dynamic process encompassing the stages of the bud, the cap, and the bell, followed by root development, and subsequent tooth eruption.^1^ Bud-to-cap transition, mediated by epithelial-mesenchymal interactions, determines the fate of the cell based on positional information via cytokines/growth factors.^2,3^ With the onset of tooth development, the odontogenic potential shifts to the dental mesenchyme prior to epithelial morphogenesis at the bud stage.^4,5^ Since dental mesenchyme is primarily important in the early stage of tooth development, its odontogenic potential after the cap stage must be explored and in-depth characterization should be carried out to gain more insight into this specific subject.

Tooth has been extensively studied for patterning and associated factors, such as molar vs. incisor, tooth number, tooth size, cuspal size, and shape, etc.^6–8^ Additionally, differences in critically conserved signaling molecules have been reported to determine the shape of the incisors, canines, premolars, and molars along the anterior–posterior jaw axis.^9–11^ Heterospecific recombination and reaggregation techniques involve studying the macro patterning and micropatterning of the tooth including tooth size and tooth number, and the cusp size and cusp number, respectively, in an individual tooth.^7^ Furthermore, positional information refers to the cells acquiring positional identities and interpreting their unique positions to give rise to differential spatial patterns based on the gradients of morphogens, which are diffusible signaling molecules produced by the cells.^12–14^ This positional information is related to pattern formation, which refers to the process by which cells create spatial patterns based on their position.^12^ The evidence of positional information has been reported in the antennae and legs of a Drosophila.^15^ However, positional information in the dental mesenchyme is yet to be studied during tooth development.

Teeth grow typically in a buccal, lingual, anterior, and posterior directions. During tooth development, tooth germs develop differentially depending on the genes expressed on the lingual-buccal side. It is known that cusps are patterned from buccal to lingual side, which is driven by antagonistic Bmp4-Osr2 expression pathways.^16–18^ Tooth replacement in vertebrates is initiated from the end of the dental lamina, known as the successional dental lamina.^19^ Sox2 is a transcription factor expressed in the lingual part of successional dental lamina, marking epithelial competence for tooth development in mammals and reptiles.^19,20^ Studies indicate that Npnt plays a localized role in the basement membrane on the buccal side of the tooth germ and regulates the expression pattern of Sox2.^21^ The dental lamina splits off from the lingual side of the replaced tooth for subsequent tooth renewal.^22^ Although some gene expression patterns in the lingual-buccal side of tooth germs during tooth development are well-known during tooth development, its positional characteristics in the dental mesenchyme have not been studied adequately. Therefore, an understanding of lingual-buccal differences is vital.

In this study, we aimed to identify the characteristics and differences between the lingual and buccal sides of tooth germs, a research that has the potential to significantly impact our understanding of tooth development. To achieve this objective, tooth germs were cut in the mesio-distal direction to compare gene expression between the lingual and buccal sides of the dental mesenchyme at the cap stage. We discovered the role of the unraveled gene, R-spondin1, in odontogenesis. Furthermore, we identified the cellular self-organization, where cells can remember their original position by reaggregating the dental mesenchymal cells from the lingual or buccal parts respectively. These findings provide new insights into the molecular mechanisms underlying tooth development and highlight the importance of positional information in the dental mesenchyme, paving the way for future studies on tooth patterning and regeneration.

## Results

### Temporal transcriptomic information of dental mesenchyme at cap and bell stage

**Fig. 1:**
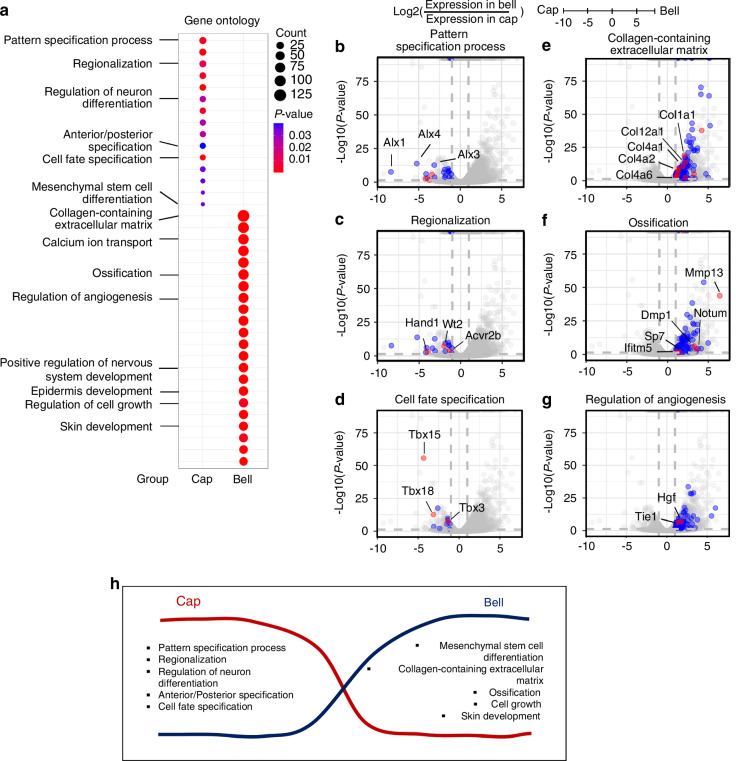
Transcriptomic analysis in dental mesenchyme between cap and bell stage. **a** Dot plot for GO analysis. In cap stage, GO terms for pattern specification, regionalization, regulation of neuron differentiation, anterior/posterior specification, cell fate specification, and mesenchymal stem cell differentiation are enriched at cap stage, while GO terms for collagen-containing extracellular matrix, calcium transport, ossification, regulation of angiogenesis, positive regulation of nervous system development, epidermis development, regulation of cell growth, and skin development were upregulated in bell stage. Fold changes are shown for genes related to specific ontology terms as red (interested genes in |fold change | >2 and *P* value < 0.001), blue ( | fold change | <2 or *P* value > 0.001), and gray (not related to the indicated GO term) dots. **b**–**d** The genes related to GO terms are upregulated in the dental mesenchyme at cap stage. **e**–**g** The genes related to GO terms are enriched in the dental mesenchyme at bell stage. **h** Illustration of upregulated GO terms at cap and bell stage

To identify the temporal transcriptomic information of the dental mesenchyme, it was isolated from the cap and bell stages and RNAs were collected for bulk RNA sequencing. Gene ontology (GO) analysis based on differentially expressed genes (DEG) (cap vs. bell stage, *P* < 0.05) identified 2 174 and 152 terms for the upregulated and downregulated gene groups, respectively (Supplementary Tables 1, 2). Dot plots of GO analysis overrepresented the results of upregulated DEGs in the dental mesenchyme at the cap stage compared to the bell stage (Supplementary Fig. 1a). The major terms of GO analysis of dental mesenchyme at the cap stage are pattern specification, regionalization, regulation of neuron differentiation, anterior/posterior specification, cell fate specification, and mesenchymal differentiation (Fig. 1a, h). Genes related to pattern specification (*Alx1/3/4*) (Fig. 1b), regionalization (*Hand1*, *Wt2*, *Acvr2b*) (Fig. 1c), and cell fate specification (*Tbx3/15/18*) (Fig. 1d), were upregulated. At the Bell stage, collagen-containing extracellular matrix, calcium ion transport, ossification, regulation of angiogenesis, positive regulation of nervous system development, epidermis development, regulation of cell growth, and skin development were annotated (Fig. 1a, h). The upregulated genes in the dental mesenchyme at the bell stage were associated with collagen-containing ECM (*Col1/2/4*) (Fig. 1e), ossification (*Mmp13, Notum, Dmp1, Ifitm5, Sp7*) (Fig. 1f), and regulation of angiogenesis (*Hgf, Tie1*) (Fig. 1g). The GO terms which were upregulated at cap, or bell stage were illustrated in Fig. 1h. Genes upregulated at cap stage (Supplementary Fig. 1b) and at bell stage (Supplementary Fig. 1c) were validated with RT-qPCR.

### The asymmetrical expression pattern of tooth germs at cap stage

**Fig. 2:**
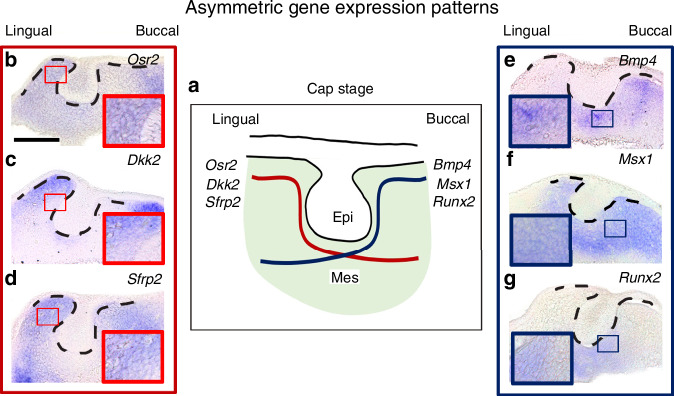
Differences of gene expression in lingual-buccal asymmetry during tooth development at cap stage. **a** Diagram of differential gene expression in the developing tooth germ at cap stage. **b**, **c** *Osr2* and *Dkk2* are expressed in lingual dental mesenchyme at the cap stage. **d** *Sfrp2* expression is more pronounced in lingual dental mesenchyme than in the buccal dental mesenchyme. **e** *Bmp4* expression in the buccal dental mesenchyme. **f** *Msx1* expression is shown in buccal and lingual dental mesenchyme, but strong expression is detected in the lingual part of dental mesenchyme. **g** *Runx2* expression is more pronounced in lingual dental mesenchyme. (Black dotted lines; tooth germs; Scale bar = 100 µm)

Previous studies have suggested that several genes are asymmetrically expressed in the lingual or buccal parts of the dental mesenchyme at the cap stage (Fig. 2a). In situ hybridization experiments revealed that the genes *Osr2*, *Dkk2*, and *Sfrp2* were expressed in the lingual area of the dental mesenchyme (Fig. 2b–d).^23–25^ In contrast, *Bmp4*, *Msx1*, and *Runx2* were found to be expressed in the buccal region (Fig. 2e–g).^25,26^

It is known that most genes are expressed evenly in the dental epithelium. *Sox2* expressed in the lingual part, is associated with the development of successional teeth in this region.^20^ However, it is controversial whether it is involved in epithelial cell proliferation or not.^19^ Thus, this study focused on the dental mesenchyme at the cap stage, marking the period when the odontogenic potential shifts from the bud stage epithelium to the mesenchyme.

### Bulk RNA sequencing analysis of dental mesenchyme at cap and bell stage

To further identify transcriptomic information of the dental mesenchyme, the lingual and buccal parts of the tooth germ were divided using a fine syringe at the cap and bell stages. Each part of the dental mesenchyme was isolated, and RNA was collected for bulk RNA sequencing analysis.

Dot plots of GO overrepresented the results of upregulated DEGs in the lingual part of the dental mesenchyme compared to the buccal part at the cap and bell stages (Supplementary Fig. 2a and Tables 3–6). The GO terms of the upregulated DEGs in the lingual part of the dental mesenchyme at the cap stage were embryonic organ morphogenesis, extracellular matrix organization, ossification, and skeletal system morphogenesis, whereas GO results for the upregulated DEGs in the buccal part of the dental mesenchyme show that they were enriched in mesenchyme development, muscle tissue development, neural crest differentiation and organ growth, regeneration, and stem cell development.

**Fig. 3:**
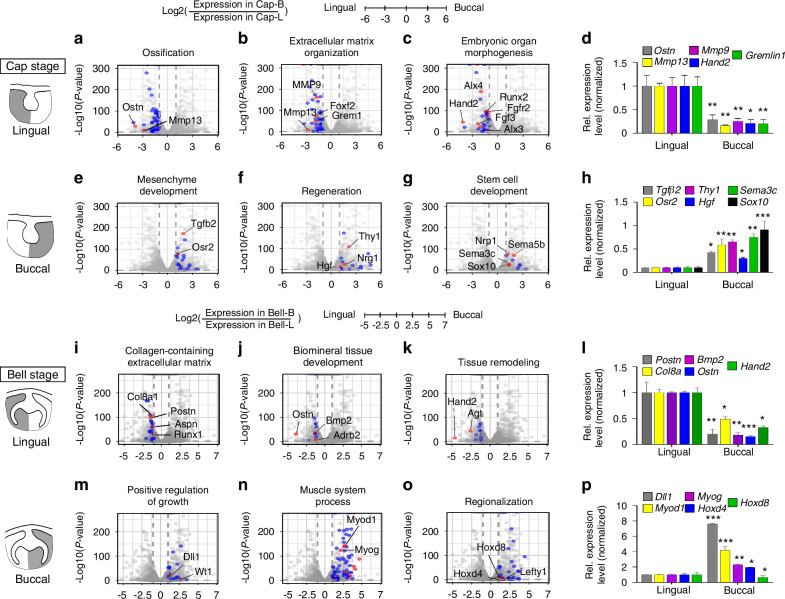
Transcriptomic change in lingual and buccal part of tooth germs at cap and bell stage. Volcano plots of DEGs of lingual or buccal part of the dental mesenchymal cells between cap and bell stage. Indicated GO term-related genes are shown as red, blue, and gray (not related to the indicated GO term) dots. The related genes are enriched (**a**–**c**) in lingual part, (**e**–**g**) buccal part of dental mesenchymal cells at cap stage. GO term-related genes are upregulated (**i**–**k**) in lingual part, and (**m**–**o**) in buccal part of the dental mesenchymal cells at bell stage. **d**, **h**, **l**, **p** Validation of the related genes in each group with RT-qPCR. (*P < 0.05, **P < 0.01; ***P < 0.001). Data are presented as means ± standard deviations (SD)

Genes related to osteogenesis (*Ostn* and *MMP13*) (Fig. 3a), extracellular matrix organization (*Mmp9/13*, *Foxf2*, *Grem1*) (Fig. 3b), and embryonic organ morphogenesis (*Alx3/4*, *Hand2*, *Runx2*, *Fgf3*, *Fgfr2*) (Fig. 3c) were upregulated in the lingual part at the cap stage (Fig. 3d). In the corresponding buccal part, the upregulated genes were enriched in mesenchyme development (*Tgfb2*, *Osr2*) (Fig. 3e), regeneration (*Thy1*, *Nrg1*, *Hgf*) (Fig. 3f), and stem cell development (*Nrp1*, *Sema3c/5b*, *Sox10*) (Fig. 3g). Some of upregulated genes were validated (Fig. 3h).

**Fig. 4:**
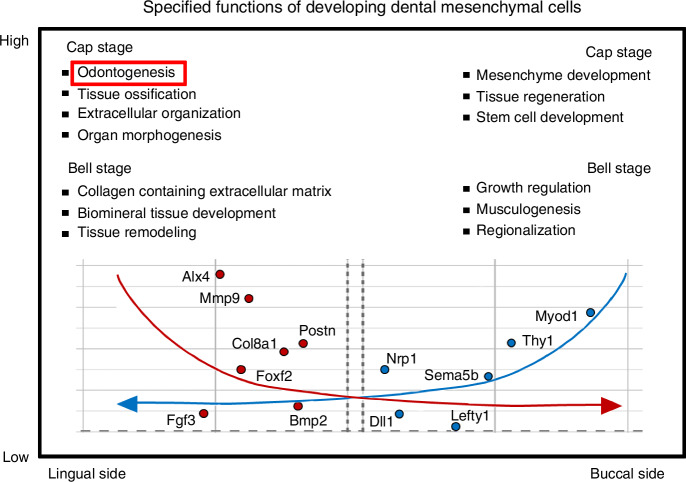
Diagram for specified functions of developing dental mesenchymal cells along the lingual-buccal axis. It depicts gene expression patterns and GO terms related to biological processes in the dental mesenchyme during the cap and bell stages of tooth development. The upper panel presents GO terms that are enriched in the lingual (left) and buccal (right) sides during the cap and bell stages. The lower panel features volcano plots that compare gene expression between the lingual (left) and buccal (right) sides. The left plot highlights genes that are upregulated in the lingual mesenchyme at both the cap and bell stages, while the right plot illustrates genes that are upregulated in the buccal mesenchyme during these same stages. The annotated genes are those that demonstrate significant differential expression

The GO terms of the upregulated DEGs in the lingual part of the dental mesenchyme at the bell stage were biomineral tissue development, bone development, and collagen-containing extracellular matrix. In contrast, the GO terms of the upregulated DEGs in the buccal part were calcium ion transport, positive regulation of growth (Supplementary Fig. 2a). Genes related to collagen-containing extracellular matrix (*Col8a1, Postn, Aspn, Runx1*) (Fig. 3i), biomineral tissue development (*Ostn, Bmp2, Adrb2*) (Fig. 3j), and tissue remodeling (*Agt, Hand2*) (Fig. 3k) were upregulated (Fig. 3l). In the buccal part, at the bell stage, the upregulated genes were involved in different GO terms including positive regulation of growth (*Dll1*, *Wt1*) (Fig. 3m), muscle system process (*Myod1*, *Myog*) (Fig. 3n), and regionalization (*Hoxd4/8*, *Lefty1*) (Fig. 3o, p). Consequently, on the lingual side of the dental mesenchyme, during tooth development, the characteristics of tooth morphogenesis, complexed hard tissue formation, and differentiation were exhibited, whereas on the buccal side, the information including stem cell regeneration and growth were more pronounced. Therefore, distinct molecular signatures in the lingual and buccal dental mesenchyme suggest their specialized roles in different stages of tooth morphogenesis (Fig. 4).

### The odontogenic capacity of the lingual and buccal parts of dental mesenchyme

**Fig. 5:**
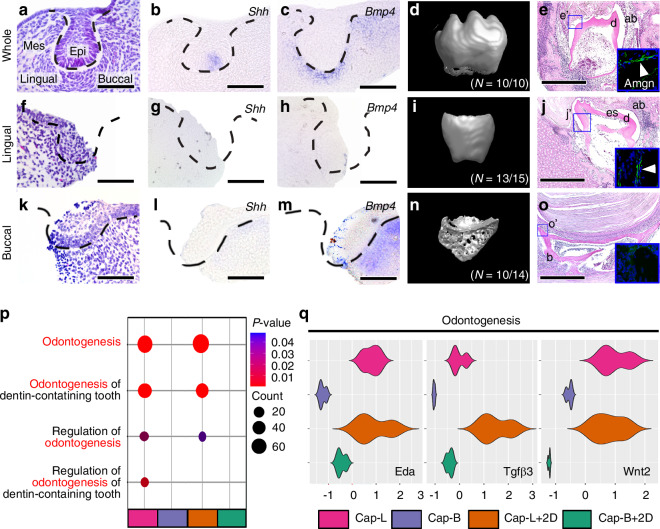
Comparison of the capacity for tooth formation between the lingual and buccal part of tooth germs at cap stage (**a**, **f**, **k**) H&E staining shows **a** whole tooth germs, **f** lingual, and **k** buccal part of tooth germs, after dissection with lingual buccal direction. Enamel knot markers, such as (**b**, **g**, **l**) *Shh* and (**c**, **h**, **m**) *Bmp4*, were validated in **b**, **c** whole, **g**, **h** lingual, and **l**, **m** buccal part of the tooth germs at cap stage. **d** Whole tooth germs, **i** lingual, and **n** buccal part of the tooth germs at cap stage were cultured in vitro for 2 days, and transplanted into kidney capsule for 4 weeks. **e**, **j**, **o** H&E staining of the sectioned transplants showed a calcified tooth and bone from **e** whole tooth germ and **j** lingual part of tooth germ transplants, however, **o** only bone was observed from buccal part of the tooth germ transplants. **e**’, **j**’, **o**’ The blue box highlights the high-magnification view of Amgn expression in **e**, **j**, and **o**. **p** A dot plot of GO terms including “odontogenesis” results for upregulated DEGs in lingual part of cap stage (Cap-L) than in buccal part of cap stage (Cap-B), and in separated lingual part of dental mesenchyme, and cultured in vitro for 2 days without buccal part (Cap-L + 2D) than in separated buccal part of dental mesenchyme, and cultured in vitro for 2 days without lingual part (Cap-B + 2D). **q** The violin plot for GSEA. In odontogenesis, *Eda*, *Tgfβ3*, *Wnt2* are enriched in lingual part of dental mesenchyme in cap and bell stage, and the lingual dental mesenchyme which was cultured in vitro for 2 days. (Black dotted line; tooth germs; Epi Epithelium, Mes Mesenchyme, d dentin, es enamel space, ab ameloblasts, b bone, Scale bar: **a**–**c**, **e**–**h**, **k**–**m** = 100 µm, **e**, **j**, **o** = 1 mm)

To identify the odontogenic capacity, the lingual or buccal parts of the tooth germs at the cap stage were isolated and analyzed. The whole tooth germ at the cap stage (Fig. 5a) expressed *Shh* in the presumptive enamel knot (Fig. 5b) and *Bmp4* in the dental mesenchyme (Fig. 5c). When the tooth germ was transplanted into the kidney capsule for calcification, mineralized teeth were found with six to seven cusps were observed (Fig. 5d), and Amelogenin (Amgn) was expressed in ameloblasts in a mineralized tooth (Fig. 5e). In case of the lingual part of the tooth germ (Fig. 5f), *Shh* and *Bmp4* expressions were not found (Fig. 5g, h). Additionally, after kidney transplantation, a calcified tooth with a flattened cusp was observed (Fig. 5i), and Amgn expression was shown in ameloblasts (Fig. 5j). However, the buccal part of the tooth germ (Fig. 5k) did not express *Shh* (Fig. 5l), but *Bmp4* expression was indicated in buccal dental mesenchyme (Fig. 5m). Furthermore, only surrounding bone without calcified tooth was evident in the kidney transplants (Fig. 5n). Therefore, Amgn expression not found in a mineralized tissue (Fig. 5o).

The GO terms analysis revealed that odontogenesis, odontogenesis of dentin-containing tooth, regulation of odontogenesis, and regulation of odontogenesis of the dentin-containing tooth were upregulated only in the lingual part of the dental mesenchyme (Cap-L), not in the buccal part at cap stage (Cap-B) (Fig. 5p). The related genes in odontogenesis (*Eda*, *Tgfβ3*, *Wnt2*) were upregulated in the lingual part of the dental mesenchyme at cap stage (Cap-L) and in the lingual part of dental mesenchyme, which were cultured in vitro for 2 days (Cap-L + 2D), compared to the buccal part of dental mesenchyme (Cap-B), and to the buccal part of dental mesenchyme, which were cultured in vitro for 2 days (Cap-B + 2D) (Fig. 5q).

**Fig. 6:**
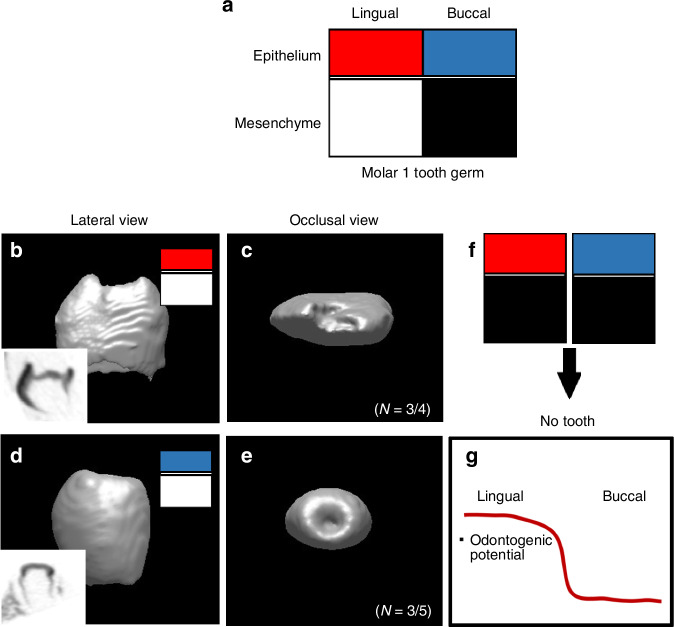
Assessment of odontogenic potential through recombination experiments of lingual and buccal dental mesenchyme at the cap stage. **a** Schematic representation of the experimental design. Tooth germs were isolated at the cap stage, and their lingual and buccal parts were separated. The dental epithelium and mesenchyme were individually dissociated and recombined in various combinations. **b**, **c** microCT view shows that calcified teeth were observed in recombinants composed of lingual epithelium with lingual mesenchyme. **d**, **e** Similarly, calcified teeth formed in recombinants of buccal epithelium with lingual mesenchyme. (the box in **b**, **d**) Sagittal view showed the mineralization in tooth. **f** Recombinants consisting of lingual epithelium with buccal mesenchyme or buccal epithelium with buccal mesenchyme did not form calcified teeth. **g** Diagram shows odontogenic potential in lingual part of dental mesenchyme

To assess the odontogenic potential of Cap-L dental mesenchyme, tooth germs were isolated at the cap stage and separated into their lingual and buccal parts. The dental epithelium and mesenchyme were then individually seperated and recombined in a cross-experimental manner (Fig. 6a). Calcified teeth were observed in recombinants composed of lingual epithelium with lingual mesenchyme (Fig. 6b, c), as well as in those recombining buccal epithelium with lingual mesenchyme (Fig. 6d, e). To validate that tooth formation occurred as a result of this recombination, we included sectional micro-computed tomography (micro-CT) images.^8^ In contrast, recombinants consisting of lingual epithelium with buccal mesenchyme or buccal epithelium with buccal mesenchyme did not form calcified teeth (Fig. 6f). Therefore, at the cap stage, the lingual mesenchyme exhibits greater odontogenic potential than the buccal mesenchyme (Fig. 6g).

### The cellular self-organization

**Fig. 7:**
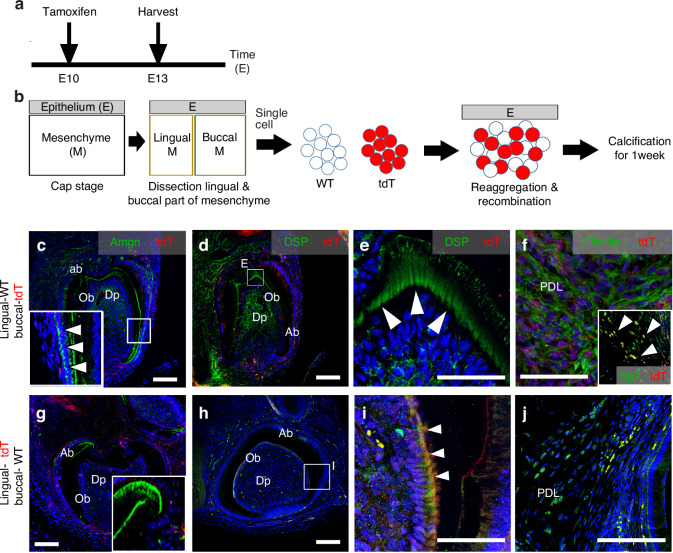
Positional value of the randomized dental mesenchymal cells. **a** Schematic of the experimental design for Wnt1^CreERT2/+^;R26R^tdT/+^ mouse. Tamoxifen-induced Cre activates tdTomato fluorescent protein (red), which labels Wnt1 cells, meaning dental mesenchymal cells. **b** The diagram shows the reaggregated system. Dissociated single cell from lingual and buccal dental mesenchyme were collected. Lingual dental mesenchymal cells from WT and buccal dental mesenchymal cells from Wnt1^CreERT2/+^;R26R^tdT/+^ tooth germs or lingual dental mesenchymal cells from Wnt1^CreERT2/+^;R26R^tdT/+^ tooth germs and buccal dental mesenchymal cells from WT were reaggregated. These mixed cells were reaggregated and recombined with dental epithelium from WT at cap stage. For the calcification, the recombinants were transplanted into kidney capsule for 1 week. After 1 week, in calcified teeth, in recombinants of lingual dental mesenchymal cells from WT and buccal from Wnt1^CreERT2/+^;R26R^tdT/+^ tooth germs, **c** Amgn (ameloblast), **d**, **e** DSP (odontoblasts) are not co-localized with tdTomato. However, **f** Fibillin (PDL cells) and Sp7 (osteoblasts, in box) are co-localized with tdTomato. In recombinants of lingual dental mesenchymal cells from Wnt1^CreERT2/+^;R26R^tdT/+^ and buccal from WT tooth germs, **g** Amgn and **j** Fibrillin are not co-localized with tdTomato, however, **h**, **i** DSP is co-localized with tdTomato. (E Epithelium, M Mesenchyme, ab ameloblast, Ob odontoblast, Dp dental pulp, PDL periodontal ligament, white arrow head=expressed cells, Scale bar: **c**, **d**, **g**, **h** = 100 µm, **e**, **f**, **i**, **j** = 50 µm)

To investigate the regional characteristics of dental mesenchymal cells, we used Wnt1cre^ERT/+^;R26R^tdT/+^ mice. In these mice, cells derived from Wnt1 are specifically marked by the tdTomato label, allowing us to identify the dental mesenchymal cell population. The mice were treated with tamoxifen for one day at embryonic day 10 (E10), and the dental mesenchyme was harvested at the cap stage (Fig. 7a). For the reaggregation system, we harvested the lingual or buccal sides of the dental mesenchyme, dissociated them into single cells, reaggregated these cells separately, and then recombined them with the epithelium (Fig. 7b). First, we reaggregated lingual mesenchymal cells from wild-type (WT) mice with buccal mesenchymal cells from Wnt1cre^ERT/+^;R26R^tdT/+^ mice, and these were recombined with dental epithelium from WT mice (labeled as Lingual-WT/Buccal-tdT, Fig. 7c–f). Similarly, we reaggregated lingual mesenchymal cells from Wnt1cre^ERT/+^;R26R^tdT/+^ mice with buccal mesenchymal cells from WT mice, and recombined these with dental epithelium from WT mice (labeled as Lingual-tdT/Buccal-WT, Fig. 7g–j). After one week, calcified teeth and bone were observed in the kidney capsule. The surrounding Fibrillin-positive periodontal ligament cells and Sp7-positive osteoblasts were composed of buccal dental mesenchymal cells derived from Wnt1-derived tdTomato (Fig. 7f). Amelogenin (Amgn), a marker for ameloblasts, was expressed in both ameloblasts and odontoblasts, as well as in the surrounding alveolar bone (Fig. 7c, g).^27^ On the other hand, Dentin sialoprotein (Dsp), a marker for odontoblasts, was found in odontoblasts, dental pulp, and the surrounding periodontal ligament (PDL) and bone (Fig. 7d, e).^28^ Notably, neither Amgn nor Dsp co-localized with tdTomato. In contrast, within the dental pulp cells and odontoblasts that express tdTomato and were derived from lingual mesenchyme, Dsp was co-localized with tdTomato (Fig. 7h, i). However, the surrounding PDL, which tested positive for Fibrillin, did not co-express tdTomato (Fig. 7j). These findings indicate that lingual dental mesenchymal cells primarily contribute to tooth formation, whereas buccal dental mesenchymal cells play a role in forming the surrounding tissues.

### Rspondin1 as a novel regulator of the odontogenic potential at cap stage

To detect the novel regulatory genes involved in tooth formation, we analyzed the commonly upregulated signaling pathways related to the “signaling pathway” GO term in the following three groups: 1) dental mesenchyme of the lingual (Cap-L) or buccal (Cap-B) part at the cap stage, 2) dental mesenchyme of the lingual (Bell-L) or buccal (Bell-B) part at the bell stage, 3) dental mesenchyme of the lingual (Cap-L + 2D) or buccal (Cap-B + 2D) part after cultured in vitro for two days at the cap stage (Supplementary Tables 7, 8). It is known that there is approximately a two-day difference between the cap stage and the bell stage in mouse tooth development. Therefore, we attempted to analyze the lingual and buccal sides after culturing them in vitro for two days.

**Fig. 8:**
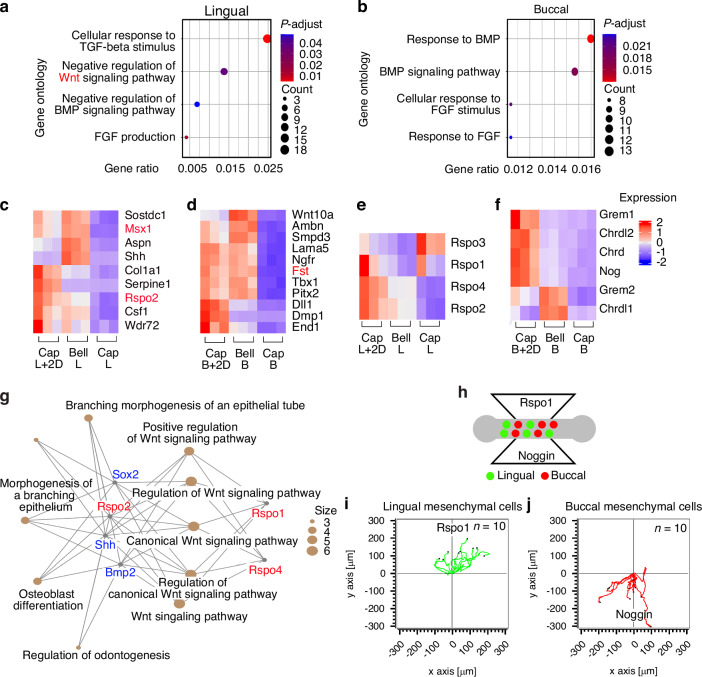
Regulation of odontogenic/osteogenic capacity at cap stage. **a** A dot plot visualizing GO overrepresentation results for upregulated genes. GO terms related with signaling pathway are identified in commonly upregulated signaling pathway in dental mesenchyme of the lingual part at cap stage, in dental mesenchyme of the lingual at bell stage; color and size of the dots indicate the *p* value and gene ratio of each representative GO term, respectively. **b** A dot plot shows GO terms related with signaling pathway in commonly upregulated signaling pathway in dental mesenchyme of the buccal part at cap and bell stage, and dental mesenchyme of the buccal part cultured in vitro for 2 days from cap stage. Heat map visualizes the upregulated genes related to the “odontogenesis” GO terms among Cap-L + 2D, Bell-L, and Cap-L groups (**c**), and Cap-B + 2D, Bell-B, and Cap-B groups (**d**). **e** Among the R-spondin family members, *Rspo1*, *2*, and *4* were upregulated in Cap-L + 2D group. **f** Among Bmp inhibitors, *Grem1*, *Chrdl2* (chordin-like 2), *Chrd* (chordin), and *Noggin* were upregulated in Cap-B + 2D. **g** cnet plot analysis for Rspo1, Rspo2, and Rspo4 are associated with key dental epithelial cell factors through various GO terms. All GO terms are mediated via the Wnt signaling pathway. **h** Lingual dental mesenchymal cells labeled with green and buccal dental mesenchymal cells labeled with red were seeded a microslide chemotaxis chamber with Rspo1 CM and Noggin protein. **i**, **j** Trajectory plots depicted the movement path of individual lingual mesenchymal cells (*n* = 10) and buccal mesenchymal cells (*n* = 10). Through transformation, all trajectories started at the origin (x = 0, *y* = 0) in order to visualize the movements of all cells relative to each other

The upregulation of GO terms was related to cellular response to TGF-beta stimulus, negative regulation of Wnt and Bmp signaling pathway, negative regulation of BMP signaling pathway, and FGF production in Cap-L, Bell-L, and Cap-L + 2D groups (Fig. 8a). In addition, the response to bone morphogenetic protein, BMP signaling pathway, cellular response to FGF stimulus, and response to FGF were upregulated commonly in the buccal part of the dental mesenchyme Cap-B, Bell-B, and Cap-B + 2D groups (Fig. 8b). Furthermore, the upregulated genes related to the “odontogenesis” GO terms were analyzed in the three groups. Among these genes, *Rspo2* was upregulated in Cap-L + 2D, compared to Cap-L and Bell-L (Fig. 8c), while *Fst* was upregulated in Cap-B + 2D and Bell-B (Fig. 8d). R-spondin 2 (Rspo2) is a powerful stem cell growth factor and a key regulator in dental and craniofacial development. It significantly enhances Wnt/β-catenin signaling and plays a crucial role in cell differentiation and regeneration. Therefore, in this study, we focused on the roles of R-spondins.^29,30^ Among the R-spondin family members, *Rspo1*, *2*, and *4* were upregulated in Cap-L + 2D group (Fig. 8e). Furthermore, among Bmp inhibitors, *Grem1*, *Chrdl2* (chordin-like 2), *Chrd* (chordin), and *Noggin* were upregulated in Cap-B + 2D (Fig. 8f). Interestingly, the analysis of cnet plots indicated that Rspo1, Rspo2, and Rspo4 are linked to important factors in dental epithelial cells, such as *Shh*,^31^ *Bmp2*,^32^ and *Sox2*.^20^ These connections were associated with various GO terms, such as ‘branching morphogenesis of an epithelial tube’, ‘morphogenesis of a branching epithelium’, ‘osteoblast differentiation’, and ‘regulation of odontoblast differentiation’. All of these processes were mediated through the Wnt signaling pathway (Fig. 8g). These analyses indicated that Wnt inhibitory signaling is predominantly expressed in the lingual part, meaning that Wnt activity is naturally suppressed in this region in mouse tooth development, whereas BMP signaling is more highly expressed in the buccal part of the dental mesenchyme during the cap stage of tooth development.^33,34^ When only the lingual part was cultured (Cap-L+2D), Wnt signaling, particularly R-spondin (Wnt activator), was upregulated, enabling continued tooth germ development. In contrast, culturing only the buccal part (Cap-B+2D) led to increased BMP signaling along with upregulation of BMP inhibitors. This shift in signaling caused the buccal mesenchyme to favor bone development rather than tooth formation. These findings highlight the distinct regulatory roles of Wnt and BMP signaling in the regional specialization of dental mesenchyme.

To evaluate whether dental mesenchymal cells migrate via chemotaxis between the two regions of the dental mesenchyme (the lingual and buccal parts), and to determine if differences in growth factors can induce this migration during tooth formation, two populations of dental mesenchymal cells were stained with different colors, such as red, and green. Subsequently, Cell mobility toward the chemoattractant was monitored in real-time using a microslide-based chemotaxis assay (Fig. 8h). Equal numbers of green-stained lingual and red-stained buccal dental mesenchymal cells were seeded onto microslides. After treating the medium with R-spondin1 conditioned medium (CM) on one side and Noggin protein on the opposite side, the direction of cell migration was examined using trajectory plots (Fig. 8i, j). The individual cell migration was tracked within a 2D plane, as the trajectory plots. The lingual dental mesenchymal cells moved toward the Rspo1 CM (Fig. 8i), while buccal cells moved toward Noggin protein (Fig. 8j). These results suggested that the lingual-buccal axis in the dental mesenchyme defined the Wnt and Bmp signaling pathways.

### Differences in cell proliferation, apoptosis, and migration along the lingual-buccal axis of the dental mesenchyme

**Fig. 9:**
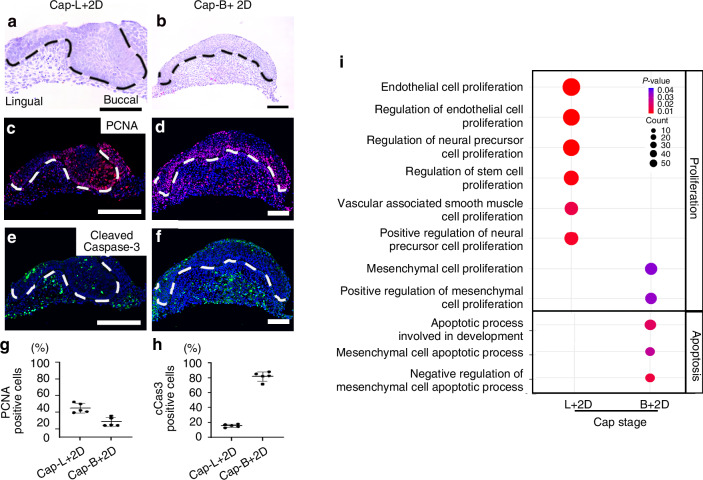
Changes of Cell proliferation and apoptosis in lingual and buccal part of dental mesenchyme. **a**, **b** H&E staining, **c**, **d** Cell proliferation (PCNA), and **e**, **f** cell death (cleaved Caspase-3) expression are compared between lingual part of dental mesenchyme, which were cultured in vitro for 2 days without buccal part (Cap-L+2D), and buccal part of dental mesenchyme, which were cultured in vitro for 2 days without lingual part (Cap-B + 2D), and (**g**) are quantified in graph. PCNA positive dental mesenchymal cells are more in Cap-L + 2D than in Cap-B + 2D, **h** while cleaved Caspase-3 positive cells are elevated in Cap-B + 2D compared to Cap-L + 2D. **i** GO terms related with cell proliferation more enriched in Cap-L + 2D, while GO terms related with apoptosis enriched more in Cap-B + 2D. (White and black dotted line; tooth germs, Scale bar = 100 µm)

To examine the changes in cell proliferation and apoptosis in the lingual (Cap-L + 2D) and buccal (Cap-B + 2D) dental mesenchyme after 2 days of in vitro culture we performed immunofluorescence with proliferating cell nuclear antigen (PCNA) and cleaved Caspase-3 expression (Fig. 9a–h). PCNA was expressed in the dental epithelium and mesenchyme of the lingual part of the tooth germ that was cultured in vitro for 2 days (Cap-L + 2D) (Fig. 9c). However, its expression decreased in the dental mesenchyme of the buccal part of the tooth germ cultured for 2 days (Cap-B + 2D) (Fig. 9d, g). Cleaved Caspase-3 expression was detected in the region of the dental epithelium that had been cut using a syringe, as well as in the dental mesenchyme in Cap-L + 2D (Fig. 9e). In contrast, an increase in cleaved Caspase-3 positive mesenchymal cells was observed in Cap-B + 2D compared to Cap-L + 2D (Fig. 9f, h). Furthermore, GO analysis showed that proliferation and apoptosis-related GO terms were upregulated in the Cap-L + 2D and in the Cap-B + 2D, respectively (Fig. 9i).

**Fig. 10:**
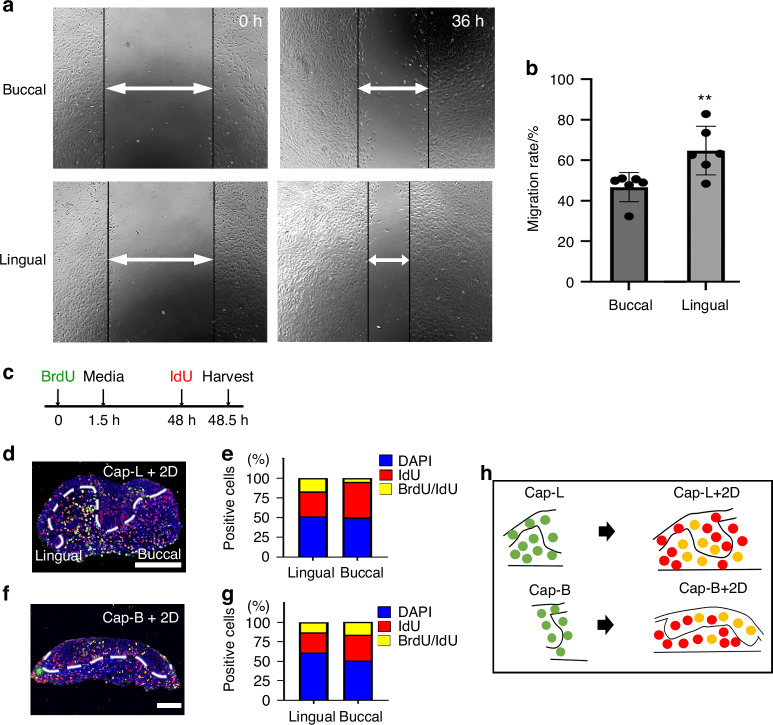
Migration rates and direction of lingual and buccal dental mesenchymal cells. **a** The migration of lingual and buccal dental mesenchymal cells was observed following a scratch wound after 36 h. **b** Quantitative analysis of cell migration rates 36 h post-scratch. **c** Media was treated with BrdU for 1.5 h, and media contained BrdU was changed into normal media. After in vitro culture for 48 h, IdU was treated for 30 min, and Cap-L + 2D and Cap-B + 2D were harvested to analyze for proliferative cells. **d** The BrdU/IdU co-localized cells are localized in lingual side, and newly proliferative red cells are localized on the other side (buccal side) of tooth germ in Cap-L + 2D. **f** While the BrdU/IdU co-localized cells were localized in buccal side and center, the red IdU cells proliferated toward both lingual and buccal side of tooth germ in Cap-B + 2D. **e**, **g** BrdU/IdU co-labeled cells, IdU-positive cells, and total nuclei (DAPI) were quantified using ImageJ, and the results were presented as graphs. **h** Diagram the directional movement of newly proliferating cells when the lingual or buccal part of the tooth germ is cultured in vitro. Data are presented as means ± standard deviations (SD). (White dotted line; tooth germ, Scale bar = 100 µm)

To observe the migration of lingual and buccal part of dental mesenchyme, wound healing assay was performed. Wound closure was determined 36 h after scratch. The results showed that lingual dental mesenchymal cells exhibited a higher migration rate compared to buccal dental mesenchymal cells (Fig. 10a, b). In addition, to determine the direction of growth of the tooth germs, BrdU was treated in the culture medium for 1.5 h, changed to normal medium, and cultured for 2 days. Subsequently, IdU was treated for 0.5 h just before the tooth germs were harvested (Fig. 10c). When the lingual part of the tooth was cultured in vitro for 2 days (Cap-L + 2D), BrdU-positive cells (green) proliferated and co-localized with IdU. The BrdU+IdU+ cells (yellow) proliferated from the lingual side, while IdU+ cells (red, newly proliferating cells) were observed on the buccal side (Fig. 10d, e). In contrast, when the buccal part was cultured in vitro for 2 days, BrdU+IdU+ cells (yellow) and IdU+ cells (red) were observed on both the lingual and buccal sides (Fig. 10f, g). Therefore, it was evident that the lingual dental mesenchymal cells proliferate toward the buccal side to form the tooth, whereas the buccal cells proliferate in both the directions to form the tissues surrounding the tooth (Fig. 10h).

## Discussion

Recent studies have investigated bulk and single-cell transcriptomics to provide an atlas of epithelial and mesenchymal compartments involved in tooth development in human and mouse teeth.^35,36^ Here, we comprehensively analyzed the transcriptomic differences between the lingual and buccal regions of the dental mesenchyme during tooth development.

In one of the previous studies, proximal-distal split tooth germs develop into multiple teeth via the spatiotemporal regulation of reaction-diffusion waves in the tooth-forming field.^37^ A very recent study suggested that tooth germs exhibit polarization along the lingual-buccal axis, influencing tooth growth and cuspal patterning.^38^ However, only a few studies have systematically compared the lingual and buccal regions, especially in relation to mesenchymal compartmentalization. In this study, at the cap stage, the lingual mesenchyme showed an enrichment of GO associated with extracellular matrix (ECM) organization and morphogenesis, indicating its crucial role in establishing a structural framework for tooth development, including enamel organ and dental papilla formation. In addition, the lingual mesenchyme exhibited a high capacity for odontoblast differentiation, which is essential for dentin formation. In contrast, the buccal mesenchyme at the cap stage was enriched in GO terms related to stemness and regeneration, suggesting its involvement in maintaining progenitor populations and supporting tooth growth and regeneration. Our findings revealed the existence of distinct molecular signatures in these regions at different stages of tooth morphogenesis. These further suggest that the lingual and buccal regions of the dental mesenchyme may possess specialized capabilities in tooth development.

Tooth development follows a sequential pattern in the lingual region, as seen in reptiles with multiple tooth rows.^39^ In mammals, however, tooth formation is limited to a single row per jaw, regulated by opposing gradients along the lingual-buccal axis. Cuspal patterning begins with enamel knot formation on the buccal side and moves toward the lingual side.^18^ Since cuspal patterning is an epithelial-driven process,^7^ this study focuses on the mesenchymal compartment.

Morphogens, crucial for embryonic development, create concentration gradients that provide positional information during pattern formation.^12^ Retinoic acid influences positional cues during limb development, while Hox genes regulate tissue patterning, including skull development.^33,40^ Although teeth arise from the first branchial arch, their development is unaffected by Hoxa2 expression, suggesting distinct regulatory mechanisms for tooth formation.^41^ While there have been many studies on cuspal morphogenesis and gene expression in the lingual and buccal regions, the characteristics based on positional information in these regions have not been adequately studied. In this study, lingual dental mesenchymal cells cultured in vitro for 2 days at the cap stage showed high proliferative capacity for tooth formation, with upregulation of Wnt-associated genes (*Dkk2, Dkk3, Wnt11, Wif1, Apoe, Sfrp2*). In contrast, buccal dental mesenchymal cells cultured in vitro had greater osteogenic potential, with upregulation of BMP signaling genes (*Fst, Lefty1*). Lingual cells without buccal cells preferred Rspo1, to make the tooth, while buccal cells without lingual cells preferred Noggin to produce the surrounding tissue. The Rspo family, particularly *Rspo1*, was significantly upregulated in the lingual part, activating the Wnt pathway. BMP signaling is crucial for tooth development, with disruptions leading to dental abnormalities. Bmp inhibitors (*Grem1, Chordin, Noggin*) were upregulated in buccal cells. Our results show that the tissue designated for tooth formation is prespecified at the cap stage. This highlights a new mechanism in lingual-buccal tooth patterning, where the WNT/BMP pathway plays a key role in interpreting positional information during tooth development.

An in vitro reaggregation system, in which all mesenchymal cells are reset to an equivalent state and have the same probability of bud initiation, has been utilized in many model systems, such as feather and tooth patterning.^7,42^ In one of the previous studies, when a feather reconstitution assay was used for feather patterning, the patterning process was reverted to the initial state during which the mesenchymal cells self-organized from an equivalent state into periodic patterns.^42^ These dissociated mesenchymal cells were analyzed with modulation of morphogens, which can change the primordial size and spacing. Furthermore, the reaggregation of dental mesenchymal tissue was useful for studying the predetermination of the dental mesenchyme.^7^ In this study, we used a reaggregation system to identify the fate of dental mesenchymal cells. The tooth and its components namely, dentin and dental pulp were determined by the lingual part of tooth germs, and the surrounding tissue by the buccal part of tooth germs.

In conclusion, we proposed a model of dental mesenchymal cell patterning along the lingual-buccal axis for tooth and surrounding tissue formation. The characteristics of dental mesenchymal cells vary along the lingual-buccal axis, and the fate of the tooth and surrounding tissue formation is determined by dental mesenchymal cells via WNT/BMP signaling. These findings could be explored further in terms of their functional significance, particularly in tissue engineering and regenerative therapies, which may ultimately lead to advancements in stem cell-based tooth regeneration and more effective therapeutic applications for dental restoration and repair.

## Materials and methods

All experiments were approved by the Institutional Animal Use and Care Committee, Yonsei University College of Dentistry, and were performed in accordance with their guidelines (2023-0053).

### Animals

Adult ICR mice were housed in a temperature-controlled room (22 °C) under artificial illumination (lights on from 05:00 to 17:00) at a relative humidity of 55% with access to food and water ad libitum. The embryos were obtained from time-mated pregnant mice. Embryonic day 0 (E0) was designated as the day on which a vaginal plug was confirmed. First mandible molar tooth germs of ICR mice at E13 for the cap stage, and E15 for the bell stage were used. For reaggregation experiment of dental mesenchymal cells, Wnt1^CreERT2^ were bred with R26^flox-STOP-tdTomato^ mice to generate Wnt1^CreERT2/+^; R26R^Tom/+^. In these mice, dental mesenchymal cells expressing Tdtomato under the control of Wnt1. They were maintained on a C57BL/6 background. Cre-mediated recombination was induced in mice by intraperitoneal injection of tamoxifen (T5648, Sigma, and St. Louis, MO) at a dose of 100 mg/kg at E10.

### Tooth split in lingual-buccal direction

The tooth germs from mouse mandible at cap and bell stage were divide two region with lingual and buccal part with 30 gauge fine needle, and were analyzed for RNA sequencing, immunofluorescence, in situ hybridization, and kidney transplantation. 50 samples were used for in in situ hybridization and immunofluorescence, and 10 ~ 15 samples were used for kidney transplantation, respectively.

### In situ hybridization

Tissues were fixed overnight in 4% paraformaldehyde (PFA). The hybridizations were performed on the limb buds with digoxigenin-labeled RNA probes in hybridization buffer for 18 h at 72 °C, and the hybridization signals were detected using alkaline phosphatase-conjugated, anti-digoxigenin antibodies with a nitro blue tetrazolium chloride/5-bromo-4-chloro-3-indolyl phosphate toluidine salt substrate (Roche, Mannheim, Germany).

### Histology

The samples were fixed with 4% paraformaldehyde (PFA) for fixation. Paraffin-embedded specimens were sectioned into 5 µm thick sections. Hematoxylin and eosin (HE) staining was performed after deparaffinization. For immunological staining of histological sections, the following antibodies were used, as previously described.^43^ The antibodies for immunofluorescent staining were: Amgn (SantaCruz, USA), DSP (SantaCruz, USA), Fibrillin (Invitrogen, USA), PCNA (Abcam, UK), cleaved Caspase-3 (cCaspase-3; Cell signaling). BrdU (Abcam, UK), IdU (Invitrogen, USA).

The sections were counterstained with TO-PRO3 (Molecular probes, USA). For visualization, anti-mouse or rabbit IgG conjugated with Alexa Fluor 488 or 555 dye (Invitrogen, USA) was applied and observed under a confocal microscope (DMi8, Leica, Germany or C1si, Nikon, Japan).

### Reaggregation and recombination

20 of tooth germs were dissected from mandible at cap stage, dental epithelium and mesenchyme were isolated, made single cell. Furthermore, 1.25 × 10^5^ of buccal mesenchymal cells from tooth germs of Wnt1^creERT/+^;R26R^Tom/+^ mice were obtained, and reaggregated with 1.25 × 10^5^ of lingual mesenchymal cells from tooth germs of WT mice. Or same cells of buccal mesenchyhmal cells from WT mice were reaggregated with 1.2 × 10^5^ of lingual mesenchymal cells from tooth germs of Wnt1^creERT/+^;R26R^Tom/+^ mice. The reaggregated dental mesenchymal cells were recombined with dental epithelium from WT mice, and incubated 1week underneath the kidney capsule.

### RNA sequencing

36 embryos from 3 pregnant mice were obtained. The tooth germs were isolated from the mandibular M1 tooth germs at the cap and bell stage, were divded into lingual and buccal part. The only dental mesenchyme were isolated from each part, respectively. RNA was extracted by TRIzol. mRNA libraries were prepared using TruSeq Stranded mRNA Preparation kit (Illumina, CA, USA) according to the manufacturer’s instruction. RNA-seq was performed with triplicate using Illumina HiSeq2500 sequencing platform. Cutadapt (version 2.8) was used to trim adaptor sequence and discard low quality reads. Two biological replicates for each species were performed with RNA-seq, and were analyzed. The trimmed and fitered reads were mapped on reference genome GRCM Reference,^39^ INSDC Assembly GCA_000001635.9 for mouse) using STAR version 2.7.1a.^44^ The expression level of genes and transcripts were calculated using Cufflinks version 2.2.1.^45^ DEGs were identified using DESeq version 1.44.0^46^ with *p*-Value < 0.05 and |fold change | > 1 threshold. Volcano plots, violin plots, cnet plots and dot plots were generated using ggplot2 (version 3.4.1). Gene set analysis of significant DEGs for GO terms were performed using clusterProfiler (version 4.0.4), a R package for interpretation of omics data.^47^ Visaulization of DEGs on a map of signaling pathway was performed using Pathview version 1.30.1, a R package for pathway based data integration and visualization.^48^ The RNA-seq data have been deposited in the Gene Express Omnibus (GEO) database [GEO: GSE292940].

### Transcriptomic analysis

Raw sequence reads produced by the sequencer were cleaned using FastQC version 0.11.7. The cleaned reads were aligned on mouse reference genome (GRCm39.107) and quantified using Subread version 2.0.0.^49^ Differentially expressed genes (DEGs) were identified using DESeq2 version 1.46.0^50^ with the *P* value < 0.05 and fold change >1 threshold for significance. GO terms, Kyoto Encyclopedia of Genes and Genomes (KEGG) pathways, and hall mark gene sets (H, MSigDB collections, BROAD Institute) enriched on the significant DEGs were accessed using clusterProfiler version 4.14.4.^47^ The KEGG pathway map colored by fold change of DEGs were generated using Pathview version 1.46.0.^48^ Enrichment scores of each sample on gene sets and cell markers were calculated using GSEABase version 1.68.0 Volcano plots, bar plots, box plots, dot plots and heatmaps were generated using ggplot2 (version 3.5.1) and ComplexHeatmap (version 2.22.0). All statistical analysis and visualizations were performed under R (version 4.4.1) and RStudio environment.

### Chemotaxis assay

The chemotaxis assay was performed using a microslide-based system to evaluate the directed migration of dental mesenchymal cells in response to specific proteins. Lingual (stained with CellTracker^TM^ Green, Thermofisher, USA) or Buccal (stained with CellTracker^TM^ Red) part of dental mesenchymal cells isolated from tooth germ at cap stage were cultured in DMEM with 10% FBS at 37 °C and 5%CO_2_. A microslide chemotaxis chamber (Ibidi, Germany) was used. For the assay, R-spondin1 conditioned medium (Rspo1 CM) and Noggin were used as chemoattractants. Each cell was seeded into the microslide chamber and allowed to adhere for 2 h before the introduction of the chemoattractant. Time-lapse imaging was conducted at 37 °C with 5% CO₂ equipped with a live-cell imaging system (CQ1, Yokogawa, Japan). Cell migration was tracked and analyzed using ImageJ software with the Chemotaxis and Migration Tool plugin.

### RT-qPCR

For quantification of the level of RNA, the cells were harvested from each group using TRIzol reagent (Invitrogen, NY, USA). RT-qPCR was performed using a Thermal Cycler Dice^TM^ Real Time System and SYBR premix EX Taq^TM^ (Takara, Japan) according to the manufacturer’s instruction. For RT-qPCR, the reaction mixture was initially incubated for one min at 95 °C. The amplification program consisted of 40 cycles of denaturation at 95 °C for 5 s, annealing at 55–60 °C for 10 s, and extension at 72 °C for 10 s. The RT-qPCR analysis of each sample was performed in triplicate, and the amount of each of the RT-qPCR products was normalized using β-2-microglobulin as an internal control. The primers used for amplification were as Table 1:

**Table 1:** Primer sequences

	Forward (5′-3′)	Reverse (5′-3′)
*B2m*	GGGAAGCCGAACATACTGAA	TCACATGTCTCGATCCCAGT
*Alx1*	GATGAGTGAAATGTCAGCCCC	TTAGGGCTTCTTGGGCGATT
*Tbx15*	GGGCTGCCAGCTCAGTAG	TGAGCCGATCAAGGCTTCAA
*Hand1*	GAGGAGAGGAAAGGACGCAG	CTCGGCGGGAAGTGAACATA
*Tbx18*	GTGTTAGTCCTCTCGGGCTC	TCCTCTTTCTCTGGCGTTGG
*Col4a2*	CGCCTGGTACAAAAACCTCCA	CCGTGATAAAGTGCGTGCCA
*Mmp13*	GCCATTACCAGTCTCCGAGG	GGTCACGGGATGGATGTTCA
*IFITM5*	CTGGTCTGTCTTCAGCACGA	CCGCAGAGTCTTTGGCTAAC
*Hgf*	TGACCTGCAATGGTGAAAGC	GGGTCAAGAGTGTAGCACCA
Mmp9	AGACCTGAAAACCTCCAACCT	CGAATGGCCTTTAGTGTCTGG
*Hand2*	GGAAGGCGAGATGAGTCTGG	CATCTCCGGGTGGCCAATAA
*Tgfb2*	CAGGAGTGGCTTCACCACAAA	TGGCATATGTAGAGGTGCCAT
*Gremlin1*	GAAAGGTTCCCAAGGAGCCA	TCGCTTCAGATACTTGCGCT
*Osr2*	TCACCTACGGGATCACAGGT	TGTGGGACATTTGTGTGGAG
*Thy1*	CACCAAGGATGAGGGCGACTA	GCTTATGCCGCCACACTTGA
*Sema3c*	CGTCCGGGTGATTCAGACAT	AGCATCGTCCTTTGCCAGAT
*Sox10*	AACCACCCCAAAGACAGAGC	TTGGGTGGCAGGTATTGGTC
*Posn*	CGAATCATTACAGACACACCTGC	AACGGCCTTCTCTTGATCGT
*Col8a*	CGGGTGGTAAAGGGGAACAA	CCCAGGAACACCCCCAATAC
*Bmp2*	TCAGCATGTTTGGCCTGAAGCA	GCCGTTTTCCCACTCATCTCTGGAA
*Ostn*	GCTTCAACTGTGTCAGAAGGC	TCACCCCTAGGCTGGTAGAA
*Hand2*	CCAGCTACATCGCCTACGTC	TTGCTGCTCACTGTGCTTTT
*Dll1*	TCCACATTGTCCTCGCAGTA	ACGGACATTGAGGACAGCTT
*Myod1*	GACTGCCTGTCCAGCATAGT	TTCCCTGTTCTGTGTCGCTT
*Myog*	AGCCCCCTTGTTAATGTCCC	GTCAGGGCACTCATGTCTCT
*Hoxd4*	AGTGCAGATGCTTGGTTCCT	CAGGGCACTCATGTCCATCT
*Hoxd8*	CCTGACTGTAAATCGTCCAGTGGTA	AGTTTGGAAGCGACTGTAGGTTTG

### MicroCT

Three-dimensional reconstructed computed tomography images were obtained by scanning calcified bone using micro-computed tomography (Micro-CT, Skyscan 1076, Skyscan, Antwerp, Belgium). The data were then digitalized using a frame grabber, and the resulting images were transmitted to a computer for analysis using Comprehensive TeX Archive Network (CTAN) topographic reconstruction software.

### Statistical analysis

All results are presented as the means and standard deviations of at least five independent experiments for in vitro assays and at least five independent experiments for in vivo analysis. Comparisons between two groups were analyzed using Student’s *t* test. All statistical analyses were performed in GraphPad Prism 9 software. All graphs are presented as means ± standard deviations. *, **, and *** describe *P* values of <0.05, 0.01, and 0.001, respectively.

## Supplementary information

Supplementary Figures
