# Supplementary material for: Prespecified dental mesenchymal cells for the making of a tooth
Source: Int J Oral Sci. 2025 Oct 9;17:67. doi: 10.1038/s41368-025-00391-7 (PMC12511381; doi:10.1038/s41368-025-00391-7)
Supplement: Supplementary file 1 — Supplementary Figures [file 41368_2025_391_MOESM1_ESM.docx]

**Prespecified dental mesenchymal cells for the making of a tooth**

**Eun-Jung Kim ^a^, Hyun-Yi Kim ^b^, Suyeon Lee ^a^, Junsu Kim ^a^, Shujin Li ^a^, Anish Ashok Adpaikar ^a^, Thantrira Porntaveetus ^c^, Senthil Kumar Baskaran ^a^, Jong-Min Lee ^a^,**

**Han-Sung Jung^† a, c^**

^a^ Division in Anatomy and Developmental Biology, Department of Oral Biology, Taste Research Center, Oral Science Research Center, BK21 FOUR Project, Yonsei University College of Dentistry, Seoul, Korea

^b^ NGeneS Inc., Ansan-si, Korea

^c^ Center of Excellence in Precision Medicine and Digital Health, Geriatric Dentistry and Special Patients Care, Department of Physiology, Faculty of Dentistry, Chulalongkorn University, Bangkok 10330, Thailand

**Supplementary Figures**

**
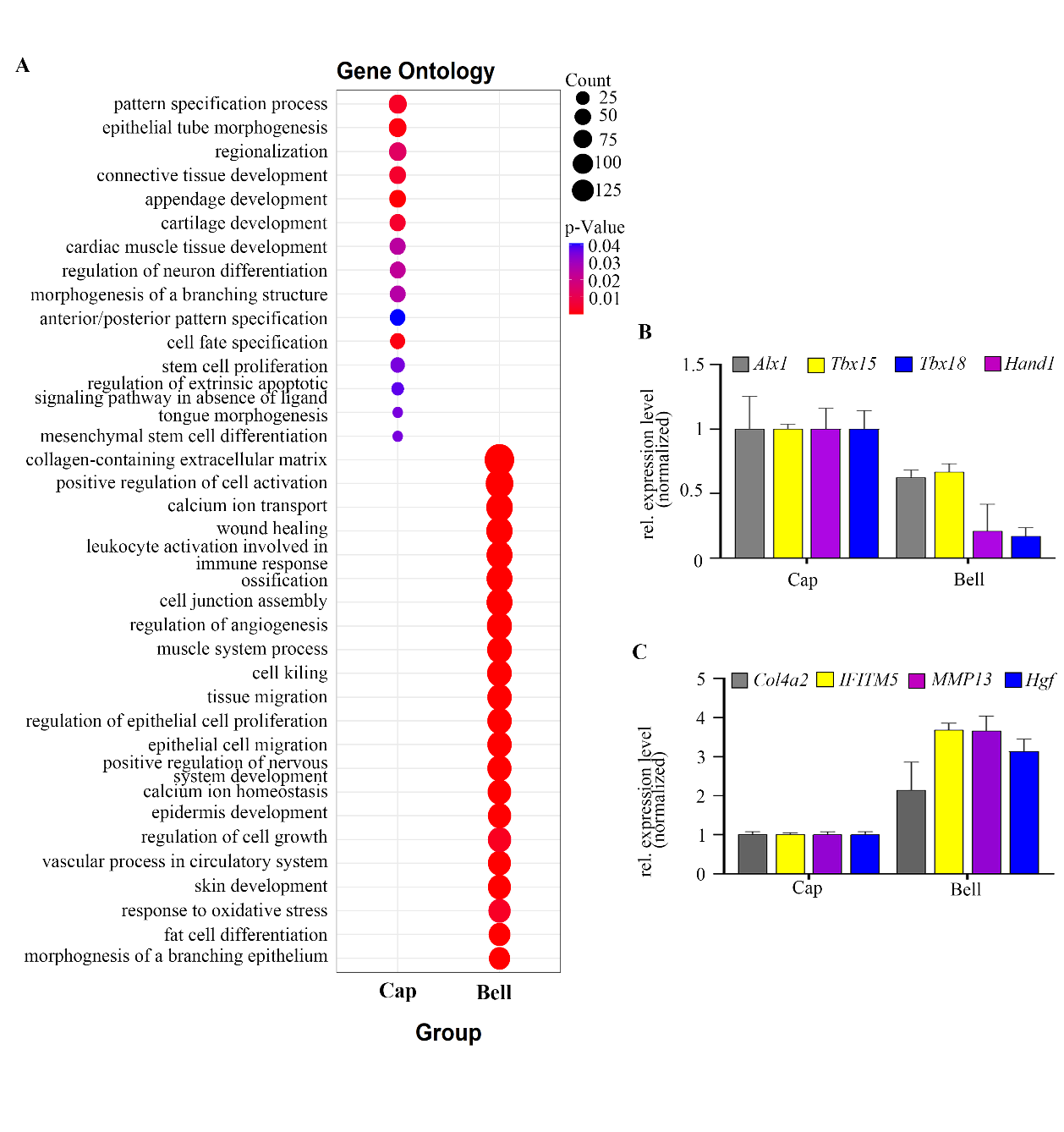
**

**Supplementary Figure 1. Dot plots representing GO term enrichment analysis of upregulated differentially expressed genes (DEGs) at the cap and bell stages and validation of upregulated genes at cap and bell stage** (A) GO terms enriched in the mesenchyme at cap and bell stage. (B, C) The genes which were enriched in the dental mesenchyme at cap (A) and bell (B) stage were validated in RT-qPCR. (**p* < 0.1, ***p* < 0.01; ****p* < 0.001). Data are presented as means ± standard deviations (SD)


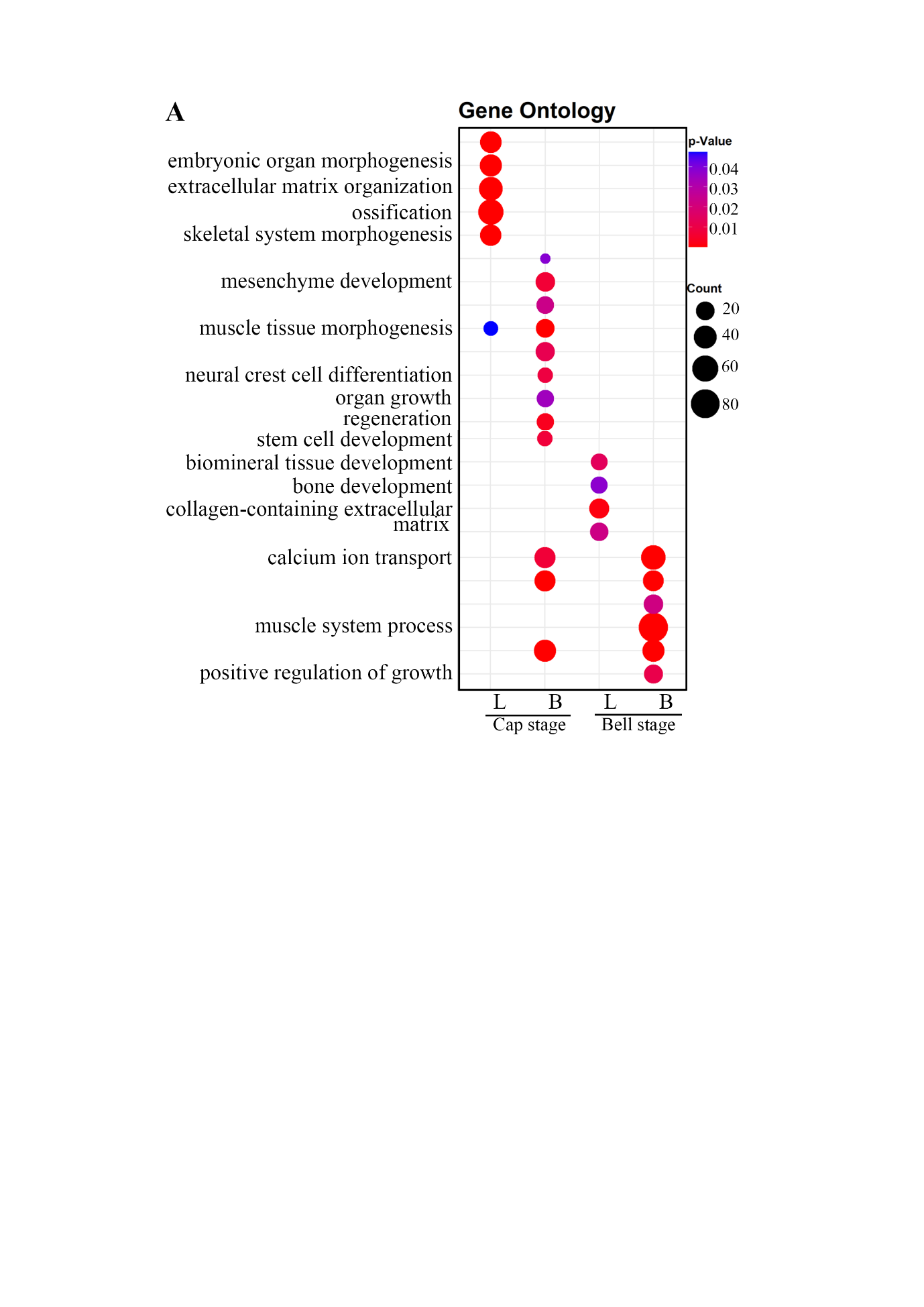


**Supplementary Figure 2. Dot plots representing GO term enrichment analysis of upregulated differentially expressed genes in the lingual part of the dental mesenchyme compared to the buccal part at the cap and bell stages.** (A) GO terms enriched in the lingual mesenchyme at the cap stage include embryonic organ morphogenesis, extracellular matrix organization, ossification, and skeletal system morphogenesis. In contrast, GO terms enriched in the buccal mesenchyme include mesenchyme development, muscle tissue development, neural crest differentiation, organ growth, regeneration, and stem cell development.

**Table1. Upregulated DEGs of whole tooth germs at cap stage than at bell stage**

|  | SYMBOL | log2FoldChange | pvalue |
| --- | --- | --- | --- |
| 16726 | Hand2os1 | -9.28576 | 1.87E-08 |
| 16725 | Hand2 | -8.36428 | 7.33E-10 |
| 10453 | Gm20594 | -5.7707 | 0 |
| 16763 | Hbb-y | -5.73063 | 1.05E-92 |
| 16810 | Hdnr | -5.34833 | 5.58E-05 |
| 24146 | Pax3 | -5.23878 | 1.84E-16 |
| 16759 | Hbb-bh1 | -4.80976 | 5.17E-48 |
| 16757 | Hba-x | -4.68738 | 4.49E-90 |
| 25385 | Prok2 | -4.42577 | 6.28E-05 |
| 29463 | Tbx15 | -4.33234 | 1.04E-59 |
| 17059 | Hpse2 | -4.25824 | 0.001347 |
| 2146 | Alx1 | -4.1857 | 0.000224 |
| 13693 | Gm34184 | -4.14041 | 0.000366 |
| 7450 | Fgf8 | -4.10828 | 3.13E-08 |
| 16724 | Hand1 | -3.96154 | 8.98E-05 |
| 12844 | Gm25890 | -3.91131 | 0.004362 |
| 27030 | Rxfp2 | -3.72039 | 8.49E-19 |
| 2147 | Alx3 | -3.69116 | 0.004497 |
| 27029 | Rxfp1 | -3.53909 | 3.38E-09 |
| 2148 | Alx4 | -3.40189 | 9.95E-08 |
| 22279 | Nr5a2 | -3.32859 | 5.98E-40 |
| 4851 | Cnnm1 | -3.26262 | 7.52E-10 |
| 29464 | Tbx18 | -3.13106 | 2.85E-15 |
| 17889 | Isl1 | -3.09637 | 1.59E-05 |
| 24402 | Peg10 | -3.0428 | 4.21E-40 |
| 16520 | Gucy2f | -2.9395 | 1.62E-05 |
| 18712 | Ldoc1 | -2.91732 | 5.59E-06 |
| 26380 | Rmst | -2.83341 | 1.12E-06 |
| 4955 | Col9a1 | -2.82776 | 1.12E-12 |
| 20414 | Mir6239 | -2.8189 | 0.047189 |
| 5256 | Cst8 | -2.80194 | 0.048201 |
| 18155 | Kcns2 | -2.76231 | 5.69E-11 |
| 10184 | Gm18981 | -2.75138 | 1.53E-07 |
| 30892 | Tspan8 | -2.73265 | 4.55E-19 |
| 4935 | Col2a1 | -2.72396 | 1.90E-18 |
| 24695 | Pkhd1l1 | -2.68876 | 0.000728 |
| 28176 | Slitrk5 | -2.67992 | 1.83E-17 |
| 4847 | Cnmd | -2.64256 | 3.67E-10 |
| 29469 | Tbx22 | -2.59238 | 1.65E-20 |
| 17106 | Hsd3b6 | -2.58149 | 5.01E-07 |
| 28807 | Spsb4 | -2.56751 | 1.98E-20 |
| 27642 | Shisa3 | -2.56169 | 8.37E-09 |
| 17783 | Insm1 | -2.55107 | 0.001017 |
| 25971 | Rasef | -2.48023 | 0.002792 |
| 27251 | Scn3a | -2.41431 | 1.43E-17 |
| 17890 | Isl2 | -2.36131 | 0.000575 |
| 28175 | Slitrk4 | -2.34661 | 4.96E-10 |
| 552 | 2810442N19Rik | -2.32007 | 3.15E-11 |
| 3073 | BC006965 | -2.31972 | 0.000586 |
| 29471 | Tbx3os1 | -2.27128 | 1.00E-19 |
| 17067 | Hrh2 | -2.24741 | 0.002562 |
| 8114 | Glra3 | -2.15377 | 0.00282 |
| 27081 | Sall4 | -2.13026 | 6.68E-05 |
| 2662 | Asb4 | -2.12668 | 1.98E-21 |
| 29579 | Tecta | -2.11048 | 0.005597 |
| 9712 | Gm17938 | -2.11038 | 0.001296 |
| 3688 | Car14 | -2.09564 | 1.14E-11 |
| 524 | 2610318N02Rik | -2.07198 | 7.70E-09 |
| 7628 | Foxp2 | -2.03978 | 2.02E-10 |
| 30816 | Trpc5 | -2.03384 | 0.001212 |
| 26166 | Ret | -2.01037 | 1.88E-05 |
| 18252 | Kif26b | -2.00159 | 1.47E-17 |
| 32219 | Wnt2 | -1.9933 | 1.56E-09 |
| 16683 | H3c3 | -1.97682 | 0.000637 |
| 32002 | Vnn1 | -1.96952 | 0.000835 |
| 31365 | Usp44 | -1.96482 | 2.41E-05 |
| 24389 | Pdzk1ip1 | -1.95202 | 0.003445 |
| 23945 | Otogl | -1.94112 | 5.06E-05 |
| 1759 | Adam23 | -1.93995 | 1.85E-14 |
| 21503 | Mybpc1 | -1.9338 | 0.002399 |
| 30265 | Tnn | -1.91486 | 1.44E-16 |
| 14819 | Gm5860 | -1.90333 | 0.00041 |
| 17667 | Il1r2 | -1.89873 | 6.10E-06 |
| 8574 | Gm12248 | -1.88301 | 0.003711 |
| 3625 | Caln1 | -1.88283 | 7.92E-07 |
| 17653 | Il17rb | -1.87141 | 0.000604 |
| 15832 | Gm8934 | -1.86614 | 0 |
| 22270 | Nr2f1 | -1.86318 | 8.42E-12 |
| 7563 | Fnd3c2 | -1.86164 | 0.000508 |
| 14472 | Gm50045 | -1.8568 | 0.003178 |
| 3269 | Bmpr1b | -1.85579 | 2.16E-05 |
| 7569 | Fndc3c1 | -1.85419 | 5.87E-20 |
| 22449 | Nwd2 | -1.84998 | 7.48E-06 |
| 25803 | Rab17 | -1.84661 | 0.006092 |
| 22181 | Nos1 | -1.84536 | 2.34E-09 |
| 6036 | Dlk1 | -1.81757 | 9.66E-11 |
| 29025 | Stk32b | -1.79854 | 2.32E-16 |
| 18344 | Klhl34 | -1.79454 | 4.00E-08 |
| 17324 | Igf2bp1 | -1.78558 | 6.38E-12 |
| 19627 | Meis2 | -1.77175 | 6.31E-07 |
| 2706 | Astn1 | -1.7372 | 9.16E-12 |
| 1786 | Adamts15 | -1.72961 | 1.28E-15 |
| 26983 | Rtl3 | -1.7231 | 4.01E-12 |
| 15244 | Gm7289 | -1.69795 | 0.001427 |
| 30087 | Tmem26 | -1.68729 | 1.43E-15 |
| 24676 | Pitx1 | -1.67426 | 1.03E-07 |
| 9305 | Gm15267 | -1.67423 | 0.003556 |
| 22354 | Ntf3 | -1.66582 | 7.06E-10 |
| 26240 | Rgs9 | -1.66259 | 2.18E-10 |
| 18795 | Lin28b | -1.66162 | 8.18E-06 |
| 1787 | Adamts16 | -1.64652 | 0 |
| 5007 | Corin | -1.62684 | 1.02E-09 |
| 17789 | Insyn2b | -1.61665 | 0.002572 |
| 21787 | Ncam2 | -1.61494 | 7.96E-07 |
| 27635 | Shcbp1l | -1.61039 | 0.004168 |
| 29240 | Syt14 | -1.59962 | 3.48E-08 |
| 6459 | Ebf2 | -1.59593 | 2.76E-08 |
| 29227 | Synpo2 | -1.57682 | 2.30E-12 |
| 6049 | Dlx6 | -1.57362 | 5.49E-10 |
| 4564 | Chrdl2 | -1.56746 | 2.50E-05 |
| 17077 | Hs3st3a1 | -1.54909 | 1.24E-07 |
| 29216 | Syne4 | -1.54674 | 3.50E-09 |
| 6930 | Esrrg | -1.54055 | 0.000314 |
| 22293 | Nrg1 | -1.52865 | 8.58E-05 |
| 6759 | Enpep | -1.51973 | 1.09E-11 |
| 24180 | Pcdh11x | -1.51341 | 6.85E-05 |
| 16558 | H1f4 | -1.51255 | 0 |
| 9577 | Gm16853 | -1.51141 | 0.005806 |
| 22015 | Ngf | -1.50153 | 7.81E-06 |
| 28897 | Ssc4d | -1.48922 | 6.59E-07 |
| 27503 | Sertm1 | -1.48 | 2.99E-05 |
| 28592 | Sox6 | -1.47231 | 1.02E-07 |
| 32495 | Zfhx4 | -1.47148 | 2.94E-09 |
| 24283 | Pcsk5 | -1.46461 | 3.28E-12 |
| 1445 | A830082K12Rik | -1.46009 | 1.56E-05 |
| 3276 | Bnc2 | -1.45887 | 7.67E-08 |
| 32847 | Zfp941 | -1.45722 | 0.000101 |
| 27247 | Scn1a | -1.45302 | 3.01E-06 |
| 4845 | Cnksr2 | -1.44928 | 2.00E-08 |
| 32643 | Zfp462 | -1.44658 | 3.17E-13 |
| 16901 | Hipk2 | -1.43092 | 2.88E-10 |
| 7297 | Fbxl22 | -1.4296 | 0.000121 |
| 30957 | Ttc6 | -1.41691 | 0.004434 |
| 16361 | Greb1l | -1.41684 | 7.86E-07 |
| 24332 | Pde7b | -1.40996 | 1.40E-11 |
| 26317 | Rhpn2 | -1.40654 | 4.19E-05 |
| 32494 | Zfhx3 | -1.4013 | 2.77E-11 |
| 21065 | Mn1 | -1.39961 | 2.67E-12 |
| 3432 | Btn2a2 | -1.39565 | 0.003262 |
| 5560 | Cysltr1 | -1.38713 | 0.000218 |
| 18075 | Kcnb1 | -1.36922 | 1.19E-07 |
| 29601 | Tenm4 | -1.36357 | 3.45E-12 |
| 2173 | Amot | -1.36138 | 4.81E-11 |
| 29470 | Tbx3 | -1.35314 | 7.13E-10 |
| 1736 | Acvr2b | -1.33665 | 1.02E-05 |
| 16928 | Hmga2 | -1.33251 | 4.98E-13 |
| 16459 | Gstm7 | -1.32299 | 1.05E-06 |
| 7010 | Eya1 | -1.31686 | 6.44E-11 |
| 21024 | Mlxipl | -1.31197 | 0.000401 |
| 24496 | Phactr1 | -1.30559 | 0.000775 |
| 24485 | Pgm2l1 | -1.30471 | 1.17E-07 |
| 7126 | Fam169a | -1.30307 | 7.95E-07 |
| 5661 | Dapk1 | -1.30247 | 1.89E-11 |
| 1958 | Agtr1a | -1.2892 | 0.000137 |
| 19573 | Mecom | -1.28866 | 2.94E-06 |
| 32212 | Wnk3 | -1.2862 | 1.44E-05 |
| 26922 | Rragb | -1.2791 | 0.000196 |
| 29961 | Tmem132b | -1.2785 | 9.31E-07 |
| 31349 | Usp29 | -1.27724 | 5.06E-07 |
| 28173 | Slitrk2 | -1.27605 | 8.93E-06 |
| 26990 | Rtl9 | -1.2758 | 5.58E-07 |
| 31267 | Unc5c | -1.27278 | 1.10E-07 |
| 28576 | Sox11 | -1.26653 | 2.40E-08 |
| 3776 | Cbln4 | -1.26595 | 0.004172 |
| 27718 | Six4 | -1.2631 | 1.98E-10 |
| 13 | 0610040J01Rik | -1.25589 | 0.004045 |
| 5606 | D430041D05Rik | -1.25384 | 0.001594 |
| 21544 | Myl4 | -1.25299 | 3.66E-05 |
| 27282 | Scx | -1.24772 | 0.000171 |
| 17323 | Igf2 | -1.24729 | 4.39E-08 |
| 7013 | Eya4 | -1.2412 | 3.85E-08 |
| 2845 | Atp7b | -1.23702 | 0.000832 |
| 1793 | Adamts3 | -1.23263 | 5.47E-06 |
| 26760 | Rprm | -1.22875 | 0.000378 |
| 4931 | Col25a1 | -1.21891 | 1.01E-07 |
| 26451 | Rnf182 | -1.20968 | 1.18E-08 |
| 6031 | Dlgap1 | -1.20551 | 0.001667 |
| 27802 | Slc16a8 | -1.18604 | 0.007046 |
| 18828 | Lix1 | -1.17726 | 0.000365 |
| 14349 | Gm4811 | -1.1752 | 0.00155 |
| 7488 | Fign | -1.15699 | 4.63E-06 |
| 16299 | Gpr37 | -1.14804 | 0.001733 |
| 27509 | Setbp1 | -1.14632 | 5.38E-09 |
| 1824 | Adcy8 | -1.14328 | 1.94E-07 |
| 29625 | Tesl1 | -1.14093 | 0.005232 |
| 21990 | Nfia | -1.13532 | 5.90E-08 |
| 24706 | Pknox2 | -1.13414 | 0.000934 |
| 18296 | Klf12 | -1.12424 | 8.24E-06 |
| 26321 | Ric3 | -1.11651 | 0.001021 |
| 30202 | Tmtc1 | -1.11598 | 4.26E-06 |
| 1790 | Adamts19 | -1.1148 | 9.88E-06 |
| 21561 | Myo16 | -1.11343 | 0.000212 |
| 19680 | Mex3b | -1.11337 | 5.11E-07 |
| 28596 | Sox9 | -1.11079 | 0.003225 |
| 24183 | Pcdh17 | -1.10906 | 4.62E-07 |
| 32196 | Wfikkn2 | -1.10819 | 7.83E-07 |
| 24189 | Pcdh9 | -1.10818 | 0.000142 |
| 22154 | Nod2 | -1.09913 | 0.000985 |
| 14999 | Gm6392 | -1.09496 | 0.00629 |
| 26386 | Rn7sk | -1.08713 | 0 |
| 17879 | Irx3 | -1.08588 | 0.000774 |
| 21139 | Mpped2 | -1.08583 | 5.81E-06 |
| 28830 | Srcap | -1.08238 | 0.00064 |
| 18805 | Lingo2 | -1.0813 | 0.00037 |
| 5439 | Cyp26b1 | -1.07995 | 4.95E-08 |
| 1438 | A830018L16Rik | -1.06002 | 0.003446 |
| 6808 | Epha4 | -1.05579 | 1.26E-07 |
| 16883 | Hic2 | -1.04864 | 0.000136 |
| 5396 | Cxxc4 | -1.04802 | 7.19E-05 |
| 18246 | Kif21b | -1.04628 | 2.22E-05 |
| 28177 | Slitrk6 | -1.04574 | 4.56E-05 |
| 29619 | Tes | -1.04008 | 3.56E-05 |
| 7743 | Fxyd7 | -1.03553 | 0.00058 |
| 8741 | Gm12904 | -1.03166 | 0.004761 |
| 28062 | Slc4a8 | -1.02848 | 0.000416 |
| 27413 | Septin3 | -1.02753 | 1.24E-05 |
| 25453 | Prrc2c | -1.02639 | 7.49E-08 |
| 32938 | Zkscan2 | -1.02544 | 0.000212 |
| 6811 | Epha7 | -1.0225 | 1.70E-06 |
| 16224 | Gpc6 | -1.02102 | 2.44E-05 |
| 5943 | Dgki | -1.02094 | 0.005804 |
| 17842 | Iqgap2 | -1.02067 | 6.44E-07 |
| 3142 | Bcl11a | -1.01039 | 8.29E-06 |
| 19250 | Mab21l2 | -1.00829 | 1.19E-05 |
| 30332 | Tox3 | -1.00721 | 0.000107 |
| 4312 | Cecr2 | -1.00693 | 0.002707 |
| 17602 | Iglon5 | -1.00214 | 0.000155 |
| 2551 | Arid5b | -1.00054 | 1.64E-07 |
| 6693 | Elovl2 | -0.99816 | 0.000634 |
| 3668 | Capn6 | -0.99609 | 1.01E-06 |
| 19491 | Mbnl3 | -0.98808 | 0.002594 |
| 4626 | Cilp | -0.98542 | 0.005784 |
| 14801 | Gm5815 | -0.98519 | 0.000271 |
| 26381 | Rn18s | -0.98427 | 0 |
| 16989 | Hook1 | -0.98142 | 0.00308 |
| 24339 | Pdgfc | -0.97417 | 1.90E-06 |
| 3163 | Bcl9 | -0.97353 | 9.79E-07 |
| 24186 | Pcdh20 | -0.96649 | 0.00149 |
| 29679 | Tfap2c | -0.96301 | 1.89E-06 |
| 30212 | Tnc | -0.95877 | 3.32E-07 |
| 27939 | Slc2a13 | -0.95236 | 4.35E-05 |
| 17739 | Inava | -0.9447 | 0.000593 |
| 3041 | Batf | -0.94034 | 0.001102 |
| 1855 | Adgrl2 | -0.92538 | 1.36E-06 |
| 17881 | Irx5 | -0.91452 | 0.005034 |
| 25462 | Prrx1 | -0.911 | 3.78E-05 |
| 5159 | Crnde | -0.90997 | 0.002382 |
| 27641 | Shisa2 | -0.90289 | 4.27E-06 |
| 18586 | L3mbtl3 | -0.90233 | 1.32E-05 |
| 32624 | Zfp423 | -0.90104 | 5.50E-05 |
| 6697 | Elovl6 | -0.89813 | 2.03E-05 |
| 19332 | Maml3 | -0.89764 | 6.11E-06 |
| 3013 | Bach2 | -0.89678 | 0.00033 |
| 18753 | Lgr5 | -0.89642 | 6.46E-05 |
| 25152 | Ppp1r9a | -0.89322 | 0.001525 |
| 7630 | Foxp4 | -0.8926 | 0.000733 |
| 32088 | Wasf1 | -0.89112 | 0.00274 |
| 3569 | Cacna1d | -0.89067 | 0.005648 |
| 32678 | Zfp568 | -0.88773 | 2.21E-05 |
| 17327 | Igf2os | -0.88548 | 0.001357 |
| 2899 | Auts2 | -0.88494 | 2.67E-05 |
| 29214 | Syne2 | -0.88326 | 1.05E-05 |
| 17083 | Hs6st2 | -0.88291 | 5.92E-05 |
| 15612 | Gm8291 | -0.87346 | 0.003492 |
| 6489 | Edaradd | -0.87294 | 0.006863 |
| 2977 | B3gnt5 | -0.87036 | 0.000391 |
| 27132 | Satb2 | -0.86985 | 6.77E-05 |
| 30291 | Tob1 | -0.86274 | 0.001828 |
| 5715 | Dclk3 | -0.86017 | 0.000162 |
| 28318 | Snai2 | -0.85905 | 3.44E-05 |
| 17326 | Igf2bp3 | -0.85881 | 1.81E-05 |
| 24405 | Peg3 | -0.85854 | 0.000249 |
| 18245 | Kif21a | -0.85281 | 1.09E-05 |
| 1377 | A330074K22Rik | -0.85217 | 0.004092 |
| 3022 | Bahcc1 | -0.85117 | 0.000192 |
| 28758 | Spock1 | -0.85087 | 1.81E-05 |
| 29983 | Tmem151b | -0.85003 | 0.000476 |
| 28942 | St6gal2 | -0.84988 | 0.001764 |
| 7469 | Fhip1a | -0.84946 | 0.004782 |
| 27279 | Scube1 | -0.8403 | 7.75E-05 |
| 26804 | Rps15a | -0.83997 | 9.51E-06 |
| 7843 | Galnt5 | -0.8398 | 0.001273 |
| 22203 | Npas3 | -0.83796 | 0.000689 |
| 3597 | Cadm2 | -0.83772 | 0.004208 |
| 32376 | Zbtb12 | -0.83647 | 0.000374 |
| 1792 | Adamts20 | -0.83342 | 0.005662 |
| 18265 | Kif5c | -0.8334 | 0.001075 |
| 8093 | Gli2 | -0.83284 | 1.90E-05 |
| 27131 | Satb1 | -0.82739 | 0.001142 |
| 29600 | Tenm3 | -0.82721 | 1.86E-05 |
| 25062 | Ppargc1a | -0.82551 | 0.005894 |
| 32421 | Zc3h12c | -0.82512 | 0.001346 |
| 25103 | Ppm1l | -0.82199 | 5.99E-05 |
| 16360 | Greb1 | -0.82075 | 0.00154 |
| 18698 | Ldb2 | -0.82022 | 0.000213 |
| 27728 | Skida1 | -0.81737 | 7.50E-05 |
| 5356 | Cul4b | -0.81477 | 0.000745 |
| 16906 | Hivep1 | -0.8146 | 2.47E-05 |
| 16986 | Homer2 | -0.81415 | 0.000701 |
| 28604 | Sp4 | -0.8132 | 0.000841 |
| 7434 | Fgf13 | -0.81075 | 9.03E-05 |
| 1796 | Adamts6 | -0.81027 | 0.000739 |
| 5778 | Ddx25 | -0.808 | 0.00203 |
| 27016 | Runx1t1 | -0.8065 | 0.001934 |
| 31386 | Utp14b | -0.80182 | 0.001552 |
| 24799 | Plekha7 | -0.80068 | 0.005438 |
| 28952 | St8sia2 | -0.79813 | 0.002324 |
| 18786 | Limch1 | -0.79728 | 0.00017 |
| 27715 | Six2 | -0.79485 | 0.000117 |
| 21934 | Nek3 | -0.79355 | 0.005428 |
| 26886 | Rps6ka6 | -0.79268 | 0.000461 |
| 32660 | Zfp518b | -0.79127 | 4.98E-05 |
| 21753 | Nasp | -0.79114 | 5.95E-05 |
| 28577 | Sox12 | -0.78888 | 0.000289 |
| 18221 | Khdrbs3 | -0.78863 | 0.004698 |
| 6786 | Ep300 | -0.78656 | 0.000147 |
| 5128 | Crebbp | -0.78081 | 0.0001 |
| 24166 | Pbx3 | -0.78002 | 0.000104 |
| 24623 | Pik3r1 | -0.77758 | 9.41E-05 |
| 18427 | Kmt2e | -0.77267 | 7.28E-05 |
| 7531 | Flrt2 | -0.77214 | 0.000187 |
| 24292 | Pcyt1b | -0.77156 | 0.003054 |
| 19120 | Lrrtm1 | -0.76994 | 0.002458 |
| 4319 | Celf2 | -0.76676 | 0.000544 |
| 19005 | Lrch3 | -0.76638 | 0.001961 |
| 18982 | Lpar4 | -0.76558 | 0.002416 |
| 26967 | Rspo3 | -0.76359 | 0.001289 |
| 25039 | Pou2f1 | -0.76165 | 0.001649 |
| 18772 | Lhx8 | -0.75903 | 0.00047 |
| 18231 | Kif14 | -0.75875 | 0.000337 |
| 25262 | Prdm16 | -0.75855 | 0.001627 |
| 28708 | Spen | -0.75707 | 0.000363 |
| 24040 | Pag1 | -0.75591 | 0.000198 |
| 27538 | Sfmbt2 | -0.75562 | 0.00287 |
| 32107 | Wdfy3 | -0.75182 | 0.000335 |
| 13249 | Gm2792 | -0.75038 | 0.001021 |
| 24814 | Plekhh2 | -0.74874 | 0.000629 |
| 30814 | Trpc4 | -0.7455 | 0.002522 |
| 27388 | Sema6a | -0.74112 | 0.000107 |
| 30862 | Tshz1 | -0.74106 | 0.000155 |
| 22059 | Nkain1 | -0.73392 | 0.005422 |
| 27714 | Six1 | -0.72999 | 0.00033 |
| 19357 | Map1b | -0.72794 | 0.001128 |
| 30330 | Tox | -0.72597 | 0.005479 |
| 3784 | Cbx2 | -0.72407 | 0.000876 |
| 18203 | Kdm5d | -0.71736 | 0.002763 |
| 31031 | Tut4 | -0.71645 | 0.000141 |
| 1603 | Abtb2 | -0.71549 | 0.004412 |
| 19147 | Lss | -0.7132 | 0.003708 |
| 28551 | Soga1 | -0.71015 | 0.002183 |
| 4515 | Chd7 | -0.7101 | 0.002212 |
| 18250 | Kif24 | -0.70665 | 0.005115 |
| 17325 | Igf2bp2 | -0.70649 | 0.000235 |
| 27107 | Sap130 | -0.70631 | 0.001419 |
| 4334 | Cenpe | -0.70572 | 0.002955 |
| 30762 | Trio | -0.7057 | 0.000713 |
| 18979 | Lpar1 | -0.70104 | 0.000403 |
| 6795 | Epb41l4b | -0.70075 | 0.007094 |
| 2716 | Atad2b | -0.69825 | 0.000938 |
| 22469 | Nynrin | -0.69777 | 0.000574 |
| 24288 | Pcx | -0.69755 | 0.005815 |
| 3329 | Brd4 | -0.69563 | 0.002014 |
| 25002 | Pom121 | -0.69174 | 0.004073 |
| 6727 | Eml4 | -0.6916 | 0.000543 |
| 7651 | Frem1 | -0.69004 | 0.002238 |
| 22372 | Nuak1 | -0.68905 | 0.002215 |
| 24800 | Plekha8 | -0.68829 | 0.000682 |
| 21820 | Ncoa6 | -0.68666 | 0.000334 |
| 24412 | Penk | -0.68517 | 0.005949 |
| 25998 | Raver2 | -0.68027 | 0.000598 |
| 21112 | Mov10 | -0.67974 | 0.003169 |
| 3317 | Bptf | -0.67329 | 0.000506 |
| 28008 | Slc38a4 | -0.67271 | 0.004096 |
| 17177 | Huwe1 | -0.67078 | 0.000777 |
| 29163 | Suv39h2 | -0.6698 | 0.002708 |
| 32338 | Ypel2 | -0.66719 | 0.00172 |
| 21991 | Nfib | -0.66611 | 0.001757 |
| 18040 | Kat6a | -0.66593 | 0.000318 |
| 29788 | Ticrr | -0.66483 | 0.002293 |
| 28211 | Smarcc1 | -0.66477 | 0.000297 |
| 24185 | Pcdh19 | -0.6625 | 0.003108 |
| 27386 | Sema5a | -0.6618 | 0.001556 |
| 22572 | Ogfrl1 | -0.66045 | 0.001675 |
| 5989 | Dicer1 | -0.65874 | 0.002938 |
| 26165 | Rest | -0.65852 | 0.001051 |
| 6792 | Epb41l3 | -0.65794 | 0.000521 |
| 17873 | Irs1 | -0.65672 | 0.002036 |
| 21486 | Mvd | -0.65655 | 0.003348 |
| 2604 | Arnt2 | -0.65591 | 0.001057 |
| 28314 | Smyd3 | -0.65329 | 0.003358 |
| 24473 | Pgf | -0.65279 | 0.002463 |
| 32434 | Zc3hav1l | -0.65233 | 0.001716 |
| 26438 | Rnf150 | -0.65195 | 0.002419 |
| 6851 | Erc1 | -0.65077 | 0.00061 |
| 21710 | Naca | -0.64924 | 0.005918 |
| 3263 | Bmp6 | -0.64808 | 0.004109 |
| 21684 | N4bp2 | -0.64795 | 0.006953 |
| 28895 | Ssbp3 | -0.64556 | 0.002937 |
| 3504 | C2cd3 | -0.64165 | 0.001743 |
| 21119 | Mpdz | -0.64126 | 0.001552 |
| 27723 | Ska2l-ps | -0.64021 | 0.003451 |
| 2542 | Arid1a | -0.64018 | 0.00343 |
| 32529 | Zfp184 | -0.63862 | 0.004634 |
| 24772 | Plce1 | -0.63856 | 0.005595 |
| 6082 | Dna2 | -0.63779 | 0.00467 |
| 30254 | Tnik | -0.63708 | 0.003633 |
| 27416 | Septin6 | -0.63624 | 0.001514 |
| 7268 | Faxc | -0.63567 | 0.001229 |
| 31376 | Usp6nl | -0.63562 | 0.001682 |
| 4684 | Cldn12 | -0.63508 | 0.002488 |
| 29959 | Tmem131l | -0.63198 | 0.004376 |
| 2068 | Akt3 | -0.63113 | 0.004724 |
| 27511 | Setd1b | -0.6306 | 0.004986 |
| 27608 | Sh3d19 | -0.63035 | 0.001592 |
| 6343 | Dusp7 | -0.63024 | 0.002863 |
| 29337 | Tanc2 | -0.62981 | 0.003318 |
| 3012 | Bach1 | -0.62882 | 0.002947 |
| 4373 | Cep350 | -0.62765 | 0.004379 |
| 25600 | Psrc1 | -0.62692 | 0.003059 |
| 17174 | Hunk | -0.62567 | 0.006705 |
| 3171 | Bcr | -0.62125 | 0.007177 |
| 19551 | Mdc1 | -0.62062 | 0.001145 |
| 6426 | E2f7 | -0.61946 | 0.006738 |
| 7867 | Garem1 | -0.61894 | 0.00464 |
| 6276 | Drosha | -0.61738 | 0.004355 |
| 24499 | Phactr4 | -0.61625 | 0.004868 |
| 30284 | Tnrc6c | -0.61522 | 0.003372 |
| 4658 | Clasp1 | -0.61268 | 0.002005 |
| 9043 | Gm14230 | -0.60956 | 0.003405 |
| 5645 | Dach1 | -0.6076 | 0.005462 |
| 7243 | Fancm | -0.60744 | 0.006416 |
| 7902 | Gatad2b | -0.60655 | 0.003375 |
| 19576 | Med1 | -0.60433 | 0.003841 |
| 27667 | Shroom4 | -0.60413 | 0.006878 |
| 22029 | Nhsl1 | -0.60319 | 0.004316 |
| 2250 | Ankrd26 | -0.60227 | 0.007183 |
| 28006 | Slc38a2 | -0.60207 | 0.004507 |
| 6787 | Ep400 | -0.59988 | 0.003422 |
| 27692 | Simc1 | -0.59878 | 0.005683 |
| 4855 | Cnot1 | -0.59844 | 0.001465 |
| 32082 | Wac | -0.59837 | 0.004186 |
| 29418 | Tbc1d16 | -0.59828 | 0.00673 |
| 3788 | Cbx5 | -0.59787 | 0.006484 |
| 30362 | Tpr | -0.59537 | 0.003852 |
| 16773 | Hcfc1 | -0.59429 | 0.005133 |
| 6030 | Dlg5 | -0.59357 | 0.001778 |
| 3827 | Ccdc136 | -0.59275 | 0.004989 |
| 32394 | Zbtb39 | -0.59109 | 0.004081 |
| 18425 | Kmt2c | -0.59087 | 0.005632 |
| 32706 | Zfp618 | -0.59073 | 0.005802 |
| 21288 | Mrtfb | -0.5847 | 0.007085 |
| 6789 | Epb41 | -0.58409 | 0.001593 |
| 25914 | Ralgapa2 | -0.58181 | 0.004386 |
| 24165 | Pbx2 | -0.57911 | 0.003643 |
| 31196 | Uck2 | -0.57571 | 0.003118 |
| 22413 | Numa1 | -0.57571 | 0.002628 |
| 7658 | Frmd4a | -0.5755 | 0.005856 |
| 26261 | Rhobtb1 | -0.57443 | 0.005069 |
| 6814 | Ephb2 | -0.5727 | 0.002816 |
| 3327 | Brd3 | -0.572 | 0.003291 |
| 28003 | Slc38a1 | -0.57196 | 0.003295 |
| 7648 | Fras1 | -0.56958 | 0.00235 |
| 32806 | Zfp827 | -0.56906 | 0.006588 |
| 5785 | Ddx3y | -0.56738 | 0.005338 |
| 19368 | Map2k6 | -0.56259 | 0.006217 |
| 25389 | Prom1 | -0.55626 | 0.006394 |
| 5545 | Cyp51 | -0.55602 | 0.005759 |
| 24970 | Polr1a | -0.55516 | 0.005336 |
| 6381 | Dyrk1a | -0.55429 | 0.006189 |
| 7620 | Foxn3 | -0.55286 | 0.004823 |
| 29571 | Tead2 | -0.55116 | 0.006437 |
| 2502 | Arhgap35 | -0.5466 | 0.004592 |
| 3442 | Bub1 | -0.54135 | 0.005483 |
| 2245 | Ankrd17 | -0.54104 | 0.005745 |
| 27512 | Setd2 | -0.54052 | 0.006153 |
| 16439 | Gsk3b | -0.52089 | 0.004757 |
| 18041 | Kat6b | -0.51592 | 0.006842 |
| 30177 | Tmpo | -0.51108 | 0.005575 |

**Table2. Upregulated DEGs of whole tooth germs at bell stage than at cap stage**

|  | SYMBOL | log2FoldChange | pvalue |
| --- | --- | --- | --- |
| 19180 | Ly6d | 6.922656 | 0 |
| 18460 | Krt13 | 6.829643 | 0 |
| 21040 | Mmp13 | 6.426953 | 1.66E-47 |
| 8088 | Gldn | 6.405759 | 3.21E-35 |
| 30190 | Tmprss3 | 6.064065 | 2.23E-10 |
| 4801 | Cma1 | 6.032327 | 3.20E-20 |
| 29038 | Stmn3 | 5.857335 | 0 |
| 2827 | Atp6v0d2 | 5.816047 | 4.51E-38 |
| 27186 | Scg2 | 5.725605 | 2.01E-58 |
| 27353 | Sele | 5.713985 | 6.88E-14 |
| 7445 | Fgf3 | 5.593054 | 1.10E-52 |
| 1956 | Agt | 5.534838 | 8.82E-14 |
| 26150 | Rem1 | 5.506057 | 1.78E-26 |
| 22620 | Olig1 | 5.470962 | 0 |
| 3950 | Cckar | 5.439473 | 5.01E-24 |
| 17141 | Hspb7 | 5.269087 | 4.51E-12 |
| 19155 | Ltbp2 | 5.244448 | 6.13E-45 |
| 31462 | Vip | 5.242415 | 2.26E-07 |
| 32066 | Vtn | 5.194152 | 0 |
| 28085 | Slc6a11 | 5.179546 | 0 |
| 6046 | Dlx4 | 5.126296 | 6.54E-39 |
| 29746 | Thbs4 | 5.124002 | 8.05E-68 |
| 3122 | Bcan | 5.109128 | 0 |
| 27080 | Sall3 | 5.092052 | 8.01E-31 |
| 19233 | Lyve1 | 5.058072 | 8.74E-49 |
| 17337 | Igfbpl1 | 5.023228 | 0.000333 |
| 6508 | Eef1a2 | 5.011695 | 0 |
| 6047 | Dlx4os | 5.005784 | 1.63E-24 |
| 17708 | Il6 | 4.999519 | 7.72E-11 |
| 16261 | Gpr149 | 4.995669 | 7.06E-10 |
| 16369 | Gria2 | 4.966732 | 1.12E-47 |
| 32079 | Vwde | 4.944203 | 3.21E-19 |
| 26552 | Rph3a | 4.912314 | 0 |
| 29731 | Th | 4.869857 | 1.62E-08 |
| 22546 | Ocstamp | 4.833579 | 1.47E-10 |
| 21182 | Mrgprf | 4.832694 | 5.95E-20 |
| 24741 | Plac9 | 4.82256 | 5.37E-24 |
| 24361 | Pdlim3 | 4.819418 | 7.47E-36 |
| 17736 | Ina | 4.775081 | 0 |
| 27049 | S100a3 | 4.725964 | 3.26E-07 |
| 27057 | S100b | 4.722878 | 2.82E-41 |
| 24380 | Pdyn | 4.720303 | 0 |
| 3484 | C1qtnf3 | 4.700908 | 8.33E-77 |
| 30369 | Tpsb2 | 4.682269 | 4.31E-11 |
| 7920 | Gbp6 | 4.639493 | 1.16E-05 |
| 28084 | Slc6a1 | 4.628828 | 0 |
| 26968 | Rspo4 | 4.623219 | 5.53E-37 |
| 18484 | Krt4 | 4.582486 | 0.000139 |
| 5490 | Cyp2f2 | 4.568962 | 8.28E-06 |
| 6061 | Dmkn | 4.563155 | 0.000114 |
| 17155 | Htr1a | 4.554844 | 6.94E-08 |
| 5687 | Dbx2 | 4.543232 | 1.20E-26 |
| 5729 | Dcstamp | 4.526029 | 2.12E-09 |
| 22368 | Ntrk3 | 4.507262 | 8.00E-53 |
| 29957 | Tmem130 | 4.485254 | 0 |
| 18135 | Kcnk9 | 4.458184 | 0.001539 |
| 3605 | Calca | 4.457211 | 1.22E-57 |
| 3615 | Calhm4 | 4.44046 | 1.41E-08 |
| 6481 | Ecrg4 | 4.437026 | 6.92E-11 |
| 23943 | Otof | 4.436885 | 1.95E-07 |
| 27244 | Scml4 | 4.433387 | 7.74E-17 |
| 19207 | Lypd1 | 4.422253 | 2.95E-36 |
| 7595 | Foxd2 | 4.401256 | 6.98E-16 |
| 21351 | Mt3 | 4.394467 | 3.55E-05 |
| 4202 | Cdh17 | 4.376847 | 3.55E-30 |
| 3977 | Ccl9 | 4.334035 | 3.81E-33 |
| 25476 | Prss30 | 4.324685 | 9.65E-05 |
| 19151 | Ltb | 4.3217 | 2.02E-09 |
| 25975 | Rasgrf2 | 4.308117 | 7.27E-20 |
| 22625 | Omd | 4.276251 | 3.55E-69 |
| 6664 | Elavl3 | 4.272028 | 0 |
| 22482 | Oas2 | 4.259532 | 2.86E-12 |
| 4738 | Clec4a2 | 4.251131 | 1.42E-17 |
| 1673 | Acp5 | 4.242503 | 2.46E-51 |
| 29680 | Tfap2d | 4.242125 | 0 |
| 19824 | Mir124a-1hg | 4.232464 | 0.002385 |
| 4945 | Col5a3 | 4.215915 | 2.79E-41 |
| 3961 | Ccl21a | 4.213998 | 2.53E-14 |
| 21604 | Myt1l | 4.197967 | 0 |
| 27275 | Scrt1 | 4.195048 | 0 |
| 26997 | Rtn4rl1 | 4.185357 | 9.14E-20 |
| 7479 | Fhl5 | 4.180781 | 0.000296 |
| 32227 | Wnt7a | 4.180203 | 3.21E-06 |
| 3975 | Ccl7 | 4.15049 | 6.66E-09 |
| 17757 | Inmt | 4.147278 | 3.38E-07 |
| 19820 | Mir124-2hg | 4.139887 | 0.003097 |
| 17202 | Ibsp | 4.13347 | 3.14E-06 |
| 22574 | Ogn | 4.127867 | 1.42E-74 |
| 27056 | S100a9 | 4.124253 | 2.43E-15 |
| 1711 | Actl6b | 4.117419 | 0 |
| 7022 | F13a1 | 4.11539 | 5.07E-69 |
| 7058 | Fabp7 | 4.109329 | 0 |
| 23932 | Osm | 4.08532 | 3.77E-11 |
| 18120 | Kcnj8 | 4.073241 | 1.36E-06 |
| 27809 | Slc17a6 | 4.068579 | 0 |
| 2279 | Ankrd63 | 4.064482 | 1.54E-05 |
| 25464 | Prrxl1 | 4.054747 | 8.43E-07 |
| 4471 | Cfh | 4.047017 | 7.27E-35 |
| 18765 | Lhx1 | 4.040951 | 0.004069 |
| 3884 | Ccdc3 | 4.0291 | 4.93E-72 |
| 22551 | Odam | 3.99961 | 0.000106 |
| 3436 | Btnl2 | 3.995287 | 0.000536 |
| 18488 | Krt6a | 3.98328 | 0 |
| 29641 | Tex14 | 3.980819 | 6.25E-12 |
| 2140 | Alpk2 | 3.977272 | 2.92E-10 |
| 18761 | Lhfpl3 | 3.971981 | 2.05E-09 |
| 22326 | Nsg2 | 3.944325 | 0 |
| 16799 | Hdc | 3.941051 | 1.05E-21 |
| 32593 | Zfp36 | 3.93676 | 3.98E-45 |
| 14505 | Gm5084 | 3.926513 | 8.56E-10 |
| 7915 | Gbp2 | 3.925197 | 1.90E-10 |
| 7327 | Fbxo40 | 3.922986 | 2.54E-05 |
| 18852 | Lmo1 | 3.90892 | 3.55E-29 |
| 25160 | Ppp2r2c | 3.89458 | 2.91E-05 |
| 7947 | Gcnt4 | 3.885661 | 2.89E-23 |
| 5350 | Cubn | 3.873222 | 4.39E-18 |
| 19538 | Mcpt4 | 3.869407 | 0.001052 |
| 30368 | Tpsab1 | 3.869126 | 1.66E-06 |
| 29246 | Syt4 | 3.864724 | 7.12E-05 |
| 27055 | S100a8 | 3.841706 | 4.61E-10 |
| 26203 | Rfx4 | 3.837635 | 0 |
| 28333 | Sncb | 3.831884 | 0.001563 |
| 21006 | Mlc1 | 3.797546 | 0 |
| 7636 | Foxs1 | 3.791565 | 9.12E-12 |
| 8364 | Gm11413 | 3.788574 | 0.000453 |
| 17663 | Il1b | 3.782915 | 9.03E-11 |
| 32215 | Wnt10a | 3.781844 | 9.90E-17 |
| 27677 | Siglec1 | 3.778206 | 8.34E-09 |
| 18149 | Kcnq2 | 3.777988 | 0 |
| 13917 | Gm3764 | 3.765749 | 0 |
| 2162 | Amer3 | 3.764538 | 0.00682 |
| 21551 | Mylk2 | 3.760473 | 1.15E-05 |
| 29039 | Stmn4 | 3.751514 | 0 |
| 2831 | Atp6v1b1 | 3.737683 | 2.74E-06 |
| 19544 | Mctp1 | 3.734854 | 4.05E-11 |
| 18146 | Kcnn4 | 3.724378 | 1.94E-11 |
| 30821 | Trpm1 | 3.714515 | 3.85E-16 |
| 27041 | Ryr3 | 3.714264 | 9.03E-43 |
| 4825 | Cmya5 | 3.710495 | 9.97E-29 |
| 25283 | Prelp | 3.706398 | 1.22E-27 |
| 17251 | Ifi27l2a | 3.696736 | 7.77E-13 |
| 29281 | Tac1 | 3.685618 | 1.55E-20 |
| 2406 | Apod | 3.678419 | 1.42E-52 |
| 26170 | Retnlg | 3.673089 | 0.00237 |
| 18051 | Kazald1 | 3.658039 | 1.57E-25 |
| 2349 | Ap3b2 | 3.657526 | 0 |
| 21863 | Ndufa4l2 | 3.654273 | 8.32E-06 |
| 24858 | Plppr5 | 3.650088 | 3.46E-21 |
| 18470 | Krt23 | 3.621702 | 1.94E-07 |
| 22192 | Notum | 3.615783 | 1.14E-05 |
| 27187 | Scg3 | 3.603998 | 0 |
| 22242 | Nptx2 | 3.603041 | 6.60E-18 |
| 16111 | Gmnc | 3.59447 | 0.003075 |
| 17239 | Ifi202b | 3.588147 | 1.95E-22 |
| 19235 | Lyz2 | 3.582286 | 1.79E-29 |
| 29870 | Tlr8 | 3.571389 | 4.43E-05 |
| 4417 | Ces2g | 3.562885 | 4.94E-05 |
| 7802 | Gabrg3 | 3.559701 | 1.13E-11 |
| 28953 | St8sia3 | 3.558466 | 0.006573 |
| 2780 | Atp1a3 | 3.553087 | 0 |
| 25363 | Prlr | 3.533255 | 0.000256 |
| 20409 | Mir599 | 3.529113 | 0.000541 |
| 27370 | Selp | 3.525838 | 0.005047 |
| 19539 | Mcpt8 | 3.525144 | 0.004491 |
| 22468 | Nyap2 | 3.514134 | 0.004094 |
| 2333 | Aox4 | 3.512586 | 6.21E-09 |
| 27640 | Shh | 3.507958 | 0.000594 |
| 6771 | Entpd3 | 3.502796 | 1.78E-35 |
| 27031 | Rxfp3 | 3.500005 | 4.81E-25 |
| 21603 | Myt1 | 3.499142 | 0.001083 |
| 16916 | Hlf | 3.489114 | 1.82E-27 |
| 6571 | Egr3 | 3.487395 | 5.51E-05 |
| 27462 | Serpinb12 | 3.484887 | 0.001469 |
| 3426 | Btg2 | 3.476752 | 6.25E-35 |
| 17226 | Idi2 | 3.466663 | 3.99E-06 |
| 2726 | Atf3 | 3.449598 | 3.29E-25 |
| 27523 | Sez6 | 3.441355 | 0 |
| 4740 | Clec4a4 | 3.42611 | 0.005863 |
| 28999 | Steap4 | 3.423831 | 3.75E-18 |
| 3480 | C1ql4 | 3.416874 | 9.21E-05 |
| 5054 | Cpa6 | 3.413357 | 3.65E-16 |
| 28769 | Spp1 | 3.409972 | 4.53E-18 |
| 21853 | Ndst4 | 3.406019 | 0.000218 |
| 29750 | Them5 | 3.399121 | 1.39E-05 |
| 25479 | Prss34 | 3.393516 | 0.001997 |
| 22480 | Oas1g | 3.385438 | 0.00025 |
| 5048 | Cp | 3.382565 | 1.24E-11 |
| 21302 | Ms4a4a | 3.379921 | 3.83E-08 |
| 18607 | Lamc3 | 3.379095 | 0.001843 |
| 16175 | Golga7b | 3.37547 | 1.60E-08 |
| 25626 | Ptgds | 3.37298 | 0.006222 |
| 22563 | Ofcc1 | 3.367665 | 0.000217 |
| 22350 | Nt5e | 3.367632 | 8.82E-55 |
| 4206 | Cdh20 | 3.359734 | 1.47E-10 |
| 18773 | Lhx9 | 3.357717 | 0 |
| 7836 | Galnt15 | 3.355545 | 0.000371 |
| 17169 | Htra1 | 3.34742 | 4.90E-26 |
| 5183 | Crybb1 | 3.347115 | 0.001141 |
| 25836 | Rab3c | 3.343135 | 0 |
| 27256 | Scn7a | 3.33794 | 1.35E-08 |
| 17240 | Ifi203 | 3.326478 | 5.04E-20 |
| 19777 | Mill2 | 3.324845 | 5.15E-06 |
| 25976 | Rasgrp1 | 3.319035 | 1.10E-13 |
| 27252 | Scn3b | 3.314151 | 5.80E-08 |
| 7215 | Fam83a | 3.312615 | 0.000702 |
| 27761 | Slc10a4 | 3.312592 | 1.74E-05 |
| 30264 | Tnmd | 3.311356 | 3.63E-32 |
| 28149 | Slco4a1 | 3.308417 | 1.98E-09 |
| 19178 | Ly6c1 | 3.294177 | 5.23E-09 |
| 17039 | Hoxd8 | 3.291354 | 1.77E-05 |
| 24857 | Plppr4 | 3.285569 | 3.15E-19 |
| 18740 | Lgals3 | 3.282384 | 9.47E-14 |
| 4415 | Ces2e | 3.279349 | 1.57E-05 |
| 3606 | Calcb | 3.275739 | 1.77E-15 |
| 27703 | Sirpb1c | 3.273757 | 0.001418 |
| 4950 | Col6a5 | 3.272066 | 4.35E-07 |
| 28502 | Sntg2 | 3.271023 | 1.05E-08 |
| 6062 | Dmp1 | 3.270046 | 2.41E-08 |
| 14821 | Gm5862 | 3.267819 | 0.007186 |
| 7446 | Fgf4 | 3.262596 | 0.000467 |
| 17871 | Irgm2 | 3.261375 | 2.95E-14 |
| 4739 | Clec4a3 | 3.261345 | 3.36E-07 |
| 24854 | Plppr1 | 3.256889 | 1.44E-41 |
| 6765 | Enpp6 | 3.253555 | 0.000305 |
| 23993 | P2rx7 | 3.252531 | 6.76E-19 |
| 17671 | Il1rl1 | 3.250453 | 2.80E-13 |
| 7055 | Fabp4 | 3.249958 | 0.000349 |
| 5376 | Cxcl1 | 3.241072 | 1.66E-29 |
| 6015 | Dkk1 | 3.237853 | 2.33E-25 |
| 24681 | Piwil4 | 3.237081 | 0.003761 |
| 7596 | Foxd2os | 3.229741 | 1.34E-12 |
| 29487 | Tcea3 | 3.227591 | 1.16E-14 |
| 28000 | Slc37a2 | 3.221092 | 2.60E-27 |
| 18108 | Kcnj10 | 3.216222 | 0 |
| 27392 | Sema7a | 3.213949 | 1.74E-20 |
| 31068 | Tyrobp | 3.213398 | 1.52E-30 |
| 31467 | Vit | 3.206786 | 1.22E-25 |
| 5266 | Cstdc5 | 3.203753 | 2.07E-05 |
| 27046 | S100a14 | 3.200243 | 9.34E-05 |
| 7482 | Fibcd1 | 3.195808 | 2.91E-10 |
| 28739 | Spink5 | 3.186668 | 9.26E-05 |
| 18570 | Krtdap | 3.168981 | 0 |
| 32081 | Vxn | 3.168779 | 0.002662 |
| 32200 | Wif1 | 3.16813 | 4.68E-21 |
| 18975 | Loxl1 | 3.161266 | 1.03E-34 |
| 18066 | Kcna2 | 3.157251 | 2.50E-12 |
| 16405 | Grm5 | 3.149512 | 0.001794 |
| 5377 | Cxcl10 | 3.146197 | 2.09E-07 |
| 16436 | Gsg1l | 3.142197 | 7.69E-17 |
| 24083 | Pappa2 | 3.131166 | 5.14E-07 |
| 19428 | Mapt | 3.127988 | 0 |
| 1645 | Ackr4 | 3.127634 | 2.17E-14 |
| 6232 | Dpp10 | 3.122199 | 5.80E-05 |
| 4719 | Clec11a | 3.118331 | 3.15E-26 |
| 24613 | Pik3ap1 | 3.116015 | 4.92E-25 |
| 5802 | Ddx60 | 3.113425 | 4.94E-06 |
| 5055 | Cpb1 | 3.111566 | 2.28E-05 |
| 7970 | Gdpd2 | 3.10404 | 1.95E-13 |
| 4010 | Ccno | 3.100878 | 1.32E-05 |
| 26363 | Rit2 | 3.098308 | 0.004844 |
| 21308 | Ms4a6c | 3.094664 | 5.00E-16 |
| 7380 | Fcna | 3.094249 | 0.000251 |
| 25514 | Psapl1 | 3.09156 | 0.006506 |
| 18729 | Lepr | 3.090373 | 2.06E-33 |
| 23933 | Osmr | 3.089422 | 2.91E-10 |
| 17241 | Ifi204 | 3.086332 | 1.44E-07 |
| 16876 | Hhip | 3.076581 | 2.01E-31 |
| 21052 | Mmp24 | 3.072803 | 0.003761 |
| 4747 | Clec4n | 3.068622 | 1.14E-05 |
| 2328 | Aoc3 | 3.064838 | 1.91E-18 |
| 24278 | Pcsk1n | 3.054345 | 0.006357 |
| 16758 | Hbb-b1 | 3.053794 | 3.59E-21 |
| 4664 | Clca3a1 | 3.047315 | 5.80E-09 |
| 19468 | Matn4 | 3.045834 | 0 |
| 3146 | Bcl2a1b | 3.040745 | 0.003628 |
| 25856 | Rab7b | 3.038837 | 1.92E-10 |
| 18489 | Krt6b | 3.037552 | 0 |
| 2698 | Aspn | 3.036146 | 7.03E-42 |
| 27780 | Slc13a3 | 3.035748 | 0.000213 |
| 13710 | Gm34354 | 3.034487 | 5.46E-10 |
| 18462 | Krt15 | 3.034315 | 0.000901 |
| 27052 | S100a6 | 3.029089 | 9.34E-47 |
| 16762 | Hbb-bt | 3.027242 | 3.07E-20 |
| 3491 | C1rb | 3.024879 | 1.87E-09 |
| 4919 | Col14a1 | 3.023113 | 2.51E-27 |
| 27946 | Slc2a7 | 3.020812 | 0.004447 |
| 3036 | Barhl1 | 3.020109 | 0 |
| 3974 | Ccl6 | 3.019412 | 0.000718 |
| 3959 | Ccl2 | 3.018914 | 7.86E-12 |
| 5051 | Cpa3 | 3.01358 | 3.45E-18 |
| 21309 | Ms4a6d | 3.001502 | 1.78E-13 |
| 21601 | Myrip | 3.000409 | 1.26E-10 |
| 16235 | Gpihbp1 | 2.998885 | 9.11E-14 |
| 25875 | Rac2 | 2.995643 | 2.50E-28 |
| 30750 | Trim67 | 2.989043 | 1.01E-06 |
| 6045 | Dlx3 | 2.983976 | 1.68E-18 |
| 29842 | Tlcd3b | 2.981293 | 9.88E-05 |
| 29176 | Svop | 2.979528 | 0 |
| 5182 | Cryba4 | 2.979353 | 0.004759 |
| 21310 | Ms4a7 | 2.978895 | 4.77E-06 |
| 3953 | Ccl11 | 2.973979 | 0.000517 |
| 6168 | Dner | 2.972614 | 0.002527 |
| 4089 | Cd300c2 | 2.970685 | 2.54E-06 |
| 21307 | Ms4a6b | 2.970247 | 1.44E-13 |
| 25922 | Ramp1 | 2.962861 | 1.90E-08 |
| 21906 | Neat1 | 2.95262 | 1.34E-14 |
| 29820 | Timp4 | 2.951368 | 0.002316 |
| 21788 | Ncan | 2.946348 | 0 |
| 24732 | Pla2g7 | 2.94326 | 4.33E-20 |
| 24736 | Plaat3 | 2.94109 | 5.62E-17 |
| 27262 | Scnn1g | 2.939761 | 1.74E-05 |
| 3188 | Best1 | 2.939407 | 2.03E-06 |
| 30477 | Trarg1 | 2.937919 | 0.002686 |
| 17673 | Il1rn | 2.933362 | 0.002044 |
| 5719 | Dcn | 2.926883 | 1.42E-31 |
| 30658 | Trem2 | 2.924398 | 6.03E-08 |
| 26078 | Rbpjl | 2.924251 | 8.69E-07 |
| 27448 | Serpina3h | 2.924095 | 7.76E-09 |
| 3716 | Cartpt | 2.923638 | 0.032501 |
| 4142 | Cd86 | 2.922386 | 3.13E-07 |
| 4720 | Clec12a | 2.918771 | 3.29E-12 |
| 6018 | Dkk4 | 2.916735 | 7.90E-05 |
| 24656 | Pira1 | 2.916039 | 0.001568 |
| 17849 | Irag1 | 2.909592 | 2.79E-10 |
| 6217 | Dpep1 | 2.907879 | 0.000389 |
| 4199 | Cdh13 | 2.906621 | 7.07E-31 |
| 29345 | Tap2 | 2.902516 | 4.67E-12 |
| 3490 | C1ra | 2.89659 | 1.90E-19 |
| 18089 | Kcne4 | 2.894783 | 1.43E-21 |
| 6281 | Dscam | 2.892055 | 0.001744 |
| 1526 | Abca8a | 2.887247 | 4.05E-11 |
| 13231 | Gm27239 | 2.8838 | 0.03429 |
| 25693 | Ptprn | 2.882471 | 7.58E-10 |
| 1992 | AI662270 | 2.87528 | 1.49E-05 |
| 22018 | Ngp | 2.873672 | 0.000714 |
| 7122 | Fam167a | 2.869991 | 3.18E-05 |
| 6315 | Duoxa1 | 2.868599 | 1.24E-06 |
| 25563 | Psmb9 | 2.866981 | 1.37E-08 |
| 4523 | Chga | 2.864369 | 0.001723 |
| 23992 | P2rx6 | 2.863191 | 2.75E-07 |
| 4737 | Clec4a1 | 2.863146 | 9.86E-09 |
| 14669 | Gm5431 | 2.862381 | 0.004487 |
| 2080 | Aldh1a3 | 2.861746 | 3.40E-18 |
| 28181 | Slpi | 2.858255 | 1.08E-07 |
| 27454 | Serpina3n | 2.855391 | 0.002678 |
| 1528 | Abca9 | 2.84772 | 4.37E-15 |
| 6327 | Dusp15 | 2.847465 | 2.00E-07 |
| 28295 | Smpd3 | 2.846238 | 3.91E-16 |
| 24658 | Pira2 | 2.83575 | 0.000794 |
| 3954 | Ccl12 | 2.833795 | 2.80E-05 |
| 17162 | Htr3a | 2.827838 | 2.75E-06 |
| 32054 | Vsnl1 | 2.82774 | 9.90E-15 |
| 19168 | Lum | 2.822725 | 3.90E-29 |
| 24358 | Pdk4 | 2.81816 | 1.36E-21 |
| 6469 | Ecel1 | 2.81707 | 4.55E-06 |
| 3723 | Casp1 | 2.815502 | 3.84E-06 |
| 6286 | Dsg1a | 2.814926 | 0.002366 |
| 16238 | Gpm6a | 2.814191 | 5.76E-13 |
| 3623 | Calml3 | 2.799249 | 0.000124 |
| 17620 | Iigp1 | 2.790521 | 1.53E-15 |
| 17914 | Itga7 | 2.787965 | 3.70E-09 |
| 17750 | Inhbb | 2.784704 | 1.12E-05 |
| 5208 | Csf1r | 2.782199 | 6.89E-31 |
| 4023 | Ccr3 | 2.773045 | 0.045923 |
| 25695 | Ptpro | 2.772413 | 2.45E-06 |
| 17679 | Il21r | 2.769892 | 1.95E-06 |
| 4889 | Cntnap4 | 2.769478 | 1.86E-17 |
| 17041 | Hp | 2.767065 | 3.91E-05 |
| 7817 | Gal | 2.765864 | 0.00173 |
| 21845 | Ndp | 2.763686 | 1.02E-16 |
| 27053 | S100a7a | 2.760684 | 0.001285 |
| 26704 | Rpl39l | 2.758091 | 0.001296 |
| 5211 | Csf2rb | 2.757363 | 3.61E-18 |
| 17886 | Isg15 | 2.757137 | 6.45E-05 |
| 31223 | Ugt1a6b | 2.756949 | 0.00444 |
| 6538 | Efcc1 | 2.747485 | 3.49E-06 |
| 28162 | Slfn4 | 2.744226 | 0.002414 |
| 17777 | Insc | 2.743474 | 8.19E-22 |
| 19334 | Mamstr | 2.743349 | 5.02E-17 |
| 25694 | Ptprn2 | 2.743124 | 0.00341 |
| 17266 | Ifitm10 | 2.735831 | 2.75E-06 |
| 22439 | Nupr1 | 2.735029 | 9.28E-07 |
| 21317 | Msc | 2.72848 | 5.54E-07 |
| 19053 | Lrrc25 | 2.726942 | 1.90E-08 |
| 27011 | Rufy4 | 2.723112 | 1.67E-06 |
| 6840 | Epsti1 | 2.72196 | 8.31E-05 |
| 16913 | Hk3 | 2.721366 | 1.05E-12 |
| 7245 | Fap | 2.719544 | 7.20E-17 |
| 7463 | Fgl2 | 2.719188 | 1.37E-12 |
| 28635 | Sparcl1 | 2.717933 | 3.69E-32 |
| 28169 | Slit1 | 2.717576 | 0 |
| 5212 | Csf2rb2 | 2.714179 | 2.87E-10 |
| 7366 | Fcer1g | 2.712129 | 8.92E-20 |
| 2013 | Ajap1 | 2.71186 | 3.17E-06 |
| 17870 | Irgm1 | 2.707786 | 4.45E-22 |
| 17250 | Ifi27 | 2.704734 | 9.42E-18 |
| 24803 | Plekhd1 | 2.703613 | 3.40E-07 |
| 2908 | Avpr1a | 2.70272 | 2.19E-06 |
| 18499 | Krt79 | 2.702221 | 0.00029 |
| 3724 | Casp12 | 2.700305 | 0.001266 |
| 4141 | Cd84 | 2.699759 | 3.58E-07 |
| 7726 | Fut2 | 2.690807 | 1.06E-06 |
| 30684 | Trhr | 2.687217 | 0.001473 |
| 22362 | Ntn5 | 2.686389 | 1.12E-08 |
| 29965 | Tmem132e | 2.68578 | 9.86E-14 |
| 5711 | Dchs2 | 2.684738 | 6.05E-19 |
| 16942 | Hmgcs2 | 2.683547 | 5.19E-15 |
| 16279 | Gpr176 | 2.681905 | 1.94E-07 |
| 29878 | Tm4sf1 | 2.674486 | 5.89E-16 |
| 19569 | Me3 | 2.670637 | 2.57E-14 |
| 21804 | Ncf4 | 2.668407 | 2.88E-11 |
| 19324 | Mall | 2.665572 | 1.48E-06 |
| 5569 | Cytip | 2.659406 | 7.03E-05 |
| 17257 | Ifi47 | 2.655574 | 2.53E-08 |
| 16778 | Hck | 2.654046 | 1.23E-13 |
| 14370 | Gm4841 | 2.651451 | 2.28E-09 |
| 17617 | Igtp | 2.64656 | 1.36E-12 |
| 6234 | Dpp4 | 2.645514 | 1.12E-07 |
| 28285 | Smoc2 | 2.644479 | 5.39E-37 |
| 17171 | Htra3 | 2.633405 | 0.000191 |
| 17233 | Ier3 | 2.633189 | 5.05E-16 |
| 2469 | Arfgef3 | 2.633041 | 5.80E-13 |
| 17926 | Itgb2 | 2.632875 | 4.04E-10 |
| 24002 | P2ry6 | 2.632552 | 5.89E-09 |
| 29879 | Tm4sf19 | 2.629542 | 0.001866 |
| 24662 | Pirb | 2.626322 | 1.94E-05 |
| 25604 | Ptafr | 2.625359 | 4.92E-05 |
| 7244 | Fank1 | 2.619128 | 0.003459 |
| 3260 | Bmp3 | 2.619096 | 0.000762 |
| 21069 | Mndal | 2.616116 | 6.81E-09 |
| 18487 | Krt5 | 2.614123 | 0.001483 |
| 28869 | Srrm3 | 2.613344 | 0.006449 |
| 28160 | Slfn2 | 2.613166 | 5.82E-10 |
| 32645 | Zfp469 | 2.612099 | 3.41E-21 |
| 5385 | Cxcl2 | 2.596979 | 8.13E-11 |
| 133 | 1700017G19Rik | 2.595248 | 8.69E-06 |
| 7844 | Galnt6 | 2.587884 | 1.98E-08 |
| 24441 | Pf4 | 2.582544 | 8.08E-14 |
| 32409 | Zbtb7c | 2.582361 | 1.84E-11 |
| 4020 | Ccr10 | 2.578894 | 0.005596 |
| 21155 | Mrc1 | 2.578426 | 6.59E-31 |
| 5330 | Ctsk | 2.57663 | 1.37E-32 |
| 31224 | Ugt1a7c | 2.572867 | 0.000548 |
| 25042 | Pou3f1 | 2.57267 | 0.002032 |
| 25602 | Pstpip1 | 2.572441 | 1.28E-11 |
| 5133 | Creg2 | 2.571352 | 0.001736 |
| 22047 | Nipal1 | 2.570911 | 1.07E-06 |
| 6268 | Drd1 | 2.568198 | 0.004545 |
| 25255 | Prdm1 | 2.567529 | 3.31E-19 |
| 4744 | Clec4e | 2.563342 | 0.002963 |
| 1292 | 9330188P03Rik | 2.563083 | 0.001684 |
| 24468 | Pgap6 | 2.559787 | 2.17E-10 |
| 3476 | C1qc | 2.559692 | 2.13E-18 |
| 25562 | Psmb8 | 2.557977 | 1.01E-06 |
| 27762 | Slc10a4-ps | 2.556733 | 0.002703 |
| 24786 | Pld4 | 2.556467 | 1.40E-13 |
| 18302 | Klf2 | 2.555035 | 5.70E-14 |
| 3473 | C1qa | 2.549687 | 4.20E-21 |
| 30381 | Traf1 | 2.549084 | 1.80E-08 |
| 5337 | Ctss | 2.546499 | 1.07E-21 |
| 5323 | Ctsc | 2.545343 | 4.16E-24 |
| 4019 | Ccr1 | 2.544924 | 6.00E-09 |
| 8014 | Ggt1 | 2.541864 | 0.000382 |
| 27906 | Slc25a48 | 2.540864 | 0.000692 |
| 22232 | Nppc | 2.537536 | 0.001191 |
| 21058 | Mmp8 | 2.537207 | 0.004038 |
| 1839 | Adgre1 | 2.534506 | 7.05E-18 |
| 5078 | Cpne5 | 2.533299 | 6.99E-17 |
| 28598 | Sp100 | 2.533011 | 4.55E-07 |
| 29594 | Tekt5 | 2.531834 | 0.006097 |
| 2497 | Arhgap30 | 2.531773 | 8.45E-16 |
| 24794 | Plekha2 | 2.531747 | 2.95E-15 |
| 21106 | Morrbid | 2.522037 | 4.24E-07 |
| 13435 | Gm30648 | 2.519041 | 0.000416 |
| 4314 | Cela1 | 2.51862 | 0.002388 |
| 28751 | Spn | 2.513241 | 0.001003 |
| 18783 | Lilrb4b | 2.510927 | 7.35E-08 |
| 4683 | Cldn11 | 2.510305 | 3.41E-16 |
| 2322 | Aoah | 2.50931 | 0.000114 |
| 18078 | Kcnc2 | 2.508274 | 0.001468 |
| 24707 | Pkp1 | 2.507041 | 0.0012 |
| 18743 | Lgals7 | 2.499865 | 9.08E-18 |
| 4119 | Cd53 | 2.498874 | 1.11E-10 |
| 19650 | Metrnl | 2.498278 | 6.32E-18 |
| 22474 | Oas1a | 2.496627 | 6.14E-07 |
| 30107 | Tmem37 | 2.494549 | 2.38E-08 |
| 6570 | Egr2 | 2.494386 | 1.61E-15 |
| 7921 | Gbp7 | 2.494241 | 1.03E-08 |
| 29698 | Tfpi2 | 2.489838 | 0.000537 |
| 7388 | Fcrls | 2.486793 | 6.19E-22 |
| 5168 | Crtac1 | 2.482722 | 2.59E-18 |
| 26219 | Rgs1 | 2.48005 | 1.44E-11 |
| 24870 | Plxdc1 | 2.479453 | 1.20E-08 |
| 19148 | Lst1 | 2.477089 | 4.13E-06 |
| 29730 | Tgtp2 | 2.472182 | 0.000158 |
| 1502 | Aard | 2.471706 | 6.28E-14 |
| 6579 | Ehf | 2.470207 | 0.001049 |
| 4116 | Cd48 | 2.468868 | 0.000539 |
| 1285 | 9330159F19Rik | 2.466361 | 0.000271 |
| 2400 | Apobr | 2.465479 | 2.54E-08 |
| 5221 | Csmd3 | 2.463795 | 1.89E-07 |
| 28718 | Spi1 | 2.463634 | 5.38E-12 |
| 28761 | Spon1 | 2.460017 | 2.72E-20 |
| 26225 | Rgs16 | 2.45924 | 2.20E-15 |
| 28609 | Sp9 | 2.45885 | 1.56E-09 |
| 26213 | Rgl3 | 2.456938 | 0.000249 |
| 3923 | Ccdc81 | 2.456482 | 8.86E-07 |
| 22097 | Nlrc5 | 2.455098 | 0.001588 |
| 16839 | Hepacam | 2.452915 | 0.003454 |
| 5067 | Cplx1 | 2.4527 | 0.00454 |
| 30223 | Tnfrsf11a | 2.451692 | 2.61E-14 |
| 7554 | Fmod | 2.450919 | 3.79E-18 |
| 26191 | Rflna | 2.449869 | 2.90E-07 |
| 7263 | Fat2 | 2.447049 | 5.23E-10 |
| 3011 | Bace2 | 2.44251 | 8.58E-09 |
| 18154 | Kcns1 | 2.440585 | 4.93E-08 |
| 31259 | Unc13c | 2.440178 | 1.34E-14 |
| 17657 | Il18 | 2.438713 | 2.14E-08 |
| 22448 | Nwd1 | 2.433128 | 0.00211 |
| 19739 | Mgp | 2.427834 | 2.57E-13 |
| 6863 | Ereg | 2.427254 | 0.00599 |
| 4101 | Cd33 | 2.426613 | 4.90E-08 |
| 32252 | Xaf1 | 2.425913 | 8.28E-08 |
| 16250 | Gpr132 | 2.425225 | 0.001274 |
| 29204 | Syn1 | 2.423868 | 1.51E-09 |
| 19211 | Lypd3 | 2.423737 | 4.07E-05 |
| 30226 | Tnfrsf13b | 2.421653 | 2.32E-06 |
| 29691 | Tfec | 2.420648 | 7.78E-05 |
| 25651 | Ptk2b | 2.419447 | 1.39E-07 |
| 4207 | Cdh22 | 2.418863 | 2.23E-09 |
| 16865 | Hfe | 2.418646 | 1.14E-13 |
| 19123 | Lrrtm4 | 2.418541 | 2.21E-14 |
| 21808 | Nckap1l | 2.416405 | 1.01E-20 |
| 17632 | Il10ra | 2.416032 | 1.02E-06 |
| 1634 | Ace | 2.412442 | 2.21E-06 |
| 2423 | Apold1 | 2.410399 | 1.33E-19 |
| 24657 | Pira11 | 2.410244 | 0.007065 |
| 29605 | Tent5a | 2.407016 | 2.06E-18 |
| 1959 | Agtr1b | 2.405658 | 0.003118 |
| 29934 | Tmem106a | 2.403837 | 7.08E-11 |
| 30045 | Tmem215 | 2.403241 | 0.002522 |
| 22315 | Nrxn2 | 2.403226 | 8.90E-08 |
| 21082 | Mocos | 2.400998 | 5.41E-05 |
| 7953 | Gdap1l1 | 2.399677 | 0.004365 |
| 18017 | Junb | 2.399139 | 6.41E-14 |
| 12349 | Gm25005 | 2.398899 | 0.003059 |
| 5080 | Cpne7 | 2.394711 | 1.49E-13 |
| 28863 | Srpx | 2.393452 | 5.41E-17 |
| 30683 | Trhde | 2.392689 | 0.000795 |
| 25101 | Ppm1j | 2.391954 | 2.70E-11 |
| 21570 | Myo1f | 2.391953 | 6.58E-14 |
| 4322 | Celf5 | 2.391279 | 5.14E-16 |
| 21535 | Myh8 | 2.391101 | 0.000506 |
| 4051 | Cd163 | 2.390918 | 0.000815 |
| 18973 | Lox | 2.38758 | 5.77E-33 |
| 21336 | Msr1 | 2.387153 | 1.47E-15 |
| 28569 | Sostdc1 | 2.385132 | 2.58E-21 |
| 24739 | Plac8 | 2.384567 | 2.54E-06 |
| 27183 | Scel | 2.382413 | 1.23E-05 |
| 28665 | Spata6l | 2.380947 | 0.002796 |
| 17247 | Ifi211 | 2.376481 | 0.000295 |
| 3577 | Cacna2d2 | 2.373436 | 3.92E-14 |
| 22584 | Olfm4 | 2.373057 | 0.000575 |
| 18693 | Lcp2 | 2.372871 | 1.23E-12 |
| 13955 | Gm38576 | 2.371116 | 3.51E-06 |
| 27820 | Slc1a1 | 2.370985 | 3.32E-06 |
| 29166 | Sv2b | 2.368718 | 1.42E-05 |
| 3211 | Bhlhe40 | 2.36842 | 1.58E-14 |
| 28960 | Stac | 2.365467 | 7.89E-15 |
| 24105 | Parp10 | 2.364742 | 6.67E-08 |
| 17847 | Iqsec3 | 2.364478 | 0.005828 |
| 19234 | Lyz1 | 2.363627 | 0.003385 |
| 4963 | Colq | 2.363162 | 0.00188 |
| 4718 | Clec10a | 2.361731 | 0.001129 |
| 27261 | Scnn1b | 2.361538 | 0.001369 |
| 29780 | Thy1 | 2.35934 | 6.62E-23 |
| 7548 | Fmo2 | 2.358396 | 0.001543 |
| 1791 | Adamts2 | 2.357733 | 2.97E-15 |
| 29248 | Syt6 | 2.356595 | 3.74E-07 |
| 7372 | Fcgr3 | 2.35583 | 1.80E-11 |
| 2364 | Apba1 | 2.355782 | 7.08E-13 |
| 18457 | Krt1 | 2.355297 | 4.71E-07 |
| 32222 | Wnt3a | 2.353537 | 5.28E-05 |
| 16122 | Gna15 | 2.352462 | 0.000282 |
| 4048 | Cd14 | 2.352007 | 2.47E-08 |
| 21358 | Mtarc1 | 2.351447 | 0.001287 |
| 6818 | Ephx1 | 2.349433 | 1.31E-11 |
| 27095 | Samd9l | 2.348121 | 5.96E-12 |
| 7278 | Fbln7 | 2.344373 | 1.23E-10 |
| 2556 | Arl11 | 2.342699 | 0.000382 |
| 7795 | Gabrb1 | 2.340778 | 1.24E-15 |
| 23997 | P2ry12 | 2.3398 | 2.49E-05 |
| 5240 | Cspg5 | 2.338052 | 1.01E-13 |
| 3801 | Ccbe1 | 2.337222 | 1.64E-14 |
| 28864 | Srpx2 | 2.336891 | 3.29E-13 |
| 16311 | Gpr65 | 2.336043 | 9.34E-05 |
| 17654 | Il17rc | 2.334863 | 6.52E-06 |
| 1559 | Abcg3 | 2.334489 | 2.47E-06 |
| 4681 | Cldn1 | 2.334179 | 2.76E-16 |
| 24812 | Plekhg6 | 2.329918 | 1.65E-06 |
| 6335 | Dusp26 | 2.329377 | 0.002261 |
| 30759 | Trim9 | 2.329156 | 1.38E-12 |
| 1748 | Adam12 | 2.328267 | 2.08E-18 |
| 18092 | Kcng2 | 2.325506 | 0.000176 |
| 1804 | Adamtsl5 | 2.324266 | 2.50E-08 |
| 25678 | Ptpn5 | 2.323071 | 0 |
| 27545 | Sfrp5 | 2.319289 | 7.25E-05 |
| 6744 | Endou | 2.317413 | 0.001141 |
| 18501 | Krt80 | 2.313074 | 9.15E-05 |
| 30931 | Ttc22 | 2.311858 | 0.000124 |
| 4749 | Clec7a | 2.311294 | 0.001038 |
| 24884 | Pmaip1 | 2.306814 | 3.28E-09 |
| 30987 | Ttyh1 | 2.3064 | 0 |
| 21013 | Mlkl | 2.304961 | 0.001505 |
| 30710 | Trim30a | 2.304041 | 1.73E-08 |
| 6191 | Dock10 | 2.303754 | 4.04E-22 |
| 6193 | Dock2 | 2.30048 | 6.85E-12 |
| 4803 | Cmah | 2.295966 | 0.0003 |
| 30180 | Tmprss11b | 2.295713 | 0.00013 |
| 23998 | P2ry13 | 2.294296 | 0.000111 |
| 28913 | Sstr2 | 2.293468 | 0.000123 |
| 2628 | Arsi | 2.290798 | 1.42E-12 |
| 27316 | Sec1 | 2.285792 | 4.33E-06 |
| 3493 | C1s1 | 2.283168 | 1.68E-15 |
| 4885 | Cntn6 | 2.281196 | 8.74E-22 |
| 31444 | Vdr | 2.28043 | 3.15E-08 |
| 3779 | Cbr3 | 2.28018 | 1.28E-15 |
| 3440 | Btnl9 | 2.279152 | 4.52E-08 |
| 29868 | Tlr6 | 2.277193 | 1.29E-05 |
| 1806 | Adap2 | 2.276476 | 0.000113 |
| 17336 | Igfbp7 | 2.272876 | 4.60E-16 |
| 29505 | Tcf24 | 2.270134 | 7.31E-05 |
| 25958 | Rarres1 | 2.268887 | 0.000325 |
| 17850 | Irag2 | 2.267425 | 1.09E-08 |
| 4884 | Cntn5 | 2.266754 | 6.64E-07 |
| 7564 | Fndc1 | 2.266036 | 1.30E-14 |
| 7370 | Fcgr1 | 2.263779 | 3.12E-07 |
| 17089 | Hsd11b2 | 2.258196 | 6.33E-07 |
| 27639 | Shfl | 2.250285 | 2.38E-13 |
| 16779 | Hcls1 | 2.250222 | 6.55E-15 |
| 3474 | C1qb | 2.249851 | 2.21E-15 |
| 29742 | Thbd | 2.247693 | 1.59E-26 |
| 27464 | Serpinb1a | 2.247513 | 0.00027 |
| 24697 | Pkib | 2.243746 | 0.006637 |
| 29509 | Tcf7 | 2.240852 | 3.21E-17 |
| 2055 | Akr1c14 | 2.240477 | 0.000527 |
| 9037 | Gm14207 | 2.239905 | 0.002762 |
| 22588 | Olfml2b | 2.236676 | 1.39E-21 |
| 16567 | H2-D1 | 2.233397 | 2.26E-17 |
| 24761 | Plaur | 2.233365 | 2.97E-09 |
| 3933 | Ccdc88b | 2.22946 | 1.34E-07 |
| 16864 | Heyl | 2.229434 | 5.27E-14 |
| 4934 | Col28a1 | 2.229351 | 0.000697 |
| 29863 | Tlr13 | 2.226399 | 8.29E-08 |
| 3756 | Cav1 | 2.22346 | 3.60E-24 |
| 3429 | Btk | 2.222928 | 1.51E-07 |
| 16752 | Havcr2 | 2.22255 | 0.001117 |
| 32239 | Wscd1 | 2.221312 | 8.33E-08 |
| 31364 | Usp43 | 2.220733 | 1.12E-09 |
| 27371 | Selplg | 2.219649 | 6.88E-07 |
| 29754 | Themis2 | 2.215872 | 6.98E-06 |
| 4921 | Col16a1 | 2.211763 | 1.86E-16 |
| 21571 | Myo1g | 2.206906 | 5.45E-08 |
| 556 | 2900005J15Rik | 2.203915 | 0.000454 |
| 32068 | Vwa2 | 2.19922 | 0.001198 |
| 22084 | Nkx3-1 | 2.196497 | 6.91E-06 |
| 24108 | Parp14 | 2.193784 | 2.00E-10 |
| 6686 | Eln | 2.192679 | 8.36E-16 |
| 18626 | Laptm5 | 2.192286 | 2.91E-15 |
| 29822 | Tinagl1 | 2.189192 | 6.18E-10 |
| 28961 | Stac2 | 2.187891 | 2.87E-14 |
| 18738 | Lgals12 | 2.184818 | 0.000511 |
| 25969 | Rasd1 | 2.183732 | 1.48E-05 |
| 25989 | Rassf2 | 2.183717 | 2.22E-22 |
| 29024 | Stk32a | 2.181298 | 2.99E-08 |
| 30832 | Trpv2 | 2.180377 | 1.87E-09 |
| 16992 | Hopx | 2.180288 | 0.00101 |
| 29744 | Thbs2 | 2.18021 | 1.81E-20 |
| 18745 | Lgals9 | 2.179594 | 1.11E-19 |
| 25642 | Ptgs2 | 2.179068 | 7.77E-12 |
| 30331 | Tox2 | 2.178633 | 1.93E-06 |
| 3778 | Cbr2 | 2.178409 | 0.001006 |
| 18736 | Lfng | 2.175709 | 1.31E-09 |
| 28944 | St6galnac2 | 2.174367 | 1.28E-10 |
| 26074 | Rbp4 | 2.171536 | 8.29E-06 |
| 32058 | Vstm4 | 2.168228 | 1.94E-20 |
| 30880 | Tspan17 | 2.16814 | 8.03E-11 |
| 4233 | Cdk15 | 2.165588 | 0.000661 |
| 28147 | Slco2b1 | 2.164565 | 9.99E-11 |
| 7938 | Gckr | 2.162715 | 0.000731 |
| 32052 | Vsig8 | 2.161867 | 0.005017 |
| 28339 | Snhg11 | 2.160964 | 0 |
| 21548 | Myl9 | 2.160247 | 3.86E-23 |
| 1827 | Adcyap1r1 | 2.158111 | 0 |
| 31428 | Vav1 | 2.156374 | 2.84E-10 |
| 4727 | Clec2d | 2.156201 | 9.83E-10 |
| 28127 | Slc9a2 | 2.155977 | 0.000129 |
| 27524 | Sez6l | 2.155814 | 5.99E-05 |
| 5420 | Cygb | 2.153932 | 3.09E-19 |
| 17258 | Ifih1 | 2.152025 | 0.000492 |
| 7320 | Fbxo32 | 2.14858 | 1.09E-16 |
| 15821 | Gm8909 | 2.147593 | 0.002028 |
| 2659 | Asb18 | 2.143611 | 0.003507 |
| 24314 | Pde1b | 2.141216 | 2.24E-11 |
| 28200 | Smagp | 2.140905 | 8.57E-07 |
| 5568 | Cyth4 | 2.140733 | 1.02E-15 |
| 28146 | Slco2a1 | 2.14005 | 1.02E-21 |
| 1707 | Actg2 | 2.139907 | 2.66E-05 |
| 21059 | Mmp9 | 2.139079 | 2.44E-09 |
| 16798 | Hdac9 | 2.138432 | 1.07E-21 |
| 24787 | Pld5 | 2.13821 | 0.000198 |
| 1341 | a | 2.137754 | 0.000602 |
| 18156 | Kcns3 | 2.137411 | 2.52E-06 |
| 5440 | Cyp26c1 | 2.136271 | 0.006936 |
| 21114 | Moxd1 | 2.135332 | 7.77E-13 |
| 3489 | C1qtnf9 | 2.134974 | 0.007153 |
| 29241 | Syt15 | 2.13431 | 0.000336 |
| 29329 | Tagln2 | 2.133766 | 7.10E-19 |
| 25808 | Rab20 | 2.132418 | 0.001562 |
| 4260 | Cdkn1a | 2.130367 | 1.84E-09 |
| 17920 | Itgam | 2.127655 | 0.00012 |
| 28634 | Sparc | 2.126495 | 8.20E-17 |
| 24766 | Plcb2 | 2.126461 | 7.33E-08 |
| 24564 | Phyhipl | 2.125888 | 0 |
| 3367 | Bst2 | 2.118217 | 5.25E-10 |
| 18604 | Lamb3 | 2.117563 | 0.005712 |
| 7975 | Gem | 2.11733 | 5.21E-17 |
| 1548 | Abcc9 | 2.116967 | 0.003599 |
| 30233 | Tnfrsf1b | 2.114977 | 1.12E-08 |
| 4097 | Cd300lf | 2.110979 | 0.000196 |
| 25674 | Ptpn22 | 2.098886 | 1.54E-05 |
| 7785 | Gabbr2 | 2.098436 | 0.002206 |
| 16107 | Gmfg-ps | 2.098247 | 1.24E-06 |
| 4307 | Cebpd | 2.093539 | 1.64E-11 |
| 2629 | Arsj | 2.092764 | 1.85E-13 |
| 32087 | Was | 2.092456 | 6.37E-07 |
| 4917 | Col12a1 | 2.091656 | 5.65E-22 |
| 2454 | Arc | 2.091541 | 2.21E-06 |
| 7813 | Gadd45g | 2.091254 | 6.40E-11 |
| 16229 | Gper1 | 2.090313 | 1.52E-06 |
| 19716 | Mfsd6 | 2.089056 | 9.21E-09 |
| 5964 | Dhrs7 | 2.085524 | 4.48E-12 |
| 2988 | B4galnt1 | 2.08506 | 9.33E-12 |
| 5605 | D430036J16Rik | 2.084874 | 4.20E-06 |
| 2292 | Ano1 | 2.08418 | 1.81E-18 |
| 4103 | Cd36 | 2.081627 | 6.00E-07 |
| 4136 | Cd79b | 2.07514 | 2.66E-05 |
| 2256 | Ankrd33b | 2.072521 | 0.001891 |
| 24516 | Phf11d | 2.07183 | 0.004366 |
| 18576 | Ky | 2.071232 | 9.19E-05 |
| 2072 | Alas2 | 2.069184 | 1.13E-19 |
| 21349 | Mt1 | 2.069116 | 1.32E-08 |
| 7917 | Gbp3 | 2.066033 | 0.000671 |
| 27042 | S100a1 | 2.065975 | 8.79E-11 |
| 26991 | Rtn1 | 2.065844 | 0 |
| 18978 | Loxl4 | 2.061657 | 1.43E-08 |
| 16243 | Gpnmb | 2.057241 | 4.03E-05 |
| 4140 | Cd83 | 2.055562 | 4.97E-12 |
| 25635 | Ptgfr | 2.054274 | 7.91E-16 |
| 5214 | Csf3r | 2.052355 | 0.000688 |
| 27903 | Slc25a45 | 2.050782 | 6.55E-07 |
| 3355 | Brsk2 | 2.046918 | 0 |
| 7104 | Fam135b | 2.045806 | 0.000518 |
| 19198 | Ly86 | 2.043499 | 2.88E-05 |
| 5374 | Cx3cr1 | 2.043427 | 1.13E-16 |
| 24089 | Paqr6 | 2.04316 | 1.99E-06 |
| 30734 | Trim47 | 2.042724 | 9.31E-09 |
| 7544 | Fmnl1 | 2.040195 | 5.97E-10 |
| 2000 | Aif1 | 2.040033 | 4.68E-08 |
| 25974 | Rasgrf1 | 2.034959 | 0.000798 |
| 27490 | Serpine1 | 2.034669 | 3.63E-09 |
| 1805 | Adap1 | 2.033863 | 0.000138 |
| 18599 | Lama3 | 2.03087 | 1.20E-13 |
| 22105 | Nlrp3 | 2.030595 | 0.000102 |
| 27414 | Septin4 | 2.028858 | 3.37E-12 |
| 5962 | Dhrs3 | 2.028287 | 6.98E-16 |
| 25846 | Rab44 | 2.024958 | 0.003444 |
| 4022 | Ccr2 | 2.023017 | 1.20E-05 |
| 29930 | Tmem100 | 2.018873 | 1.96E-18 |
| 24937 | Podnl1 | 2.017411 | 6.45E-07 |
| 18994 | Lpl | 2.015438 | 5.71E-21 |
| 19504 | Mc5r | 2.014062 | 3.28E-08 |
| 17905 | Itga1 | 2.010175 | 1.18E-11 |
| 7583 | Fos | 2.010011 | 2.18E-12 |
| 10183 | Gm18980 | 2.007266 | 2.96E-06 |
| 18775 | Lif | 2.005821 | 8.58E-05 |
| 24107 | Parp12 | 2.005497 | 2.38E-14 |
| 30894 | Tspear | 2.004503 | 9.73E-05 |
| 32408 | Zbtb7b | 1.997683 | 3.50E-09 |
| 29869 | Tlr7 | 1.996174 | 8.70E-05 |
| 4648 | Ckb | 1.995964 | 5.14E-08 |
| 17709 | Il6ra | 1.994475 | 1.22E-07 |
| 19751 | Mical2 | 1.994112 | 8.05E-14 |
| 5142 | Crip1 | 1.99245 | 1.15E-08 |
| 2209 | Angpt2 | 1.992113 | 5.88E-09 |
| 27472 | Serpinb5 | 1.991768 | 2.78E-07 |
| 25649 | Pthlh | 1.990864 | 7.24E-17 |
| 20027 | Mir22hg | 1.990854 | 6.62E-06 |
| 26224 | Rgs14 | 1.987112 | 0.000182 |
| 767 | 4930474M22Rik | 1.985535 | 0.000195 |
| 2884 | AU021092 | 1.985083 | 0.001271 |
| 8057 | Gja5 | 1.983039 | 5.44E-06 |
| 7886 | Gas6 | 1.982925 | 2.99E-12 |
| 28100 | Slc6a4 | 1.982539 | 1.22E-06 |
| 4748 | Clec5a | 1.982152 | 0.002236 |
| 27045 | S100a13 | 1.980686 | 8.97E-12 |
| 32360 | Zar1 | 1.979831 | 0.002544 |
| 29574 | Tec | 1.979732 | 1.72E-09 |
| 27646 | Shisa7 | 1.979654 | 0 |
| 1587 | Abi3 | 1.978653 | 0.000265 |
| 27364 | Selenop | 1.977791 | 2.62E-19 |
| 19199 | Ly9 | 1.977496 | 0.000606 |
| 29498 | Tcerg1l | 1.977036 | 0.006583 |
| 28120 | Slc7a8 | 1.976969 | 6.91E-09 |
| 27861 | Slc24a3 | 1.976743 | 1.85E-12 |
| 25959 | Rarres2 | 1.976016 | 8.54E-06 |
| 6321 | Dusp1 | 1.975251 | 6.98E-13 |
| 24520 | Phf19 | 1.969603 | 3.66E-10 |
| 22204 | Npas4 | 1.96807 | 0.000202 |
| 16564 | H2-Aa | 1.966016 | 0.001432 |
| 32108 | Wdfy4 | 1.962167 | 1.51E-08 |
| 27238 | Scin | 1.9604 | 6.33E-06 |
| 7123 | Fam167b | 1.960086 | 7.58E-06 |
| 3511 | C3 | 1.959788 | 5.79E-05 |
| 16519 | Gucy2e | 1.958813 | 0.000204 |
| 27047 | S100a16 | 1.958035 | 2.45E-10 |
| 6344 | Dusp8 | 1.957556 | 4.90E-06 |
| 3479 | C1ql3 | 1.956699 | 0.001172 |
| 32155 | Wdr72 | 1.954867 | 0.000146 |
| 3616 | Calhm5 | 1.953566 | 3.73E-05 |
| 27525 | Sez6l2 | 1.951183 | 0.001368 |
| 1769 | Adam33 | 1.95113 | 2.13E-09 |
| 22359 | Ntn1 | 1.949765 | 0.002573 |
| 30008 | Tmem178b | 1.948466 | 0.004093 |
| 6672 | Elfn1 | 1.946543 | 7.88E-10 |
| 19116 | Lrrn2 | 1.946344 | 7.61E-08 |
| 7925 | Gbx2 | 1.943527 | 0.003885 |
| 5575 | D030025E07Rik | 1.941491 | 3.36E-05 |
| 5397 | Cxxc5 | 1.937946 | 2.52E-10 |
| 4149 | Cda | 1.935285 | 0.000465 |
| 21347 | Msx2 | 1.933149 | 1.86E-06 |
| 30225 | Tnfrsf12a | 1.932812 | 2.27E-09 |
| 6001 | Dipk1c | 1.928248 | 4.85E-12 |
| 4030 | Ccrl2 | 1.927588 | 1.11E-06 |
| 7476 | Fhl2 | 1.926937 | 7.70E-11 |
| 1167 | 5730416F02Rik | 1.925607 | 1.45E-06 |
| 27127 | Sash3 | 1.924079 | 8.83E-06 |
| 17206 | Icam1 | 1.923571 | 5.46E-09 |
| 27676 | Sigirr | 1.923007 | 8.45E-07 |
| 16383 | Grin2a | 1.922349 | 1.68E-08 |
| 3529 | C5ar1 | 1.921874 | 0.000406 |
| 5392 | Cxcr4 | 1.920171 | 1.16E-08 |
| 29218 | Syngr1 | 1.919063 | 0.001007 |
| 17371 | Ighm | 1.915315 | 7.92E-08 |
| 29215 | Syne3 | 1.914776 | 6.42E-07 |
| 19633 | Meox1 | 1.909233 | 7.23E-09 |
| 1700 | Acta2 | 1.908755 | 4.98E-12 |
| 4055 | Cd180 | 1.90782 | 3.50E-05 |
| 28581 | Sox17 | 1.903872 | 3.23E-07 |
| 4925 | Col1a1 | 1.903584 | 2.27E-13 |
| 3691 | Car3 | 1.899747 | 0.000124 |
| 19009 | Lrfn2 | 1.899521 | 2.61E-05 |
| 6780 | Entrep2 | 1.898452 | 3.65E-14 |
| 22367 | Ntrk2 | 1.898415 | 2.24E-13 |
| 25761 | Pyroxd2 | 1.896394 | 1.27E-10 |
| 2310 | Anxa1 | 1.895214 | 1.22E-14 |
| 18187 | Kdelr3 | 1.893885 | 2.26E-10 |
| 21782 | Nbl1 | 1.893884 | 6.47E-12 |
| 31262 | Unc45b | 1.893534 | 0.00013 |
| 17864 | Irf5 | 1.892948 | 1.57E-06 |
| 22202 | Npas2 | 1.891523 | 2.78E-05 |
| 28520 | Snx20 | 1.890157 | 0.000145 |
| 25679 | Ptpn6 | 1.888041 | 8.41E-10 |
| 31291 | Upp1 | 1.88736 | 4.43E-05 |
| 18862 | Lmx1a | 1.886718 | 0.001344 |
| 25742 | Pxdc1 | 1.884939 | 5.40E-10 |
| 27379 | Sema3g | 1.881446 | 8.19E-08 |
| 24275 | Pcp4 | 1.880334 | 0.003629 |
| 6497 | Edil3 | 1.879795 | 2.43E-14 |
| 2036 | Akap6 | 1.875117 | 1.03E-07 |
| 28331 | Snca | 1.87335 | 2.40E-10 |
| 7068 | Fah | 1.871217 | 2.35E-13 |
| 21705 | Naaladl2 | 1.869906 | 2.81E-07 |
| 16556 | H1f2 | 1.869461 | 6.45E-13 |
| 384 | 1810006J02Rik | 1.868446 | 0.004059 |
| 16907 | Hivep2 | 1.86769 | 3.59E-17 |
| 7371 | Fcgr2b | 1.864035 | 1.47E-06 |
| 1532 | Abcb1b | 1.863509 | 9.27E-06 |
| 2981 | B3gnt9 | 1.861143 | 3.73E-12 |
| 22090 | Nlgn1 | 1.859706 | 3.81E-14 |
| 17696 | Il33 | 1.859648 | 0.003198 |
| 22485 | Oasl2 | 1.856165 | 5.39E-07 |
| 21926 | Negr1 | 1.853854 | 9.27E-17 |
| 29322 | Tafa3 | 1.853208 | 3.98E-05 |
| 17697 | Il34 | 1.853035 | 0.001802 |
| 29866 | Tlr4 | 1.846642 | 5.62E-05 |
| 3704 | Card9 | 1.846133 | 7.05E-05 |
| 30213 | Tnf | 1.844635 | 0.002171 |
| 4118 | Cd52 | 1.844257 | 0.000952 |
| 2449 | Ar | 1.843714 | 5.98E-06 |
| 17692 | Il2rg | 1.843471 | 2.27E-05 |
| 5123 | Creb3l1 | 1.842919 | 5.73E-09 |
| 28543 | Socs3 | 1.839938 | 1.54E-13 |
| 17986 | Jam2 | 1.839294 | 1.23E-07 |
| 16740 | Has3 | 1.83695 | 1.18E-06 |
| 16384 | Grin2b | 1.836812 | 8.54E-06 |
| 28321 | Snap25 | 1.834742 | 0.001242 |
| 17081 | Hs3st6 | 1.834649 | 5.69E-09 |
| 26357 | Ripor2 | 1.833826 | 1.72E-06 |
| 27119 | Sardh | 1.832662 | 7.11E-13 |
| 21120 | Mpeg1 | 1.8325 | 2.32E-11 |
| 17876 | Irs4 | 1.829418 | 6.61E-08 |
| 18072 | Kcnab1 | 1.829254 | 1.85E-09 |
| 17244 | Ifi207 | 1.829018 | 4.14E-07 |
| 26412 | Rnf112 | 1.828413 | 5.82E-08 |
| 3761 | Cavin3 | 1.827887 | 1.20E-11 |
| 25768 | Qpct | 1.827779 | 1.21E-05 |
| 30666 | Trf | 1.827464 | 3.04E-10 |
| 27801 | Slc16a7 | 1.825435 | 7.65E-09 |
| 18298 | Klf14 | 1.825014 | 1.77E-08 |
| 30961 | Ttc9 | 1.824795 | 1.67E-05 |
| 16755 | Hba-a2 | 1.824703 | 1.32E-13 |
| 1974 | Ahrr | 1.824386 | 6.64E-10 |
| 28163 | Slfn5 | 1.819786 | 3.54E-09 |
| 26220 | Rgs10 | 1.81826 | 8.24E-09 |
| 1900 | Adssl1 | 1.815177 | 9.36E-06 |
| 27479 | Serpinb8 | 1.814992 | 1.04E-07 |
| 5179 | Cryab | 1.812538 | 3.66E-06 |
| 2407 | Apoe | 1.809055 | 8.05E-14 |
| 29774 | Thsd7a | 1.808649 | 2.32E-10 |
| 31270 | Unc79 | 1.80749 | 4.03E-05 |
| 3658 | Capg | 1.806098 | 9.56E-09 |
| 25983 | Rasl11a | 1.805283 | 2.45E-09 |
| 31056 | Txnip | 1.804712 | 3.87E-15 |
| 2829 | Atp6v0e2 | 1.804098 | 8.17E-08 |
| 17061 | Hr | 1.802245 | 1.23E-05 |
| 7890 | Gask1b | 1.800941 | 3.83E-10 |
| 2721 | Atcay | 1.800119 | 3.61E-06 |
| 26946 | Rsad2 | 1.799989 | 0.000298 |
| 22275 | Nr4a1 | 1.799191 | 4.04E-07 |
| 5642 | Dab1 | 1.799162 | 4.02E-06 |
| 1594 | Ablim3 | 1.79912 | 0.000837 |
| 26226 | Rgs17 | 1.798649 | 7.69E-10 |
| 6806 | Epha2 | 1.798528 | 2.38E-07 |
| 3466 | C130074G19Rik | 1.798085 | 1.24E-14 |
| 25316 | Prkcb | 1.795744 | 3.42E-06 |
| 16908 | Hivep3 | 1.794207 | 1.93E-07 |
| 17335 | Igfbp6 | 1.793313 | 0.002305 |
| 18847 | Lmna | 1.792868 | 1.13E-11 |
| 17268 | Ifitm3 | 1.792397 | 9.16E-10 |
| 16868 | Hgf | 1.79087 | 4.25E-09 |
| 17935 | Itgbl1 | 1.790802 | 4.56E-15 |
| 24395 | Pear1 | 1.789938 | 3.90E-11 |
| 24338 | Pdgfb | 1.789875 | 2.52E-08 |
| 16884 | Hid1 | 1.788964 | 5.26E-05 |
| 28284 | Smoc1 | 1.784911 | 6.96E-13 |
| 24360 | Pdlim2 | 1.783344 | 8.77E-10 |
| 27495 | Serping1 | 1.782012 | 2.70E-13 |
| 25377 | Prnp | 1.78035 | 2.70E-14 |
| 25702 | Ptprv | 1.779902 | 0.005272 |
| 24784 | Pld2 | 1.779892 | 1.50E-09 |
| 5207 | Csf1 | 1.77989 | 5.38E-14 |
| 26966 | Rspo2 | 1.7787 | 4.78E-15 |
| 16754 | Hba-a1 | 1.774743 | 7.17E-12 |
| 32053 | Vsir | 1.774155 | 5.82E-10 |
| 5639 | D930048N14Rik | 1.774061 | 9.08E-07 |
| 4145 | Cd9 | 1.772919 | 1.35E-07 |
| 18857 | Lmod1 | 1.772379 | 0.000425 |
| 24774 | Plcg2 | 1.768793 | 7.67E-09 |
| 6331 | Dusp2 | 1.768642 | 4.56E-07 |
| 32382 | Zbtb20 | 1.76766 | 1.52E-08 |
| 7586 | Fosl2 | 1.766856 | 5.65E-10 |
| 6050 | Dlx6os1 | 1.7657 | 3.97E-15 |
| 3521 | C3ar1 | 1.764441 | 9.85E-07 |
| 14728 | Gm5637 | 1.763983 | 2.76E-10 |
| 5111 | Cracr2b | 1.762159 | 1.35E-05 |
| 16732 | Hapln3 | 1.758015 | 1.08E-05 |
| 6720 | Emid1 | 1.757716 | 1.74E-12 |
| 1960 | Agtr2 | 1.756841 | 2.78E-14 |
| 4888 | Cntnap3 | 1.756739 | 0.003281 |
| 29476 | Tbxa2r | 1.755397 | 0.000307 |
| 30379 | Trabd2b | 1.753737 | 1.76E-12 |
| 19778 | Milr1 | 1.752563 | 0.001529 |
| 16573 | H2-K1 | 1.752454 | 1.20E-11 |
| 30165 | Tmem95 | 1.748756 | 0.001638 |
| 27061 | S1pr1 | 1.745117 | 1.74E-14 |
| 16363 | Grem2 | 1.744678 | 2.13E-15 |
| 27747 | Sla | 1.742299 | 4.68E-06 |
| 22287 | Nrbp2 | 1.740052 | 1.27E-09 |
| 3699 | Card10 | 1.739581 | 1.29E-08 |
| 7452 | Fgfbp1 | 1.736899 | 0.003107 |
| 28119 | Slc7a7 | 1.733678 | 8.85E-08 |
| 29791 | Tifab | 1.732029 | 0.000359 |
| 28824 | Sqor | 1.731723 | 2.42E-07 |
| 24760 | Plau | 1.729387 | 4.81E-13 |
| 2675 | Ascl5 | 1.728611 | 5.79E-05 |
| 4256 | Cdkl2 | 1.727199 | 0.000322 |
| 19128 | Lsamp | 1.726566 | 2.21E-12 |
| 4098 | Cd300lg | 1.726557 | 0.003238 |
| 16121 | Gna14 | 1.724798 | 0.0067 |
| 28958 | Stab1 | 1.723097 | 7.83E-14 |
| 2480 | Arhgap15 | 1.722982 | 0.001574 |
| 17769 | Inpp5d | 1.722783 | 9.17E-09 |
| 29173 | Svep1 | 1.718909 | 6.50E-13 |
| 3757 | Cav2 | 1.718305 | 3.61E-09 |
| 2135 | Alox5ap | 1.71414 | 0.000137 |
| 18872 | Lnx1 | 1.713834 | 2.17E-07 |
| 19395 | Map6 | 1.710473 | 0.000137 |
| 27621 | Sh3tc1 | 1.710412 | 1.90E-07 |
| 2396 | Apobec1 | 1.70965 | 3.57E-07 |
| 26519 | Robo4 | 1.709044 | 1.98E-12 |
| 28762 | Spon2 | 1.707205 | 7.75E-07 |
| 24112 | Parp4 | 1.706437 | 7.50E-11 |
| 22310 | Nrros | 1.706434 | 2.19E-09 |
| 2088 | Aldh3b1 | 1.705067 | 0.001326 |
| 21801 | Ncf1 | 1.702824 | 7.42E-07 |
| 27374 | Sema3b | 1.702687 | 3.48E-05 |
| 26405 | Rnd1 | 1.699618 | 0.002164 |
| 31026 | Tunar | 1.698731 | 0.007087 |
| 24805 | Plekhf1 | 1.698726 | 4.29E-08 |
| 25684 | Ptprc | 1.695468 | 1.57E-08 |
| 19403 | Mapk10 | 1.692476 | 0.003527 |
| 25949 | Rapgef4 | 1.692385 | 1.89E-07 |
| 16353 | Grap | 1.689577 | 9.01E-07 |
| 1794 | Adamts4 | 1.688113 | 1.59E-08 |
| 7815 | Gadl1 | 1.687738 | 1.93E-05 |
| 4134 | Cd74 | 1.687474 | 4.98E-05 |
| 2961 | B2m | 1.685475 | 1.09E-13 |
| 1562 | Abcg8 | 1.684878 | 0.000785 |
| 5916 | Depp1 | 1.684273 | 0.000737 |
| 25628 | Ptger2 | 1.684147 | 0.000103 |
| 1531 | Abcb1a | 1.683939 | 0.004371 |
| 5296 | Ctla2a | 1.683351 | 3.30E-05 |
| 8056 | Gja4 | 1.683117 | 3.40E-06 |
| 30888 | Tspan4 | 1.680796 | 2.35E-08 |
| 3980 | Ccn1 | 1.680213 | 1.43E-09 |
| 4290 | Ceacam1 | 1.680122 | 0.004725 |
| 28754 | Spns2 | 1.679063 | 7.32E-09 |
| 17232 | Ier2 | 1.67877 | 5.93E-09 |
| 26274 | Rhov | 1.676624 | 0.006183 |
| 23986 | Oxtr | 1.675613 | 0.000329 |
| 19157 | Ltbp4 | 1.674167 | 1.73E-10 |
| 19342 | Man2b2 | 1.673033 | 9.56E-10 |
| 24085 | Papss2 | 1.667085 | 2.36E-13 |
| 4554 | Chodl | 1.666682 | 1.02E-16 |
| 26394 | Rnase4 | 1.666251 | 3.64E-09 |
| 17254 | Ifi35 | 1.663612 | 9.89E-08 |
| 25471 | Prss23 | 1.662544 | 8.33E-12 |
| 5559 | Cys1 | 1.661919 | 4.82E-06 |
| 16266 | Gpr153 | 1.660395 | 2.32E-08 |
| 6341 | Dusp5 | 1.660172 | 3.80E-07 |
| 30386 | Traf3ip3 | 1.657066 | 0.004436 |
| 29477 | Tbxas1 | 1.65608 | 0.001216 |
| 1511 | AB124611 | 1.65498 | 0.000259 |
| 2368 | Apbb1ip | 1.654784 | 6.86E-10 |
| 7927 | Gca | 1.654564 | 0.002259 |
| 6899 | Esam | 1.653937 | 5.67E-08 |
| 30179 | Tmprss11a | 1.651866 | 0.002774 |
| 1890 | Adra2a | 1.651453 | 1.05E-06 |
| 8037 | Gimap8 | 1.65046 | 3.90E-06 |
| 4946 | Col6a1 | 1.649431 | 4.39E-12 |
| 7547 | Fmo1 | 1.647177 | 1.94E-11 |
| 6479 | Ecm2 | 1.646424 | 1.49E-06 |
| 6966 | Evi2a | 1.64567 | 0.002761 |
| 19615 | Megf11 | 1.644406 | 1.80E-06 |
| 26460 | Rnf207 | 1.641499 | 0.005867 |
| 24620 | Pik3cd | 1.641384 | 1.96E-09 |
| 6788 | Epas1 | 1.641211 | 1.07E-09 |
| 6731 | Emp2 | 1.640183 | 4.72E-08 |
| 6986 | Exoc3l4 | 1.638919 | 8.86E-06 |
| 29252 | Sytl1 | 1.638691 | 0.00151 |
| 4663 | Clca2 | 1.637543 | 0.000953 |
| 1474 | AA414768 | 1.636277 | 1.38E-05 |
| 7410 | Fermt3 | 1.633518 | 2.32E-08 |
| 31323 | Ushbp1 | 1.630474 | 1.75E-09 |
| 7907 | Gatm | 1.629259 | 4.78E-10 |
| 5412 | Cybb | 1.626382 | 1.27E-06 |
| 25670 | Ptpn18 | 1.625324 | 0.000442 |
| 30236 | Tnfrsf23 | 1.621964 | 0.000205 |
| 22243 | Nptxr | 1.620009 | 1.17E-09 |
| 29705 | Tgfb1 | 1.61812 | 2.36E-05 |
| 6261 | Dram1 | 1.617298 | 1.35E-05 |
| 25127 | Ppp1r16b | 1.616291 | 8.05E-07 |
| 2451 | Arap1 | 1.615993 | 1.04E-10 |
| 27620 | Sh3rf3 | 1.615584 | 1.16E-09 |
| 16625 | H2ac25 | 1.614642 | 3.94E-06 |
| 3157 | Bcl3 | 1.61349 | 0.000161 |
| 1972 | Ahnak2 | 1.613042 | 0.006639 |
| 25680 | Ptpn7 | 1.612328 | 2.91E-05 |
| 30224 | Tnfrsf11b | 1.611977 | 1.98E-06 |
| 27002 | Rtp4 | 1.610975 | 0.000301 |
| 15927 | Gm9240 | 1.610869 | 0.002931 |
| 3334 | Brdt | 1.610847 | 5.60E-10 |
| 18165 | Kctd11 | 1.602996 | 3.67E-07 |
| 8969 | Gm13889 | 1.602897 | 5.36E-07 |
| 19555 | Mdga1 | 1.600181 | 2.13E-08 |
| 21496 | Mxra8 | 1.600057 | 3.32E-06 |
| 3740 | Castor1 | 1.598329 | 7.34E-05 |
| 16432 | Gsdmd | 1.597598 | 3.01E-09 |
| 27649 | Shisal1 | 1.596309 | 1.69E-07 |
| 7254 | Fas | 1.595636 | 0.000107 |
| 21802 | Ncf2 | 1.59559 | 0.00352 |
| 1783 | Adamts12 | 1.594737 | 2.48E-09 |
| 7082 | Fam110d | 1.594219 | 0.000268 |
| 24060 | Palmd | 1.593895 | 1.29E-05 |
| 21846 | Ndrg1 | 1.59363 | 6.14E-07 |
| 25683 | Ptprb | 1.593104 | 7.48E-08 |
| 17608 | Igsf11 | 1.59307 | 0.000883 |
| 19205 | Lyn | 1.592304 | 1.15E-10 |
| 5204 | Csdc2 | 1.592076 | 1.27E-06 |
| 6040 | Dll4 | 1.591801 | 8.79E-06 |
| 25837 | Rab3d | 1.59068 | 1.02E-09 |
| 22183 | Nos2 | 1.590476 | 0.000154 |
| 7812 | Gadd45b | 1.588338 | 2.72E-06 |
| 21515 | Myct1 | 1.586104 | 6.10E-07 |
| 18971 | Lonrf3 | 1.583486 | 0.000202 |
| 24823 | Plekhs1 | 1.582907 | 0.003472 |
| 2783 | Atp1b2 | 1.579884 | 1.32E-07 |
| 21605 | Myzap | 1.579152 | 4.36E-10 |
| 4146 | Cd93 | 1.577452 | 2.03E-11 |
| 17716 | Ildr2 | 1.576297 | 0.005309 |
| 22212 | Npdc1 | 1.573448 | 2.57E-09 |
| 4954 | Col8a2 | 1.573138 | 2.10E-11 |
| 21516 | Myd88 | 1.573102 | 1.95E-09 |
| 3043 | Batf3 | 1.572004 | 1.41E-07 |
| 28595 | Sox8 | 1.568564 | 0.000114 |
| 31266 | Unc5b | 1.567611 | 6.18E-07 |
| 4722 | Clec14a | 1.56705 | 2.27E-09 |
| 18064 | Kcna1 | 1.56564 | 0.000108 |
| 24076 | Panx3 | 1.564899 | 1.54E-06 |
| 1362 | A2m | 1.564656 | 0.004258 |
| 2513 | Arhgap9 | 1.562446 | 0.000222 |
| 26332 | Rimbp2 | 1.56203 | 0.002479 |
| 2029 | Akap12 | 1.561708 | 2.32E-12 |
| 26210 | Rgcc | 1.561403 | 6.83E-06 |
| 32216 | Wnt10b | 1.559324 | 6.93E-05 |
| 1580 | Abhd3 | 1.557189 | 0.004953 |
| 27480 | Serpinb9 | 1.554467 | 7.97E-06 |
| 3759 | Cavin1 | 1.553619 | 2.69E-14 |
| 19333 | Mamld1 | 1.553321 | 1.45E-07 |
| 8033 | Gimap4 | 1.55222 | 1.23E-09 |
| 15852 | Gm8995 | 1.551027 | 0.002676 |
| 28161 | Slfn3 | 1.550627 | 0.001721 |
| 17891 | Islr | 1.549775 | 4.38E-08 |
| 2016 | Ak1 | 1.549686 | 2.86E-09 |
| 4216 | Cdh8 | 1.549457 | 9.92E-06 |
| 3578 | Cacna2d3 | 1.548565 | 8.00E-05 |
| 29860 | Tlr1 | 1.546709 | 0.000326 |
| 28844 | Srgn | 1.538968 | 0.000329 |
| 2550 | Arid5a | 1.538362 | 1.91E-06 |
| 2151 | Amacr | 1.537513 | 1.44E-11 |
| 3831 | Ccdc141 | 1.537156 | 3.72E-07 |
| 24273 | Pcolce2 | 1.534282 | 3.16E-06 |
| 21550 | Mylk | 1.533411 | 6.43E-08 |
| 30361 | Tppp3 | 1.531572 | 9.22E-06 |
| 3638 | Camk2n1 | 1.529822 | 2.70E-07 |
| 18083 | Kcnd3 | 1.529742 | 8.40E-07 |
| 6003 | Dipk2b | 1.529339 | 0.001188 |
| 3477 | C1ql1 | 1.529035 | 2.46E-06 |
| 18133 | Kcnk6 | 1.527672 | 6.85E-07 |
| 5518 | Cyp46a1 | 1.527613 | 5.95E-06 |
| 6310 | Dtx3l | 1.526523 | 1.51E-08 |
| 19345 | Manba | 1.524523 | 2.52E-08 |
| 5662 | Dapk2 | 1.521334 | 0.000223 |
| 28498 | Snta1 | 1.518242 | 3.92E-09 |
| 26518 | Robo3 | 1.51764 | 0.000876 |
| 6897 | Errfi1 | 1.515883 | 8.31E-12 |
| 7923 | Gbp9 | 1.515413 | 0.00011 |
| 5414 | Cybrd1 | 1.514556 | 2.30E-13 |
| 26251 | Rhbdl3 | 1.51154 | 7.69E-06 |
| 22039 | Nim1k | 1.508622 | 4.76E-05 |
| 22153 | Nod1 | 1.508246 | 1.00E-07 |
| 1522 | Abca4 | 1.5049 | 9.25E-08 |
| 1857 | Adgrl4 | 1.502375 | 2.93E-06 |
| 28594 | Sox7 | 1.500285 | 2.36E-07 |
| 17993 | Jdp2 | 1.498343 | 1.15E-07 |
| 4711 | Cldn5 | 1.498004 | 9.39E-07 |
| 8015 | Ggt5 | 1.497743 | 0.000236 |
| 4903 | Cobll1 | 1.496435 | 1.84E-08 |
| 5680 | Dbndd2 | 1.495752 | 0.001533 |
| 5243 | Csrnp1 | 1.495239 | 1.78E-07 |
| 5615 | D630033O11Rik | 1.493316 | 0.002254 |
| 3557 | Cabp1 | 1.49315 | 0.001425 |
| 16312 | Gpr68 | 1.492866 | 0.006876 |
| 4547 | Chmp4c | 1.490189 | 0.000477 |
| 16154 | Gngt2 | 1.490082 | 0.00059 |
| 7790 | Gabra2 | 1.488713 | 1.19E-05 |
| 19696 | Mfng | 1.488543 | 7.11E-07 |
| 16729 | Hap1 | 1.487981 | 0.000556 |
| 31412 | Vamp5 | 1.487761 | 7.09E-05 |
| 29253 | Sytl2 | 1.487631 | 6.33E-06 |
| 17269 | Ifitm5 | 1.486583 | 0.003372 |
| 7456 | Fgfr2 | 1.484298 | 3.14E-09 |
| 29770 | Thrb | 1.484008 | 0.003936 |
| 28715 | Sphk1 | 1.483719 | 0.00054 |
| 4113 | Cd44 | 1.481584 | 4.61E-10 |
| 17156 | Htr1b | 1.479902 | 1.41E-06 |
| 29706 | Tgfb1i1 | 1.479774 | 4.32E-08 |
| 7026 | F2rl1 | 1.479071 | 0.004771 |
| 20991 | Mitf | 1.479033 | 3.04E-09 |
| 17626 | Ikzf1 | 1.478669 | 0.000185 |
| 2316 | Anxa4 | 1.477737 | 4.71E-12 |
| 21963 | Neurl1b | 1.477225 | 4.31E-06 |
| 17857 | Irf1 | 1.477203 | 3.23E-12 |
| 28972 | Stap2 | 1.476787 | 1.72E-06 |
| 27766 | Slc11a1 | 1.474191 | 0.003218 |
| 14561 | Gm5200 | 1.474156 | 5.52E-06 |
| 17705 | Il4ra | 1.473374 | 6.89E-09 |
| 2312 | Anxa11 | 1.473109 | 2.74E-08 |
| 6569 | Egr1 | 1.472586 | 1.60E-08 |
| 19335 | Man1a | 1.471635 | 9.98E-11 |
| 3836 | Ccdc149 | 1.467873 | 2.25E-07 |
| 7432 | Fgf11 | 1.467255 | 1.96E-07 |
| 22364 | Ntng2 | 1.467128 | 0.001219 |
| 3659 | Capn1 | 1.463786 | 2.34E-07 |
| 30328 | Tor3a | 1.461035 | 1.46E-06 |
| 28032 | Slc41a2 | 1.46075 | 0.000191 |
| 1538 | Abcb9 | 1.459712 | 0.002643 |
| 7833 | Galnt12 | 1.459676 | 0.000474 |
| 31430 | Vav3 | 1.458651 | 8.74E-08 |
| 24454 | Pfkp | 1.456862 | 2.95E-07 |
| 29690 | Tfeb | 1.45466 | 1.99E-06 |
| 29790 | Tifa | 1.453999 | 7.53E-05 |
| 32814 | Zfp853 | 1.453494 | 5.64E-09 |
| 17050 | Hpgds | 1.45039 | 6.08E-06 |
| 7744 | Fyb | 1.448369 | 3.98E-07 |
| 25813 | Rab25 | 1.448266 | 0.006276 |
| 2437 | Aqp1 | 1.447507 | 1.13E-06 |
| 16356 | Grb14 | 1.446852 | 5.35E-07 |
| 2021 | Ak5 | 1.445195 | 0.00162 |
| 4105 | Cd38 | 1.444358 | 2.34E-05 |
| 18465 | Krt18 | 1.4419 | 2.53E-07 |
| 1985 | AI467606 | 1.441841 | 0.002613 |
| 16419 | Gsap | 1.441722 | 6.05E-06 |
| 22233 | Npr1 | 1.441703 | 1.83E-06 |
| 18741 | Lgals3bp | 1.440898 | 8.96E-07 |
| 4102 | Cd34 | 1.440281 | 3.34E-11 |
| 4578 | Chrna7 | 1.439623 | 0.000254 |
| 17913 | Itga6 | 1.43651 | 2.25E-09 |
| 24815 | Plekhh3 | 1.435962 | 2.22E-06 |
| 2598 | Armh1 | 1.434099 | 0.001353 |
| 29758 | Thnsl2 | 1.433672 | 5.90E-05 |
| 24792 | Plek2 | 1.433193 | 0.000625 |
| 28582 | Sox18 | 1.432652 | 6.19E-07 |
| 6043 | Dlx1as | 1.432253 | 6.56E-08 |
| 31009 | Tubb6 | 1.431918 | 3.15E-09 |
| 4212 | Cdh4 | 1.431909 | 8.47E-05 |
| 3158 | Bcl6 | 1.428672 | 7.09E-05 |
| 4267 | Cdkn2c | 1.428258 | 1.29E-08 |
| 28499 | Sntb1 | 1.427049 | 5.49E-09 |
| 19104 | Lrrc8e | 1.42298 | 0.000477 |
| 7741 | Fxyd5 | 1.422354 | 1.37E-07 |
| 4995 | Copz2 | 1.422275 | 2.26E-11 |
| 26197 | Rftn1 | 1.421377 | 2.03E-09 |
| 4789 | Clstn2 | 1.421141 | 2.48E-09 |
| 24115 | Parp9 | 1.419999 | 1.01E-07 |
| 21499 | Myb | 1.419312 | 1.17E-06 |
| 19718 | Mfsd7a | 1.418895 | 0.002335 |
| 4602 | Chsy3 | 1.417711 | 4.58E-09 |
| 17332 | Igfbp3 | 1.417625 | 2.96E-14 |
| 16858 | Hexb | 1.417417 | 5.85E-10 |
| 4953 | Col8a1 | 1.414504 | 2.45E-09 |
| 24745 | Plat | 1.414048 | 1.53E-09 |
| 3494 | C1s2 | 1.413059 | 0.003581 |
| 19272 | Mafb | 1.409226 | 2.72E-07 |
| 24398 | Pecam1 | 1.406695 | 2.88E-11 |
| 31274 | Unc93b1 | 1.406653 | 2.67E-07 |
| 29344 | Tap1 | 1.405674 | 4.20E-07 |
| 7889 | Gask1a | 1.404362 | 0.000162 |
| 5031 | Cox4i2 | 1.404158 | 5.99E-07 |
| 14666 | Gm5424 | 1.404091 | 0.000759 |
| 1803 | Adamtsl4 | 1.404039 | 0.000208 |
| 4306 | Cebpb | 1.403144 | 0.003051 |
| 6279 | Dsc2 | 1.402123 | 0.000369 |
| 17092 | Hsd17b11 | 1.401647 | 2.27E-08 |
| 18353 | Klhl6 | 1.400417 | 1.39E-06 |
| 7163 | Fam20c | 1.397642 | 1.62E-05 |
| 2142 | Alpl | 1.397411 | 1.37E-06 |
| 26464 | Rnf213 | 1.396298 | 3.90E-08 |
| 3981 | Ccn2 | 1.395887 | 3.39E-09 |
| 18998 | Lpxn | 1.395591 | 0.000709 |
| 21978 | Nfam1 | 1.394177 | 7.49E-07 |
| 8034 | Gimap5 | 1.391328 | 0.002889 |
| 28887 | Srxn1 | 1.391151 | 6.20E-08 |
| 18856 | Lmo7 | 1.390907 | 6.51E-11 |
| 16632 | H2aj | 1.390903 | 4.38E-06 |
| 18630 | Larp1b | 1.390003 | 0.001759 |
| 1852 | Adgrg6 | 1.386878 | 9.47E-11 |
| 5398 | Cyb561 | 1.383198 | 0.00044 |
| 2341 | Ap1s2 | 1.383032 | 3.46E-08 |
| 19729 | Mgat4c | 1.382954 | 0.001121 |
| 29817 | Timp1 | 1.382807 | 5.77E-09 |
| 3632 | Camk1d | 1.38184 | 3.95E-08 |
| 30818 | Trpc6 | 1.38111 | 0.001492 |
| 22657 | Optn | 1.380102 | 1.57E-05 |
| 6058 | Dmc1 | 1.37998 | 0.005624 |
| 31460 | Vim | 1.37854 | 7.02E-10 |
| 27129 | Sat1 | 1.378177 | 7.54E-10 |
| 7064 | Fads3 | 1.377606 | 6.01E-09 |
| 19384 | Map3k6 | 1.376193 | 0.001007 |
| 24954 | Pold4 | 1.372772 | 2.36E-06 |
| 17919 | Itgal | 1.372735 | 0.000375 |
| 4615 | Cib2 | 1.372123 | 2.61E-07 |
| 2608 | Arpc1b | 1.371545 | 3.67E-09 |
| 22298 | Nrgn | 1.370826 | 6.07E-05 |
| 16668 | H2bc4 | 1.369989 | 6.56E-06 |
| 32433 | Zc3hav1 | 1.369056 | 5.55E-07 |
| 27361 | Selenom | 1.368848 | 1.21E-08 |
| 1640 | Ache | 1.368379 | 0.000722 |
| 22393 | Nudt16 | 1.367409 | 0.000123 |
| 24122 | Parvg | 1.36692 | 0.000127 |
| 8100 | Glis1 | 1.366278 | 7.15E-05 |
| 19354 | Maob | 1.365456 | 8.22E-08 |
| 3205 | Bgn | 1.365444 | 2.59E-10 |
| 17051 | Hpn | 1.364147 | 0.001265 |
| 16918 | Hlx | 1.363335 | 0.000412 |
| 24090 | Paqr7 | 1.362507 | 0.000236 |
| 5246 | Csrp1 | 1.360419 | 6.46E-08 |
| 27912 | Slc26a10 | 1.359885 | 0.000748 |
| 7542 | Fmn1 | 1.359762 | 3.83E-08 |
| 16284 | Gpr183 | 1.359733 | 0.000291 |
| 6482 | Ecscr | 1.358393 | 4.26E-05 |
| 21847 | Ndrg2 | 1.356512 | 4.39E-07 |
| 6016 | Dkk2 | 1.355584 | 1.46E-09 |
| 3082 | BC028528 | 1.355548 | 0.000903 |
| 28759 | Spock2 | 1.352989 | 5.26E-10 |
| 16397 | Grk3 | 1.352496 | 2.63E-05 |
| 19505 | Mcam | 1.352151 | 2.39E-08 |
| 1912 | Afap1l2 | 1.352063 | 2.76E-05 |
| 30030 | Tmem200b | 1.351247 | 3.04E-07 |
| 4124 | Cd59b | 1.350755 | 0.004301 |
| 19556 | Mdga2 | 1.350672 | 5.93E-06 |
| 6746 | Eng | 1.349227 | 1.89E-07 |
| 16377 | Grik2 | 1.34868 | 0.000273 |
| 22224 | Npl | 1.347392 | 3.68E-06 |
| 7453 | Fgfbp3 | 1.346905 | 0.003332 |
| 23938 | Ostf1 | 1.344211 | 8.10E-09 |
| 19204 | Lyl1 | 1.343185 | 9.63E-05 |
| 16376 | Grik1 | 1.342199 | 1.59E-08 |
| 27169 | Scarf1 | 1.341423 | 9.35E-07 |
| 29346 | Tapbp | 1.340407 | 8.40E-09 |
| 24272 | Pcolce | 1.340054 | 7.67E-11 |
| 30887 | Tspan33 | 1.339948 | 0.00436 |
| 28115 | Slc7a4 | 1.338469 | 0.000293 |
| 7690 | Fst | 1.337869 | 3.47E-07 |
| 6819 | Ephx2 | 1.337567 | 0.000114 |
| 29789 | Tie1 | 1.337411 | 2.28E-08 |
| 26345 | Rinl | 1.33649 | 0.001402 |
| 25977 | Rasgrp2 | 1.336445 | 6.13E-05 |
| 4174 | Cdc42ep2 | 1.336374 | 3.43E-05 |
| 25755 | Pygl | 1.336254 | 7.84E-10 |
| 27668 | Shtn1 | 1.333305 | 6.63E-05 |
| 2260 | Ankrd35 | 1.33314 | 0.006546 |
| 24170 | Pcbd1 | 1.33256 | 2.96E-05 |
| 5143 | Crip2 | 1.325555 | 7.18E-06 |
| 28982 | Stard8 | 1.324157 | 1.68E-07 |
| 3226 | Bin2 | 1.323848 | 0.002061 |
| 18016 | Jun | 1.323298 | 1.04E-06 |
| 17132 | Hspa4l | 1.323223 | 1.51E-05 |
| 21578 | Myo6 | 1.319897 | 7.50E-09 |
| 26103 | Rcsd1 | 1.319746 | 3.98E-09 |
| 24087 | Paqr4 | 1.319226 | 8.11E-05 |
| 18762 | Lhfpl4 | 1.317342 | 0.003381 |
| 18343 | Klhl33 | 1.316773 | 0.00594 |
| 25381 | Procr | 1.3163 | 0.000542 |
| 22194 | Nova2 | 1.315801 | 0.000188 |
| 30135 | Tmem63a | 1.315798 | 4.18E-06 |
| 16875 | Hhex | 1.313797 | 0.000932 |
| 17756 | Inka2 | 1.312715 | 0.005433 |
| 27311 | Sdr42e1 | 1.310765 | 0.00258 |
| 2317 | Anxa5 | 1.31073 | 3.88E-09 |
| 17449 | Igip | 1.308973 | 0.000197 |
| 22000 | Nfkbid | 1.308919 | 0.000734 |
| 4305 | Cebpa | 1.307568 | 2.05E-05 |
| 25394 | Pros1 | 1.30717 | 2.94E-10 |
| 7084 | Fam114a1 | 1.306729 | 2.50E-10 |
| 4952 | Col7a1 | 1.304955 | 0.000133 |
| 3732 | Casp8 | 1.304297 | 3.95E-08 |
| 8992 | Gm14032 | 1.304128 | 3.41E-08 |
| 16819 | Hebp1 | 1.300366 | 4.12E-07 |
| 19466 | Matn2 | 1.298215 | 7.77E-10 |
| 1089 | 4933421O10Rik | 1.29768 | 0.001831 |
| 28298 | Smpdl3a | 1.297224 | 8.31E-06 |
| 22184 | Nos3 | 1.296127 | 3.44E-05 |
| 6714 | Emc9 | 1.294763 | 0.004468 |
| 18103 | Kcnip1 | 1.294372 | 0.00586 |
| 3774 | Cbln2 | 1.292865 | 0.001122 |
| 7374 | Fcgrt | 1.292661 | 2.03E-07 |
| 24798 | Plekha6 | 1.291434 | 0.000146 |
| 4947 | Col6a2 | 1.28967 | 6.97E-10 |
| 4938 | Col4a2 | 1.289274 | 1.40E-11 |
| 25520 | Psd4 | 1.288839 | 3.97E-05 |
| 1571 | Abhd14b | 1.287794 | 0.000915 |
| 6199 | Dock8 | 1.287705 | 1.60E-06 |
| 26269 | Rhoj | 1.287354 | 6.26E-09 |
| 30243 | Tnfsf11 | 1.28708 | 2.26E-05 |
| 30895 | Tspo | 1.284014 | 7.83E-05 |
| 7468 | Fhdc1 | 1.283263 | 1.02E-05 |
| 17633 | Il10rb | 1.282724 | 2.77E-06 |
| 29894 | Tmbim1 | 1.282327 | 2.20E-06 |
| 31081 | Uap1l1 | 1.281622 | 4.48E-05 |
| 3690 | Car2 | 1.279119 | 2.43E-07 |
| 19068 | Lrrc4 | 1.278503 | 0.000215 |
| 6905 | Esm1 | 1.278191 | 0.004058 |
| 17207 | Icam2 | 1.277563 | 1.12E-05 |
| 5058 | Cpe | 1.27755 | 3.30E-11 |
| 19614 | Megf10 | 1.276924 | 7.45E-07 |
| 4566 | Chrm2 | 1.276453 | 2.75E-05 |
| 1781 | Adamts1 | 1.276189 | 2.82E-07 |
| 8018 | Ggta1 | 1.274331 | 1.91E-07 |
| 30350 | Tpm1 | 1.27372 | 1.03E-11 |
| 29566 | Tdrd7 | 1.273634 | 2.30E-06 |
| 16535 | Gypa | 1.273026 | 0.003301 |
| 5154 | Crlf1 | 1.272956 | 7.10E-08 |
| 18853 | Lmo2 | 1.271807 | 6.73E-07 |
| 26920 | Rrad | 1.271605 | 0.005337 |
| 16237 | Gpld1 | 1.271148 | 0.000385 |
| 6564 | Egflam | 1.270781 | 1.41E-09 |
| 30217 | Tnfaip6 | 1.267215 | 3.08E-07 |
| 1211 | 6530402F18Rik | 1.267177 | 0.001634 |
| 16465 | Gstt1 | 1.266931 | 6.25E-05 |
| 24181 | Pcdh12 | 1.266227 | 4.77E-06 |
| 17057 | Hps6 | 1.265729 | 0.00127 |
| 6769 | Entpd1 | 1.265567 | 5.24E-09 |
| 25629 | Ptger3 | 1.265564 | 0.003082 |
| 16951 | Hmox1 | 1.265038 | 1.44E-07 |
| 7792 | Gabra4 | 1.263632 | 1.24E-05 |
| 16143 | Gng11 | 1.262983 | 1.58E-07 |
| 18076 | Kcnb2 | 1.262718 | 0.000184 |
| 2167 | Amigo2 | 1.261712 | 1.88E-05 |
| 2515 | Arhgdib | 1.258376 | 1.31E-05 |
| 21713 | Nacc2 | 1.257122 | 2.75E-06 |
| 25612 | Ptch1 | 1.257089 | 1.84E-07 |
| 19172 | Luzp2 | 1.256617 | 0.00163 |
| 29775 | Thsd7b | 1.254682 | 2.82E-05 |
| 4937 | Col4a1 | 1.253648 | 2.97E-10 |
| 19099 | Lrrc75b | 1.253152 | 0.002061 |
| 30958 | Ttc7 | 1.252551 | 3.00E-05 |
| 2453 | Arap3 | 1.252291 | 2.65E-07 |
| 3598 | Cadm3 | 1.250304 | 1.20E-07 |
| 3179 | Bean1 | 1.249552 | 0.003593 |
| 2493 | Arhgap27 | 1.249208 | 9.06E-06 |
| 17253 | Ifi30 | 1.248658 | 2.82E-07 |
| 16379 | Grik4 | 1.248153 | 6.02E-05 |
| 19406 | Mapk13 | 1.248101 | 1.53E-05 |
| 25950 | Rapgef5 | 1.247188 | 7.60E-07 |
| 29026 | Stk32c | 1.247012 | 0.000894 |
| 21911 | Necab3 | 1.246102 | 0.000557 |
| 6578 | Ehd4 | 1.244876 | 2.28E-07 |
| 6038 | Dll1 | 1.244871 | 0.000842 |
| 21525 | Myh11 | 1.243213 | 9.27E-05 |
| 2789 | Atp2a3 | 1.24256 | 0.004363 |
| 5052 | Cpa4 | 1.241316 | 0.000791 |
| 26144 | Relb | 1.2398 | 1.19E-05 |
| 2485 | Arhgap20 | 1.239651 | 5.48E-07 |
| 21579 | Myo7a | 1.239508 | 0.000167 |
| 5571 | Cyyr1 | 1.239357 | 6.00E-06 |
| 30258 | Tnk1 | 1.239185 | 0.001365 |
| 2511 | Arhgap6 | 1.238351 | 4.31E-06 |
| 29219 | Syngr2 | 1.23786 | 9.11E-07 |
| 24674 | Pitpnm3 | 1.236104 | 0.000202 |
| 21721 | Naglu | 1.235305 | 2.15E-06 |
| 22349 | Nt5dc3 | 1.234145 | 5.40E-05 |
| 6723 | Emilin3 | 1.232996 | 3.96E-09 |
| 22035 | Nid1 | 1.232799 | 4.16E-10 |
| 28242 | Smim1 | 1.232702 | 9.75E-05 |
| 4886 | Cntnap1 | 1.232464 | 0.000805 |
| 25980 | Rasip1 | 1.231874 | 1.23E-05 |
| 32903 | Zfpm2 | 1.231236 | 1.41E-06 |
| 5411 | Cyba | 1.230486 | 2.36E-06 |
| 18299 | Klf15 | 1.230063 | 0.001662 |
| 27927 | Slc27a6 | 1.229781 | 2.58E-06 |
| 16605 | H2-T23 | 1.229555 | 0.000537 |
| 26086 | Rcan2 | 1.22924 | 0.000341 |
| 24111 | Parp3 | 1.226844 | 1.17E-06 |
| 23990 | P2rx4 | 1.226041 | 9.16E-06 |
| 7176 | Fam222a | 1.225489 | 0.0018 |
| 25641 | Ptgs1 | 1.225323 | 2.02E-05 |
| 30923 | Ttc12 | 1.221561 | 0.000284 |
| 3979 | Ccm2l | 1.221278 | 4.83E-05 |
| 29707 | Tgfb2 | 1.221035 | 5.50E-08 |
| 6574 | Ehbp1l1 | 1.220965 | 1.05E-06 |
| 4918 | Col13a1 | 1.219727 | 1.20E-06 |
| 29865 | Tlr3 | 1.21897 | 0.000702 |
| 5999 | Dipk1a | 1.218833 | 4.83E-08 |
| 28541 | Socs1 | 1.218676 | 0.000337 |
| 19464 | Matk | 1.218176 | 1.26E-05 |
| 32410 | Zbtb8a | 1.216786 | 0.000199 |
| 8035 | Gimap6 | 1.215507 | 4.22E-08 |
| 26989 | Rtl8c | 1.215027 | 1.46E-05 |
| 7533 | Flt1 | 1.214865 | 9.10E-09 |
| 6722 | Emilin2 | 1.214856 | 2.71E-09 |
| 22363 | Ntng1 | 1.213857 | 2.90E-08 |
| 5322 | Ctsb | 1.212919 | 2.60E-09 |
| 2295 | Ano3 | 1.212206 | 0.002872 |
| 1973 | Ahr | 1.211318 | 2.59E-07 |
| 30836 | Trpv6 | 1.209891 | 0.000264 |
| 1821 | Adcy5 | 1.20953 | 2.34E-05 |
| 24310 | Pde10a | 1.209432 | 6.29E-05 |
| 3261 | Bmp4 | 1.209234 | 1.35E-05 |
| 30215 | Tnfaip2 | 1.207144 | 1.68E-05 |
| 7337 | Fbxo6 | 1.206919 | 4.69E-05 |
| 3159 | Bcl6b | 1.206409 | 3.22E-06 |
| 7551 | Fmo5 | 1.206295 | 0.005245 |
| 25854 | Rab6b | 1.203656 | 7.02E-07 |
| 18751 | Lgmn | 1.202861 | 2.57E-08 |
| 145 | 1700019D03Rik | 1.202391 | 7.48E-05 |
| 28033 | Slc41a3 | 1.201496 | 0.000524 |
| 25013 | Pon2 | 1.201332 | 1.56E-07 |
| 27726 | Skap2 | 1.200716 | 0.000448 |
| 29255 | Sytl4 | 1.200034 | 8.54E-07 |
| 6576 | Ehd2 | 1.199328 | 1.40E-07 |
| 29063 | Strip2 | 1.198046 | 0.000982 |
| 27491 | Serpine2 | 1.197754 | 8.87E-07 |
| 19121 | Lrrtm2 | 1.195589 | 0.000679 |
| 1841 | Adgre5 | 1.195105 | 7.83E-06 |
| 29772 | Thsd1 | 1.194784 | 3.79E-05 |
| 4792 | Cltb | 1.192641 | 1.67E-05 |
| 18095 | Kcnh1 | 1.192158 | 0.004156 |
| 6017 | Dkk3 | 1.191347 | 1.35E-08 |
| 6562 | Egfl7 | 1.190785 | 1.35E-07 |
| 9516 | Gm16364 | 1.190651 | 0.006734 |
| 16149 | Gng4 | 1.189347 | 2.87E-05 |
| 28998 | Steap3 | 1.18823 | 0.000291 |
| 19405 | Mapk12 | 1.188204 | 0.000817 |
| 24769 | Plcd1 | 1.187804 | 1.35E-05 |
| 5713 | Dclk1 | 1.18634 | 8.66E-09 |
| 4598 | Chst7 | 1.185994 | 0.000963 |
| 30359 | Tppp | 1.185126 | 0.00076 |
| 27290 | Sdc4 | 1.184524 | 2.58E-07 |
| 2373 | Apcdd1 | 1.183589 | 4.11E-07 |
| 19060 | Lrrc32 | 1.182577 | 0.000146 |
| 17672 | Il1rl2 | 1.181082 | 3.03E-05 |
| 8156 | Gm10125 | 1.181059 | 0.002961 |
| 5075 | Cpne2 | 1.181049 | 5.68E-05 |
| 25080 | Ppic | 1.180342 | 4.54E-08 |
| 7428 | Fgd5 | 1.180188 | 1.69E-06 |
| 26260 | Rhob | 1.179841 | 2.20E-06 |
| 26171 | Retreg1 | 1.17942 | 3.34E-06 |
| 25978 | Rasgrp3 | 1.177359 | 2.05E-05 |
| 32067 | Vwa1 | 1.176483 | 2.19E-05 |
| 7491 | Filip1 | 1.176076 | 0.000188 |
| 3790 | Cbx7 | 1.174181 | 0.000271 |
| 7020 | F11r | 1.173215 | 8.28E-05 |
| 4960 | Colec12 | 1.171628 | 7.33E-09 |
| 18273 | Kifc3 | 1.171569 | 1.26E-06 |
| 5387 | Cxcl5 | 1.170648 | 4.35E-05 |
| 7449 | Fgf7 | 1.170647 | 5.35E-05 |
| 30244 | Tnfsf12 | 1.170354 | 6.39E-05 |
| 32596 | Zfp366 | 1.16926 | 1.27E-05 |
| 18306 | Klf6 | 1.168369 | 1.35E-08 |
| 4129 | Cd68 | 1.168145 | 0.000268 |
| 3340 | Brinp1 | 1.167189 | 7.20E-06 |
| 3983 | Ccn4 | 1.165458 | 6.57E-08 |
| 30329 | Tor4a | 1.165126 | 0.001032 |
| 1642 | Ackr1 | 1.164355 | 0.001673 |
| 1749 | Adam15 | 1.16362 | 1.41E-05 |
| 2387 | Aplnr | 1.163338 | 1.11E-07 |
| 5665 | Dapp1 | 1.162318 | 4.25E-05 |
| 22003 | Nfkbiz | 1.162073 | 2.58E-07 |
| 22397 | Nudt18 | 1.16178 | 0.000618 |
| 24936 | Podn | 1.161074 | 0.000162 |
| 25159 | Ppp2r2b | 1.158755 | 3.85E-06 |
| 5186 | Crybg1 | 1.157189 | 0.00231 |
| 29537 | Tcta | 1.156769 | 1.98E-05 |
| 25948 | Rapgef3 | 1.155527 | 5.32E-06 |
| 24920 | Pnp | 1.152099 | 0.000333 |
| 1846 | Adgrf5 | 1.151607 | 1.31E-06 |
| 4397 | Cerox1 | 1.151087 | 0.000199 |
| 3482 | C1qtnf12 | 1.150077 | 3.12E-06 |
| 24627 | Pik3r5 | 1.150025 | 0.000644 |
| 21909 | Necab1 | 1.148754 | 0.003051 |
| 7502 | Fkbp14 | 1.148118 | 9.41E-08 |
| 28590 | Sox5 | 1.148065 | 1.52E-05 |
| 25458 | Prrt1 | 1.147943 | 0.000596 |
| 5110 | Cracr2a | 1.147743 | 0.000168 |
| 6742 | Endod1 | 1.146836 | 1.73E-06 |
| 4213 | Cdh5 | 1.145736 | 1.49E-08 |
| 30875 | Tspan11 | 1.145078 | 4.97E-06 |
| 32217 | Wnt11 | 1.144781 | 0.000161 |
| 16409 | Grn | 1.144182 | 1.75E-06 |
| 5328 | Ctsh | 1.143852 | 0.001297 |
| 2509 | Arhgap45 | 1.141917 | 0.000121 |
| 18208 | Kdr | 1.141604 | 6.14E-07 |
| 21497 | Myadm | 1.140159 | 8.98E-07 |
| 24881 | Plxnd1 | 1.139363 | 1.13E-06 |
| 24276 | Pcp4l1 | 1.136389 | 0.000822 |
| 1725 | Actr3b | 1.135533 | 0.00063 |
| 6783 | Eogt | 1.134892 | 3.64E-05 |
| 27877 | Slc25a21 | 1.134816 | 0.001466 |
| 30688 | Trib2 | 1.134383 | 9.35E-07 |
| 19192 | Ly6h | 1.133532 | 0.000277 |
| 24628 | Pik3r6 | 1.131852 | 0.001947 |
| 28760 | Spock3 | 1.131839 | 0.002545 |
| 26426 | Rnf135 | 1.1314 | 3.82E-05 |
| 25924 | Ramp3 | 1.131258 | 0.004943 |
| 21947 | Nell2 | 1.131137 | 0.001224 |
| 2911 | AW011738 | 1.130456 | 0.000703 |
| 18748 | Lgi2 | 1.130068 | 0.000453 |
| 4951 | Col6a6 | 1.127911 | 0.00011 |
| 1823 | Adcy7 | 1.127911 | 4.74E-05 |
| 27569 | Sgk1 | 1.127391 | 1.03E-06 |
| 24869 | Plvap | 1.127213 | 9.97E-06 |
| 17052 | Hprt | 1.126934 | 8.20E-08 |
| 19117 | Lrrn3 | 1.126793 | 0.000252 |
| 24836 | Plk3 | 1.126496 | 4.02E-05 |
| 29883 | Tm6sf1 | 1.126457 | 0.000499 |
| 8017 | Ggt7 | 1.125341 | 0.000449 |
| 27937 | Slc2a10 | 1.12507 | 7.95E-05 |
| 29589 | Tek | 1.124016 | 4.63E-07 |
| 1927 | Agap2 | 1.122982 | 8.27E-06 |
| 5383 | Cxcl16 | 1.120024 | 0.000414 |
| 22190 | Notch4 | 1.11943 | 0.000334 |
| 29697 | Tfpi | 1.119215 | 5.32E-07 |
| 8053 | Gja1 | 1.119211 | 4.31E-06 |
| 16123 | Gnai1 | 1.119095 | 2.44E-07 |
| 29223 | Synj2 | 1.11906 | 1.35E-05 |
| 2828 | Atp6v0e | 1.117961 | 5.89E-05 |
| 29202 | Syk | 1.11717 | 8.09E-06 |
| 30134 | Tmem62 | 1.116138 | 0.001918 |
| 1902 | Aebp1 | 1.114818 | 4.01E-07 |
| 16513 | Gucy1a1 | 1.114809 | 1.64E-06 |
| 30351 | Tpm2 | 1.114475 | 1.07E-07 |
| 21488 | Mvp | 1.112959 | 6.72E-05 |
| 16115 | Gmpr | 1.112142 | 5.82E-05 |
| 21852 | Ndst3 | 1.111254 | 3.40E-05 |
| 30128 | Tmem53 | 1.11007 | 0.004161 |
| 4915 | Col11a1 | 1.109632 | 3.16E-08 |
| 7213 | Fam81a | 1.109365 | 0.001086 |
| 6764 | Enpp5 | 1.109172 | 3.90E-06 |
| 6732 | Emp3 | 1.106344 | 4.14E-05 |
| 19273 | Maff | 1.106147 | 8.93E-06 |
| 24363 | Pdlim5 | 1.104504 | 1.17E-05 |
| 25965 | Rasa4 | 1.100384 | 3.78E-06 |
| 13371 | Gm29778 | 1.100208 | 0.002116 |
| 25630 | Ptger4 | 1.10009 | 0.00396 |
| 24783 | Pld1 | 1.099682 | 1.44E-05 |
| 32055 | Vstm2a | 1.098058 | 0.001055 |
| 19361 | Map2 | 1.097202 | 3.46E-08 |
| 29905 | Tmc8 | 1.09512 | 0.0049 |
| 29937 | Tmem107 | 1.094457 | 9.78E-05 |
| 24343 | Pdgfrl | 1.094411 | 5.16E-05 |
| 16349 | Gramd1c | 1.093804 | 4.03E-05 |
| 18348 | Klhl4 | 1.093567 | 0.000409 |
| 26358 | Ripor3 | 1.093268 | 0.001045 |
| 3487 | C1qtnf6 | 1.09302 | 2.80E-07 |
| 24470 | Pgbd5 | 1.089544 | 0.000413 |
| 4942 | Col4a6 | 1.089489 | 0.000148 |
| 25144 | Ppp1r3c | 1.088749 | 0.000315 |
| 18593 | Lag3 | 1.088732 | 0.003022 |
| 24543 | Phlda1 | 1.088487 | 0.003172 |
| 17867 | Irf8 | 1.087185 | 0.000444 |
| 558 | 2900026A02Rik | 1.086492 | 0.000194 |
| 7657 | Frmd3 | 1.08618 | 0.005316 |
| 17624 | Ikbke | 1.08275 | 0.000222 |
| 27043 | S100a10 | 1.082443 | 1.41E-05 |
| 2618 | Arrb1 | 1.08101 | 2.22E-06 |
| 8624 | Gm12420 | 1.080992 | 0.000115 |
| 19170 | Lurap1l | 1.080706 | 8.51E-05 |
| 21181 | Mrgpre | 1.079702 | 0.001045 |
| 28136 | Slc9a9 | 1.07921 | 0.000264 |
| 5008 | Coro1a | 1.078979 | 0.00013 |
| 4482 | Cgas | 1.078043 | 9.95E-05 |
| 5757 | Ddit3 | 1.075963 | 1.12E-05 |
| 32718 | Zfp641 | 1.074707 | 0.000996 |
| 5101 | Cpxm1 | 1.074434 | 6.47E-08 |
| 3177 | Bdnf | 1.07369 | 0.001006 |
| 5324 | Ctsd | 1.072114 | 1.14E-05 |
| 21979 | Nfasc | 1.071373 | 0.005528 |
| 24670 | Pitpnm1 | 1.070846 | 0.000205 |
| 25100 | Ppm1h | 1.069769 | 0.001121 |
| 29912 | Tmco4 | 1.069022 | 0.000617 |
| 3481 | C1qtnf1 | 1.068653 | 0.000552 |
| 28041 | Slc44a5 | 1.068532 | 7.67E-06 |
| 4920 | Col15a1 | 1.06817 | 3.15E-06 |
| 7457 | Fgfr3 | 1.06353 | 0.002253 |
| 21687 | N4bp3 | 1.063288 | 0.000138 |
| 4936 | Col3a1 | 1.058795 | 5.55E-07 |
| 21055 | Mmp28 | 1.058441 | 0.001737 |
| 24545 | Phlda3 | 1.057648 | 2.58E-05 |
| 7747 | Fyn | 1.056748 | 4.56E-07 |
| 28074 | Slc5a3 | 1.056206 | 1.85E-05 |
| 2318 | Anxa6 | 1.055185 | 1.60E-07 |
| 30834 | Trpv4 | 1.053093 | 0.001155 |
| 4822 | Cmtm8 | 1.052856 | 0.005838 |
| 18742 | Lgals4 | 1.052426 | 0.001027 |
| 28607 | Sp7 | 1.051813 | 0.00209 |
| 6761 | Enpp2 | 1.051754 | 1.47E-05 |
| 21992 | Nfic | 1.051739 | 1.48E-05 |
| 26238 | Rgs7bp | 1.051545 | 0.000506 |
| 23896 | Orai1 | 1.050645 | 0.001324 |
| 7929 | Gcc1 | 1.05013 | 1.85E-06 |
| 5937 | Dgka | 1.049248 | 0.000293 |
| 2075 | Alcam | 1.048545 | 2.91E-06 |
| 6545 | Efhd2 | 1.048417 | 1.44E-05 |
| 4047 | Cd109 | 1.047762 | 1.29E-05 |
| 7501 | Fkbp11 | 1.04718 | 9.15E-05 |
| 29438 | Tbc1d8b | 1.046499 | 3.16E-05 |
| 14914 | Gm6149 | 1.046336 | 0.00542 |
| 2342 | Ap1s3 | 1.045203 | 0.00114 |
| 26153 | Renbp | 1.044931 | 1.22E-05 |
| 24621 | Pik3cg | 1.044723 | 0.000325 |
| 31001 | Tubb2a | 1.044067 | 0.004196 |
| 7652 | Frem2 | 1.042905 | 2.70E-06 |
| 2253 | Ankrd29 | 1.042765 | 2.77E-05 |
| 2491 | Arhgap25 | 1.042526 | 0.005074 |
| 29864 | Tlr2 | 1.042386 | 2.02E-05 |
| 28270 | Smim4 | 1.041579 | 0.001973 |
| 2866 | Atxn1 | 1.038678 | 0.000282 |
| 3126 | Bcar3 | 1.037781 | 0.00075 |
| 6002 | Dipk2a | 1.037477 | 2.46E-05 |
| 3193 | Bet1l | 1.035925 | 0.000529 |
| 18843 | Lmcd1 | 1.035317 | 5.11E-05 |
| 31436 | Vcan | 1.034749 | 1.59E-05 |
| 5745 | Dcxr | 1.030478 | 0.00112 |
| 1203 | 6430548M08Rik | 1.030454 | 3.33E-05 |
| 26884 | Rps6ka4 | 1.028937 | 7.81E-05 |
| 30118 | Tmem45a | 1.028776 | 1.31E-05 |
| 29855 | Tll1 | 1.028331 | 0.000836 |
| 1277 | 9330102E08Rik | 1.026213 | 0.001924 |
| 5339 | Ctsz | 1.025716 | 4.30E-06 |
| 4614 | Cib1 | 1.025299 | 6.98E-05 |
| 21781 | Nbeal2 | 1.02404 | 0.003525 |
| 14777 | Gm5749 | 1.023951 | 0.003017 |
| 29818 | Timp2 | 1.023464 | 8.14E-07 |
| 26433 | Rnf144b | 1.02176 | 0.003844 |
| 1081 | 4933416I08Rik | 1.020722 | 0.002193 |
| 2529 | Arhgef26 | 1.020227 | 1.03E-05 |
| 2820 | Atp6ap2 | 1.017497 | 1.52E-06 |
| 5334 | Ctso | 1.015542 | 0.000158 |
| 32402 | Zbtb46 | 1.013975 | 0.000615 |
| 15668 | Gm8436 | 1.013607 | 4.72E-05 |
| 6670 | Elf4 | 1.012722 | 0.000529 |
| 6464 | Ebp | 1.010337 | 2.26E-05 |
| 21343 | Mst1r | 1.01009 | 0.004771 |
| 28984 | Stat1 | 1.009996 | 0.000669 |
| 27354 | Selenbp1 | 1.009325 | 0.001867 |
| 4198 | Cdh12 | 1.009317 | 0.002035 |
| 28114 | Slc7a3 | 1.008853 | 0.006781 |
| 4849 | Cnn2 | 1.008144 | 1.37E-06 |
| 18136 | Kcnma1 | 1.00784 | 0.002636 |
| 6311 | Dtx4 | 1.006995 | 7.13E-06 |
| 7188 | Fam241a | 1.006246 | 0.003558 |
| 16598 | H2-Q4 | 1.00602 | 0.000899 |
| 30115 | Tmem42 | 1.004115 | 0.001387 |
| 7411 | Fes | 1.00384 | 0.000152 |
| 28336 | Sned1 | 1.0034 | 0.001311 |
| 27960 | Slc31a2 | 1.002745 | 0.0006 |
| 1322 | 9530077C05Rik | 1.002616 | 0.003975 |
| 16269 | Gpr157 | 1.001949 | 0.0011 |
| 4923 | Col18a1 | 1.001876 | 9.74E-05 |
| 1655 | Acot11 | 1.000795 | 0.002212 |
| 27307 | Sdk2 | 0.99958 | 5.47E-05 |
| 2483 | Arhgap18 | 0.999335 | 3.86E-05 |
| 17321 | Igf1 | 0.998695 | 9.38E-05 |
| 5170 | Crtap | 0.997676 | 1.23E-06 |
| 16260 | Gpr146 | 0.996719 | 0.004701 |
| 18020 | Jup | 0.994365 | 0.005915 |
| 17293 | Ifngr1 | 0.99402 | 2.14E-05 |
| 19154 | Ltbp1 | 0.992993 | 7.29E-07 |
| 31038 | Twist2 | 0.992599 | 9.96E-05 |
| 28990 | Stat6 | 0.992365 | 9.61E-05 |
| 6339 | Dusp3 | 0.992237 | 0.000238 |
| 21748 | Naprt | 0.991191 | 0.000259 |
| 21282 | Mrps6 | 0.990701 | 0.000102 |
| 21041 | Mmp14 | 0.990601 | 6.35E-06 |
| 16340 | Gpx3 | 0.990383 | 0.000998 |
| 18598 | Lama2 | 0.990091 | 2.29E-05 |
| 19024 | Lrp10 | 0.989743 | 0.000196 |
| 21908 | Nebl | 0.989016 | 0.000984 |
| 4948 | Col6a3 | 0.988744 | 0.000144 |
| 18633 | Larp6 | 0.986221 | 0.000879 |
| 22229 | Npnt | 0.985618 | 1.21E-06 |
| 4590 | Chst12 | 0.985604 | 0.000804 |
| 24291 | Pcyt1a | 0.985072 | 2.06E-06 |
| 19095 | Lrrc73 | 0.984699 | 0.007221 |
| 19181 | Ly6e | 0.98434 | 0.000839 |
| 4128 | Cd63-ps | 0.983267 | 4.32E-06 |
| 19762 | Mid2 | 0.982476 | 1.95E-05 |
| 28035 | Slc43a2 | 0.981748 | 0.000745 |
| 28125 | Slc8b1 | 0.978952 | 0.000339 |
| 31129 | Ube2l6 | 0.978875 | 0.000446 |
| 26079 | Rbpms | 0.978846 | 0.000132 |
| 21061 | Mmrn2 | 0.978818 | 5.40E-05 |
| 30175 | Tmod3 | 0.977835 | 2.45E-07 |
| 27161 | Scamp5 | 0.977683 | 2.97E-05 |
| 26327 | Rigi | 0.977174 | 0.000198 |
| 27268 | Scpep1 | 0.975969 | 1.51E-06 |
| 17333 | Igfbp4 | 0.974624 | 1.31E-05 |
| 30130 | Tmem59 | 0.973099 | 3.68E-05 |
| 30256 | Tnip2 | 0.972843 | 0.000492 |
| 6386 | Dysf | 0.970196 | 0.000124 |
| 28530 | Snx32 | 0.968168 | 0.000966 |
| 13797 | Gm3549 | 0.967133 | 0.004673 |
| 6956 | Etv4 | 0.966461 | 0.000655 |
| 2620 | Arrdc1 | 0.965883 | 0.000392 |
| 19692 | Mfge8 | 0.965812 | 0.000511 |
| 26925 | Rras | 0.96581 | 0.000404 |
| 30231 | Tnfrsf19 | 0.965763 | 2.50E-05 |
| 2567 | Arl4a | 0.965545 | 2.49E-05 |
| 7858 | Gap43 | 0.964209 | 0.004154 |
| 4394 | Cercam | 0.962878 | 0.000203 |
| 16877 | Hhipl1 | 0.962489 | 0.005276 |
| 26836 | Rps2 | 0.962405 | 0.006094 |
| 2218 | Ank | 0.962063 | 1.64E-05 |
| 1931 | Agbl3 | 0.960902 | 0.000747 |
| 27700 | Sirpa | 0.960091 | 9.58E-06 |
| 1627 | Acbd4 | 0.959916 | 0.000603 |
| 26517 | Robo2 | 0.959866 | 3.96E-05 |
| 7277 | Fbln5 | 0.959 | 5.58E-06 |
| 28970 | Stambpl1 | 0.958843 | 0.000521 |
| 26248 | Rhbdf2 | 0.958782 | 0.001696 |
| 18240 | Kif1a | 0.957403 | 0.001673 |
| 27248 | Scn1b | 0.957352 | 0.00227 |
| 16807 | Hdhd3 | 0.957161 | 0.005837 |
| 22240 | Nptn | 0.956392 | 1.49E-06 |
| 16345 | Gpx7 | 0.955683 | 7.38E-05 |
| 25899 | Radil | 0.954462 | 0.004291 |
| 4820 | Cmtm6 | 0.954221 | 2.51E-05 |
| 17853 | Irak2 | 0.953518 | 0.007114 |
| 25275 | Prdx5 | 0.952985 | 5.27E-05 |
| 27932 | Slc29a1 | 0.952914 | 0.000208 |
| 844 | 4930523C07Rik | 0.952862 | 0.004538 |
| 7135 | Fam174b | 0.952781 | 4.39E-05 |
| 15334 | Gm7537 | 0.952758 | 0.001383 |
| 18091 | Kcng1 | 0.952321 | 0.004219 |
| 21988 | Nfe2l2 | 0.952199 | 1.14E-05 |
| 15143 | Gm6942 | 0.951246 | 0.003765 |
| 30201 | Tmsb4x | 0.94764 | 8.87E-05 |
| 24938 | Podxl | 0.946656 | 1.24E-05 |
| 3667 | Capn5 | 0.946636 | 0.001158 |
| 6161 | Dnase1l1 | 0.946625 | 0.002626 |
| 1676 | Acpp | 0.945676 | 0.004821 |
| 24875 | Plxna4 | 0.944953 | 0.002399 |
| 24313 | Pde1a | 0.944867 | 0.000273 |
| 25750 | Pycard | 0.944816 | 0.003916 |
| 24206 | Pcdhb11 | 0.944711 | 0.005446 |
| 14534 | Gm5131 | 0.944606 | 0.001024 |
| 17908 | Itga2 | 0.94321 | 5.61E-05 |
| 4812 | Cmpk2 | 0.942702 | 0.00164 |
| 19643 | Mest | 0.942191 | 1.51E-06 |
| 19175 | Lxn | 0.941602 | 0.000229 |
| 8102 | Glis3 | 0.939741 | 0.001506 |
| 18452 | Kremen1 | 0.938404 | 0.001009 |
| 29516 | Tcim | 0.937916 | 5.37E-06 |
| 6106 | Dnah8 | 0.936465 | 0.006027 |
| 31455 | Vgll3 | 0.935068 | 2.64E-05 |
| 16536 | Gypc | 0.934975 | 1.70E-05 |
| 24066 | Pamr1 | 0.934856 | 0.004544 |
| 30889 | Tspan5 | 0.934811 | 0.000563 |
| 32213 | Wnk4 | 0.933 | 0.001751 |
| 5434 | Cyp20a1 | 0.932468 | 0.000229 |
| 7499 | Fjx1 | 0.932294 | 0.001383 |
| 2386 | Apln | 0.931481 | 4.55E-05 |
| 21335 | Msn | 0.931147 | 1.27E-06 |
| 29921 | Tmed3 | 0.930418 | 1.85E-05 |
| 27428 | Serinc3 | 0.930295 | 1.09E-06 |
| 17742 | Inf2 | 0.930068 | 0.002 |
| 27571 | Sgk3 | 0.930051 | 0.000217 |
| 30153 | Tmem86a | 0.929508 | 0.000498 |
| 26071 | Rbp1 | 0.929136 | 2.22E-05 |
| 25751 | Pycr1 | 0.929101 | 0.000184 |
| 17153 | Htatip2 | 0.928677 | 0.004282 |
| 19101 | Lrrc8b | 0.928139 | 1.31E-05 |
| 26032 | Rbm20 | 0.928037 | 0.004009 |
| 29167 | Sv2c | 0.927952 | 0.000188 |
| 25513 | Psap | 0.927603 | 6.20E-05 |
| 6933 | Esyt1 | 0.927039 | 2.61E-05 |
| 28116 | Slc7a5 | 0.92697 | 0.00211 |
| 26965 | Rspo1 | 0.926826 | 0.000391 |
| 24867 | Pltp | 0.926802 | 0.004564 |
| 19338 | Man1c1 | 0.926787 | 5.84E-05 |
| 16730 | Hapln1 | 0.926291 | 0.002534 |
| 21016 | Mllt11 | 0.92547 | 0.002497 |
| 3934 | Ccdc88c | 0.924898 | 0.00314 |
| 6945 | Ethe1 | 0.923651 | 0.00156 |
| 31425 | Vat1 | 0.922895 | 3.88E-05 |
| 2765 | Atox1 | 0.921539 | 0.000424 |
| 29334 | Tamalin | 0.92073 | 0.000193 |
| 209 | 1700029J07Rik | 0.919233 | 0.006595 |
| 22288 | Nrcam | 0.919036 | 0.000136 |
| 29213 | Syne1 | 0.918582 | 0.000138 |
| 24414 | Per1 | 0.916236 | 0.000835 |
| 6753 | Eno2 | 0.9157 | 0.000347 |
| 5903 | Dennd3 | 0.914533 | 0.002953 |
| 31448 | Vegfd | 0.912632 | 0.001032 |
| 22589 | Olfml3 | 0.911903 | 9.21E-06 |
| 25819 | Rab29 | 0.910962 | 0.001013 |
| 2531 | Arhgef3 | 0.909298 | 0.000213 |
| 32469 | Zdhhc24 | 0.908452 | 0.001448 |
| 24871 | Plxdc2 | 0.906944 | 4.60E-05 |
| 30704 | Trim25 | 0.905547 | 0.000397 |
| 21706 | Nab1 | 0.905455 | 1.24E-05 |
| 8083 | Glb1l2 | 0.902621 | 0.003749 |
| 4563 | Chrdl1 | 0.90207 | 0.000311 |
| 24209 | Pcdhb14 | 0.901699 | 0.002046 |
| 27892 | Slc25a35 | 0.901178 | 0.005923 |
| 21703 | Naalad2 | 0.901174 | 7.02E-05 |
| 16526 | Gusb | 0.90045 | 1.37E-05 |
| 6730 | Emp1 | 0.900285 | 1.16E-05 |
| 24791 | Plek | 0.899175 | 0.001038 |
| 17945 | Itm2b | 0.897512 | 4.01E-05 |
| 6715 | Emcn | 0.896912 | 8.45E-05 |
| 30139 | Tmem65 | 0.895399 | 1.03E-05 |
| 18145 | Kcnn3 | 0.894965 | 0.003575 |
| 26266 | Rhof | 0.894718 | 0.006045 |
| 28002 | Slc37a4 | 0.893429 | 0.002712 |
| 6547 | Efna1 | 0.892427 | 0.000819 |
| 2315 | Anxa3 | 0.89204 | 0.000404 |
| 30371 | Tpst1 | 0.891529 | 6.75E-06 |
| 21997 | Nfkb2 | 0.890795 | 0.000421 |
| 14921 | Gm6169 | 0.89049 | 0.004363 |
| 7698 | Fth1 | 0.890422 | 0.000562 |
| 16604 | H2-T22 | 0.889239 | 0.000691 |
| 18589 | Lactb | 0.889086 | 0.000168 |
| 7691 | Fstl1 | 0.888511 | 8.62E-06 |
| 3789 | Cbx6 | 0.888204 | 0.003012 |
| 7737 | Fxyd1 | 0.887009 | 0.000389 |
| 29016 | Stk17b | 0.886857 | 0.000384 |
| 2569 | Arl4c | 0.886396 | 0.001591 |
| 1654 | Acot10 | 0.88588 | 0.000511 |
| 7755 | Fzd5 | 0.88518 | 0.004852 |
| 30915 | Tst | 0.884326 | 0.003621 |
| 17982 | Jak3 | 0.884174 | 0.0034 |
| 23939 | Ostm1 | 0.883178 | 0.00099 |
| 29338 | Tango2 | 0.882345 | 0.001587 |
| 19628 | Meis3 | 0.881352 | 0.000315 |
| 24284 | Pcsk6 | 0.880346 | 0.000247 |
| 18008 | Jph4 | 0.880162 | 0.001584 |
| 5151 | Crispld2 | 0.880049 | 0.000505 |
| 27473 | Serpinb6a | 0.878871 | 0.00013 |
| 18809 | Lipa | 0.878624 | 1.85E-05 |
| 3613 | Calhm2 | 0.878548 | 0.005163 |
| 25676 | Ptpn3 | 0.878062 | 0.000513 |
| 4587 | Chst1 | 0.877195 | 0.000354 |
| 6817 | Ephb6 | 0.876921 | 9.25E-05 |
| 25861 | Rabac1 | 0.875736 | 0.004381 |
| 3006 | Baalc | 0.875392 | 0.001903 |
| 29408 | Tax1bp3 | 0.874308 | 0.001026 |
| 7973 | Gdpd5 | 0.872993 | 0.004065 |
| 16882 | Hic1 | 0.87283 | 0.000433 |
| 5565 | Cyth1 | 0.871702 | 5.79E-05 |
| 16251 | Gpr135 | 0.871023 | 0.005934 |
| 19701 | Mfsd12 | 0.870885 | 0.00049 |
| 17668 | Il1rap | 0.870558 | 0.000101 |
| 25422 | Prps2 | 0.870433 | 0.000248 |
| 28138 | Slc9b2 | 0.869695 | 0.005485 |
| 19432 | Marchf2 | 0.866344 | 0.00031 |
| 19145 | Lsp1 | 0.865952 | 0.000211 |
| 4268 | Cdkn2d | 0.86496 | 0.000916 |
| 7235 | Fancc | 0.862975 | 0.000668 |
| 30881 | Tspan18 | 0.862574 | 0.000212 |
| 28988 | Stat5a | 0.862469 | 0.002755 |
| 30876 | Tspan12 | 0.861614 | 0.00199 |
| 32105 | Wdfy1 | 0.861234 | 2.89E-05 |
| 19404 | Mapk11 | 0.860192 | 0.00155 |
| 31246 | Ulbp1 | 0.859863 | 0.000898 |
| 28344 | Snhg18 | 0.859766 | 4.20E-05 |
| 28345 | Snhg20 | 0.85915 | 0.005343 |
| 7854 | Gamt | 0.858547 | 0.00528 |
| 23965 | Otulinl | 0.858365 | 0.002005 |
| 26492 | Rnft1 | 0.858299 | 0.000134 |
| 7500 | Fkbp10 | 0.857936 | 4.36E-05 |
| 6692 | Elovl1 | 0.857815 | 0.000602 |
| 25800 | Rab13 | 0.857341 | 8.73E-05 |
| 24351 | Pdia5 | 0.857291 | 0.00024 |
| 30056 | Tmem229b | 0.854691 | 0.000836 |
| 30121 | Tmem47 | 0.854291 | 0.000277 |
| 21878 | Ndufb1 | 0.85366 | 0.001093 |
| 17883 | Isca1 | 0.853434 | 0.000561 |
| 22067 | Nkd2 | 0.853249 | 8.75E-05 |
| 19616 | Megf6 | 0.853197 | 0.000936 |
| 18024 | Kank3 | 0.852587 | 0.000474 |
| 2728 | Atf5 | 0.851177 | 0.000454 |
| 18730 | Leprot | 0.85112 | 4.04E-05 |
| 13395 | Gm2a | 0.849498 | 0.000158 |
| 1513 | Abca1 | 0.847921 | 0.001033 |
| 25842 | Rab40b | 0.84734 | 0.005613 |
| 24508 | Pheta2 | 0.84731 | 0.000436 |
| 24895 | Pmp22 | 0.847192 | 0.000177 |
| 25923 | Ramp2 | 0.846855 | 0.000541 |
| 24047 | Pak1 | 0.845737 | 6.08E-05 |
| 26495 | Rnh1 | 0.845623 | 0.001371 |
| 5399 | Cyb561a3 | 0.844886 | 0.002865 |
| 4661 | Clba1 | 0.844839 | 0.004639 |
| 4049 | Cd151 | 0.844308 | 0.002002 |
| 28801 | Spryd3 | 0.843501 | 0.001871 |
| 2522 | Arhgef15 | 0.842448 | 0.000825 |
| 18074 | Kcnab3 | 0.842284 | 0.00536 |
| 21945 | Nell1 | 0.841037 | 0.006268 |
| 6470 | Ech1 | 0.840255 | 0.000361 |
| 29986 | Tmem158 | 0.839369 | 0.00715 |
| 2314 | Anxa2 | 0.83921 | 0.000319 |
| 24768 | Plcb4 | 0.839033 | 7.75E-05 |
| 28130 | Slc9a3r2 | 0.838157 | 2.98E-05 |
| 30380 | Tradd | 0.837173 | 0.0035 |
| 24393 | Pea15a | 0.836733 | 0.000222 |
| 32454 | Zdhhc1 | 0.835629 | 0.003563 |
| 18309 | Klf9 | 0.835063 | 0.001179 |
| 6984 | Exoc3l | 0.833372 | 0.003049 |
| 2539 | Arhgef6 | 0.831501 | 0.001262 |
| 21337 | Msra | 0.830409 | 0.001744 |
| 6116 | Dnajb1 | 0.829852 | 0.00205 |
| 7099 | Fam131a | 0.829743 | 0.001188 |
| 32224 | Wnt5a | 0.829109 | 0.004552 |
| 2594 | Armcx3 | 0.82881 | 0.000296 |
| 16239 | Gpm6b | 0.828385 | 0.000301 |
| 21729 | Nalcn | 0.828359 | 0.001373 |
| 19275 | Mafk | 0.828247 | 0.003785 |
| 29489 | Tceal3 | 0.82809 | 0.002121 |
| 31251 | Umad1 | 0.827384 | 0.002358 |
| 5262 | Cstb | 0.827345 | 0.000373 |
| 26233 | Rgs3 | 0.8269 | 0.000809 |
| 18294 | Klf10 | 0.826252 | 0.0002 |
| 21786 | Ncam1 | 0.825897 | 0.000151 |
| 24078 | Papln | 0.82573 | 0.003028 |
| 30701 | Trim21 | 0.82325 | 0.002671 |
| 24809 | Plekhg3 | 0.82229 | 0.003669 |
| 7511 | Fkbp7 | 0.821724 | 0.000247 |
| 16706 | H6pd | 0.821665 | 0.000429 |
| 5016 | Cotl1 | 0.821639 | 0.001075 |
| 28986 | Stat3 | 0.81911 | 0.00013 |
| 2687 | Asl | 0.818218 | 0.000602 |
| 14756 | Gm5700 | 0.817405 | 0.002519 |
| 25661 | Ptp4a3 | 0.816014 | 0.00147 |
| 1808 | Adarb1 | 0.815774 | 0.003354 |
| 4874 | Cnrip1 | 0.815721 | 0.000719 |
| 31413 | Vamp8 | 0.815637 | 0.000647 |
| 30801 | Trp53i11 | 0.815391 | 0.003336 |
| 24880 | Plxnc1 | 0.815183 | 0.000554 |
| 30232 | Tnfrsf1a | 0.814909 | 0.000285 |
| 4943 | Col5a1 | 0.814415 | 0.000224 |
| 31204 | Ucp2 | 0.813308 | 0.005366 |
| 4926 | Col1a2 | 0.813194 | 3.27E-05 |
| 6845 | Erap1 | 0.811989 | 0.000831 |
| 5804 | Decr1 | 0.811704 | 0.000304 |
| 30877 | Tspan13 | 0.811686 | 0.00016 |
| 8373 | Gm11451 | 0.811214 | 0.002502 |
| 18597 | Lama1 | 0.810878 | 0.000661 |
| 16857 | Hexa | 0.80909 | 8.07E-05 |
| 18715 | Lef1 | 0.805816 | 0.000172 |
| 17954 | Itpr2 | 0.805158 | 0.001158 |
| 28804 | Spsb1 | 0.804885 | 0.005844 |
| 6378 | Dynlt3 | 0.804168 | 0.000649 |
| 30168 | Tmem9b | 0.80356 | 0.000369 |
| 30084 | Tmem256 | 0.80349 | 0.000882 |
| 27430 | Serinc5 | 0.802429 | 0.000518 |
| 29711 | Tgfbr2 | 0.802017 | 2.11E-05 |
| 5188 | Crybg3 | 0.801933 | 0.000602 |
| 6950 | Ets1 | 0.801727 | 0.000549 |
| 25621 | Pter | 0.801108 | 0.001257 |
| 27383 | Sema4d | 0.800456 | 0.003731 |
| 28578 | Sox13 | 0.800045 | 0.003124 |
| 21540 | Myl12a | 0.799426 | 0.000601 |
| 16227 | Gpd1l | 0.799092 | 0.000659 |
| 6549 | Efna3 | 0.798787 | 0.001588 |
| 24847 | Plpp1 | 0.798482 | 0.00226 |
| 4870 | Cnpy3 | 0.796482 | 0.000148 |
| 32238 | Wsb2 | 0.794194 | 0.003519 |
| 2784 | Atp1b3 | 0.793059 | 0.000121 |
| 5238 | Cspg4 | 0.793032 | 0.006632 |
| 17622 | Ikbip | 0.792157 | 0.00031 |
| 5659 | Dap | 0.790912 | 0.004465 |
| 24622 | Pik3ip1 | 0.789778 | 0.002205 |
| 6985 | Exoc3l2 | 0.789584 | 0.005968 |
| 4099 | Cd302 | 0.789326 | 0.000573 |
| 2352 | Ap3m2 | 0.789217 | 0.00169 |
| 4997 | Coq10b | 0.788348 | 0.005215 |
| 7481 | Fhod3 | 0.787607 | 0.002737 |
| 25022 | Porcn | 0.786537 | 0.002774 |
| 2319 | Anxa7 | 0.786449 | 0.000972 |
| 19258 | Macrod2 | 0.786103 | 0.001524 |
| 1688 | Acsl5 | 0.785493 | 0.000669 |
| 17294 | Ifngr2 | 0.785199 | 0.000367 |
| 24121 | Parvb | 0.783437 | 0.000317 |
| 25550 | Psmb10 | 0.782813 | 0.004009 |
| 24946 | Poglut3 | 0.782759 | 0.000672 |
| 18583 | L3hypdh | 0.78191 | 0.002107 |
| 18038 | Kat2b | 0.781126 | 0.001958 |
| 28509 | Snx10 | 0.780521 | 0.003281 |
| 31340 | Usp2 | 0.780237 | 0.004835 |
| 7669 | Frrs1 | 0.779999 | 0.005802 |
| 7315 | Fbxo27 | 0.778985 | 0.004357 |
| 2389 | Aplp2 | 0.777837 | 0.000184 |
| 19761 | Mid1ip1 | 0.77783 | 0.003263 |
| 25143 | Ppp1r3b | 0.777607 | 0.000333 |
| 26885 | Rps6ka5 | 0.776934 | 0.001371 |
| 18018 | Jund | 0.77686 | 0.005392 |
| 21840 | Ndfip1 | 0.77686 | 9.58E-05 |
| 7997 | Gfra2 | 0.775897 | 0.000399 |
| 21567 | Myo1c | 0.774642 | 0.000426 |
| 29743 | Thbs1 | 0.77407 | 0.000575 |
| 21132 | Mpp1 | 0.773766 | 0.000598 |
| 1800 | Adamtsl1 | 0.773102 | 0.00086 |
| 22066 | Nkd1 | 0.77016 | 0.003202 |
| 27669 | Siae | 0.769576 | 0.001442 |
| 18826 | Litaf | 0.769388 | 0.004859 |
| 7109 | Fam149a | 0.769294 | 0.000363 |
| 7276 | Fbln2 | 0.768866 | 0.001182 |
| 28029 | Slc3a2 | 0.768668 | 0.000989 |
| 2612 | Arpc5 | 0.768511 | 0.000118 |
| 21495 | Mxra7 | 0.767647 | 0.001736 |
| 21975 | Nexn | 0.767171 | 0.003711 |
| 2722 | Ate1 | 0.766997 | 0.00203 |
| 15943 | Gm9294 | 0.766371 | 0.002047 |
| 4562 | Chrd | 0.766154 | 0.003401 |
| 23982 | Oxr1 | 0.765898 | 3.70E-05 |
| 4821 | Cmtm7 | 0.764692 | 0.004745 |
| 28904 | Sspn | 0.763516 | 0.001283 |
| 25840 | Rab3il1 | 0.762892 | 0.000695 |
| 25320 | Prkch | 0.761956 | 0.001073 |
| 18624 | Laptm4a | 0.761901 | 0.000478 |
| 4831 | Cndp2 | 0.760853 | 0.000583 |
| 28051 | Slc48a1 | 0.760638 | 0.000586 |
| 17334 | Igfbp5 | 0.758915 | 0.00316 |
| 26133 | Reep5 | 0.758174 | 0.000837 |
| 5280 | Ctbs | 0.757725 | 0.005152 |
| 50 | 1600014C10Rik | 0.757499 | 0.005603 |
| 18600 | Lama4 | 0.756787 | 0.000312 |
| 16114 | Gmppb | 0.75615 | 0.004999 |
| 27644 | Shisa5 | 0.755731 | 0.001618 |
| 27166 | Scara5 | 0.755653 | 0.006575 |
| 16140 | Gnb5 | 0.754143 | 0.001042 |
| 5706 | Dcc | 0.754131 | 0.003634 |
| 24003 | P3h1 | 0.753379 | 0.000309 |
| 19724 | Mgat1 | 0.752476 | 0.001601 |
| 1947 | Agpat2 | 0.752303 | 0.002546 |
| 7715 | Fuca1 | 0.751854 | 0.000765 |
| 25692 | Ptprm | 0.750279 | 0.000195 |
| 14147 | Gm44126 | 0.749103 | 0.001773 |
| 26343 | Rin3 | 0.748052 | 0.0052 |
| 6678 | Ell2 | 0.747645 | 0.006453 |
| 22581 | Olfm1 | 0.74762 | 0.006117 |
| 15426 | Gm7776 | 0.746939 | 0.001198 |
| 25325 | Prkd1 | 0.746939 | 0.000908 |
| 3250 | Blvrb | 0.746804 | 0.003416 |
| 31409 | Vamp2 | 0.746181 | 0.001163 |
| 27158 | Scamp2 | 0.74458 | 0.003076 |
| 6724 | Eml1 | 0.742322 | 0.001777 |
| 21950 | Nenf | 0.74184 | 0.000532 |
| 26099 | Rcn3 | 0.739017 | 0.0006 |
| 18977 | Loxl3 | 0.738876 | 0.000742 |
| 22041 | Ninj1 | 0.737008 | 0.002168 |
| 21993 | Nfil3 | 0.736445 | 0.005145 |
| 32253 | Xbp1 | 0.73384 | 0.002396 |
| 25312 | Prkar1b | 0.733536 | 0.003258 |
| 7887 | Gas7 | 0.732953 | 0.003506 |
| 2452 | Arap2 | 0.732451 | 0.005384 |
| 2605 | Arntl | 0.732047 | 0.004139 |
| 24006 | P3h4 | 0.731097 | 0.001814 |
| 19113 | Lrrk1 | 0.730772 | 0.005489 |
| 5197 | Cryl1 | 0.73047 | 0.007064 |
| 30081 | Tmem254 | 0.72899 | 0.00661 |
| 8099 | Glipr2 | 0.727993 | 0.001448 |
| 27602 | Sh3bgrl3 | 0.727548 | 0.003502 |
| 3924 | Ccdc82 | 0.727437 | 0.004499 |
| 16253 | Gpr137b | 0.726358 | 0.002579 |
| 4944 | Col5a2 | 0.724846 | 0.00253 |
| 21151 | Mr1 | 0.724244 | 0.000773 |
| 6486 | Eda | 0.723987 | 0.00346 |
| 25943 | Rap2a | 0.723563 | 0.002984 |
| 2861 | Atraid | 0.723399 | 0.001382 |
| 4882 | Cntn3 | 0.722382 | 0.001419 |
| 25703 | Ptprz1 | 0.722101 | 0.001208 |
| 4060 | Cd200 | 0.720258 | 0.002307 |
| 15446 | Gm7823 | 0.718679 | 0.007191 |
| 27493 | Serpinf1 | 0.718205 | 0.0006 |
| 1606 | Acaa2 | 0.717829 | 0.003986 |
| 16124 | Gnai2 | 0.717479 | 0.001291 |
| 24352 | Pdia6 | 0.717351 | 0.000966 |
| 2826 | Atp6v0d1 | 0.716892 | 0.0047 |
| 24892 | Pmm1 | 0.715477 | 0.002205 |
| 4178 | Cdc42se1 | 0.714496 | 0.002283 |
| 6774 | Entpd5 | 0.714482 | 0.000861 |
| 2625 | Arsa | 0.714342 | 0.001888 |
| 22253 | Nqo2 | 0.713177 | 0.001903 |
| 22273 | Nr3c1 | 0.712611 | 0.001512 |
| 27822 | Slc1a3 | 0.712482 | 0.001445 |
| 2429 | App | 0.712288 | 0.000544 |
| 29840 | Tlcd2 | 0.711368 | 0.006126 |
| 17217 | Id3 | 0.710417 | 0.003487 |
| 16341 | Gpx4 | 0.710059 | 0.006183 |
| 7536 | Flt4 | 0.707825 | 0.002917 |
| 31053 | Txndc5 | 0.707576 | 0.000489 |
| 4634 | Cisd1 | 0.706749 | 0.006959 |
| 5203 | Csad | 0.70625 | 0.004344 |
| 3629 | Calu | 0.705949 | 0.000395 |
| 24370 | Pdrg1 | 0.705919 | 0.004234 |
| 4753 | Clic1 | 0.703691 | 0.001757 |
| 3141 | Bcl10 | 0.703005 | 0.00127 |
| 8009 | Ggh | 0.702802 | 0.002107 |
| 28535 | Snx7 | 0.701636 | 0.002973 |
| 8504 | Gm11964 | 0.700437 | 0.002669 |
| 32041 | Vps8 | 0.70035 | 0.001914 |
| 19783 | Mindy3 | 0.69838 | 0.002909 |
| 29134 | Sumf1 | 0.697459 | 0.000625 |
| 17990 | Jazf1 | 0.69621 | 0.003754 |
| 24143 | Pawr | 0.69531 | 0.005607 |
| 27637 | She | 0.695141 | 0.006794 |
| 29939 | Tmem109 | 0.694825 | 0.004381 |
| 32205 | Wipi1 | 0.693703 | 0.001238 |
| 15353 | Gm7588 | 0.692431 | 0.001097 |
| 17961 | Itpripl2 | 0.691881 | 0.002996 |
| 27572 | Sgms1 | 0.691744 | 0.001547 |
| 3636 | Camk2d | 0.690763 | 0.002244 |
| 8092 | Gli1 | 0.69037 | 0.004735 |
| 24212 | Pcdhb17 | 0.690355 | 0.004264 |
| 22403 | Nudt4 | 0.689945 | 0.000271 |
| 25133 | Ppp1r2 | 0.68974 | 0.001856 |
| 2087 | Aldh3a2 | 0.6889 | 0.003978 |
| 15571 | Gm8185 | 0.688175 | 0.002011 |
| 21922 | Nedd9 | 0.68748 | 0.001182 |
| 3070 | BC004004 | 0.687414 | 0.004367 |
| 26229 | Rgs2 | 0.68682 | 0.00621 |
| 7513 | Fkbp9 | 0.68552 | 0.000349 |
| 31040 | Twsg1 | 0.684645 | 0.00029 |
| 24790 | Plec | 0.683659 | 0.006885 |
| 6253 | Dpysl3 | 0.680193 | 0.001204 |
| 22472 | Oaf | 0.68002 | 0.002719 |
| 2610 | Arpc3 | 0.679301 | 0.002289 |
| 6494 | Edem2 | 0.678607 | 0.005205 |
| 28664 | Spata6 | 0.677506 | 0.002399 |
| 2626 | Arsb | 0.675842 | 0.00085 |
| 25587 | Psme2b | 0.675813 | 0.004086 |
| 5326 | Ctsf | 0.675208 | 0.002227 |
| 22379 | Nucb2 | 0.674965 | 0.00506 |
| 19156 | Ltbp3 | 0.674407 | 0.00536 |
| 2212 | Angptl2 | 0.67413 | 0.002128 |
| 30216 | Tnfaip3 | 0.673893 | 0.002378 |
| 16144 | Gng12 | 0.673732 | 0.000387 |
| 29430 | Tbc1d2b | 0.672236 | 0.001484 |
| 27985 | Slc35f2 | 0.67177 | 0.004901 |
| 30101 | Tmem30a | 0.665823 | 0.003789 |
| 19353 | Maoa | 0.664842 | 0.002575 |
| 6205 | Dok4 | 0.662743 | 0.006879 |
| 17907 | Itga11 | 0.661515 | 0.00654 |
| 29082 | Stx4a | 0.661026 | 0.001665 |
| 5071 | Cpm | 0.660656 | 0.004433 |
| 27588 | Sh2b3 | 0.659989 | 0.005462 |
| 2390 | Apmap | 0.659808 | 0.003165 |
| 29524 | Tcn2 | 0.659302 | 0.007148 |
| 26124 | Reck | 0.657248 | 0.001727 |
| 22308 | Nrp1 | 0.657042 | 0.004031 |
| 28589 | Sox4 | 0.656943 | 0.000427 |
| 4173 | Cdc42ep1 | 0.656696 | 0.006249 |
| 24942 | Pofut2 | 0.655611 | 0.002377 |
| 4646 | Ckap4 | 0.651756 | 0.001331 |
| 18333 | Klhl23 | 0.65175 | 0.003195 |
| 29459 | Tbrg1 | 0.648992 | 0.005769 |
| 2803 | Atp5e | 0.647005 | 0.004589 |
| 8041 | Ginm1 | 0.646765 | 0.002551 |
| 7725 | Fut11 | 0.646465 | 0.002001 |
| 1836 | Adgrb2 | 0.645939 | 0.004269 |
| 18605 | Lamc1 | 0.645358 | 0.00124 |
| 6883 | Erlec1 | 0.645156 | 0.001306 |
| 25104 | Ppm1m | 0.644924 | 0.005115 |
| 3922 | Ccdc80 | 0.643962 | 0.004578 |
| 22241 | Nptx1 | 0.643645 | 0.002322 |
| 18303 | Klf3 | 0.641713 | 0.002638 |
| 7409 | Fermt2 | 0.640957 | 0.00074 |
| 26264 | Rhoc | 0.639379 | 0.002027 |
| 27562 | Sgcb | 0.637028 | 0.002798 |
| 22234 | Npr2 | 0.636807 | 0.006361 |
| 18609 | Lamp2 | 0.636323 | 0.002066 |
| 2707 | Astn2 | 0.635402 | 0.003475 |
| 5747 | Ddah1 | 0.635367 | 0.002079 |
| 7779 | Gab2 | 0.633576 | 0.006782 |
| 28018 | Slc39a13 | 0.632882 | 0.006403 |
| 28675 | Spcs1 | 0.631048 | 0.003538 |
| 1496 | Aagab | 0.630225 | 0.006445 |
| 31297 | Uqcr10 | 0.628559 | 0.006407 |
| 25778 | Qsox1 | 0.628116 | 0.006074 |
| 24056 | Pald1 | 0.625879 | 0.004124 |
| 25709 | Pttg1ip | 0.624534 | 0.003632 |
| 19229 | Lysmd2 | 0.623876 | 0.005836 |
| 5925 | Desi2 | 0.622924 | 0.007213 |
| 4929 | Col23a1 | 0.622312 | 0.002852 |
| 2019 | Ak3 | 0.621717 | 0.001815 |
| 21569 | Myo1e | 0.621666 | 0.002412 |
| 30004 | Tmem176a | 0.621098 | 0.006538 |
| 5890 | Degs1 | 0.620495 | 0.002301 |
| 22286 | Nrbp1 | 0.619547 | 0.005028 |
| 30764 | Trip10 | 0.616499 | 0.005178 |
| 6712 | Emc7 | 0.615934 | 0.005027 |
| 29085 | Stx7 | 0.615284 | 0.003717 |
| 25456 | Prrg3 | 0.614296 | 0.004526 |
| 7486 | Fig4 | 0.613523 | 0.005761 |
| 4138 | Cd81 | 0.613484 | 0.002905 |
| 2538 | Arhgef5 | 0.612663 | 0.007072 |
| 29896 | Tmbim6 | 0.611444 | 0.006564 |
| 3938 | Ccdc91 | 0.611423 | 0.005382 |
| 2461 | Arf4 | 0.611113 | 0.001184 |
| 28825 | Sqstm1 | 0.609284 | 0.005205 |
| 6342 | Dusp6 | 0.609082 | 0.004705 |
| 17607 | Igsf10 | 0.608571 | 0.004331 |
| 30890 | Tspan6 | 0.604552 | 0.00321 |
| 3413 | Btd | 0.596376 | 0.004909 |
| 18608 | Lamp1 | 0.596205 | 0.002587 |
| 19755 | Micos10 | 0.591846 | 0.005549 |
| 21340 | Msrb3 | 0.591213 | 0.003046 |
| 29927 | Tmed9 | 0.591002 | 0.004624 |
| 29727 | Tgoln1 | 0.590749 | 0.005279 |
| 24008 | P4ha1 | 0.589514 | 0.003202 |
| 3227 | Bin3 | 0.588702 | 0.006988 |
| 19725 | Mgat2 | 0.575975 | 0.007121 |
| 30122 | Tmem50a | 0.568596 | 0.006881 |
| 24456 | Pfn2 | 0.56847 | 0.003834 |
| 19248 | M6pr | 0.566886 | 0.006101 |
| 19269 | Maf | 0.562922 | 0.003183 |
| 31125 | Ube2j1 | 0.561894 | 0.005792 |
| 5402 | Cyb5a | 0.556232 | 0.004505 |
| 3344 | Brip1os | 0.556124 | 0.006076 |
| 17931 | Itgb5 | 0.554547 | 0.004186 |
| 31079 | Uaca | 0.546304 | 0.005095 |
| 7492 | Filip1l | 0.541152 | 0.006358 |
| 9710 | Gm17936 | 0.539217 | 0.007 |
| 5331 | Ctsl | 0.534359 | 0.005514 |
| 17980 | Jak1 | 0.517487 | 0.006927 |

**Table3. Upregulated DEGs of lingual part of tooth germs at cap stage than of buccal part**

|  | SYMBOL | log2FoldChange | pvalue |
| --- | --- | --- | --- |
| 30892 | Tspan8 | -5.50196 | 1.44E-131 |
| 22279 | Nr5a2 | -5.34191 | 7.43E-306 |
| 25263 | Prdm16os | -4.78483 | 7.72E-16 |
| 27030 | Rxfp2 | -4.67111 | 7.17E-133 |
| 2878 | AU015836 | -4.48231 | 7.24E-12 |
| 4621 | Cidea | -4.22785 | 5.97E-06 |
| 10184 | Gm18981 | -4.15232 | 9.46E-37 |
| 16725 | Hand2 | -4.13481 | 4.69E-48 |
| 16520 | Gucy2f | -4.05888 | 7.02E-15 |
| 16810 | Hdnr | -4.03523 | 1.85E-13 |
| 29166 | Sv2b | -3.99309 | 1.00E-21 |
| 30861 | Tshr | -3.94444 | 1.83E-23 |
| 23940 | Ostn | -3.90864 | 6.83E-31 |
| 2772 | Atp12a | -3.88266 | 2.67E-06 |
| 16726 | Hand2os1 | -3.83857 | 2.04E-24 |
| 22084 | Nkx3-1 | -3.83575 | 2.06E-08 |
| 16289 | Gpr25 | -3.7896 | 4.30E-07 |
| 679 | 4930426D05Rik | -3.75698 | 7.06E-06 |
| 25262 | Prdm16 | -3.69584 | 5.71E-136 |
| 5256 | Cst8 | -3.6715 | 0.000273 |
| 21764 | Nat8f3 | -3.64627 | 1.71E-06 |
| 27642 | Shisa3 | -3.6294 | 3.85E-25 |
| 7402 | Fendrr | -3.55103 | 0 |
| 19176 | Ly6a | -3.54435 | 8.69E-22 |
| 16942 | Hmgcs2 | -3.50411 | 7.85E-102 |
| 1824 | Adcy8 | -3.49173 | 2.75E-171 |
| 6740 | Enam | -3.45924 | 6.89E-05 |
| 25697 | Ptprr | -3.3658 | 4.52E-38 |
| 25803 | Rab17 | -3.34936 | 2.48E-12 |
| 7601 | Foxf1 | -3.32004 | 0 |
| 4218 | Cdhr1 | -3.31512 | 2.34E-70 |
| 30290 | Tnxb | -3.27476 | 5.77E-40 |
| 28263 | Smim32 | -3.24694 | 1.07E-06 |
| 5102 | Cpxm2 | -3.23959 | 1.20E-18 |
| 19779 | Minar1 | -3.23234 | 2.21E-18 |
| 7613 | Foxl1 | -3.2192 | 4.49E-05 |
| 13958 | Gm38684 | -3.17952 | 0.000304 |
| 28101 | Slc6a5 | -3.17085 | 2.85E-06 |
| 18325 | Klhl14 | -3.15237 | 0.000363 |
| 19776 | Mill1 | -3.1446 | 7.79E-07 |
| 17088 | Hsd11b1 | -3.13329 | 6.35E-09 |
| 5348 | Ctxnd1 | -3.1292 | 6.42E-151 |
| 7116 | Fam162b | -3.11683 | 9.31E-09 |
| 6108 | Dnai1 | -3.11507 | 7.14E-13 |
| 22449 | Nwd2 | -3.10487 | 2.45E-19 |
| 5438 | Cyp26a1 | -3.09482 | 8.07E-30 |
| 3773 | Cbln1 | -3.06087 | 2.18E-87 |
| 7441 | Fgf20 | -3.04468 | 1.09E-23 |
| 24498 | Phactr3 | -3.02167 | 2.58E-30 |
| 7055 | Fabp4 | -3.01631 | 4.98E-07 |
| 17161 | Htr2c | -3.01621 | 0.000203 |
| 27951 | Slc30a2 | -3.0145 | 3.99E-10 |
| 570 | 3100003L05Rik | -3.00724 | 0.000859 |
| 17168 | Htr7 | -3.007 | 4.69E-59 |
| 29160 | Susd5 | -3.00253 | 1.75E-15 |
| 1648 | Acnat1 | -2.9789 | 0.004126 |
| 28942 | St6gal2 | -2.96964 | 1.56E-172 |
| 6863 | Ereg | -2.96778 | 0.004801 |
| 1256 | 9130024F11Rik | -2.96324 | 6.23E-124 |
| 28336 | Sned1 | -2.94597 | 1.23E-222 |
| 24083 | Pappa2 | -2.93589 | 0 |
| 16290 | Gpr26 | -2.93468 | 0.004844 |
| 16261 | Gpr149 | -2.90382 | 3.14E-05 |
| 22254 | Nr0b1 | -2.87549 | 0.006077 |
| 26530 | Rorb | -2.85974 | 2.34E-37 |
| 25648 | Pth2r | -2.84932 | 2.08E-20 |
| 2152 | Ambn | -2.84741 | 2.25E-137 |
| 1363 | A2ml1 | -2.84054 | 3.41E-06 |
| 22120 | Nmbr | -2.82632 | 1.11E-10 |
| 3265 | Bmp8a | -2.80612 | 5.23E-06 |
| 7809 | Gad1os | -2.8029 | 1.55E-06 |
| 21040 | Mmp13 | -2.80261 | 2.02E-11 |
| 1447 | A930001A20Rik | -2.80002 | 0.00269 |
| 2675 | Ascl5 | -2.77198 | 5.01E-27 |
| 17079 | Hs3st4 | -2.7674 | 8.23E-08 |
| 25159 | Ppp2r2b | -2.75973 | 1.51E-64 |
| 26983 | Rtl3 | -2.73567 | 2.52E-291 |
| 3341 | Brinp2 | -2.72574 | 2.96E-10 |
| 4198 | Cdh12 | -2.71556 | 3.05E-58 |
| 30184 | Tmprss11f | -2.71364 | 0.000153 |
| 18747 | Lgi1 | -2.69845 | 3.62E-17 |
| 21702 | Naaa | -2.68465 | 1.79E-155 |
| 30458 | Trank1 | -2.66618 | 1.76E-34 |
| 6737 | En1 | -2.66526 | 3.56E-82 |
| 2132 | Alox12e | -2.65109 | 2.56E-20 |
| 7199 | Fam43a | -2.63932 | 1.89E-148 |
| 30182 | Tmprss11d | -2.63722 | 3.10E-08 |
| 7690 | Fst | -2.63602 | 9.85E-223 |
| 16304 | Gpr50 | -2.6335 | 1.54E-26 |
| 26152 | Ren1 | -2.62586 | 3.65E-06 |
| 17878 | Irx2 | -2.61741 | 3.73E-69 |
| 28605 | Sp5 | -2.61459 | 1.39E-29 |
| 27166 | Scara5 | -2.59284 | 5.12E-163 |
| 6268 | Drd1 | -2.58901 | 9.79E-07 |
| 32649 | Zfp488 | -2.57437 | 2.32E-17 |
| 15609 | Gm8285 | -2.56944 | 0.006686 |
| 104 | 1700010K23Rik | -2.56875 | 0.008354 |
| 18597 | Lama1 | -2.55812 | 1.29E-213 |
| 1759 | Adam23 | -2.55561 | 7.30E-189 |
| 7607 | Foxi3 | -2.55053 | 4.42E-23 |
| 6760 | Enpp1 | -2.54261 | 5.35E-283 |
| 4324 | Celrr | -2.5304 | 3.65E-06 |
| 4887 | Cntnap2 | -2.50251 | 1.13E-40 |
| 19269 | Maf | -2.49122 | 7.67E-251 |
| 5440 | Cyp26c1 | -2.47343 | 3.21E-10 |
| 23999 | P2ry14 | -2.47061 | 1.14E-08 |
| 2849 | Atp8b1 | -2.45374 | 3.00E-57 |
| 3588 | Cacng5 | -2.44692 | 3.31E-08 |
| 4953 | Col8a1 | -2.44086 | 0 |
| 16827 | Hecw1 | -2.41588 | 4.89E-16 |
| 27132 | Satb2 | -2.41588 | 0 |
| 17890 | Isl2 | -2.41171 | 0.000208 |
| 2295 | Ano3 | -2.39517 | 5.32E-10 |
| 30007 | Tmem178 | -2.39416 | 1.48E-11 |
| 7594 | Foxd1 | -2.39414 | 6.11E-16 |
| 24919 | Pnoc | -2.37741 | 6.87E-07 |
| 17877 | Irx1 | -2.37336 | 1.48E-76 |
| 4233 | Cdk15 | -2.35926 | 7.94E-07 |
| 4730 | Clec2g | -2.34946 | 3.61E-06 |
| 1547 | Abcc8 | -2.34561 | 4.62E-23 |
| 30894 | Tspear | -2.34515 | 1.57E-15 |
| 5433 | Cyp1b1 | -2.34501 | 7.24E-108 |
| 1792 | Adamts20 | -2.33719 | 1.91E-243 |
| 2146 | Alx1 | -2.33561 | 2.05E-40 |
| 4955 | Col9a1 | -2.32682 | 1.78E-41 |
| 17907 | Itga11 | -2.32148 | 2.75E-127 |
| 4210 | Cdh26 | -2.31074 | 0.001854 |
| 18317 | Klhdc8a | -2.3033 | 0.000292 |
| 27691 | Sim2 | -2.30106 | 3.29E-09 |
| 6812 | Epha8 | -2.29297 | 6.43E-22 |
| 26074 | Rbp4 | -2.29237 | 8.15E-15 |
| 4056 | Cd19 | -2.29119 | 0.004291 |
| 4958 | Colec10 | -2.28886 | 0.000231 |
| 4524 | Chgb | -2.28865 | 5.95E-08 |
| 21358 | Mtarc1 | -2.28014 | 0.022746 |
| 29775 | Thsd7b | -2.27692 | 8.62E-35 |
| 510 | 2610016A17Rik | -2.2724 | 0.020904 |
| 32002 | Vnn1 | -2.27087 | 3.85E-15 |
| 32982 | Zpld1 | -2.27068 | 7.29E-08 |
| 4882 | Cntn3 | -2.26293 | 1.01E-102 |
| 21767 | Nat8f6 | -2.25801 | 0.022941 |
| 6101 | Dnah5 | -2.25047 | 0.001292 |
| 5106 | Crabp1 | -2.24873 | 1.41E-166 |
| 6807 | Epha3 | -2.24778 | 0 |
| 18160 | Kcnv1 | -2.24315 | 2.81E-09 |
| 4591 | Chst13 | -2.24312 | 0.000118 |
| 2292 | Ano1 | -2.24139 | 6.07E-76 |
| 7962 | Gdf5 | -2.24109 | 4.96E-10 |
| 5151 | Crispld2 | -2.2367 | 1.95E-153 |
| 26340 | Rims4 | -2.23499 | 9.40E-11 |
| 17644 | Il15 | -2.23273 | 5.08E-11 |
| 21561 | Myo16 | -2.22667 | 6.05E-36 |
| 6769 | Entpd1 | -2.20806 | 6.88E-196 |
| 8015 | Ggt5 | -2.19479 | 8.07E-24 |
| 29721 | Tgm2 | -2.19448 | 3.42E-164 |
| 4568 | Chrm4 | -2.19025 | 4.48E-08 |
| 27281 | Scube3 | -2.18857 | 1.70E-206 |
| 7808 | Gad1 | -2.18195 | 1.72E-09 |
| 22031 | Niban1 | -2.18195 | 2.34E-187 |
| 32931 | Zim1 | -2.18091 | 5.37E-250 |
| 22198 | Noxa1 | -2.18025 | 0.018035 |
| 25070 | Ppef2 | -2.17933 | 2.22E-06 |
| 7104 | Fam135b | -2.17655 | 4.77E-10 |
| 16299 | Gpr37 | -2.17222 | 2.51E-21 |
| 22016 | Ngfr | -2.16924 | 4.96E-201 |
| 18385 | Klk4 | -2.16069 | 0.000499 |
| 27081 | Sall4 | -2.15339 | 0.000695 |
| 24275 | Pcp4 | -2.15003 | 0.000552 |
| 19742 | Mgst2 | -2.13985 | 0.000156 |
| 5387 | Cxcl5 | -2.13691 | 9.96E-42 |
| 30079 | Tmem252 | -2.13049 | 7.01E-217 |
| 32422 | Zc3h12d | -2.11067 | 1.17E-08 |
| 7435 | Fgf14 | -2.10265 | 5.79E-40 |
| 27446 | Serpina3f | -2.09464 | 1.91E-12 |
| 25988 | Rassf10 | -2.09434 | 6.81E-09 |
| 6066 | Dmrt2 | -2.08517 | 2.78E-16 |
| 7445 | Fgf3 | -2.08411 | 8.25E-26 |
| 5388 | Cxcl9 | -2.07562 | 0.012084 |
| 31265 | Unc5a | -2.07334 | 6.25E-09 |
| 14370 | Gm4841 | -2.06115 | 1.11E-17 |
| 7081 | Fam110c | -2.04732 | 0.000302 |
| 24412 | Penk | -2.04554 | 1.79E-148 |
| 17083 | Hs6st2 | -2.04509 | 1.45E-237 |
| 27056 | S100a9 | -2.0445 | 0.001682 |
| 21508 | Myc | -2.04099 | 2.08E-156 |
| 19504 | Mc5r | -2.03163 | 5.10E-20 |
| 27052 | S100a6 | -2.03067 | 2.22E-72 |
| 28030 | Slc40a1 | -2.02712 | 4.86E-86 |
| 19197 | Ly75 | -2.02102 | 5.39E-68 |
| 17935 | Itgbl1 | -2.01315 | 2.47E-108 |
| 30335 | Tpbgl | -2.01058 | 4.50E-06 |
| 6759 | Enpep | -2.00471 | 4.64E-146 |
| 29729 | Tgtp1 | -1.99778 | 0.000374 |
| 24544 | Phlda2 | -1.99488 | 1.25E-10 |
| 29323 | Tafa4 | -1.9828 | 2.04E-07 |
| 32057 | Vstm2l | -1.97509 | 5.92E-06 |
| 18817 | Lipm | -1.96708 | 0.00271 |
| 28769 | Spp1 | -1.96519 | 7.84E-12 |
| 2148 | Alx4 | -1.96146 | 1.82E-192 |
| 3053 | BB557941 | -1.95914 | 0.007315 |
| 17078 | Hs3st3b1 | -1.94886 | 2.15E-76 |
| 1439 | A830019L24Rik | -1.93714 | 0.000136 |
| 21768 | Nat8f7 | -1.93436 | 0.022421 |
| 7938 | Gckr | -1.93421 | 0.000209 |
| 17202 | Ibsp | -1.93032 | 6.39E-177 |
| 2629 | Arsj | -1.92506 | 1.35E-23 |
| 24487 | Pgm5 | -1.92159 | 4.20E-208 |
| 7841 | Galnt3 | -1.91528 | 4.06E-08 |
| 19675 | Mettl7a3 | -1.91002 | 0.000201 |
| 6061 | Dmkn | -1.90841 | 0.00053 |
| 6489 | Edaradd | -1.90719 | 4.62E-48 |
| 17646 | Il16 | -1.90547 | 1.26E-37 |
| 7547 | Fmo1 | -1.90146 | 8.39E-71 |
| 7371 | Fcgr2b | -1.89842 | 1.24E-15 |
| 24078 | Papln | -1.89181 | 1.22E-61 |
| 18144 | Kcnn2 | -1.89158 | 1.80E-14 |
| 5533 | Cyp4f15 | -1.89054 | 5.63E-05 |
| 16362 | Grem1 | -1.88954 | 6.66E-50 |
| 27015 | Runx1 | -1.87998 | 3.13E-103 |
| 32216 | Wnt10b | -1.87689 | 2.70E-46 |
| 5995 | Dio3os | -1.86923 | 2.05E-12 |
| 6748 | Enho | -1.8692 | 1.07E-38 |
| 15483 | Gm7932 | -1.86855 | 0.017327 |
| 5361 | Cutal | -1.86832 | 0.011553 |
| 28971 | Stap1 | -1.86767 | 1.21E-07 |
| 19569 | Me3 | -1.86126 | 1.02E-33 |
| 28944 | St6galnac2 | -1.85857 | 1.28E-20 |
| 24313 | Pde1a | -1.85836 | 8.00E-73 |
| 27995 | Slc36a2 | -1.85767 | 4.59E-05 |
| 24781 | Plcxd3 | -1.8553 | 0.000905 |
| 30264 | Tnmd | -1.85324 | 4.75E-31 |
| 29731 | Th | -1.85178 | 0.000481 |
| 31306 | Urah | -1.85121 | 0.000206 |
| 6877 | Erich2 | -1.84933 | 1.90E-08 |
| 19751 | Mical2 | -1.84422 | 1.62E-119 |
| 4757 | Clic6 | -1.8408 | 3.41E-25 |
| 29039 | Stmn4 | -1.8337 | 9.77E-05 |
| 19736 | Mgll | -1.83324 | 1.97E-68 |
| 28088 | Slc6a14 | -1.83228 | 0.000391 |
| 21731 | Nalf2 | -1.83197 | 5.52E-22 |
| 30858 | Tsga13 | -1.82869 | 7.29E-08 |
| 29177 | Svopl | -1.82639 | 3.58E-05 |
| 7100 | Fam131b | -1.82468 | 6.80E-34 |
| 3462 | C130050O18Rik | -1.82445 | 1.75E-05 |
| 25061 | Pparg | -1.82305 | 9.23E-05 |
| 26235 | Rgs5 | -1.81565 | 6.70E-110 |
| 24743 | Plagl1 | -1.81537 | 0 |
| 19607 | Medag | -1.81531 | 1.12E-10 |
| 7602 | Foxf2 | -1.81261 | 4.57E-77 |
| 4928 | Col22a1 | -1.81238 | 7.16E-36 |
| 25693 | Ptprn | -1.79514 | 2.25E-17 |
| 18805 | Lingo2 | -1.79331 | 7.39E-24 |
| 6488 | Edar | -1.79255 | 8.69E-23 |
| 28758 | Spock1 | -1.79197 | 2.14E-137 |
| 2161 | Amer2 | -1.78997 | 2.59E-39 |
| 5054 | Cpa6 | -1.78535 | 6.26E-12 |
| 30224 | Tnfrsf11b | -1.78053 | 1.88E-20 |
| 24858 | Plppr5 | -1.77532 | 0.000204 |
| 19627 | Meis2 | -1.7736 | 2.23E-137 |
| 29027 | Stk33 | -1.77076 | 0.001443 |
| 29730 | Tgtp2 | -1.7698 | 0.000671 |
| 1541 | Abcc12 | -1.76886 | 0.000469 |
| 7992 | Gfod1 | -1.76783 | 3.39E-57 |
| 27251 | Scn3a | -1.76483 | 3.02E-45 |
| 18154 | Kcns1 | -1.76314 | 1.30E-11 |
| 17879 | Irx3 | -1.76252 | 1.69E-43 |
| 29634 | Tex11 | -1.76126 | 0.000434 |
| 7463 | Fgl2 | -1.75553 | 2.50E-15 |
| 5993 | Dio2 | -1.755 | 3.17E-15 |
| 2333 | Aox4 | -1.75267 | 1.35E-06 |
| 9629 | Gm17315 | -1.74995 | 8.20E-05 |
| 21059 | Mmp9 | -1.74934 | 3.57E-164 |
| 3493 | C1s1 | -1.74758 | 2.48E-24 |
| 4202 | Cdh17 | -1.74684 | 0.000287 |
| 27523 | Sez6 | -1.74359 | 0.00781 |
| 17080 | Hs3st5 | -1.74235 | 1.27E-20 |
| 27057 | S100b | -1.74188 | 5.17E-12 |
| 7759 | Fzd9 | -1.74061 | 4.01E-21 |
| 6194 | Dock3 | -1.74023 | 1.76E-21 |
| 26990 | Rtl9 | -1.73205 | 5.04E-38 |
| 17786 | Insrr | -1.72607 | 9.97E-21 |
| 6036 | Dlk1 | -1.72588 | 2.04E-113 |
| 30179 | Tmprss11a | -1.72269 | 6.67E-08 |
| 4847 | Cnmd | -1.72206 | 4.24E-13 |
| 5439 | Cyp26b1 | -1.71965 | 1.87E-202 |
| 5647 | Dact1 | -1.71923 | 1.11E-104 |
| 24348 | Pdia2 | -1.718 | 0.000671 |
| 3754 | Catsperg2 | -1.71542 | 0.018189 |
| 24551 | Phospho1 | -1.71538 | 2.30E-08 |
| 27053 | S100a7a | -1.71219 | 0.001618 |
| 29031 | Stk38l | -1.7079 | 1.81E-62 |
| 21033 | Mme | -1.70243 | 7.24E-232 |
| 19664 | Mettl24 | -1.69628 | 6.03E-05 |
| 16175 | Golga7b | -1.69611 | 2.53E-05 |
| 16361 | Greb1l | -1.69464 | 1.82E-78 |
| 19206 | Lynx1 | -1.69221 | 4.13E-23 |
| 24563 | Phyhip | -1.69093 | 0.001019 |
| 4924 | Col19a1 | -1.68682 | 1.01E-06 |
| 5664 | Dapl1 | -1.67988 | 3.32E-09 |
| 3690 | Car2 | -1.67951 | 9.25E-64 |
| 2706 | Astn1 | -1.67845 | 4.20E-109 |
| 27040 | Ryr2 | -1.6781 | 9.22E-20 |
| 28242 | Smim1 | -1.67681 | 4.28E-21 |
| 30957 | Ttc6 | -1.67606 | 7.35E-07 |
| 27262 | Scnn1g | -1.67458 | 0.004045 |
| 27641 | Shisa2 | -1.67246 | 2.72E-85 |
| 17881 | Irx5 | -1.66977 | 2.72E-38 |
| 24000 | P2ry2 | -1.66967 | 2.97E-07 |
| 18298 | Klf14 | -1.66184 | 4.22E-26 |
| 27238 | Scin | -1.65906 | 9.16E-19 |
| 31270 | Unc79 | -1.65131 | 4.86E-11 |
| 25975 | Rasgrf2 | -1.65018 | 1.48E-05 |
| 31365 | Usp44 | -1.64827 | 7.67E-09 |
| 22181 | Nos1 | -1.6473 | 2.50E-39 |
| 25678 | Ptpn5 | -1.64447 | 1.78E-06 |
| 8606 | Gm12371 | -1.64409 | 0.00023 |
| 6811 | Epha7 | -1.64389 | 9.50E-166 |
| 29157 | Susd2 | -1.64265 | 9.40E-21 |
| 16403 | Grm3 | -1.64078 | 7.26E-07 |
| 26967 | Rspo3 | -1.63799 | 5.12E-85 |
| 19174 | Lvrn | -1.63728 | 5.10E-31 |
| 31455 | Vgll3 | -1.63436 | 2.83E-103 |
| 3029 | Bambi | -1.62768 | 6.32E-71 |
| 28042 | Slc45a1 | -1.62551 | 0.001048 |
| 7599 | Foxe1 | -1.61902 | 3.85E-08 |
| 19395 | Map6 | -1.60828 | 6.14E-16 |
| 24870 | Plxdc1 | -1.6079 | 1.65E-10 |
| 26236 | Rgs6 | -1.60622 | 1.64E-14 |
| 27566 | Sgcz | -1.60494 | 0.000493 |
| 28759 | Spock2 | -1.59383 | 4.02E-145 |
| 27524 | Sez6l | -1.58979 | 3.93E-16 |
| 27573 | Sgms2 | -1.58904 | 8.93E-55 |
| 7804 | Gabrq | -1.58524 | 7.17E-05 |
| 7755 | Fzd5 | -1.58513 | 1.59E-32 |
| 4208 | Cdh23 | -1.58257 | 9.58E-10 |
| 27279 | Scube1 | -1.5801 | 9.10E-138 |
| 23914 | Osbp2 | -1.57681 | 1.09E-13 |
| 5557 | Cyria | -1.57681 | 2.20E-53 |
| 22295 | Nrg3 | -1.5749 | 3.02E-06 |
| 1923 | Afp | -1.57323 | 0.013462 |
| 7628 | Foxp2 | -1.57274 | 3.96E-38 |
| 32458 | Zdhhc14 | -1.56732 | 9.99E-24 |
| 3597 | Cadm2 | -1.56701 | 2.23E-43 |
| 26553 | Rph3al | -1.56383 | 3.04E-18 |
| 13789 | Gm35340 | -1.56024 | 0.00263 |
| 30180 | Tmprss11b | -1.55992 | 4.72E-08 |
| 7813 | Gadd45g | -1.55979 | 7.98E-44 |
| 6500 | Edn3 | -1.55718 | 8.40E-52 |
| 28055 | Slc4a11 | -1.55502 | 4.06E-48 |
| 2474 | Arg2 | -1.55413 | 0.000535 |
| 6069 | Dmrta2 | -1.54694 | 3.43E-05 |
| 8969 | Gm13889 | -1.54417 | 7.94E-18 |
| 30212 | Tnc | -1.54077 | 2.21E-184 |
| 18863 | Lmx1b | -1.53965 | 0.00225 |
| 27280 | Scube2 | -1.53909 | 1.22E-12 |
| 25614 | Ptchd1 | -1.5375 | 5.30E-08 |
| 16368 | Gria1 | -1.53696 | 3.10E-89 |
| 16193 | Got1l1 | -1.536 | 1.22E-05 |
| 19189 | Ly6g6e | -1.5346 | 0.001448 |
| 5994 | Dio3 | -1.53215 | 4.49E-23 |
| 5168 | Crtac1 | -1.52848 | 1.37E-49 |
| 3981 | Ccn2 | -1.52582 | 2.01E-65 |
| 18150 | Kcnq3 | -1.52112 | 8.35E-10 |
| 23930 | Osgin1 | -1.5209 | 0.00127 |
| 13572 | Gm32618 | -1.52028 | 4.08E-27 |
| 26086 | Rcan2 | -1.51912 | 3.06E-32 |
| 18500 | Krt8 | -1.51854 | 1.78E-09 |
| 17061 | Hr | -1.51557 | 5.59E-28 |
| 24040 | Pag1 | -1.51199 | 3.19E-82 |
| 32222 | Wnt3a | -1.51042 | 2.77E-07 |
| 7433 | Fgf12 | -1.50816 | 1.15E-24 |
| 4485 | Cgnl1 | -1.50613 | 1.99E-116 |
| 32468 | Zdhhc23 | -1.50595 | 5.22E-18 |
| 25330 | Prkg2 | -1.50457 | 5.38E-29 |
| 22276 | Nr4a2 | -1.50238 | 2.11E-89 |
| 3157 | Bcl3 | -1.50217 | 3.62E-20 |
| 17239 | Ifi202b | -1.50202 | 4.76E-07 |
| 7169 | Fam217b | -1.50154 | 1.22E-20 |
| 5109 | Cracdl | -1.49685 | 4.79E-30 |
| 5503 | Cyp2s1 | -1.49682 | 6.41E-25 |
| 22633 | Ooep | -1.49534 | 0.007516 |
| 17077 | Hs3st3a1 | -1.49395 | 1.29E-23 |
| 16286 | Gpr20 | -1.48744 | 0.007026 |
| 28586 | Sox2ot | -1.48683 | 0.002291 |
| 28324 | Snap91 | -1.48595 | 2.48E-22 |
| 27764 | Slc10a6 | -1.48186 | 0.000285 |
| 2332 | Aox3 | -1.47484 | 1.43E-17 |
| 22311 | Nrsn1 | -1.47438 | 0.00023 |
| 7430 | Fgf1 | -1.47132 | 5.38E-09 |
| 19178 | Ly6c1 | -1.47035 | 4.67E-05 |
| 1642 | Ackr1 | -1.46869 | 8.95E-26 |
| 21195 | Mro | -1.46793 | 0.000949 |
| 1399 | A530021J07Rik | -1.46342 | 0.008717 |
| 20319 | Mir483 | -1.46315 | 5.38E-05 |
| 3742 | Casz1 | -1.45832 | 1.48E-21 |
| 31349 | Usp29 | -1.45686 | 2.54E-72 |
| 25183 | Ppp4r4 | -1.45646 | 3.06E-35 |
| 4518 | Chdh | -1.45642 | 0.001984 |
| 21420 | Mtus1 | -1.45584 | 9.87E-74 |
| 18309 | Klf9 | -1.45066 | 4.97E-54 |
| 27782 | Slc13a5 | -1.44907 | 3.19E-30 |
| 17618 | Ihh | -1.44838 | 4.07E-08 |
| 7608 | Foxj1 | -1.44755 | 4.55E-07 |
| 17067 | Hrh2 | -1.44642 | 0.000131 |
| 28295 | Smpd3 | -1.44541 | 2.52E-93 |
| 17667 | Il1r2 | -1.44067 | 1.02E-05 |
| 2516 | Arhgdig | -1.43989 | 1.18E-12 |
| 18104 | Kcnip2 | -1.43952 | 1.19E-07 |
| 18287 | Kl | -1.43946 | 0.001672 |
| 29106 | Sugct | -1.43538 | 1.52E-08 |
| 8022 | Ghr | -1.43524 | 4.14E-123 |
| 17984 | Jakmip2 | -1.43524 | 9.98E-15 |
| 27715 | Six2 | -1.43251 | 8.47E-97 |
| 6396 | E030044B06Rik | -1.4325 | 0.014841 |
| 7944 | Gcnt1 | -1.43219 | 1.12E-28 |
| 19170 | Lurap1l | -1.42984 | 2.68E-26 |
| 18592 | Lad1 | -1.42902 | 9.94E-29 |
| 7007 | Extl1 | -1.42256 | 1.52E-05 |
| 28897 | Ssc4d | -1.41651 | 1.44E-24 |
| 27354 | Selenbp1 | -1.41581 | 2.15E-19 |
| 26078 | Rbpjl | -1.41505 | 0.001985 |
| 3601 | Cadps2 | -1.41501 | 1.45E-59 |
| 22241 | Nptx1 | -1.41017 | 5.74E-40 |
| 1838 | Adgrd1 | -1.40892 | 2.77E-09 |
| 19430 | Marchf10 | -1.40687 | 0.001533 |
| 24392 | Pdzrn4 | -1.40679 | 1.76E-11 |
| 29621 | Tesc | -1.40561 | 0.000229 |
| 25471 | Prss23 | -1.40471 | 4.51E-57 |
| 30816 | Trpc5 | -1.40401 | 0.005585 |
| 25969 | Rasd1 | -1.40048 | 1.03E-06 |
| 3904 | Ccdc60 | -1.39618 | 0.001531 |
| 26334 | Rimkla | -1.39483 | 0.00416 |
| 18284 | Kit | -1.39377 | 2.63E-71 |
| 29678 | Tfap2b | -1.39341 | 1.14E-153 |
| 27837 | Slc22a18 | -1.39192 | 0.000588 |
| 25835 | Rab3b | -1.39173 | 1.26E-14 |
| 2086 | Aldh3a1 | -1.39168 | 3.27E-05 |
| 2503 | Arhgap36 | -1.3914 | 5.79E-10 |
| 6486 | Eda | -1.3908 | 2.78E-46 |
| 2142 | Alpl | -1.39051 | 5.02E-108 |
| 19503 | Mc4r | -1.39048 | 0.000159 |
| 3598 | Cadm3 | -1.38926 | 1.92E-83 |
| 4330 | Cend1 | -1.38872 | 2.17E-08 |
| 23994 | P2ry1 | -1.38714 | 5.68E-27 |
| 1789 | Adamts18 | -1.38701 | 1.86E-37 |
| 2662 | Asb4 | -1.38665 | 8.74E-61 |
| 27042 | S100a1 | -1.38252 | 8.69E-11 |
| 7068 | Fah | -1.38212 | 9.25E-23 |
| 8257 | Gm10584 | -1.38023 | 0.021513 |
| 7648 | Fras1 | -1.37741 | 2.96E-77 |
| 18165 | Kctd11 | -1.37685 | 1.18E-19 |
| 6793 | Epb41l4a | -1.37638 | 6.38E-27 |
| 2074 | Albfm1 | -1.37274 | 6.91E-07 |
| 28607 | Sp7 | -1.36953 | 5.80E-102 |
| 22079 | Nkx2-3 | -1.3648 | 1.37E-10 |
| 28960 | Stac | -1.3642 | 1.24E-19 |
| 30243 | Tnfsf11 | -1.36301 | 2.26E-20 |
| 30825 | Trpm5 | -1.35879 | 5.31E-60 |
| 24144 | Pax1 | -1.35805 | 0.000941 |
| 30823 | Trpm3 | -1.35565 | 1.11E-16 |
| 24736 | Plaat3 | -1.3521 | 5.18E-08 |
| 25076 | Ppfibp2 | -1.35104 | 3.15E-65 |
| 14555 | Gm5176 | -1.35012 | 0.004429 |
| 28134 | Slc9a7 | -1.34667 | 3.93E-05 |
| 3116 | Bc1 | -1.34637 | 6.67E-06 |
| 21004 | Mkx | -1.34593 | 2.61E-56 |
| 7013 | Eya4 | -1.34554 | 1.92E-82 |
| 29159 | Susd4 | -1.34414 | 3.20E-25 |
| 5169 | Crtam | -1.34107 | 0.002753 |
| 29708 | Tgfb3 | -1.3388 | 1.12E-98 |
| 3819 | Ccdc122 | -1.33842 | 0.00026 |
| 16524 | Gulo | -1.33766 | 0.003833 |
| 30165 | Tmem95 | -1.33617 | 0.000294 |
| 24775 | Plch1 | -1.33522 | 9.38E-08 |
| 7047 | Faah | -1.33426 | 1.98E-05 |
| 18252 | Kif26b | -1.33346 | 1.75E-97 |
| 7543 | Fmn2 | -1.32961 | 4.21E-31 |
| 28592 | Sox6 | -1.32032 | 2.37E-60 |
| 29204 | Syn1 | -1.31803 | 4.59E-15 |
| 16740 | Has3 | -1.31802 | 3.08E-21 |
| 18970 | Lonrf2 | -1.3178 | 1.61E-63 |
| 7221 | Fam83g | -1.31739 | 9.54E-10 |
| 2437 | Aqp1 | -1.31671 | 2.52E-45 |
| 1798 | Adamts8 | -1.31658 | 1.03E-11 |
| 24915 | Pnma8c | -1.31534 | 0.014228 |
| 2079 | Aldh1a2 | -1.31526 | 1.48E-86 |
| 21078 | Mob3b | -1.31314 | 4.22E-46 |
| 27775 | Slc12a8 | -1.31193 | 6.37E-05 |
| 18464 | Krt17 | -1.31162 | 7.45E-47 |
| 6344 | Dusp8 | -1.31063 | 4.73E-31 |
| 24735 | Plaat1 | -1.3103 | 0.00064 |
| 24543 | Phlda1 | -1.30973 | 1.40E-26 |
| 29130 | Sult4a1 | -1.30972 | 4.21E-17 |
| 19650 | Metrnl | -1.30868 | 6.36E-23 |
| 30971 | Ttll10 | -1.30851 | 1.85E-05 |
| 2328 | Aoc3 | -1.30713 | 5.98E-09 |
| 2130 | Alox12 | -1.30664 | 4.01E-12 |
| 4564 | Chrdl2 | -1.30661 | 4.72E-11 |
| 4120 | Cd55 | -1.30428 | 1.66E-14 |
| 6962 | Eva1c | -1.30409 | 3.97E-06 |
| 3910 | Ccdc68 | -1.3035 | 0.000851 |
| 3691 | Car3 | -1.30241 | 3.41E-23 |
| 29625 | Tesl1 | -1.30117 | 4.06E-14 |
| 7935 | Gch1 | -1.30116 | 5.29E-07 |
| 25023 | Postn | -1.29937 | 1.11E-167 |
| 29175 | Svip | -1.29925 | 6.89E-11 |
| 22301 | Nrip3 | -1.29458 | 0.004681 |
| 22439 | Nupr1 | -1.29397 | 0.000153 |
| 7998 | Gfra3 | -1.29273 | 5.12E-28 |
| 26814 | Rps17-ps1 | -1.28973 | 0.017219 |
| 2285 | Anks1b | -1.28837 | 0.000148 |
| 3430 | Btla | -1.28769 | 2.38E-05 |
| 19249 | Mab21l1 | -1.28733 | 4.82E-36 |
| 32409 | Zbtb7c | -1.28548 | 7.36E-24 |
| 9053 | Gm14267 | -1.28332 | 0.001202 |
| 21923 | Nefh | -1.28112 | 0.020481 |
| 5943 | Dgki | -1.28099 | 1.49E-10 |
| 17269 | Ifitm5 | -1.27996 | 7.01E-12 |
| 25695 | Ptpro | -1.27896 | 8.81E-08 |
| 16149 | Gng4 | -1.27726 | 7.03E-17 |
| 30879 | Tspan15 | -1.27605 | 2.33E-31 |
| 450 | 2310001H17Rik | -1.27554 | 0.003456 |
| 30821 | Trpm1 | -1.27491 | 0.007681 |
| 16565 | H2-Ab1 | -1.27283 | 0.00269 |
| 4598 | Chst7 | -1.27221 | 3.60E-08 |
| 4935 | Col2a1 | -1.2722 | 1.65E-89 |
| 26651 | Rpl26-ps6 | -1.27179 | 0.000489 |
| 28069 | Slc5a1 | -1.26955 | 0.019681 |
| 7651 | Frem1 | -1.26828 | 4.94E-95 |
| 28136 | Slc9a9 | -1.26693 | 7.45E-22 |
| 28807 | Spsb4 | -1.26671 | 3.72E-18 |
| 5376 | Cxcl1 | -1.26647 | 1.46E-24 |
| 18749 | Lgi3 | -1.26646 | 9.06E-16 |
| 24491 | Pgr | -1.26471 | 0.003451 |
| 4203 | Cdh18 | -1.26344 | 2.45E-05 |
| 6045 | Dlx3 | -1.26186 | 7.52E-22 |
| 27388 | Sema6a | -1.2615 | 2.34E-112 |
| 29509 | Tcf7 | -1.2612 | 9.26E-69 |
| 2496 | Arhgap29 | -1.2612 | 6.81E-109 |
| 26486 | Rnf43 | -1.2604 | 3.19E-24 |
| 28745 | Spint2 | -1.25828 | 1.15E-32 |
| 29619 | Tes | -1.25794 | 3.39E-67 |
| 18169 | Kctd14 | -1.25781 | 4.77E-09 |
| 2147 | Alx3 | -1.25776 | 1.18E-05 |
| 6530 | Efcab15 | -1.25733 | 0.001224 |
| 22305 | Nrn1 | -1.2572 | 0.000434 |
| 29944 | Tmem117 | -1.25683 | 6.08E-06 |
| 30102 | Tmem30b | -1.25608 | 6.86E-44 |
| 16404 | Grm4 | -1.24818 | 6.25E-06 |
| 25293 | Prickle1 | -1.2481 | 3.90E-90 |
| 18800 | Lin7b | -1.24762 | 0.001721 |
| 18294 | Klf10 | -1.24735 | 1.24E-33 |
| 529 | 2700046A07Rik | -1.24703 | 0.013621 |
| 5899 | Dennd1c | -1.24669 | 1.13E-08 |
| 23992 | P2rx6 | -1.24405 | 0.003615 |
| 18576 | Ky | -1.24274 | 0.001385 |
| 3724 | Casp12 | -1.24159 | 0.000937 |
| 30793 | Trnp1 | -1.23989 | 2.10E-06 |
| 24260 | Pclo | -1.2377 | 3.85E-05 |
| 25145 | Ppp1r3d | -1.23767 | 1.78E-06 |
| 24562 | Phyhd1 | -1.23698 | 0.011849 |
| 26412 | Rnf112 | -1.23636 | 1.50E-08 |
| 21592 | Myorg | -1.23615 | 2.39E-19 |
| 18465 | Krt18 | -1.23489 | 1.27E-13 |
| 13635 | Gm33302 | -1.23424 | 0.000536 |
| 24670 | Pitpnm1 | -1.23312 | 2.57E-26 |
| 32318 | Ydjc | -1.23265 | 5.47E-19 |
| 1847 | Adgrg1 | -1.23107 | 7.21E-23 |
| 7789 | Gabra1 | -1.22845 | 6.83E-29 |
| 24761 | Plaur | -1.22739 | 5.75E-07 |
| 27619 | Sh3rf2 | -1.22652 | 1.09E-13 |
| 29974 | Tmem145 | -1.22645 | 3.84E-20 |
| 24053 | Pak6 | -1.2257 | 2.81E-09 |
| 18628 | Large2 | -1.22398 | 4.69E-44 |
| 21711 | Nacad | -1.22355 | 1.57E-15 |
| 14609 | Gm53058 | -1.22203 | 0.010898 |
| 7792 | Gabra4 | -1.22143 | 6.66E-37 |
| 24692 | Pkdcc | -1.22076 | 2.36E-55 |
| 4903 | Cobll1 | -1.21715 | 3.14E-25 |
| 31376 | Usp6nl | -1.21707 | 4.34E-75 |
| 16939 | Hmgcll1 | -1.21633 | 6.30E-11 |
| 7220 | Fam83f | -1.21621 | 1.95E-07 |
| 14561 | Gm5200 | -1.21478 | 6.68E-07 |
| 28800 | Spry4 | -1.21384 | 9.68E-68 |
| 7188 | Fam241a | -1.21251 | 1.07E-10 |
| 4211 | Cdh3 | -1.2122 | 1.26E-34 |
| 7077 | Fam107a | -1.21113 | 4.76E-05 |
| 27772 | Slc12a5 | -1.20913 | 1.99E-42 |
| 21138 | Mpped1 | -1.20687 | 1.42E-46 |
| 3173 | Bdh1 | -1.20445 | 3.13E-22 |
| 7122 | Fam167a | -1.20314 | 1.22E-21 |
| 28584 | Sox2 | -1.20275 | 2.91E-10 |
| 29709 | Tgfbi | -1.20203 | 6.74E-98 |
| 13290 | Gm28659 | -1.19869 | 0.005024 |
| 2782 | Atp1b1 | -1.19856 | 9.11E-39 |
| 19121 | Lrrtm2 | -1.19536 | 3.36E-14 |
| 2268 | Ankrd45 | -1.19373 | 4.84E-11 |
| 24792 | Plek2 | -1.18859 | 1.21E-05 |
| 17749 | Inhba | -1.18854 | 1.02E-90 |
| 29025 | Stk32b | -1.18786 | 2.40E-54 |
| 1522 | Abca4 | -1.18254 | 2.01E-17 |
| 27579 | Sgpp2 | -1.18176 | 4.21E-06 |
| 17130 | Hspa2 | -1.1802 | 9.38E-14 |
| 15958 | Gm9347 | -1.17865 | 1.76E-27 |
| 3041 | Batf | -1.17747 | 6.09E-14 |
| 32219 | Wnt2 | -1.17648 | 4.56E-26 |
| 19324 | Mall | -1.17628 | 3.30E-07 |
| 24799 | Plekha7 | -1.17393 | 3.32E-28 |
| 1676 | Acpp | -1.17361 | 3.92E-12 |
| 25637 | Ptgir | -1.17239 | 0.021867 |
| 16632 | H2aj | -1.17102 | 1.45E-14 |
| 30258 | Tnk1 | -1.16726 | 6.19E-09 |
| 2023 | Ak8 | -1.16679 | 0.018054 |
| 28317 | Snai1 | -1.16599 | 9.48E-34 |
| 4134 | Cd74 | -1.16427 | 2.23E-07 |
| 1915 | Aff2 | -1.16372 | 3.34E-08 |
| 6754 | Eno3 | -1.16365 | 6.99E-25 |
| 4487 | Cgrrf1 | -1.163 | 1.70E-30 |
| 17867 | Irf8 | -1.16103 | 1.83E-23 |
| 29679 | Tfap2c | -1.16069 | 6.62E-71 |
| 18156 | Kcns3 | -1.15977 | 8.67E-08 |
| 23943 | Otof | -1.15963 | 0.001552 |
| 2847 | Atp8a1 | -1.1596 | 5.95E-30 |
| 26321 | Ric3 | -1.15934 | 1.75E-13 |
| 24535 | Phgdh | -1.15878 | 1.89E-24 |
| 31290 | Upk3bl | -1.15852 | 1.33E-05 |
| 29984 | Tmem154 | -1.1578 | 0.005015 |
| 19158 | Ltbr | -1.1576 | 4.27E-43 |
| 28749 | Spire1 | -1.15641 | 2.95E-62 |
| 15471 | Gm7901 | -1.15532 | 8.69E-43 |
| 5159 | Crnde | -1.1545 | 3.30E-18 |
| 7676 | Frzb | -1.15426 | 2.04E-95 |
| 22474 | Oas1a | -1.15302 | 0.003395 |
| 19104 | Lrrc8e | -1.1509 | 6.53E-05 |
| 25396 | Proser2 | -1.14729 | 7.26E-16 |
| 24415 | Per2 | -1.14645 | 6.50E-37 |
| 3211 | Bhlhe40 | -1.14417 | 1.17E-37 |
| 7934 | Gcgr | -1.14384 | 0.000947 |
| 17871 | Irgm2 | -1.14383 | 2.11E-11 |
| 7593 | Foxc2 | -1.14327 | 0.000976 |
| 25448 | Prr7 | -1.14312 | 2.61E-12 |
| 22028 | Nhs | -1.14306 | 2.65E-51 |
| 3258 | Bmp2 | -1.13927 | 4.45E-40 |
| 75 | 1700003E16Rik | -1.13872 | 1.55E-08 |
| 18486 | Krt42 | -1.13556 | 0.000114 |
| 2699 | Asprv1 | -1.13555 | 0.004124 |
| 22547 | Odad1 | -1.13365 | 8.58E-11 |
| 3811 | Ccdc116 | -1.1323 | 1.99E-08 |
| 4215 | Cdh7 | -1.13065 | 0.0003 |
| 30877 | Tspan13 | -1.13037 | 1.00E-44 |
| 145 | 1700019D03Rik | -1.13012 | 2.61E-09 |
| 32213 | Wnk4 | -1.12993 | 2.72E-30 |
| 19068 | Lrrc4 | -1.12734 | 2.87E-11 |
| 26250 | Rhbdl2 | -1.12687 | 0.008615 |
| 25612 | Ptch1 | -1.12511 | 4.95E-79 |
| 27131 | Satb1 | -1.12401 | 1.64E-29 |
| 30990 | Tub | -1.12266 | 1.82E-34 |
| 18775 | Lif | -1.12215 | 0.001984 |
| 26583 | Rpl15-ps5 | -1.12109 | 0.005669 |
| 24677 | Pitx2 | -1.12073 | 2.57E-46 |
| 30984 | Ttpa | -1.1207 | 2.35E-11 |
| 1848 | Adgrg2 | -1.12004 | 1.86E-19 |
| 7494 | Firre | -1.11909 | 1.37E-105 |
| 16126 | Gnal | -1.11846 | 2.04E-12 |
| 18019 | Junos | -1.11812 | 0.000442 |
| 25281 | Prelid3a | -1.1156 | 9.74E-24 |
| 2339 | Ap1m2 | -1.1151 | 2.45E-10 |
| 6103 | Dnah7a | -1.11484 | 0.008207 |
| 19181 | Ly6e | -1.11471 | 2.73E-37 |
| 5247 | Csrp2 | -1.11283 | 4.51E-48 |
| 4175 | Cdc42ep3 | -1.11273 | 2.28E-25 |
| 29980 | Tmem150c | -1.11165 | 0.000286 |
| 29704 | Tgfa | -1.11098 | 2.14E-05 |
| 14120 | Gm4335 | -1.11063 | 0.01045 |
| 6313 | Duox1 | -1.10902 | 9.21E-05 |
| 25613 | Ptch2 | -1.10756 | 3.53E-23 |
| 24059 | Palm3 | -1.10716 | 4.45E-27 |
| 5350 | Cubn | -1.10476 | 7.28E-09 |
| 31467 | Vit | -1.10248 | 0.000483 |
| 7011 | Eya2 | -1.10216 | 3.01E-18 |
| 25122 | Ppp1r14c | -1.10013 | 2.92E-11 |
| 30225 | Tnfrsf12a | -1.09964 | 7.37E-18 |
| 23919 | Osbpl3 | -1.09779 | 1.87E-35 |
| 3596 | Cadm1 | -1.09696 | 8.63E-56 |
| 27668 | Shtn1 | -1.09013 | 2.29E-09 |
| 6175 | Dnm3os | -1.08952 | 6.83E-73 |
| 30808 | Trp63 | -1.08929 | 4.07E-43 |
| 26358 | Ripor3 | -1.08471 | 7.30E-44 |
| 7213 | Fam81a | -1.08421 | 1.13E-10 |
| 21134 | Mpp3 | -1.0839 | 9.75E-18 |
| 6926 | Esrp1 | -1.08349 | 2.53E-30 |
| 26129 | Reep1 | -1.08313 | 9.63E-22 |
| 2678 | Asgr1 | -1.08297 | 0.01111 |
| 4916 | Col11a2 | -1.08194 | 3.77E-38 |
| 21496 | Mxra8 | -1.08175 | 4.95E-54 |
| 27288 | Sdc2 | -1.08046 | 3.95E-68 |
| 6956 | Etv4 | -1.0788 | 1.29E-34 |
| 18005 | Jph1 | -1.0782 | 4.03E-16 |
| 2208 | Angpt1 | -1.0782 | 1.25E-67 |
| 17766 | Inpp4b | -1.0782 | 1.19E-29 |
| 24276 | Pcp4l1 | -1.07813 | 1.48E-07 |
| 25691 | Ptprk | -1.07667 | 1.44E-69 |
| 2604 | Arnt2 | -1.07642 | 6.97E-69 |
| 4307 | Cebpd | -1.07621 | 8.20E-18 |
| 5085 | Cpq | -1.07537 | 5.78E-23 |
| 19556 | Mdga2 | -1.07525 | 4.32E-14 |
| 18007 | Jph3 | -1.07441 | 0.000334 |
| 7408 | Fermt1 | -1.07354 | 1.30E-11 |
| 24143 | Pawr | -1.07242 | 1.12E-20 |
| 27165 | Scara3 | -1.07215 | 1.28E-60 |
| 3709 | Carmil1 | -1.07156 | 6.66E-47 |
| 4256 | Cdkl2 | -1.07008 | 0.000861 |
| 29247 | Syt5 | -1.06865 | 2.91E-06 |
| 4756 | Clic5 | -1.06801 | 1.44E-06 |
| 4099 | Cd302 | -1.06582 | 4.29E-27 |
| 30381 | Traf1 | -1.06458 | 2.43E-06 |
| 26226 | Rgs17 | -1.06297 | 1.50E-21 |
| 3515 | C330013E15Rik | -1.06201 | 0.022806 |
| 7586 | Fosl2 | -1.06156 | 2.68E-86 |
| 21363 | Mtcl1 | -1.06077 | 2.49E-31 |
| 5560 | Cysltr1 | -1.05943 | 3.39E-05 |
| 27061 | S1pr1 | -1.05848 | 2.68E-48 |
| 7509 | Fkbp5 | -1.05766 | 3.28E-55 |
| 27017 | Runx2 | -1.0572 | 4.95E-97 |
| 28297 | Smpd5 | -1.05691 | 0.000263 |
| 25638 | Ptgis | -1.05688 | 3.87E-21 |
| 15374 | Gm7642 | -1.05684 | 0.016939 |
| 29531 | Tcp11 | -1.05604 | 3.23E-05 |
| 18604 | Lamb3 | -1.05563 | 4.60E-19 |
| 3494 | C1s2 | -1.05492 | 0.000232 |
| 24405 | Peg3 | -1.0525 | 4.59E-65 |
| 21843 | Ndnf | -1.05206 | 3.64E-59 |
| 6800 | Epcam | -1.05047 | 3.11E-24 |
| 27985 | Slc35f2 | -1.05034 | 1.28E-24 |
| 6153 | Dnajc6 | -1.05034 | 1.13E-11 |
| 6543 | Efhc2 | -1.04944 | 0.020048 |
| 7456 | Fgfr2 | -1.04784 | 3.53E-78 |
| 24088 | Paqr5 | -1.04769 | 0.004791 |
| 5142 | Crip1 | -1.04694 | 2.13E-14 |
| 3253 | Bmf | -1.04281 | 5.39E-52 |
| 27983 | Slc35e4 | -1.04158 | 3.37E-17 |
| 17045 | Hpcal4 | -1.04087 | 0.001208 |
| 25062 | Ppargc1a | -1.04017 | 2.63E-09 |
| 7767 | G630016G05Rik | -1.03908 | 0.000582 |
| 28299 | Smpdl3b | -1.03882 | 1.62E-14 |
| 24166 | Pbx3 | -1.03774 | 2.22E-56 |
| 6826 | Epn3 | -1.03284 | 5.19E-07 |
| 16828 | Hecw2 | -1.03182 | 5.67E-35 |
| 28127 | Slc9a2 | -1.03014 | 7.82E-08 |
| 28988 | Stat5a | -1.02887 | 5.14E-14 |
| 23996 | P2ry10b | -1.02737 | 5.70E-05 |
| 24273 | Pcolce2 | -1.02435 | 1.45E-08 |
| 28744 | Spint1 | -1.024 | 3.88E-09 |
| 4902 | Cobl | -1.02344 | 6.21E-17 |
| 21181 | Mrgpre | -1.02327 | 1.17E-06 |
| 19573 | Mecom | -1.01823 | 1.87E-22 |
| 27111 | Sap30 | -1.0178 | 8.66E-14 |
| 28543 | Socs3 | -1.01597 | 8.75E-45 |
| 4207 | Cdh22 | -1.01456 | 1.69E-06 |
| 32495 | Zfhx4 | -1.01418 | 2.20E-46 |
| 17910 | Itga3 | -1.01235 | 2.49E-28 |
| 16358 | Grb7 | -1.00989 | 4.21E-06 |
| 19016 | Lrig1 | -1.00855 | 2.24E-59 |
| 19172 | Luzp2 | -1.00765 | 0.000165 |
| 5917 | Deptor | -1.00713 | 2.99E-16 |
| 7811 | Gadd45a | -1.00667 | 2.15E-08 |
| 27503 | Sertm1 | -1.00649 | 0.000553 |
| 4719 | Clec11a | -1.0056 | 1.76E-26 |
| 342 | 1700108F19Rik | -1.00559 | 0.015815 |
| 7017 | Ezr | -1.00347 | 2.97E-29 |
| 21909 | Necab1 | -1.0012 | 2.60E-07 |
| 6049 | Dlx6 | -1.00101 | 2.68E-30 |
| 30124 | Tmem51 | -0.99947 | 6.81E-14 |
| 18748 | Lgi2 | -0.99876 | 7.81E-45 |
| 17141 | Hspb7 | -0.99736 | 0.008569 |
| 22349 | Nt5dc3 | -0.9973 | 3.90E-32 |
| 28199 | Smad9 | -0.99636 | 5.94E-34 |
| 3657 | Cap2 | -0.99607 | 2.08E-13 |
| 16351 | Gramd3 | -0.99564 | 7.50E-32 |
| 5071 | Cpm | -0.99472 | 2.43E-26 |
| 1495 | Aadat | -0.99472 | 0.013171 |
| 30687 | Trib1 | -0.9947 | 4.27E-23 |
| 17702 | Il3ra | -0.9944 | 1.47E-15 |
| 19422 | Mapkapk3 | -0.99377 | 1.25E-17 |
| 28542 | Socs2 | -0.99321 | 8.58E-37 |
| 3836 | Ccdc149 | -0.99283 | 5.11E-09 |
| 19025 | Lrp11 | -0.99278 | 3.55E-07 |
| 7029 | F3 | -0.99242 | 4.65E-09 |
| 21945 | Nell1 | -0.99115 | 2.61E-07 |
| 6280 | Dsc3 | -0.98972 | 3.47E-41 |
| 27236 | Schip1 | -0.98834 | 0.01309 |
| 2010 | Aipl1 | -0.9883 | 0.002596 |
| 18743 | Lgals7 | -0.98816 | 2.16E-09 |
| 18152 | Kcnq5 | -0.98785 | 5.00E-05 |
| 17216 | Id2 | -0.9874 | 3.26E-42 |
| 19399 | Map7d2 | -0.98683 | 0.001085 |
| 17257 | Ifi47 | -0.98609 | 0.015794 |
| 4236 | Cdk18 | -0.98587 | 2.50E-12 |
| 8203 | Gm10327 | -0.98546 | 0.0151 |
| 30708 | Trim29 | -0.98533 | 1.19E-21 |
| 25390 | Prom2 | -0.98496 | 0.007724 |
| 4484 | Cgn | -0.98479 | 1.14E-08 |
| 28079 | Slc5a7 | -0.98299 | 4.21E-12 |
| 24497 | Phactr2 | -0.98213 | 2.20E-47 |
| 24776 | Plch2 | -0.98148 | 1.03E-17 |
| 21199 | Mroh3 | -0.9813 | 0.005771 |
| 7320 | Fbxo32 | -0.97949 | 5.83E-15 |
| 28596 | Sox9 | -0.97908 | 2.74E-25 |
| 16222 | Gpc4 | -0.97893 | 8.53E-54 |
| 21787 | Ncam2 | -0.97861 | 2.23E-14 |
| 29156 | Susd1 | -0.97827 | 2.71E-09 |
| 30813 | Trpc3 | -0.97825 | 4.49E-20 |
| 6791 | Epb41l2 | -0.97764 | 5.14E-87 |
| 22154 | Nod2 | -0.97644 | 1.59E-11 |
| 6742 | Endod1 | -0.97636 | 1.24E-20 |
| 3642 | Camkk2 | -0.9763 | 1.30E-52 |
| 3779 | Cbr3 | -0.97584 | 6.04E-10 |
| 24287 | Pctp | -0.97545 | 2.94E-06 |
| 15290 | Gm7424 | -0.97479 | 0.006595 |
| 17218 | Id4 | -0.97439 | 2.88E-21 |
| 7097 | Fam124a | -0.97424 | 2.84E-05 |
| 16365 | Grhl2 | -0.97272 | 5.84E-14 |
| 19099 | Lrrc75b | -0.97061 | 1.35E-07 |
| 30880 | Tspan17 | -0.97056 | 5.72E-06 |
| 6217 | Dpep1 | -0.9693 | 8.00E-06 |
| 29329 | Tagln2 | -0.9693 | 1.16E-33 |
| 18461 | Krt14 | -0.96919 | 3.62E-33 |
| 29812 | Timm8a1 | -0.96901 | 1.20E-14 |
| 29859 | Tlnrd1 | -0.9687 | 1.54E-33 |
| 27945 | Slc2a6 | -0.96821 | 0.016936 |
| 21846 | Ndrg1 | -0.96671 | 1.01E-27 |
| 24724 | Pla2g4a | -0.96589 | 3.50E-27 |
| 28933 | St14 | -0.96516 | 5.65E-12 |
| 4192 | Cdcp1 | -0.96446 | 2.14E-14 |
| 18304 | Klf4 | -0.96374 | 1.22E-44 |
| 6327 | Dusp15 | -0.96223 | 0.002714 |
| 8467 | Gm11837 | -0.96152 | 0.000703 |
| 30288 | Tns4 | -0.95821 | 0.012431 |
| 25005 | Pomc | -0.95806 | 0.011671 |
| 21558 | Myo10 | -0.95721 | 6.47E-48 |
| 2526 | Arhgef19 | -0.95718 | 3.35E-24 |
| 24769 | Plcd1 | -0.95714 | 2.47E-34 |
| 14774 | Gm5739 | -0.95622 | 4.33E-06 |
| 3495 | C2 | -0.95429 | 0.001781 |
| 18588 | Lacc1 | -0.95356 | 9.16E-21 |
| 4195 | Cdh1 | -0.95323 | 2.00E-26 |
| 27004 | Rttn | -0.95223 | 3.05E-35 |
| 18490 | Krt7 | -0.95085 | 2.01E-06 |
| 18741 | Lgals3bp | -0.94934 | 1.62E-10 |
| 6795 | Epb41l4b | -0.94909 | 6.37E-21 |
| 6732 | Emp3 | -0.9462 | 3.03E-24 |
| 8387 | Gm11501 | -0.94477 | 0.00184 |
| 32608 | Zfp385c | -0.94414 | 1.19E-05 |
| 18601 | Lama5 | -0.94346 | 1.39E-40 |
| 6326 | Dusp14 | -0.94316 | 1.21E-08 |
| 21957 | Neto2 | -0.94269 | 2.56E-35 |
| 28089 | Slc6a15 | -0.94253 | 1.94E-05 |
| 24709 | Pkp3 | -0.94233 | 8.20E-15 |
| 18188 | Kdf1 | -0.94195 | 9.25E-06 |
| 24090 | Paqr7 | -0.9415 | 7.46E-08 |
| 6062 | Dmp1 | -0.94141 | 0.000343 |
| 27868 | Slc25a13 | -0.94114 | 2.40E-17 |
| 24395 | Pear1 | -0.93981 | 1.91E-32 |
| 1991 | AI661453 | -0.9393 | 1.63E-08 |
| 25649 | Pthlh | -0.93776 | 4.61E-13 |
| 17617 | Igtp | -0.93699 | 2.63E-07 |
| 21149 | Mpzl2 | -0.93692 | 3.65E-06 |
| 25982 | Rasl10b | -0.93631 | 7.95E-16 |
| 27014 | Rundc3b | -0.93599 | 4.12E-10 |
| 6571 | Egr3 | -0.93599 | 7.65E-52 |
| 17642 | Il13ra1 | -0.93591 | 4.13E-20 |
| 1737 | Acvrl1 | -0.93432 | 1.24E-27 |
| 25097 | Ppm1e | -0.93426 | 1.08E-14 |
| 6643 | Eif4ebp1 | -0.93351 | 1.63E-22 |
| 3511 | C3 | -0.9334 | 0.017835 |
| 1790 | Adamts19 | -0.93327 | 5.55E-20 |
| 27002 | Rtp4 | -0.93127 | 0.000137 |
| 17894 | Ism2 | -0.93033 | 5.96E-05 |
| 28624 | Spag16 | -0.92794 | 0.000399 |
| 24402 | Peg10 | -0.92749 | 2.00E-64 |
| 29037 | Stmn2 | -0.92748 | 7.64E-12 |
| 17233 | Ier3 | -0.92689 | 4.58E-19 |
| 21020 | Mlph | -0.92669 | 2.25E-05 |
| 29226 | Synpo | -0.92502 | 5.95E-53 |
| 21988 | Nfe2l2 | -0.92475 | 2.53E-37 |
| 1407 | A530072M11Rik | -0.924 | 0.003183 |
| 30809 | Trp73 | -0.9231 | 1.32E-10 |
| 9090 | Gm14418 | -0.92138 | 5.83E-07 |
| 7957 | Gdf10 | -0.92137 | 4.80E-45 |
| 26943 | Rrs1 | -0.92016 | 6.52E-24 |
| 21979 | Nfasc | -0.91979 | 6.63E-07 |
| 26920 | Rrad | -0.91962 | 0.000146 |
| 32078 | Vwce | -0.91857 | 3.89E-15 |
| 2301 | Ano9 | -0.91791 | 7.21E-05 |
| 19383 | Map3k5 | -0.91755 | 1.49E-14 |
| 18607 | Lamc3 | -0.91753 | 5.96E-17 |
| 22029 | Nhsl1 | -0.91698 | 2.46E-44 |
| 32073 | Vwa5b2 | -0.91679 | 8.10E-08 |
| 17739 | Inava | -0.9163 | 6.42E-30 |
| 19211 | Lypd3 | -0.91501 | 0.006259 |
| 4053 | Cd164l2 | -0.9142 | 0.021748 |
| 19706 | Mfsd2a | -0.91316 | 2.76E-10 |
| 18606 | Lamc2 | -0.91188 | 1.08E-21 |
| 4578 | Chrna7 | -0.91093 | 0.004232 |
| 32249 | Wwp2 | -0.9106 | 2.59E-44 |
| 5037 | Cox6b2 | -0.91023 | 1.75E-11 |
| 9435 | Gm15867 | -0.90994 | 0.001825 |
| 18157 | Kcnt1 | -0.90978 | 1.53E-06 |
| 26678 | Rpl31-ps4 | -0.9097 | 0.009591 |
| 2490 | Arhgap24 | -0.90808 | 9.85E-36 |
| 16088 | Gm9899 | -0.9076 | 5.21E-10 |
| 27797 | Slc16a3 | -0.90705 | 4.47E-24 |
| 18291 | Klc3 | -0.90484 | 7.34E-05 |
| 2312 | Anxa11 | -0.90028 | 3.30E-17 |
| 24845 | Plp2 | -0.89984 | 1.93E-15 |
| 28590 | Sox5 | -0.89927 | 4.60E-07 |
| 19157 | Ltbp4 | -0.89922 | 2.46E-44 |
| 27792 | Slc16a11 | -0.89849 | 0.000527 |
| 1544 | Abcc4 | -0.89808 | 6.58E-06 |
| 30951 | Ttc39b | -0.89501 | 3.26E-21 |
| 2209 | Angpt2 | -0.89357 | 0.000485 |
| 3539 | C77080 | -0.89301 | 3.39E-34 |
| 23915 | Osbpl10 | -0.89242 | 6.63E-15 |
| 19405 | Mapk12 | -0.89242 | 4.19E-11 |
| 5163 | Crocc2 | -0.89204 | 0.007381 |
| 18088 | Kcne3 | -0.8898 | 1.03E-14 |
| 2973 | B3glct | -0.8897 | 1.69E-45 |
| 26101 | Rcor2 | -0.88938 | 4.72E-34 |
| 32220 | Wnt2b | -0.88889 | 1.64E-08 |
| 14921 | Gm6169 | -0.88874 | 7.46E-31 |
| 18016 | Jun | -0.88869 | 4.91E-52 |
| 29690 | Tfeb | -0.88848 | 9.16E-10 |
| 30004 | Tmem176a | -0.88767 | 8.65E-25 |
| 16139 | Gnb4 | -0.887 | 3.38E-42 |
| 7630 | Foxp4 | -0.88455 | 5.84E-34 |
| 28546 | Socs6 | -0.88413 | 5.41E-41 |
| 2284 | Anks1 | -0.88375 | 3.70E-39 |
| 6819 | Ephx2 | -0.88364 | 2.47E-06 |
| 4712 | Cldn6 | -0.8833 | 2.96E-10 |
| 16875 | Hhex | -0.88272 | 1.19E-06 |
| 30701 | Trim21 | -0.88246 | 4.35E-09 |
| 30015 | Tmem184a | -0.88115 | 0.008717 |
| 19144 | Lsmem2 | -0.88099 | 5.67E-05 |
| 13753 | Gm3500 | -0.88085 | 0.001111 |
| 21910 | Necab2 | -0.88083 | 2.76E-17 |
| 6693 | Elovl2 | -0.88056 | 5.31E-08 |
| 18981 | Lpar3 | -0.87931 | 0.001451 |
| 15927 | Gm9240 | -0.87906 | 0.010009 |
| 28106 | Slc7a1 | -0.87855 | 1.03E-27 |
| 25369 | Prmt3 | -0.87822 | 7.51E-30 |
| 24860 | Pls1 | -0.87631 | 0.000213 |
| 4825 | Cmya5 | -0.87593 | 8.52E-15 |
| 4710 | Cldn4 | -0.8756 | 2.91E-06 |
| 4400 | Cers4 | -0.87272 | 4.57E-28 |
| 7756 | Fzd6 | -0.87248 | 1.50E-13 |
| 2454 | Arc | -0.87104 | 2.49E-12 |
| 29872 | Tlx1 | -0.87097 | 0.004964 |
| 4279 | Cds1 | -0.87069 | 5.88E-10 |
| 14964 | Gm6285 | -0.87068 | 0.000101 |
| 24333 | Pde8a | -0.86975 | 3.63E-22 |
| 4885 | Cntn6 | -0.86913 | 1.55E-35 |
| 10275 | Gm19175 | -0.86851 | 0.019914 |
| 19034 | Lrp8 | -0.86748 | 7.21E-23 |
| 25824 | Rab32 | -0.86737 | 9.85E-14 |
| 17162 | Htr3a | -0.86733 | 0.019743 |
| 24092 | Paqr9 | -0.86541 | 0.005692 |
| 16466 | Gstt2 | -0.86451 | 0.000198 |
| 21034 | Mmel1 | -0.86371 | 0.019401 |
| 16739 | Has2os | -0.86338 | 0.000191 |
| 5398 | Cyb561 | -0.86281 | 3.32E-05 |
| 27502 | Sertad4 | -0.86256 | 1.22E-32 |
| 28347 | Snhg4 | -0.86223 | 1.05E-17 |
| 5606 | D430041D05Rik | -0.86142 | 9.87E-05 |
| 3184 | Bend4 | -0.86141 | 3.13E-10 |
| 16080 | Gm9797 | -0.86104 | 9.52E-11 |
| 27663 | Shq1 | -0.86092 | 5.62E-10 |
| 22309 | Nrp2 | -0.86079 | 3.38E-60 |
| 6293 | Dsp | -0.85826 | 5.59E-47 |
| 7473 | Fhit | -0.8578 | 0.001653 |
| 25038 | Pou2af3 | -0.85743 | 0.002026 |
| 24732 | Pla2g7 | -0.85724 | 1.58E-08 |
| 14424 | Gm49252 | -0.85723 | 0.000478 |
| 24076 | Panx3 | -0.85623 | 2.16E-33 |
| 18344 | Klhl34 | -0.85616 | 2.19E-08 |
| 7674 | Fry | -0.85609 | 7.08E-11 |
| 25702 | Ptprv | -0.85536 | 1.29E-34 |
| 5132 | Creg1 | -0.85233 | 3.62E-25 |
| 31190 | Uchl1 | -0.85184 | 3.76E-15 |
| 8062 | Gjb3 | -0.8505 | 0.017583 |
| 14770 | Gm5732 | -0.85043 | 0.016205 |
| 18740 | Lgals3 | -0.85037 | 0.000123 |
| 552 | 2810442N19Rik | -0.85029 | 7.99E-09 |
| 26752 | Rpp38 | -0.84904 | 2.95E-05 |
| 4671 | Clcf1 | -0.84888 | 1.94E-39 |
| 31373 | Usp51 | -0.84847 | 0.020939 |
| 29866 | Tlr4 | -0.84834 | 0.00257 |
| 15435 | Gm7803 | -0.84832 | 2.88E-05 |
| 24082 | Pappa | -0.84721 | 1.17E-11 |
| 15608 | Gm8281 | -0.84688 | 0.00402 |
| 21769 | Nat8l | -0.84684 | 4.97E-07 |
| 21566 | Myo1b | -0.84624 | 8.63E-57 |
| 23965 | Otulinl | -0.8462 | 2.30E-13 |
| 31444 | Vdr | -0.84597 | 0.000875 |
| 24622 | Pik3ip1 | -0.84422 | 1.64E-13 |
| 32215 | Wnt10a | -0.84408 | 9.81E-05 |
| 17779 | Insig2 | -0.84363 | 4.75E-26 |
| 1619 | Acan | -0.84349 | 0.000876 |
| 27656 | Shmt1 | -0.84281 | 2.50E-26 |
| 27355 | Selenbp2 | -0.84241 | 0.006083 |
| 17171 | Htra3 | -0.84165 | 4.11E-13 |
| 21325 | Msi2 | -0.84129 | 1.46E-37 |
| 18873 | Lnx2 | -0.84124 | 9.45E-27 |
| 7425 | Fgd2 | -0.8412 | 7.64E-07 |
| 30065 | Tmem238 | -0.84097 | 0.009771 |
| 3921 | Ccdc8 | -0.84038 | 6.67E-34 |
| 7245 | Fap | -0.83885 | 6.54E-20 |
| 6642 | Eif4e3 | -0.83865 | 7.32E-20 |
| 30107 | Tmem37 | -0.83763 | 0.000681 |
| 31020 | Tuft1 | -0.83738 | 7.56E-10 |
| 19567 | Me1 | -0.83729 | 8.75E-09 |
| 6669 | Elf3 | -0.83691 | 0.010656 |
| 19164 | Ltv1 | -0.83669 | 2.93E-31 |
| 25730 | Pvt1 | -0.83651 | 2.11E-10 |
| 26355 | Ripk4 | -0.83642 | 6.61E-07 |
| 26 | 1110032F04Rik | -0.83589 | 0.008902 |
| 24473 | Pgf | -0.83578 | 1.40E-20 |
| 16260 | Gpr146 | -0.83499 | 3.18E-05 |
| 25140 | Ppp1r36 | -0.83406 | 0.002124 |
| 27416 | Septin6 | -0.83307 | 1.40E-36 |
| 4636 | Cisd3 | -0.83296 | 0.010707 |
| 26350 | Riox1 | -0.83282 | 3.09E-10 |
| 17059 | Hpse2 | -0.83208 | 0.010275 |
| 24566 | Pi15 | -0.83132 | 3.63E-05 |
| 6255 | Dpysl5 | -0.83104 | 3.15E-11 |
| 16912 | Hk2 | -0.83097 | 3.84E-35 |
| 24282 | Pcsk4 | -0.82925 | 7.69E-06 |
| 4446 | Cfap45 | -0.82924 | 0.001839 |
| 4845 | Cnksr2 | -0.82908 | 2.44E-24 |
| 28341 | Snhg15 | -0.82907 | 2.99E-15 |
| 16420 | Gsc | -0.82681 | 1.61E-13 |
| 17153 | Htatip2 | -0.82651 | 2.77E-05 |
| 6031 | Dlgap1 | -0.82426 | 8.48E-07 |
| 30187 | Tmprss13 | -0.82418 | 0.001024 |
| 5577 | D030028A08Rik | -0.82407 | 2.25E-07 |
| 28998 | Steap3 | -0.82374 | 4.43E-07 |
| 25314 | Prkar2b | -0.82307 | 4.54E-30 |
| 16986 | Homer2 | -0.82212 | 2.25E-14 |
| 6553 | Efnb2 | -0.82071 | 5.75E-52 |
| 29745 | Thbs3 | -0.82053 | 1.55E-35 |
| 25956 | Rarb | -0.82024 | 6.41E-13 |
| 21274 | Mrps28 | -0.82012 | 1.45E-10 |
| 3740 | Castor1 | -0.81969 | 0.000174 |
| 22219 | Nphp3 | -0.81803 | 2.52E-34 |
| 30958 | Ttc7 | -0.81792 | 1.22E-11 |
| 7744 | Fyb | -0.81773 | 1.33E-07 |
| 7778 | Gab1 | -0.81755 | 4.47E-56 |
| 22362 | Ntn5 | -0.81663 | 0.001652 |
| 3276 | Bnc2 | -0.81598 | 2.70E-18 |
| 21906 | Neat1 | -0.81594 | 1.21E-20 |
| 26261 | Rhobtb1 | -0.81532 | 2.34E-31 |
| 25486 | Prss41 | -0.81493 | 0.000344 |
| 19610 | Mef2c | -0.81437 | 2.39E-47 |
| 29843 | Tlcd4 | -0.81432 | 0.000172 |
| 1781 | Adamts1 | -0.8136 | 1.29E-50 |
| 1742 | Ada | -0.81356 | 5.12E-07 |
| 26221 | Rgs11 | -0.81298 | 8.56E-11 |
| 138 | 1700018A04Rik | -0.81231 | 0.002681 |
| 16877 | Hhipl1 | -0.81215 | 4.79E-07 |
| 18692 | Lcp1 | -0.81206 | 5.21E-25 |
| 19716 | Mfsd6 | -0.81138 | 0.000151 |
| 7082 | Fam110d | -0.81115 | 1.25E-05 |
| 27272 | Scrn1 | -0.81083 | 1.02E-12 |
| 28951 | St8sia1 | -0.81067 | 4.42E-08 |
| 17860 | Irf2bp2 | -0.81055 | 1.95E-26 |
| 25512 | Prxl2c | -0.81033 | 1.23E-17 |
| 27948 | Slc2a9 | -0.81029 | 0.002828 |
| 6761 | Enpp2 | -0.80934 | 1.82E-45 |
| 18273 | Kifc3 | -0.80933 | 1.29E-12 |
| 24829 | Plin2 | -0.80837 | 1.10E-24 |
| 7170 | Fam219a | -0.808 | 8.50E-15 |
| 5693 | Dcaf12l2 | -0.8078 | 0.000114 |
| 3151 | Bcl2l11 | -0.80723 | 7.54E-33 |
| 27296 | Sdf2l1 | -0.80689 | 2.46E-12 |
| 15096 | Gm6740 | -0.80624 | 0.007051 |
| 6806 | Epha2 | -0.80618 | 6.95E-21 |
| 32187 | Wfdc2 | -0.80574 | 1.72E-05 |
| 29686 | Tfcp2l1 | -0.80541 | 0.003492 |
| 27800 | Slc16a6 | -0.80523 | 1.74E-07 |
| 21382 | Mthfd2 | -0.80441 | 1.15E-23 |
| 26156 | Repin1 | -0.80359 | 6.54E-16 |
| 27392 | Sema7a | -0.8032 | 4.24E-12 |
| 7749 | Fzd1 | -0.80114 | 1.09E-41 |
| 13390 | Gm29920 | -0.79977 | 1.36E-06 |
| 2218 | Ank | -0.79869 | 7.73E-21 |
| 17928 | Itgb3 | -0.79702 | 9.38E-17 |
| 3687 | Car13 | -0.79687 | 4.93E-13 |
| 22587 | Olfml2a | -0.79683 | 1.85E-23 |
| 5982 | Dhx58 | -0.79662 | 1.72E-05 |
| 7146 | Fam185a | -0.79636 | 2.69E-11 |
| 29817 | Timp1 | -0.79489 | 1.43E-11 |
| 27903 | Slc25a45 | -0.79465 | 3.32E-06 |
| 18130 | Kcnk3 | -0.79447 | 0.002497 |
| 2996 | B4galt5 | -0.79445 | 4.69E-28 |
| 15563 | Gm8168 | -0.79293 | 0.004605 |
| 17930 | Itgb4 | -0.79256 | 1.02E-18 |
| 21963 | Neurl1b | -0.79208 | 6.10E-09 |
| 28124 | Slc8a3 | -0.7919 | 9.40E-22 |
| 14746 | Gm5684 | -0.79122 | 2.33E-06 |
| 2696 | Asphd2 | -0.791 | 8.01E-14 |
| 26781 | Rps12-ps16 | -0.78993 | 0.01595 |
| 32305 | Xxylt1 | -0.78805 | 4.81E-32 |
| 28939 | St3gal5 | -0.78768 | 5.77E-21 |
| 29988 | Tmem160 | -0.78765 | 4.38E-07 |
| 27927 | Slc27a6 | -0.78737 | 6.68E-08 |
| 32357 | Zadh2 | -0.7871 | 3.80E-31 |
| 25640 | Ptgr2 | -0.7867 | 2.29E-10 |
| 2441 | Aqp3 | -0.7864 | 0.000424 |
| 16436 | Gsg1l | -0.78564 | 3.06E-15 |
| 32416 | Zc2hc1c | -0.7848 | 1.37E-06 |
| 4922 | Col17a1 | -0.78425 | 2.05E-28 |
| 6048 | Dlx5 | -0.78413 | 1.05E-23 |
| 16850 | Hes1 | -0.78354 | 1.42E-13 |
| 31436 | Vcan | -0.78301 | 2.13E-40 |
| 1818 | Adcy2 | -0.78282 | 2.82E-20 |
| 25064 | Ppat | -0.78279 | 4.28E-23 |
| 26248 | Rhbdf2 | -0.78278 | 9.22E-10 |
| 14567 | Gm5214 | -0.78266 | 0.003051 |
| 17991 | Jcad | -0.78261 | 1.34E-30 |
| 16433 | Gsdme | -0.78176 | 1.51E-11 |
| 7426 | Fgd3 | -0.78154 | 4.27E-05 |
| 25381 | Procr | -0.78101 | 1.68E-05 |
| 4197 | Cdh11 | -0.78087 | 1.05E-58 |
| 28500 | Sntb2 | -0.78006 | 1.48E-27 |
| 4593 | Chst15 | -0.77989 | 2.82E-39 |
| 21990 | Nfia | -0.77968 | 7.93E-45 |
| 1793 | Adamts3 | -0.77781 | 2.85E-13 |
| 28562 | Sorcs3 | -0.77767 | 1.26E-06 |
| 6289 | Dsg2 | -0.77687 | 5.39E-17 |
| 19146 | Lsr | -0.77561 | 5.38E-07 |
| 7176 | Fam222a | -0.77552 | 9.80E-05 |
| 18630 | Larp1b | -0.77488 | 0.006959 |
| 14512 | Gm5093 | -0.77386 | 0.010185 |
| 7522 | Fli1 | -0.77316 | 1.69E-10 |
| 17671 | Il1rl1 | -0.77245 | 0.000748 |
| 16927 | Hmga1b | -0.77228 | 1.66E-27 |
| 5094 | Cpt1a | -0.77185 | 9.67E-29 |
| 1244 | 9030612E09Rik | -0.77175 | 0.012007 |
| 15746 | Gm8692 | -0.77163 | 0.006923 |
| 2538 | Arhgef5 | -0.77132 | 6.99E-26 |
| 24937 | Podnl1 | -0.77037 | 9.94E-06 |
| 30904 | Tspyl5 | -0.76955 | 1.05E-06 |
| 28979 | Stard5 | -0.76933 | 1.28E-20 |
| 29849 | Tle4 | -0.76872 | 4.39E-38 |
| 24975 | Polr1f | -0.76858 | 5.18E-19 |
| 27649 | Shisal1 | -0.76729 | 5.06E-08 |
| 26192 | Rflnb | -0.76706 | 5.43E-31 |
| 7490 | Fignl2 | -0.76702 | 7.45E-07 |
| 4594 | Chst2 | -0.76609 | 5.01E-30 |
| 18758 | Lhfp | -0.76566 | 2.75E-25 |
| 27377 | Sema3e | -0.7636 | 4.73E-15 |
| 22625 | Omd | -0.76349 | 3.75E-11 |
| 25890 | Rad51b | -0.76286 | 7.42E-08 |
| 14195 | Gm45596 | -0.76271 | 0.001889 |
| 7025 | F2r | -0.76219 | 5.99E-44 |
| 29146 | Supt3 | -0.76185 | 2.94E-08 |
| 28253 | Smim20 | -0.76166 | 7.11E-09 |
| 17870 | Irgm1 | -0.76101 | 1.27E-06 |
| 5212 | Csf2rb2 | -0.76006 | 0.00054 |
| 3703 | Card6 | -0.75964 | 5.82E-05 |
| 28146 | Slco2a1 | -0.75917 | 1.25E-15 |
| 33016 | Zswim6 | -0.75888 | 4.04E-23 |
| 2569 | Arl4c | -0.75846 | 1.58E-15 |
| 10479 | Gm20751 | -0.75836 | 0.00311 |
| 17906 | Itga10 | -0.75828 | 5.80E-13 |
| 21381 | Mthfd1l | -0.75818 | 1.07E-21 |
| 26096 | Rcl1 | -0.75802 | 1.00E-18 |
| 3713 | Carns1 | -0.75789 | 0.008332 |
| 25386 | Prokr1 | -0.75759 | 2.93E-06 |
| 22298 | Nrgn | -0.75707 | 3.54E-08 |
| 30846 | Tsc22d1 | -0.75662 | 2.14E-35 |
| 7730 | Fut8 | -0.75605 | 4.11E-19 |
| 26080 | Rbpms2 | -0.75605 | 4.49E-10 |
| 25701 | Ptpru | -0.75531 | 7.61E-30 |
| 24741 | Plac9 | -0.75482 | 0.003 |
| 6027 | Dlg2 | -0.75425 | 4.26E-14 |
| 22038 | Nifk | -0.75414 | 1.59E-19 |
| 19320 | Mak16 | -0.75374 | 2.77E-14 |
| 14527 | Gm5121 | -0.75347 | 0.000682 |
| 13666 | Gm3383 | -0.75324 | 0.005217 |
| 26317 | Rhpn2 | -0.75308 | 6.08E-05 |
| 7716 | Fuca2 | -0.75254 | 2.44E-18 |
| 4954 | Col8a2 | -0.75208 | 1.59E-27 |
| 29705 | Tgfb1 | -0.75032 | 1.13E-14 |
| 7538 | Flvcr2 | -0.75004 | 9.76E-05 |
| 25996 | Rassf9 | -0.74972 | 0.001401 |
| 22233 | Npr1 | -0.74915 | 2.98E-10 |
| 16778 | Hck | -0.74889 | 0.001435 |
| 8007 | Ggct | -0.74829 | 0.000852 |
| 32438 | Zcchc12 | -0.74796 | 5.37E-05 |
| 21425 | Muc1 | -0.74764 | 0.000139 |
| 6331 | Dusp2 | -0.74751 | 8.79E-07 |
| 5932 | Dgat2 | -0.74738 | 1.67E-09 |
| 25762 | Pyurf | -0.7466 | 4.95E-08 |
| 25130 | Ppp1r1a | -0.74611 | 5.00E-05 |
| 30891 | Tspan7 | -0.7457 | 3.45E-23 |
| 14816 | Gm5850 | -0.74569 | 0.018842 |
| 2601 | Armh4 | -0.74492 | 3.25E-12 |
| 2267 | Ankrd44 | -0.74458 | 9.93E-32 |
| 19338 | Man1c1 | -0.74385 | 3.59E-16 |
| 16926 | Hmga1 | -0.74315 | 2.97E-25 |
| 7145 | Fam184b | -0.74291 | 0.000549 |
| 15998 | Gm9449 | -0.74158 | 0.000124 |
| 21191 | Mrm1 | -0.7413 | 1.30E-07 |
| 16214 | Gpatch4 | -0.741 | 8.43E-14 |
| 22257 | Nr1d2 | -0.74085 | 8.88E-15 |
| 22414 | Numb | -0.74056 | 4.58E-28 |
| 17236 | Ier5l | -0.74052 | 3.33E-11 |
| 28116 | Slc7a5 | -0.73986 | 7.15E-28 |
| 28345 | Snhg20 | -0.73924 | 1.06E-07 |
| 22354 | Ntf3 | -0.73868 | 7.47E-08 |
| 2082 | Aldh1b1 | -0.73827 | 0.009188 |
| 28569 | Sostdc1 | -0.73786 | 1.67E-20 |
| 2752 | Atg9b | -0.73762 | 0.018986 |
| 4956 | Col9a2 | -0.73731 | 1.39E-20 |
| 6757 | Enox1 | -0.73669 | 7.68E-13 |
| 5964 | Dhrs7 | -0.73642 | 1.30E-06 |
| 1904 | Aen | -0.73559 | 3.65E-18 |
| 24318 | Pde3b | -0.73538 | 4.29E-23 |
| 22027 | Nhp2 | -0.7353 | 5.54E-14 |
| 7457 | Fgfr3 | -0.73458 | 4.38E-28 |
| 25394 | Pros1 | -0.7343 | 4.08E-30 |
| 2400 | Apobr | -0.73417 | 0.008593 |
| 5032 | Cox5a | -0.73375 | 8.47E-06 |
| 5918 | Dera | -0.73336 | 1.28E-09 |
| 17268 | Ifitm3 | -0.73296 | 2.31E-11 |
| 32408 | Zbtb7b | -0.73237 | 1.14E-05 |
| 30788 | Trmt61a | -0.73097 | 1.13E-10 |
| 22386 | Nudt10 | -0.7308 | 0.001027 |
| 4329 | Cemip2 | -0.73067 | 5.72E-37 |
| 31196 | Uck2 | -0.72986 | 1.25E-33 |
| 4082 | Cd274 | -0.72956 | 0.001615 |
| 6836 | Eps8 | -0.72877 | 2.93E-19 |
| 26166 | Ret | -0.72819 | 0.000161 |
| 24419 | Perp | -0.72809 | 9.04E-11 |
| 24810 | Plekhg4 | -0.72802 | 2.96E-05 |
| 3259 | Bmp2k | -0.72756 | 3.32E-17 |
| 18709 | Ldlrad3 | -0.72747 | 6.83E-31 |
| 28715 | Sphk1 | -0.72745 | 4.26E-05 |
| 30153 | Tmem86a | -0.72604 | 1.48E-09 |
| 7845 | Galnt7 | -0.72459 | 2.08E-13 |
| 21329 | Msl3l2 | -0.72294 | 3.80E-06 |
| 4640 | Cited2 | -0.72245 | 1.53E-19 |
| 24559 | Phtf2 | -0.72244 | 8.39E-22 |
| 32310 | Yaf2 | -0.7224 | 7.29E-14 |
| 7489 | Fignl1 | -0.72181 | 4.47E-14 |
| 2689 | Asns | -0.72173 | 1.69E-16 |
| 515 | 2610035D17Rik | -0.72124 | 4.21E-05 |
| 4283 | Cdv3 | -0.72111 | 1.39E-29 |
| 26687 | Rpl34-ps2 | -0.71966 | 0.014935 |
| 18855 | Lmo4 | -0.71928 | 1.32E-22 |
| 27726 | Skap2 | -0.71894 | 4.73E-05 |
| 4684 | Cldn12 | -0.71861 | 2.48E-22 |
| 25721 | Pus1 | -0.71856 | 2.39E-13 |
| 29321 | Tafa2 | -0.71841 | 3.67E-05 |
| 29183 | Swap70 | -0.718 | 2.36E-27 |
| 31291 | Upp1 | -0.71701 | 0.003733 |
| 15271 | Gm7384 | -0.71643 | 0.006816 |
| 29498 | Tcerg1l | -0.71613 | 0.020439 |
| 3052 | BB218582 | -0.71554 | 0.001615 |
| 16989 | Hook1 | -0.71541 | 9.84E-10 |
| 4846 | Cnksr3 | -0.71458 | 7.99E-15 |
| 1468 | A930033H14Rik | -0.71422 | 2.52E-05 |
| 17913 | Itga6 | -0.71402 | 9.76E-34 |
| 4889 | Cntnap4 | -0.71393 | 0.000115 |
| 21347 | Msx2 | -0.71342 | 0.002243 |
| 24339 | Pdgfc | -0.71338 | 9.31E-17 |
| 18834 | Llph | -0.71278 | 9.51E-10 |
| 5808 | Def6 | -0.71255 | 9.28E-10 |
| 17057 | Hps6 | -0.71243 | 0.001028 |
| 2892 | Aunip | -0.71226 | 0.000228 |
| 4553 | Chn2 | -0.71195 | 2.40E-07 |
| 14956 | Gm6257 | -0.71095 | 4.56E-11 |
| 32599 | Zfp36l1 | -0.71046 | 2.80E-45 |
| 7969 | Gdpd1 | -0.7101 | 1.15E-09 |
| 27990 | Slc35g1 | -0.71008 | 2.83E-05 |
| 3133 | Bcat1 | -0.70994 | 4.59E-14 |
| 4957 | Col9a3 | -0.70917 | 1.74E-08 |
| 6897 | Errfi1 | -0.70737 | 9.75E-21 |
| 28003 | Slc38a1 | -0.70715 | 5.84E-25 |
| 27596 | Sh2d5 | -0.70661 | 0.009486 |
| 19404 | Mapk11 | -0.70643 | 2.20E-12 |
| 28993 | Stbd1 | -0.70638 | 4.68E-10 |
| 27633 | Shc4 | -0.70617 | 0.001678 |
| 15446 | Gm7823 | -0.7057 | 8.12E-05 |
| 17049 | Hpgd | -0.70549 | 1.87E-12 |
| 1872 | Adk | -0.70547 | 3.20E-25 |
| 5110 | Cracr2a | -0.70536 | 1.06E-06 |
| 31364 | Usp43 | -0.7053 | 0.003028 |
| 13661 | Gm3373 | -0.70529 | 0.003567 |
| 7005 | Ext1 | -0.70503 | 1.50E-31 |
| 3238 | Blnk | -0.70488 | 0.002126 |
| 3176 | Bdkrb2 | -0.70488 | 0.000624 |
| 6639 | Eif4e | -0.70411 | 1.14E-27 |
| 15834 | Gm8938 | -0.70397 | 0.016834 |
| 24651 | Pip5k1b | -0.70367 | 0.006253 |
| 6196 | Dock5 | -0.70364 | 1.93E-11 |
| 24760 | Plau | -0.7032 | 1.27E-10 |
| 3263 | Bmp6 | -0.70257 | 3.65E-17 |
| 27796 | Slc16a2 | -0.70207 | 4.58E-32 |
| 29174 | Svil | -0.70192 | 2.80E-35 |
| 24047 | Pak1 | -0.70171 | 6.60E-24 |
| 26337 | Rims1 | -0.7016 | 6.13E-06 |
| 17063 | Hrc | -0.70115 | 2.05E-05 |
| 8224 | Gm10406 | -0.70024 | 0.016684 |
| 15617 | Gm8307 | -0.70024 | 0.007653 |
| 22094 | Nln | -0.6998 | 6.19E-16 |
| 4844 | Cnksr1 | -0.69838 | 0.007172 |
| 20027 | Mir22hg | -0.69828 | 0.002852 |
| 26158 | Reps2 | -0.69684 | 4.23E-12 |
| 16984 | Hoga1 | -0.69673 | 7.59E-05 |
| 16460 | Gsto1 | -0.69628 | 4.56E-17 |
| 22366 | Ntrk1 | -0.69609 | 5.93E-06 |
| 26007 | Rbbp7 | -0.69606 | 8.41E-25 |
| 7271 | Fbl | -0.69592 | 7.22E-08 |
| 8530 | Gm12047 | -0.69467 | 0.000184 |
| 24883 | Pm20d2 | -0.69454 | 0.000111 |
| 13567 | Gm3252 | -0.69452 | 0.007983 |
| 27373 | Sema3a | -0.69359 | 1.25E-36 |
| 22036 | Nid2 | -0.69235 | 2.68E-32 |
| 29848 | Tle3 | -0.69213 | 7.24E-27 |
| 5331 | Ctsl | -0.69188 | 8.68E-22 |
| 26461 | Rnf208 | -0.69186 | 1.62E-05 |
| 32769 | Zfp750 | -0.69179 | 0.000432 |
| 25725 | Pus7l | -0.69144 | 4.49E-10 |
| 26968 | Rspo4 | -0.69103 | 0.016836 |
| 2271 | Ankrd50 | -0.69052 | 3.23E-32 |
| 8247 | Gm10516 | -0.69041 | 0.007582 |
| 19673 | Mettl7a1 | -0.69029 | 7.92E-08 |
| 18069 | Kcna5 | -0.68951 | 0.003652 |
| 24613 | Pik3ap1 | -0.68929 | 0.000181 |
| 17074 | Hs2st1 | -0.68926 | 1.26E-33 |
| 30811 | Trpc1 | -0.68888 | 1.29E-06 |
| 26153 | Renbp | -0.68879 | 2.87E-11 |
| 29624 | Tesk2 | -0.68874 | 3.21E-10 |
| 1882 | Adpgk | -0.6884 | 1.48E-21 |
| 24427 | Pex11g | -0.6878 | 0.006537 |
| 1855 | Adgrl2 | -0.68714 | 7.43E-36 |
| 29825 | Tiparp | -0.68677 | 1.55E-21 |
| 32245 | Wwc1 | -0.68667 | 4.99E-12 |
| 32453 | Zdbf2 | -0.6866 | 1.16E-19 |
| 29047 | Ston2 | -0.68622 | 1.06E-11 |
| 22003 | Nfkbiz | -0.68603 | 6.80E-31 |
| 9735 | Gm17980 | -0.68575 | 5.91E-10 |
| 13672 | Gm33933 | -0.68568 | 0.008529 |
| 21578 | Myo6 | -0.68493 | 3.84E-28 |
| 3023 | Bahd1 | -0.68458 | 2.15E-18 |
| 13407 | Gm3017 | -0.68394 | 0.009771 |
| 30710 | Trim30a | -0.68366 | 0.013916 |
| 18616 | Lamtor3-ps | -0.68344 | 0.003775 |
| 1947 | Agpat2 | -0.68311 | 5.67E-08 |
| 26386 | Rn7sk | -0.68297 | 0.015023 |
| 2435 | Aprt-ps | -0.68265 | 6.21E-05 |
| 17254 | Ifi35 | -0.68215 | 0.000128 |
| 1362 | A2m | -0.68163 | 0.018844 |
| 25633 | Ptges3 | -0.68163 | 4.29E-18 |
| 28249 | Smim15 | -0.68113 | 1.44E-15 |
| 27260 | Scnn1a | -0.68019 | 8.74E-05 |
| 24361 | Pdlim3 | -0.67974 | 0.00069 |
| 18586 | L3mbtl3 | -0.67971 | 1.57E-32 |
| 28129 | Slc9a3r1 | -0.6792 | 2.51E-07 |
| 27539 | Sfn | -0.67914 | 2.17E-09 |
| 18190 | Kdm1b | -0.67907 | 4.55E-18 |
| 27380 | Sema4a | -0.6782 | 3.43E-08 |
| 4123 | Cd59a | -0.67786 | 8.09E-10 |
| 9065 | Gm14322 | -0.67776 | 7.66E-05 |
| 24710 | Pkp4 | -0.67762 | 1.32E-28 |
| 28029 | Slc3a2 | -0.67746 | 9.19E-21 |
| 4795 | Clu | -0.6773 | 6.50E-11 |
| 17215 | Id1 | -0.67721 | 5.39E-10 |
| 30867 | Tsku | -0.67688 | 1.86E-15 |
| 21917 | Nectin4 | -0.67685 | 4.45E-07 |
| 18023 | Kank2 | -0.67629 | 8.69E-31 |
| 19397 | Map7 | -0.67581 | 2.08E-05 |
| 26936 | Rrp12 | -0.67573 | 5.05E-17 |
| 1689 | Acsl6 | -0.67563 | 0.013457 |
| 18927 | LOC118568041 | -0.67546 | 0.000246 |
| 19680 | Mex3b | -0.67536 | 4.81E-28 |
| 5558 | Cyrib | -0.67513 | 2.04E-21 |
| 6771 | Entpd3 | -0.67465 | 8.94E-08 |
| 5758 | Ddit4 | -0.67418 | 9.30E-16 |
| 29331 | Tal1 | -0.67405 | 4.07E-06 |
| 24337 | Pdgfa | -0.67325 | 3.36E-16 |
| 14313 | Gm4767 | -0.67265 | 0.002853 |
| 435 | 2210039B01Rik | -0.67255 | 4.43E-05 |
| 24835 | Plk2 | -0.67241 | 1.36E-29 |
| 18621 | Lancl3 | -0.67239 | 0.021456 |
| 7212 | Fam78b | -0.6723 | 8.54E-08 |
| 29188 | Sybu | -0.67193 | 0.006647 |
| 8034 | Gimap5 | -0.67177 | 0.010707 |
| 6339 | Dusp3 | -0.67134 | 9.18E-12 |
| 25630 | Ptger4 | -0.67077 | 0.00017 |
| 16283 | Gpr182 | -0.67064 | 0.001398 |
| 5411 | Cyba | -0.66962 | 4.77E-10 |
| 26441 | Rnf157 | -0.6695 | 4.02E-26 |
| 14096 | Gm4270 | -0.66908 | 0.002587 |
| 22294 | Nrg2 | -0.66863 | 0.001964 |
| 28972 | Stap2 | -0.66792 | 3.68E-07 |
| 7123 | Fam167b | -0.66755 | 0.00257 |
| 4776 | Clp1 | -0.66651 | 7.64E-07 |
| 26777 | Rps12-ps12 | -0.66593 | 0.023061 |
| 18115 | Kcnj2 | -0.66508 | 1.84E-11 |
| 7709 | Fto | -0.6645 | 1.57E-33 |
| 4205 | Cdh2 | -0.66409 | 1.59E-30 |
| 26103 | Rcsd1 | -0.66383 | 1.85E-15 |
| 7161 | Fam20a | -0.66375 | 3.60E-07 |
| 16668 | H2bc4 | -0.66312 | 0.001655 |
| 7536 | Flt4 | -0.66305 | 1.75E-18 |
| 5804 | Decr1 | -0.66297 | 3.76E-14 |
| 1725 | Actr3b | -0.66288 | 0.00025 |
| 17219 | Ide | -0.6626 | 2.63E-22 |
| 21599 | Myrf | -0.66209 | 2.89E-08 |
| 32469 | Zdhhc24 | -0.66195 | 5.83E-09 |
| 24768 | Plcb4 | -0.66154 | 2.04E-17 |
| 24862 | Plscr1 | -0.66136 | 0.005205 |
| 26890 | Rps6kl1 | -0.6613 | 0.005378 |
| 19233 | Lyve1 | -0.66112 | 0.001751 |
| 27491 | Serpine2 | -0.66066 | 1.00E-28 |
| 844 | 4930523C07Rik | -0.66002 | 0.000761 |
| 19387 | Map3k8 | -0.65988 | 0.000338 |
| 30895 | Tspo | -0.65983 | 6.94E-05 |
| 4589 | Chst11 | -0.65976 | 2.81E-16 |
| 22465 | Nxt1 | -0.65971 | 2.45E-10 |
| 32133 | Wdr43 | -0.65898 | 8.44E-26 |
| 3658 | Capg | -0.65896 | 5.47E-07 |
| 4975 | Comt | -0.65878 | 4.05E-16 |
| 24411 | Pemt | -0.65867 | 0.004231 |
| 16501 | Gtpbp4 | -0.65851 | 3.32E-20 |
| 21290 | Mrto4-ps1 | -0.65842 | 5.88E-10 |
| 29791 | Tifab | -0.65829 | 0.017455 |
| 32600 | Zfp36l2 | -0.65817 | 1.19E-24 |
| 3847 | Ccdc163 | -0.6579 | 1.08E-08 |
| 3475 | C1qbp | -0.65784 | 8.35E-18 |
| 21569 | Myo1e | -0.65771 | 2.05E-19 |
| 4250 | Cdk6 | -0.65761 | 6.34E-26 |
| 16525 | Gulp1 | -0.65649 | 6.85E-19 |
| 24004 | P3h2 | -0.65641 | 2.01E-13 |
| 29033 | Stk4 | -0.65591 | 1.27E-23 |
| 24062 | Pals2 | -0.65587 | 4.59E-14 |
| 32967 | Znhit2 | -0.65546 | 0.010569 |
| 30690 | Tril | -0.65531 | 2.33E-31 |
| 15364 | Gm7614 | -0.65494 | 0.022846 |
| 14995 | Gm6376 | -0.65485 | 7.69E-05 |
| 16477 | Gtf2b | -0.65451 | 3.49E-11 |
| 7627 | Foxp1 | -0.65427 | 2.83E-21 |
| 15915 | Gm9172 | -0.65335 | 2.26E-09 |
| 6948 | Etnk2 | -0.65322 | 4.74E-05 |
| 17301 | Ifrd2 | -0.65317 | 1.05E-11 |
| 5775 | Ddx21 | -0.65292 | 7.13E-31 |
| 29773 | Thsd4 | -0.65249 | 2.54E-19 |
| 6865 | Erfe | -0.65181 | 0.004746 |
| 27653 | Shld1 | -0.6516 | 0.000563 |
| 5307 | Ctnnd2 | -0.65151 | 1.87E-06 |
| 25837 | Rab3d | -0.65143 | 2.73E-09 |
| 15944 | Gm9300 | -0.65138 | 0.001878 |
| 6524 | Eepd1 | -0.65083 | 3.65E-09 |
| 24286 | Pcsk9 | -0.65058 | 0.004265 |
| 17861 | Irf2bpl | -0.64986 | 2.61E-22 |
| 19227 | Lyrm9 | -0.64945 | 7.20E-05 |
| 25014 | Pon3 | -0.6494 | 7.73E-05 |
| 5328 | Ctsh | -0.64875 | 0.000693 |
| 6927 | Esrp2 | -0.64845 | 6.75E-06 |
| 27714 | Six1 | -0.64843 | 1.40E-32 |
| 15629 | Gm8337 | -0.64839 | 0.000967 |
| 31254 | Umps | -0.64832 | 1.36E-17 |
| 14912 | Gm6141 | -0.64819 | 0.000232 |
| 13230 | Gm27219 | -0.64813 | 1.16E-13 |
| 21122 | Mphosph10 | -0.64811 | 1.17E-13 |
| 8926 | Gm13676 | -0.648 | 1.90E-06 |
| 18096 | Kcnh2 | -0.6476 | 6.41E-06 |
| 1557 | Abcg1 | -0.64726 | 6.45E-07 |
| 1748 | Adam12 | -0.64705 | 1.13E-16 |
| 6358 | Dync1i1 | -0.64701 | 0.000337 |
| 13920 | Gm3774 | -0.64692 | 0.000102 |
| 19012 | Lrfn5 | -0.64677 | 0.000192 |
| 5416 | Cycs | -0.64669 | 1.63E-11 |
| 4930 | Col24a1 | -0.64635 | 4.81E-19 |
| 7652 | Frem2 | -0.64569 | 4.29E-16 |
| 4288 | Cdyl2 | -0.64517 | 3.07E-05 |
| 25266 | Prdm5 | -0.64455 | 8.26E-15 |
| 4103 | Cd36 | -0.64442 | 0.000443 |
| 22133 | Nmnat3 | -0.64438 | 0.015795 |
| 28810 | Sptb | -0.64438 | 0.001485 |
| 23938 | Ostf1 | -0.64415 | 1.41E-11 |
| 1687 | Acsl4 | -0.64388 | 7.25E-12 |
| 22256 | Nr1d1 | -0.64376 | 0.000679 |
| 4886 | Cntnap1 | -0.64366 | 0.001108 |
| 9349 | Gm15446 | -0.64316 | 3.24E-10 |
| 8804 | Gm13226 | -0.64245 | 0.013285 |
| 25990 | Rassf3 | -0.64228 | 2.92E-17 |
| 19376 | Map3k15 | -0.64228 | 0.011782 |
| 4997 | Coq10b | -0.64182 | 9.59E-10 |
| 25447 | Prr5l | -0.64159 | 1.94E-12 |
| 24836 | Plk3 | -0.64152 | 2.20E-11 |
| 6044 | Dlx2 | -0.6413 | 5.80E-20 |
| 28832 | Srd5a1 | -0.64099 | 1.25E-10 |
| 8093 | Gli2 | -0.64082 | 2.21E-23 |
| 18095 | Kcnh1 | -0.64044 | 3.33E-05 |
| 27548 | Sft2d2 | -0.64027 | 1.69E-16 |
| 29480 | Tcaf2 | -0.64007 | 8.88E-08 |
| 31109 | Ube2cbp | -0.64006 | 0.005174 |
| 29609 | Tep1 | -0.6395 | 7.65E-14 |
| 29796 | Tigd5 | -0.63932 | 0.000873 |
| 26171 | Retreg1 | -0.6391 | 5.72E-07 |
| 24848 | Plpp2 | -0.63904 | 8.89E-07 |
| 8851 | Gm13375 | -0.63889 | 0.000139 |
| 29945 | Tmem119 | -0.63762 | 2.02E-16 |
| 7083 | Fam111a | -0.63721 | 2.62E-16 |
| 4824 | Cmtr2 | -0.63712 | 7.24E-05 |
| 22285 | Nrbf2 | -0.63702 | 2.19E-08 |
| 18163 | Kctd1 | -0.63685 | 5.54E-17 |
| 2643 | As3mt | -0.63664 | 9.60E-10 |
| 15540 | Gm8108 | -0.6359 | 0.0059 |
| 16373 | Grid2 | -0.6353 | 4.58E-06 |
| 28725 | Spin2c | -0.63517 | 2.53E-07 |
| 29454 | Tbl3 | -0.63515 | 4.82E-13 |
| 13949 | Gm38456 | -0.63496 | 2.70E-07 |
| 19263 | Mad2l1bp | -0.6346 | 9.01E-06 |
| 2008 | Aimp2 | -0.63435 | 1.12E-11 |
| 19771 | Mif | -0.63413 | 1.91E-11 |
| 25935 | Rangap1 | -0.63386 | 2.26E-21 |
| 5022 | Cox16 | -0.63368 | 7.71E-05 |
| 22192 | Notum | -0.63348 | 3.64E-05 |
| 3999 | Ccne2 | -0.63257 | 3.71E-09 |
| 25680 | Ptpn7 | -0.63125 | 0.001056 |
| 27618 | Sh3rf1 | -0.63121 | 5.67E-20 |
| 28672 | Spats2l | -0.63037 | 1.75E-23 |
| 16416 | Grwd1 | -0.63019 | 2.42E-13 |
| 32200 | Wif1 | -0.62999 | 2.75E-20 |
| 1835 | Adgrb1 | -0.62992 | 8.40E-06 |
| 4822 | Cmtm8 | -0.62933 | 0.000428 |
| 32677 | Zfp566 | -0.62927 | 1.98E-06 |
| 24997 | Polr3g | -0.62912 | 9.27E-10 |
| 15939 | Gm9284 | -0.62911 | 2.15E-06 |
| 27290 | Sdc4 | -0.62896 | 3.38E-15 |
| 404 | 1810037I17Rik | -0.62895 | 7.40E-06 |
| 28491 | Snrpd1 | -0.62889 | 2.22E-09 |
| 4722 | Clec14a | -0.62843 | 2.45E-13 |
| 27794 | Slc16a13 | -0.6284 | 3.91E-08 |
| 25950 | Rapgef5 | -0.62825 | 7.42E-12 |
| 7796 | Gabrb2 | -0.62815 | 0.001258 |
| 2387 | Aplnr | -0.6278 | 4.93E-19 |
| 22152 | Noct | -0.62755 | 2.79E-13 |
| 4813 | Cmss1 | -0.62733 | 6.44E-09 |
| 21835 | ND6 | -0.6271 | 1.68E-25 |
| 30882 | Tspan2 | -0.62686 | 1.30E-06 |
| 17857 | Irf1 | -0.62632 | 6.14E-20 |
| 1914 | Aff1 | -0.62628 | 7.20E-15 |
| 30743 | Trim6 | -0.62621 | 0.015475 |
| 32056 | Vstm2b | -0.62621 | 9.50E-10 |
| 14207 | Gm45795 | -0.62595 | 0.00745 |
| 25462 | Prrx1 | -0.6258 | 1.71E-38 |
| 7115 | Fam162a | -0.62573 | 1.13E-12 |
| 8677 | Gm12655 | -0.62532 | 0.007451 |
| 27695 | Sinhcaf | -0.62507 | 3.71E-13 |
| 32854 | Zfp948 | -0.62498 | 2.95E-13 |
| 22123 | Nme2 | -0.62496 | 0.020175 |
| 18679 | Lcmt2 | -0.62486 | 0.000555 |
| 24936 | Podn | -0.62483 | 5.03E-11 |
| 26449 | Rnf180 | -0.6244 | 1.03E-06 |
| 29589 | Tek | -0.62411 | 5.23E-15 |
| 30223 | Tnfrsf11a | -0.62391 | 0.001424 |
| 25926 | Ranbp1 | -0.62344 | 2.82E-11 |
| 28541 | Socs1 | -0.62322 | 1.56E-05 |
| 528 | 2700038G22Rik | -0.62297 | 3.50E-07 |
| 30961 | Ttc9 | -0.62295 | 0.002157 |
| 30329 | Tor4a | -0.62214 | 0.002494 |
| 2829 | Atp6v0e2 | -0.62152 | 0.001297 |
| 19626 | Meis1 | -0.62117 | 5.14E-09 |
| 22657 | Optn | -0.62094 | 0.000201 |
| 18072 | Kcnab1 | -0.62077 | 0.010474 |
| 3916 | Ccdc74a | -0.61985 | 9.98E-05 |
| 21139 | Mpped2 | -0.61981 | 8.69E-17 |
| 30005 | Tmem176b | -0.61964 | 6.92E-14 |
| 22122 | Nme1 | -0.6192 | 2.50E-14 |
| 19201 | Lyar | -0.61915 | 1.43E-16 |
| 14800 | Gm5809 | -0.61889 | 4.06E-06 |
| 18645 | Lbh | -0.61865 | 2.01E-20 |
| 15041 | Gm6554 | -0.61829 | 0.000777 |
| 1496 | Aagab | -0.61768 | 4.37E-14 |
| 30035 | Tmem204 | -0.61736 | 1.17E-07 |
| 6985 | Exoc3l2 | -0.61732 | 2.07E-05 |
| 1605 | Acaa1b | -0.61621 | 3.38E-06 |
| 16140 | Gnb5 | -0.61618 | 8.60E-10 |
| 24971 | Polr1b | -0.6161 | 6.01E-15 |
| 25320 | Prkch | -0.616 | 2.23E-10 |
| 26339 | Rims3 | -0.61505 | 0.005019 |
| 25058 | Ppan | -0.61481 | 1.17E-15 |
| 18984 | Lpar6 | -0.6148 | 4.36E-09 |
| 4486 | Cgref1 | -0.61373 | 0.001311 |
| 9373 | Gm15550 | -0.61352 | 1.12E-11 |
| 27687 | Sik3 | -0.6135 | 1.44E-22 |
| 30801 | Trp53i11 | -0.61338 | 4.15E-18 |
| 21232 | Mrpl36 | -0.61306 | 1.99E-08 |
| 7611 | Foxk1 | -0.61286 | 7.67E-18 |
| 26144 | Relb | -0.61231 | 1.11E-08 |
| 21228 | Mrpl32 | -0.6119 | 1.21E-08 |
| 28125 | Slc8b1 | -0.61171 | 4.31E-06 |
| 30340 | Tpd52 | -0.61164 | 1.13E-06 |
| 29928 | Tmeff1 | -0.61163 | 1.67E-23 |
| 7020 | F11r | -0.61156 | 2.60E-11 |
| 21765 | Nat8f4 | -0.61139 | 0.005633 |
| 2622 | Arrdc3 | -0.61136 | 1.42E-26 |
| 5419 | Cyfip2 | -0.61127 | 4.68E-08 |
| 4503 | Chchd4 | -0.61114 | 2.75E-07 |
| 17606 | Igsf1 | -0.61113 | 2.32E-07 |
| 18646 | Lbhd1 | -0.61084 | 5.56E-05 |
| 29237 | Syt11 | -0.61081 | 2.50E-14 |
| 26997 | Rtn4rl1 | -0.61078 | 0.005216 |
| 15892 | Gm9104 | -0.61065 | 0.002736 |
| 32247 | Wwox | -0.61048 | 2.26E-08 |
| 29185 | Swsap1 | -0.60981 | 5.54E-06 |
| 292 | 1700066M21Rik | -0.60962 | 1.36E-08 |
| 2429 | App | -0.60956 | 2.73E-28 |
| 5781 | Ddx31 | -0.60946 | 3.82E-09 |
| 25018 | Pop7 | -0.60941 | 1.12E-05 |
| 32165 | Wdr86 | -0.60929 | 1.66E-16 |
| 24483 | Pgm1 | -0.60926 | 1.01E-11 |
| 26955 | Rsl1d1 | -0.60866 | 1.57E-20 |
| 27430 | Serinc5 | -0.60849 | 7.48E-18 |
| 25013 | Pon2 | -0.60847 | 1.54E-10 |
| 7950 | Gcsh | -0.60825 | 4.90E-12 |
| 2150 | Alyref2 | -0.60807 | 0.000995 |
| 1902 | Aebp1 | -0.60795 | 6.64E-19 |
| 6298 | Dtd1 | -0.60794 | 6.26E-07 |
| 26190 | Rfk | -0.60788 | 1.65E-16 |
| 21482 | Mustn1 | -0.60778 | 0.018938 |
| 25783 | R3hcc1l | -0.60774 | 1.97E-11 |
| 15652 | Gm8392 | -0.60747 | 0.000162 |
| 24973 | Polr1d | -0.60738 | 1.16E-12 |
| 27907 | Slc25a5 | -0.60697 | 1.74E-14 |
| 29499 | Tcf12 | -0.60693 | 2.02E-31 |
| 2083 | Aldh1l1 | -0.60646 | 0.002423 |
| 7866 | Gar1 | -0.60608 | 8.05E-13 |
| 5339 | Ctsz | -0.60606 | 5.42E-17 |
| 16443 | Gspt2 | -0.60586 | 6.09E-09 |
| 31388 | Utp18 | -0.60569 | 1.78E-14 |
| 16143 | Gng11 | -0.60546 | 9.60E-09 |
| 19755 | Micos10 | -0.6054 | 7.26E-13 |
| 29790 | Tifa | -0.60515 | 0.005704 |
| 5083 | Cpox | -0.60514 | 9.90E-12 |
| 30746 | Trim62 | -0.60499 | 3.52E-13 |
| 6763 | Enpp4 | -0.60464 | 8.44E-08 |
| 3287 | Bok | -0.60441 | 1.67E-08 |
| 30167 | Tmem98 | -0.60426 | 1.22E-12 |
| 24358 | Pdk4 | -0.60418 | 0.000293 |
| 22490 | Obi1 | -0.60309 | 2.90E-12 |
| 24461 | Pgam1 | -0.60303 | 9.44E-17 |
| 10045 | Gm18688 | -0.60258 | 0.01304 |
| 3159 | Bcl6b | -0.60229 | 4.20E-14 |
| 22652 | Oprd1 | -0.60202 | 0.012676 |
| 15303 | Gm7459 | -0.60188 | 6.48E-10 |
| 25505 | Prtg | -0.60178 | 0.000305 |
| 1576 | Abhd17b | -0.60168 | 2.74E-13 |
| 2096 | Aldh9a1 | -0.6015 | 2.69E-15 |
| 14766 | Gm5721 | -0.60123 | 1.72E-07 |
| 18306 | Klf6 | -0.60117 | 4.22E-26 |
| 28947 | St6galnac5 | -0.60106 | 2.10E-09 |
| 8952 | Gm13776 | -0.60106 | 1.85E-09 |
| 15137 | Gm6912 | -0.601 | 4.33E-06 |
| 28754 | Spns2 | -0.60048 | 2.23E-08 |
| 604 | 4732491K20Rik | -0.60045 | 0.023614 |
| 6585 | Eid2 | -0.60044 | 8.63E-11 |
| 28257 | Smim26 | -0.60042 | 0.004113 |
| 15692 | Gm8525 | -0.60037 | 0.00016 |
| 17981 | Jak2 | -0.60016 | 5.71E-22 |
| 3069 | BC003965 | -0.5999 | 8.43E-09 |
| 30344 | Tpgs2 | -0.59924 | 2.40E-13 |
| 29007 | Stim2 | -0.59906 | 5.52E-14 |
| 28887 | Srxn1 | -0.59895 | 2.22E-07 |
| 22228 | Npm3 | -0.59871 | 0.000131 |
| 32865 | Zfp958 | -0.59852 | 5.89E-05 |
| 27240 | Scly | -0.598 | 3.81E-09 |
| 27320 | Sec14l1 | -0.59797 | 7.52E-25 |
| 5141 | Crim1 | -0.59782 | 1.02E-15 |
| 26898 | Rps8-ps5 | -0.5976 | 0.00204 |
| 31060 | Txnrd1 | -0.59737 | 5.57E-26 |
| 25075 | Ppfibp1 | -0.59728 | 3.11E-20 |
| 21264 | Mrps18b | -0.59726 | 1.43E-06 |
| 25749 | Pxylp1 | -0.59701 | 7.50E-13 |
| 32320 | Yeats4 | -0.59681 | 2.19E-14 |
| 17146 | Hspd1 | -0.59681 | 2.38E-16 |
| 2914 | AW209491 | -0.59635 | 5.09E-06 |
| 17334 | Igfbp5 | -0.59622 | 1.32E-35 |
| 29792 | Tigar | -0.59622 | 1.72E-06 |
| 21826 | Ncs1 | -0.59575 | 5.91E-19 |
| 18701 | Ldha | -0.59571 | 4.38E-14 |
| 3813 | Ccdc12 | -0.59548 | 4.44E-05 |
| 3009 | Babam2 | -0.59541 | 4.75E-10 |
| 30797 | Trp53 | -0.59539 | 3.75E-18 |
| 18453 | Kremen2 | -0.59465 | 2.24E-08 |
| 15221 | Gm7192 | -0.59442 | 0.000139 |
| 27939 | Slc2a13 | -0.59425 | 7.00E-11 |
| 17052 | Hprt | -0.59409 | 1.19E-11 |
| 2903 | Aven | -0.59406 | 1.96E-06 |
| 7392 | Fdx1 | -0.59399 | 1.54E-09 |
| 18625 | Laptm4b | -0.59364 | 4.54E-10 |
| 18352 | Klhl5 | -0.59342 | 8.70E-16 |
| 2109 | Alg3 | -0.59326 | 8.69E-09 |
| 9892 | Gm18335 | -0.59312 | 5.84E-06 |
| 32969 | Znhit6 | -0.59295 | 9.38E-14 |
| 2029 | Akap12 | -0.5924 | 6.06E-25 |
| 1663 | Acot7 | -0.59193 | 2.63E-11 |
| 30078 | Tmem251 | -0.59151 | 5.71E-06 |
| 19552 | Mdfi | -0.59146 | 1.33E-12 |
| 21951 | Neo1 | -0.59135 | 3.23E-28 |
| 21718 | Naf1 | -0.5913 | 1.96E-08 |
| 15392 | Gm7694 | -0.59129 | 4.15E-05 |
| 25504 | Prss8 | -0.59128 | 0.017029 |
| 27372 | Sem1 | -0.59107 | 2.33E-14 |
| 7105 | Fam136a | -0.59097 | 1.13E-11 |
| 26715 | Rpl7-ps8 | -0.59094 | 3.70E-06 |
| 25817 | Rab27b | -0.59074 | 0.001454 |
| 18989 | Lpcat4 | -0.59051 | 1.18E-09 |
| 3279 | Bnip3 | -0.58983 | 1.49E-05 |
| 25692 | Ptprm | -0.58922 | 1.91E-16 |
| 6104 | Dnah7b | -0.58894 | 0.002864 |
| 22121 | Nmd3 | -0.58851 | 3.10E-12 |
| 3086 | BC034090 | -0.58845 | 5.37E-16 |
| 24607 | Pigw | -0.58842 | 0.001089 |
| 16835 | Helz2 | -0.58834 | 8.20E-13 |
| 5155 | Crlf2 | -0.58827 | 2.55E-05 |
| 29701 | Tfrc | -0.58789 | 1.07E-17 |
| 26965 | Rspo1 | -0.58772 | 2.49E-13 |
| 7619 | Foxn2 | -0.58762 | 3.16E-11 |
| 14572 | Gm5234 | -0.5873 | 0.000121 |
| 4060 | Cd200 | -0.58729 | 6.62E-18 |
| 12582 | Gm25432 | -0.58723 | 2.13E-11 |
| 32653 | Zfp51 | -0.58719 | 4.92E-06 |
| 5639 | D930048N14Rik | -0.58709 | 0.000533 |
| 17729 | Impa2 | -0.58698 | 1.40E-13 |
| 15151 | Gm6978 | -0.58695 | 1.56E-08 |
| 17767 | Inpp5a | -0.58692 | 6.65E-19 |
| 2753 | Atic | -0.58674 | 2.65E-22 |
| 8367 | Gm11425 | -0.58635 | 4.57E-05 |
| 26052 | Rbm46 | -0.58623 | 0.015999 |
| 21272 | Mrps26 | -0.58613 | 7.69E-08 |
| 2784 | Atp1b3 | -0.58587 | 1.07E-16 |
| 5778 | Ddx25 | -0.58579 | 8.38E-06 |
| 7544 | Fmnl1 | -0.58571 | 0.000162 |
| 31022 | Tulp1 | -0.58507 | 2.12E-06 |
| 2262 | Ankrd37 | -0.58494 | 0.008927 |
| 6226 | Dph5 | -0.58488 | 1.22E-06 |
| 3260 | Bmp3 | -0.58463 | 1.63E-10 |
| 3174 | Bdh2 | -0.58425 | 6.23E-05 |
| 16027 | Gm9583 | -0.58422 | 0.010994 |
| 9161 | Gm14706 | -0.58404 | 1.33E-07 |
| 2511 | Arhgap6 | -0.58364 | 6.40E-10 |
| 5023 | Cox17 | -0.58328 | 0.000564 |
| 17235 | Ier5 | -0.58267 | 3.55E-08 |
| 28803 | Spryd7 | -0.58264 | 1.65E-07 |
| 30314 | Top1mt | -0.58242 | 1.18E-07 |
| 22059 | Nkain1 | -0.58159 | 1.17E-10 |
| 3982 | Ccn3 | -0.58111 | 0.006866 |
| 19379 | Map3k20 | -0.58109 | 5.09E-17 |
| 30832 | Trpv2 | -0.58021 | 0.005656 |
| 5403 | Cyb5b | -0.57973 | 6.44E-20 |
| 6626 | Eif3j1 | -0.57944 | 4.20E-09 |
| 25393 | Prorsd1 | -0.57918 | 0.000124 |
| 15836 | Gm8942 | -0.57897 | 4.44E-15 |
| 24441 | Pf4 | -0.57861 | 0.011902 |
| 3636 | Camk2d | -0.57855 | 4.32E-16 |
| 32342 | Yrdc | -0.57833 | 1.51E-08 |
| 25925 | Ran | -0.5778 | 4.38E-17 |
| 29285 | Tacc2 | -0.57706 | 1.91E-19 |
| 9427 | Gm15793 | -0.57702 | 0.003793 |
| 9354 | Gm15464 | -0.57687 | 0.010505 |
| 7739 | Fxyd3 | -0.57687 | 0.020575 |
| 1509 | Aatf | -0.5768 | 1.10E-11 |
| 17110 | Hsdl1 | -0.57679 | 1.08E-09 |
| 21515 | Myct1 | -0.57677 | 0.00014 |
| 22427 | Nup37 | -0.57669 | 2.24E-10 |
| 21192 | Mrm2 | -0.57668 | 5.21E-05 |
| 21016 | Mllt11 | -0.57644 | 2.78E-06 |
| 22044 | Nip7 | -0.57617 | 7.42E-11 |
| 28540 | Sobp | -0.57595 | 2.22E-14 |
| 22472 | Oaf | -0.57592 | 2.02E-10 |
| 14261 | Gm46546 | -0.57505 | 4.02E-09 |
| 32559 | Zfp280b | -0.5749 | 9.23E-14 |
| 31393 | Utp4 | -0.57476 | 3.99E-14 |
| 6971 | Evpl | -0.57455 | 2.06E-06 |
| 5309 | Ctps | -0.57368 | 2.29E-15 |
| 8033 | Gimap4 | -0.57362 | 6.56E-08 |
| 26584 | Rpl17 | -0.57319 | 4.48E-08 |
| 28138 | Slc9b2 | -0.57316 | 0.007949 |
| 3197 | Bex4 | -0.5731 | 3.29E-06 |
| 28487 | Snrpa1 | -0.57246 | 3.12E-11 |
| 18979 | Lpar1 | -0.57219 | 5.05E-23 |
| 2075 | Alcam | -0.57216 | 2.43E-28 |
| 3449 | Bysl | -0.57203 | 1.54E-10 |
| 24332 | Pde7b | -0.57201 | 2.04E-15 |
| 32593 | Zfp36 | -0.5719 | 1.90E-18 |
| 32157 | Wdr74 | -0.57183 | 3.14E-10 |
| 18052 | Kazn | -0.57178 | 1.66E-19 |
| 27534 | Sf3b5 | -0.57144 | 4.27E-09 |
| 30897 | Tspoap1 | -0.5711 | 6.70E-05 |
| 22157 | Nol10 | -0.57086 | 8.64E-13 |
| 27083 | Samd10 | -0.57051 | 0.001567 |
| 16154 | Gngt2 | -0.56997 | 0.007364 |
| 24056 | Pald1 | -0.56986 | 2.00E-14 |
| 3892 | Ccdc40 | -0.56985 | 0.000495 |
| 13909 | Gm3739 | -0.56931 | 0.000324 |
| 32432 | Zc3h8 | -0.5692 | 4.10E-05 |
| 7667 | Frmpd3 | -0.56849 | 2.86E-05 |
| 23993 | P2rx7 | -0.56822 | 0.001709 |
| 8158 | Gm10131 | -0.56819 | 3.48E-15 |
| 15175 | Gm7049 | -0.56815 | 0.002291 |
| 7189 | Fam241b | -0.56806 | 0.000496 |
| 9534 | Gm16465 | -0.56785 | 1.11E-15 |
| 14939 | Gm6210 | -0.56774 | 9.79E-13 |
| 21564 | Myo19 | -0.56753 | 6.99E-15 |
| 32731 | Zfp660 | -0.56686 | 0.000156 |
| 18297 | Klf13 | -0.5668 | 9.62E-15 |
| 21703 | Naalad2 | -0.5663 | 2.34E-08 |
| 17135 | Hspa9 | -0.56582 | 2.95E-18 |
| 6986 | Exoc3l4 | -0.56534 | 0.000356 |
| 14082 | Gm4221 | -0.56528 | 0.016425 |
| 26438 | Rnf150 | -0.5651 | 3.33E-10 |
| 27672 | Siah2 | -0.56501 | 5.66E-10 |
| 26770 | Rps11-ps3 | -0.56457 | 0.007782 |
| 7076 | Fam104a | -0.56449 | 6.86E-08 |
| 24022 | Pacc1 | -0.56442 | 2.39E-08 |
| 1502 | Aard | -0.56427 | 0.00397 |
| 3440 | Btnl9 | -0.56414 | 0.005309 |
| 7302 | Fbxl7 | -0.5641 | 1.79E-16 |
| 15295 | Gm7436 | -0.56339 | 1.01E-08 |
| 6590 | Eif1a | -0.56324 | 1.53E-17 |
| 13863 | Gm3636 | -0.56305 | 0.007445 |
| 28982 | Stard8 | -0.56221 | 4.61E-07 |
| 21831 | ND3 | -0.56198 | 2.68E-12 |
| 24290 | Pcyox1l | -0.56175 | 1.07E-08 |
| 5498 | Cyp2j6 | -0.5616 | 2.93E-06 |
| 15265 | Gm7363 | -0.56154 | 0.000747 |
| 6636 | Eif4a3l1 | -0.56143 | 0.000429 |
| 8864 | Gm13421 | -0.56117 | 0.019889 |
| 8625 | Gm12435 | -0.56096 | 8.20E-18 |
| 24176 | Pcca | -0.56075 | 1.35E-12 |
| 27684 | Sigmar1 | -0.56072 | 8.07E-13 |
| 21737 | Nanp | -0.56049 | 0.00052 |
| 16526 | Gusb | -0.56033 | 3.01E-14 |
| 19472 | Max | -0.56028 | 1.28E-14 |
| 7758 | Fzd8 | -0.55975 | 9.33E-08 |
| 30291 | Tob1 | -0.55961 | 3.29E-11 |
| 25750 | Pycard | -0.55928 | 0.009785 |
| 16888 | Higd1a | -0.55886 | 4.77E-07 |
| 15550 | Gm8141 | -0.55875 | 0.004661 |
| 22174 | Nop2 | -0.55845 | 5.68E-13 |
| 3141 | Bcl10 | -0.55773 | 9.59E-13 |
| 21729 | Nalcn | -0.55757 | 2.84E-07 |
| 7878 | Gars | -0.55744 | 1.14E-15 |
| 29240 | Syt14 | -0.55742 | 5.46E-06 |
| 232 | 1700034H15Rik | -0.55724 | 0.00459 |
| 22149 | Noc2l | -0.55722 | 4.00E-23 |
| 22167 | Nolc1 | -0.55704 | 1.53E-15 |
| 26942 | Rrp9 | -0.55693 | 2.49E-10 |
| 23909 | Ormdl1 | -0.55679 | 1.19E-05 |
| 3898 | Ccdc51 | -0.55654 | 0.000147 |
| 28148 | Slco3a1 | -0.55649 | 4.18E-09 |
| 6183 | Dnph1 | -0.55645 | 3.51E-07 |
| 4851 | Cnnm1 | -0.5564 | 0.00047 |
| 15583 | Gm8225 | -0.55605 | 0.009325 |
| 4145 | Cd9 | -0.55598 | 2.69E-06 |
| 582 | 3110082I17Rik | -0.55592 | 2.27E-09 |
| 18993 | Lpin3 | -0.55565 | 0.012991 |
| 25724 | Pus7 | -0.55564 | 3.77E-14 |
| 17733 | Impdh2-ps | -0.55559 | 7.20E-16 |
| 3594 | Cacybp | -0.55557 | 8.97E-11 |
| 6652 | Eif5a | -0.55545 | 1.58E-14 |
| 16397 | Grk3 | -0.55527 | 5.97E-15 |
| 3223 | Bid | -0.55527 | 1.60E-07 |
| 31134 | Ube2q2 | -0.55513 | 6.44E-11 |
| 18573 | Kti12 | -0.55464 | 1.79E-09 |
| 7415 | Fez2 | -0.55461 | 1.79E-11 |
| 31193 | Uchl4 | -0.55454 | 1.30E-09 |
| 7505 | Fkbp1b | -0.55426 | 7.44E-05 |
| 31295 | Uqcc2 | -0.55396 | 7.87E-05 |
| 25198 | Praf2 | -0.55382 | 6.03E-09 |
| 14667 | Gm5425 | -0.55372 | 1.19E-08 |
| 27933 | Slc29a2 | -0.55354 | 2.96E-08 |
| 25705 | Ptrh2 | -0.5533 | 5.82E-10 |
| 26945 | Rsad1 | -0.55328 | 8.15E-06 |
| 28984 | Stat1 | -0.55322 | 1.40E-05 |
| 21782 | Nbl1 | -0.55304 | 3.97E-10 |
| 6957 | Etv5 | -0.55299 | 2.30E-17 |
| 6014 | Dkc1 | -0.55272 | 7.83E-16 |
| 30789 | Trmt61b | -0.55228 | 2.72E-05 |
| 10490 | Gm20769 | -0.55226 | 0.004849 |
| 25668 | Ptpn13 | -0.55217 | 1.15E-20 |
| 18355 | Klhl8 | -0.55209 | 7.94E-09 |
| 4267 | Cdkn2c | -0.55189 | 1.80E-08 |
| 21840 | Ndfip1 | -0.55172 | 1.24E-14 |
| 21568 | Myo1d | -0.55169 | 1.41E-09 |
| 14103 | Gm4287 | -0.55111 | 0.000196 |
| 6989 | Exoc6 | -0.55108 | 0.000198 |
| 24999 | Polr3h | -0.55107 | 1.47E-09 |
| 6580 | Ehhadh | -0.55089 | 0.002305 |
| 13807 | Gm3558 | -0.55052 | 0.000131 |
| 14228 | Gm46209 | -0.55045 | 8.02E-07 |
| 5738 | Dctpp1 | -0.55043 | 2.62E-08 |
| 6337 | Dusp28 | -0.5497 | 0.005489 |
| 1322 | 9530077C05Rik | -0.54969 | 0.005442 |
| 14601 | Gm5297 | -0.54967 | 9.75E-20 |
| 16873 | Hhat | -0.54925 | 1.38E-05 |
| 26351 | Riox2 | -0.54891 | 2.32E-06 |
| 4154 | Cdc14b | -0.54869 | 2.41E-15 |
| 4498 | Chchd1 | -0.54862 | 6.36E-09 |
| 25540 | Psma1 | -0.54844 | 6.39E-16 |
| 27581 | Sgsm1 | -0.5483 | 0.000122 |
| 15311 | Gm7492 | -0.54817 | 0.009195 |
| 17919 | Itgal | -0.54808 | 0.008955 |
| 27637 | She | -0.54791 | 3.90E-08 |
| 32128 | Wdr36 | -0.54788 | 9.54E-13 |
| 7078 | Fam107b | -0.54775 | 2.85E-14 |
| 15029 | Gm6510 | -0.54754 | 5.11E-05 |
| 29801 | Timm10 | -0.54705 | 7.31E-05 |
| 8319 | Gm11221 | -0.54694 | 7.05E-09 |
| 27384 | Sema4f | -0.54686 | 2.41E-07 |
| 31131 | Ube2n | -0.54663 | 9.32E-15 |
| 19204 | Lyl1 | -0.54656 | 0.001812 |
| 7737 | Fxyd1 | -0.54629 | 9.91E-08 |
| 27841 | Slc22a21 | -0.54616 | 4.60E-08 |
| 8121 | Glrx5 | -0.54608 | 1.13E-10 |
| 6692 | Elovl1 | -0.54591 | 6.10E-12 |
| 18303 | Klf3 | -0.54568 | 5.47E-14 |
| 17240 | Ifi203 | -0.54533 | 0.022003 |
| 28016 | Slc39a11 | -0.54527 | 5.53E-07 |
| 22649 | Opn3 | -0.54518 | 0.01213 |
| 1577 | Abhd17c | -0.54479 | 5.71E-12 |
| 26731 | Rpl7l1 | -0.5445 | 3.13E-11 |
| 29225 | Synm | -0.54444 | 0.000314 |
| 9500 | Gm16238 | -0.54421 | 0.007924 |
| 22281 | Nradd | -0.5441 | 5.32E-08 |
| 22023 | Nhlrc1 | -0.54389 | 0.009017 |
| 14793 | Gm5796 | -0.5436 | 0.013138 |
| 4758 | Clint1 | -0.54335 | 2.69E-24 |
| 5731 | Dctd | -0.54323 | 1.37E-16 |
| 29088 | Stxbp2 | -0.543 | 2.48E-11 |
| 17150 | Hspe1-rs1 | -0.54293 | 2.92E-14 |
| 5097 | Cpt2 | -0.54289 | 2.25E-07 |
| 25922 | Ramp1 | -0.54279 | 0.004787 |
| 9528 | Gm16433 | -0.54259 | 4.14E-14 |
| 7475 | Fhl1 | -0.54244 | 6.90E-20 |
| 15377 | Gm7653 | -0.54223 | 0.000119 |
| 24379 | Pdxp | -0.54197 | 0.000373 |
| 17188 | Hyi | -0.54156 | 1.83E-07 |
| 29778 | Thumpd2 | -0.54057 | 1.80E-07 |
| 13898 | Gm3696 | -0.54046 | 4.98E-05 |
| 3712 | Carnmt1 | -0.54039 | 7.47E-08 |
| 16225 | Gpcpd1 | -0.54027 | 1.04E-18 |
| 28342 | Snhg16 | -0.54027 | 5.04E-07 |
| 6416 | E230016M11Rik | -0.54009 | 0.002662 |
| 29677 | Tfap2a | -0.53988 | 7.04E-05 |
| 16971 | Hnrnpf | -0.53973 | 5.62E-23 |
| 28847 | Srm | -0.53971 | 1.50E-10 |
| 28896 | Ssbp4 | -0.53962 | 1.30E-09 |
| 5392 | Cxcr4 | -0.5396 | 1.72E-05 |
| 4917 | Col12a1 | -0.53948 | 6.37E-20 |
| 25077 | Pphln1 | -0.53937 | 8.17E-17 |
| 3732 | Casp8 | -0.53934 | 6.01E-11 |
| 24707 | Pkp1 | -0.5393 | 3.98E-10 |
| 18753 | Lgr5 | -0.53918 | 8.89E-24 |
| 29418 | Tbc1d16 | -0.53916 | 7.87E-19 |
| 7434 | Fgf13 | -0.53911 | 6.91E-11 |
| 26577 | Rpl13a-ps1 | -0.53906 | 0.000223 |
| 6037 | Dlk2 | -0.53859 | 3.03E-05 |
| 3288 | Bola1 | -0.53828 | 0.000662 |
| 29042 | Stom | -0.53822 | 7.17E-09 |
| 25960 | Rars | -0.53782 | 1.75E-16 |
| 25829 | Rab36 | -0.5375 | 9.83E-11 |
| 8520 | Gm12020 | -0.53739 | 2.62E-17 |
| 30032 | Tmem201 | -0.53735 | 7.91E-21 |
| 18333 | Klhl23 | -0.5373 | 5.30E-15 |
| 28701 | Spef1 | -0.53711 | 4.25E-08 |
| 15103 | Gm6764 | -0.53701 | 2.32E-05 |
| 2809 | Atp5j2 | -0.53699 | 2.78E-06 |
| 6321 | Dusp1 | -0.53675 | 2.61E-15 |
| 3557 | Cabp1 | -0.53669 | 0.011281 |
| 5771 | Ddx18 | -0.53634 | 8.09E-07 |
| 18022 | Kank1 | -0.53624 | 3.43E-08 |
| 6224 | Dph2 | -0.5362 | 1.22E-05 |
| 8021 | Ghitm | -0.53617 | 3.08E-17 |
| 3426 | Btg2 | -0.53607 | 3.31E-19 |
| 10440 | Gm20426 | -0.53604 | 0.018891 |
| 14688 | Gm5492 | -0.5359 | 9.72E-13 |
| 21812 | Ncl | -0.53543 | 6.39E-26 |
| 5643 | Dab2 | -0.53507 | 7.01E-22 |
| 29757 | Thnsl1 | -0.53503 | 0.001309 |
| 5253 | Cst3 | -0.53503 | 2.21E-11 |
| 27824 | Slc1a5 | -0.53497 | 2.42E-10 |
| 16481 | Gtf2f2 | -0.53469 | 6.87E-09 |
| 32158 | Wdr75 | -0.53459 | 1.66E-13 |
| 6121 | Dnajb2 | -0.53435 | 4.68E-12 |
| 14789 | Gm5786 | -0.53418 | 0.001717 |
| 22177 | Nop58 | -0.53416 | 1.57E-19 |
| 32498 | Zfp105 | -0.53412 | 4.68E-08 |
| 25923 | Ramp2 | -0.53407 | 1.45E-07 |
| 15963 | Gm9354 | -0.53387 | 0.001891 |
| 6551 | Efna5 | -0.53376 | 5.23E-17 |
| 17853 | Irak2 | -0.53373 | 0.001326 |
| 3040 | Basp1 | -0.53344 | 4.88E-14 |
| 26238 | Rgs7bp | -0.53294 | 0.000935 |
| 21357 | Mtap | -0.53277 | 5.40E-16 |
| 17868 | Irf9 | -0.53273 | 5.68E-13 |
| 30202 | Tmtc1 | -0.53247 | 1.93E-07 |
| 17974 | Jade1 | -0.53229 | 1.84E-09 |
| 24545 | Phlda3 | -0.53227 | 6.77E-08 |
| 5420 | Cygb | -0.53215 | 7.68E-08 |
| 31045 | Txn1 | -0.53125 | 6.24E-08 |
| 22572 | Ogfrl1 | -0.53107 | 9.78E-12 |
| 27010 | Rufy3 | -0.5308 | 6.18E-16 |
| 3423 | Btg1 | -0.53069 | 6.52E-14 |
| 8582 | Gm12286 | -0.53056 | 0.000528 |
| 22173 | Nop16 | -0.52979 | 5.60E-09 |
| 19250 | Mab21l2 | -0.52934 | 1.19E-17 |
| 4260 | Cdkn1a | -0.52926 | 2.12E-06 |
| 5012 | Coro2b | -0.52916 | 1.36E-09 |
| 18105 | Kcnip3 | -0.52913 | 9.15E-09 |
| 6627 | Eif3j2 | -0.52904 | 6.64E-14 |
| 21336 | Msr1 | -0.52897 | 0.005809 |
| 32228 | Wnt7b | -0.52893 | 9.37E-05 |
| 2341 | Ap1s2 | -0.52881 | 3.02E-08 |
| 30905 | Tsr1 | -0.52874 | 3.28E-11 |
| 17449 | Igip | -0.5285 | 2.92E-05 |
| 19738 | Mgmt | -0.5283 | 0.000308 |
| 27505 | Sesn2 | -0.5282 | 1.72E-07 |
| 21490 | Mx2 | -0.528 | 0.008755 |
| 6345 | Dusp9 | -0.52797 | 6.06E-07 |
| 6140 | Dnajc2 | -0.52785 | 0.000325 |
| 3619 | Calm2 | -0.52705 | 2.90E-12 |
| 5112 | Cradd | -0.52696 | 0.002668 |
| 3488 | C1qtnf7 | -0.52692 | 3.42E-09 |
| 25965 | Rasa4 | -0.52671 | 4.30E-08 |
| 1167 | 5730416F02Rik | -0.52667 | 0.004927 |
| 2303 | Anp32b | -0.5265 | 3.56E-15 |
| 25842 | Rab40b | -0.52578 | 0.000869 |
| 24434 | Pex2 | -0.52574 | 8.95E-09 |
| 29655 | Tex30 | -0.52574 | 0.000459 |
| 26518 | Robo3 | -0.52564 | 0.003181 |
| 24881 | Plxnd1 | -0.52534 | 3.98E-21 |
| 18151 | Kcnq4 | -0.52532 | 3.11E-07 |
| 28200 | Smagp | -0.52524 | 0.022041 |
| 7508 | Fkbp4 | -0.52482 | 1.18E-15 |
| 27664 | Shroom1 | -0.52462 | 0.001414 |
| 2032 | Akap17b | -0.52457 | 6.83E-06 |
| 8428 | Gm11675 | -0.52443 | 3.72E-09 |
| 27960 | Slc31a2 | -0.52413 | 4.36E-07 |
| 2453 | Arap3 | -0.52383 | 5.73E-14 |
| 18788 | Limd2 | -0.52358 | 2.43E-13 |
| 26904 | Rpsa-ps12 | -0.52356 | 2.10E-11 |
| 2743 | Atg3 | -0.52356 | 3.11E-11 |
| 3237 | Blmh | -0.52325 | 5.46E-15 |
| 25547 | Psma7 | -0.52317 | 5.93E-11 |
| 24013 | Pa2g4 | -0.52316 | 3.77E-14 |
| 14529 | Gm5124 | -0.52316 | 1.23E-12 |
| 24985 | Polr2h | -0.52315 | 3.30E-06 |
| 30354 | Tpm4 | -0.52314 | 1.03E-18 |
| 5296 | Ctla2a | -0.52311 | 0.015086 |
| 17977 | Jag1 | -0.52301 | 2.15E-20 |
| 2006 | Aim2 | -0.52278 | 0.013198 |
| 8391 | Gm11516 | -0.52265 | 9.12E-13 |
| 2810 | Atp5k | -0.52243 | 0.007669 |
| 21899 | Ndufs6 | -0.52238 | 0.000739 |
| 4271 | Cdo1 | -0.52229 | 2.31E-14 |
| 14917 | Gm6157 | -0.52223 | 2.19E-05 |
| 19027 | Lrp1b | -0.52216 | 0.02051 |
| 21278 | Mrps34 | -0.52171 | 0.002203 |
| 25093 | Ppl | -0.52163 | 1.20E-05 |
| 30721 | Trim36 | -0.52143 | 0.004287 |
| 5603 | D430019H16Rik | -0.52127 | 1.47E-16 |
| 7390 | Fdft1 | -0.52121 | 2.15E-11 |
| 26938 | Rrp1b | -0.52108 | 6.86E-16 |
| 16117 | Gmps | -0.52094 | 8.78E-16 |
| 5375 | Cxadr | -0.52068 | 1.06E-11 |
| 8629 | Gm12444 | -0.52034 | 1.38E-05 |
| 3123 | Bcap29 | -0.52026 | 1.50E-05 |
| 25067 | Ppcs | -0.5201 | 0.003207 |
| 25508 | Prune2 | -0.51978 | 0.009107 |
| 27376 | Sema3d | -0.51941 | 2.07E-14 |
| 25056 | Ppa1 | -0.51941 | 1.70E-11 |
| 5081 | Cpne8 | -0.51918 | 2.76E-09 |
| 28524 | Snx25 | -0.51915 | 3.30E-08 |
| 1880 | Adora2b | -0.51906 | 0.004739 |
| 1482 | Aacs | -0.519 | 3.54E-10 |
| 17204 | Ica1 | -0.51892 | 1.50E-05 |
| 3767 | Cbfb | -0.51886 | 3.59E-22 |
| 15211 | Gm7155 | -0.51864 | 0.016287 |
| 19752 | Mical3 | -0.51854 | 4.57E-15 |
| 29060 | Strbp | -0.51768 | 1.04E-15 |
| 25282 | Prelid3b | -0.51707 | 1.00E-11 |
| 30093 | Tmem267 | -0.51701 | 2.87E-07 |
| 2608 | Arpc1b | -0.51692 | 4.81E-07 |
| 32088 | Wasf1 | -0.51653 | 2.87E-10 |
| 1918 | Afg1l | -0.51644 | 0.002624 |
| 22171 | Nop10 | -0.51603 | 8.20E-07 |
| 32299 | Xrcc4 | -0.51601 | 0.000945 |
| 1830 | Add3 | -0.51578 | 6.91E-20 |
| 24093 | Pard3 | -0.51568 | 2.08E-19 |
| 27670 | Siah1a | -0.51556 | 5.69E-10 |
| 21687 | N4bp3 | -0.51542 | 2.91E-06 |
| 13330 | Gm2950 | -0.51518 | 0.000711 |
| 6608 | Eif2b2 | -0.51513 | 1.52E-09 |
| 8035 | Gimap6 | -0.51507 | 1.34E-08 |
| 32456 | Zdhhc12 | -0.51506 | 1.19E-05 |
| 2305 | Anpep | -0.51453 | 2.76E-13 |
| 25099 | Ppm1g | -0.51451 | 1.70E-17 |
| 6515 | Eef1d | -0.51447 | 2.22E-11 |
| 17131 | Hspa4 | -0.51438 | 1.89E-21 |
| 26750 | Rpp25l | -0.51405 | 6.20E-05 |
| 2434 | Aprt | -0.51401 | 0.001069 |
| 30787 | Trmt6 | -0.51399 | 2.91E-12 |
| 21380 | Mthfd1 | -0.51378 | 7.28E-16 |
| 5925 | Desi2 | -0.51371 | 1.24E-13 |
| 29443 | Tbcc | -0.51369 | 1.50E-05 |
| 3345 | Brix1 | -0.5135 | 1.66E-12 |
| 26516 | Robo1 | -0.51336 | 3.71E-17 |
| 8210 | Gm10358 | -0.51315 | 6.85E-06 |
| 6862 | Erdr1 | -0.51311 | 8.17E-10 |
| 24444 | Pfdn2 | -0.51311 | 1.24E-10 |
| 15334 | Gm7537 | -0.51305 | 0.000301 |
| 1897 | Adrm1b | -0.51301 | 1.05E-10 |
| 5656 | Dancr | -0.51266 | 0.00096 |
| 21805 | Nck1 | -0.51245 | 9.76E-10 |
| 17100 | Hsd17b7 | -0.51229 | 3.19E-13 |
| 18639 | Lat | -0.51216 | 0.010308 |
| 29016 | Stk17b | -0.51205 | 8.25E-07 |
| 26743 | Rplp1 | -0.51188 | 1.27E-09 |
| 27046 | S100a14 | -0.51183 | 0.012856 |
| 4037 | Cct3-ps1 | -0.51176 | 5.19E-13 |
| 10452 | Gm20587 | -0.51175 | 8.96E-07 |
| 7825 | Gale | -0.51171 | 1.25E-06 |
| 16366 | Grhl3 | -0.51168 | 0.002525 |
| 6463 | Ebna1bp2 | -0.51166 | 3.92E-13 |
| 10216 | Gm19052 | -0.51162 | 0.003391 |
| 4397 | Cerox1 | -0.51154 | 0.003205 |
| 21209 | Mrpl12 | -0.51145 | 3.03E-07 |
| 24643 | Pinx1 | -0.5114 | 1.83E-07 |
| 3139 | Bckdhb | -0.51139 | 5.05E-09 |
| 5959 | Dhrs13 | -0.51116 | 2.07E-05 |
| 30903 | Tspyl4 | -0.51112 | 4.64E-07 |
| 6675 | Elk3 | -0.51105 | 2.87E-16 |
| 6960 | Eva1a | -0.51078 | 0.000233 |
| 15724 | Gm8623 | -0.50968 | 8.17E-06 |
| 30201 | Tmsb4x | -0.50961 | 1.76E-13 |
| 28132 | Slc9a5 | -0.50959 | 9.95E-13 |
| 6320 | Dus4l | -0.50953 | 0.000138 |
| 30455 | Tram1 | -0.50938 | 1.26E-13 |
| 25600 | Psrc1 | -0.50912 | 1.31E-09 |
| 18978 | Loxl4 | -0.50897 | 0.002298 |
| 9624 | Gm17230 | -0.50895 | 9.96E-07 |
| 9678 | Gm17837 | -0.50894 | 1.36E-12 |
| 1481 | Aaas | -0.50884 | 3.82E-13 |
| 4635 | Cisd2 | -0.50852 | 3.78E-10 |
| 16254 | Gpr137c | -0.5085 | 1.28E-05 |
| 16819 | Hebp1 | -0.50809 | 4.46E-06 |
| 3822 | Ccdc126 | -0.50801 | 0.000275 |
| 17142 | Hspb8 | -0.50796 | 4.55E-05 |
| 28559 | Sorbs3 | -0.5077 | 2.02E-11 |
| 26811 | Rps16 | -0.5077 | 5.77E-07 |
| 27474 | Serpinb6b | -0.50672 | 0.004638 |
| 25120 | Ppp1r14b | -0.50672 | 2.51E-06 |
| 21847 | Ndrg2 | -0.50669 | 1.72E-11 |
| 3595 | Cad | -0.50612 | 1.68E-16 |
| 19468 | Matn4 | -0.50596 | 8.98E-07 |
| 17371 | Ighm | -0.50578 | 0.018549 |
| 23976 | Oxct1 | -0.50561 | 1.65E-19 |
| 21873 | Ndufaf4 | -0.50541 | 6.21E-06 |
| 4190 | Cdca7l | -0.50539 | 1.59E-08 |
| 21878 | Ndufb1 | -0.50537 | 1.48E-05 |
| 2813 | Atp5md | -0.50526 | 0.020951 |
| 26696 | Rpl36a-ps1 | -0.50525 | 1.71E-08 |
| 28108 | Slc7a11 | -0.50509 | 1.29E-06 |
| 7649 | Frat1 | -0.505 | 0.014731 |
| 19071 | Lrrc42 | -0.50497 | 2.18E-12 |
| 27790 | Slc16a1 | -0.50482 | 9.66E-12 |
| 25986 | Rasl2-9 | -0.50478 | 8.28E-08 |
| 26432 | Rnf144a | -0.50468 | 2.83E-19 |
| 22435 | Nup93 | -0.50444 | 3.94E-15 |
| 29682 | Tfap4 | -0.50433 | 6.52E-10 |
| 21193 | Mrm3 | -0.5043 | 3.53E-05 |
| 2740 | Atg16l2 | -0.50426 | 7.18E-07 |
| 21320 | Msh3 | -0.50422 | 1.19E-11 |
| 3255 | Bmp1 | -0.50417 | 5.68E-19 |
| 4831 | Cndp2 | -0.50414 | 7.57E-08 |
| 28126 | Slc9a1 | -0.50414 | 3.16E-15 |
| 16219 | Gpc1 | -0.50409 | 9.07E-13 |
| 24171 | Pcbd2 | -0.50385 | 0.004127 |
| 3143 | Bcl11b | -0.5037 | 5.62E-14 |
| 2293 | Ano10 | -0.50363 | 9.39E-11 |
| 6340 | Dusp4 | -0.50351 | 0.000172 |
| 14301 | Gm4742 | -0.50346 | 6.08E-06 |
| 29459 | Tbrg1 | -0.50314 | 4.78E-13 |
| 8691 | Gm12700 | -0.50301 | 3.01E-05 |
| 4829 | Cnbp | -0.50293 | 4.34E-15 |
| 5713 | Dclk1 | -0.50266 | 7.96E-12 |
| 29309 | Taf4b | -0.50261 | 5.25E-05 |
| 4261 | Cdkn1b | -0.50261 | 3.93E-16 |
| 8563 | Gm12184 | -0.50258 | 1.24E-12 |
| 21029 | Mmachc | -0.50224 | 5.18E-07 |
| 24731 | Pla2g6 | -0.5018 | 1.57E-08 |
| 25671 | Ptpn2 | -0.50152 | 8.28E-12 |
| 26645 | Rpl26 | -0.50131 | 9.81E-06 |
| 26186 | Rfc4 | -0.50122 | 5.22E-07 |
| 2315 | Anxa3 | -0.50111 | 3.19E-06 |
| 4036 | Cct3 | -0.50094 | 1.45E-12 |
| 24520 | Phf19 | -0.50087 | 2.41E-05 |
| 2026 | Akap1 | -0.50083 | 7.34E-14 |
| 7540 | Flywch2 | -0.50078 | 0.000102 |
| 32137 | Wdr46 | -0.50072 | 4.83E-11 |
| 2790 | Atp2b1 | -0.50051 | 1.69E-15 |
| 3769 | Cblb | -0.50044 | 4.54E-16 |
| 5198 | Crym | -0.50021 | 0.002912 |
| 6864 | Erf | -0.50018 | 4.73E-15 |
| 21129 | Mplkip | -0.50012 | 3.89E-07 |
| 31447 | Vegfc | -0.50011 | 3.30E-08 |

**Table4. Upregulated DEGs of buccal part of tooth germs at cap stage than of lingual part**

|  | SYMBOL | log2FoldChange | pvalue |
| --- | --- | --- | --- |
| 22142 | Nmur2 | 5.555321 | 4.64E-16 |
| 6810 | Epha6 | 5.12696 | 6.03E-47 |
| 29464 | Tbx18 | 4.832393 | 0 |
| 24151 | Pax7 | 4.820547 | 9.37E-24 |
| 18213 | Kera | 4.78053 | 0 |
| 4842 | Cnih3 | 4.748764 | 2.19E-09 |
| 27842 | Slc22a22 | 4.728928 | 2.63E-07 |
| 16378 | Grik3 | 4.649839 | 1.27E-293 |
| 30684 | Trhr | 4.593352 | 1.52E-17 |
| 32925 | Zic1 | 4.584592 | 2.45E-19 |
| 6735 | Emx2 | 4.538897 | 9.74E-14 |
| 21520 | Myf5 | 4.531565 | 1.49E-13 |
| 14000 | Gm3985 | 4.525616 | 7.10E-12 |
| 21586 | Myod1 | 4.513904 | 7.99E-79 |
| 27690 | Sim1 | 4.451685 | 2.28E-32 |
| 379 | 1700125H03Rik | 4.424948 | 6.57E-22 |
| 4206 | Cdh20 | 4.416631 | 9.21E-43 |
| 5634 | D930007P13Rik | 4.369777 | 1.97E-09 |
| 2236 | Ankrd1 | 4.238884 | 2.56E-07 |
| 941 | 4930565N06Rik | 4.155938 | 3.43E-22 |
| 22144 | Nnmt | 4.142853 | 4.61E-11 |
| 28084 | Slc6a1 | 4.129714 | 3.94E-19 |
| 18803 | Lincmd1 | 4.105161 | 6.05E-16 |
| 2073 | Alb | 4.024653 | 9.29E-19 |
| 2908 | Avpr1a | 3.999608 | 3.89E-63 |
| 21530 | Myh3 | 3.984947 | 8.65E-224 |
| 21523 | Myh1 | 3.979295 | 3.07E-16 |
| 1703 | Actc1 | 3.952498 | 0 |
| 24678 | Pitx3 | 3.745208 | 5.99E-27 |
| 3209 | Bhlhe22 | 3.731317 | 3.80E-48 |
| 4919 | Col14a1 | 3.720538 | 0 |
| 193 | 1700027H10Rik | 3.712805 | 1.27E-06 |
| 24179 | Pcdh10 | 3.70687 | 0 |
| 7615 | Foxl2os | 3.70627 | 1.16E-19 |
| 27244 | Scml4 | 3.692248 | 1.30E-35 |
| 25043 | Pou3f2 | 3.683788 | 3.33E-38 |
| 30368 | Tpsab1 | 3.676054 | 1.61E-07 |
| 7614 | Foxl2 | 3.66857 | 1.83E-09 |
| 17028 | Hoxc5 | 3.646338 | 0.000257 |
| 29503 | Tcf21 | 3.62774 | 2.74E-06 |
| 21533 | Myh7 | 3.626314 | 5.82E-09 |
| 21555 | Mymk | 3.614882 | 9.94E-18 |
| 3758 | Cav3 | 3.611261 | 1.19E-10 |
| 21553 | Mylk4 | 3.611155 | 6.96E-46 |
| 21556 | Mymx | 3.605477 | 4.87E-09 |
| 4586 | Chrng | 3.593952 | 4.56E-38 |
| 21529 | Myh2 | 3.562602 | 1.75E-12 |
| 27648 | Shisa9 | 3.540259 | 2.66E-62 |
| 21506 | Mybph | 3.537482 | 1.55E-18 |
| 31454 | Vgll2 | 3.515594 | 1.44E-08 |
| 27256 | Scn7a | 3.505698 | 2.68E-07 |
| 30983 | Ttn | 3.483335 | 9.47E-206 |
| 18068 | Kcna4 | 3.464788 | 6.74E-89 |
| 9579 | Gm16863 | 3.445167 | 2.51E-11 |
| 21503 | Mybpc1 | 3.381102 | 1.30E-63 |
| 24496 | Phactr1 | 3.37171 | 2.45E-267 |
| 17695 | Il31ra | 3.356587 | 4.12E-06 |
| 7925 | Gbx2 | 3.356569 | 0.000161 |
| 21588 | Myog | 3.356256 | 3.33E-112 |
| 3604 | Calb2 | 3.343629 | 1.70E-08 |
| 8071 | Gjd4 | 3.304631 | 0.001395 |
| 2640 | Arx | 3.30044 | 0.001356 |
| 17335 | Igfbp6 | 3.296086 | 1.17E-28 |
| 691 | 4930431F12Rik | 3.295753 | 9.44E-13 |
| 1858 | Adgrv1 | 3.253077 | 4.27E-62 |
| 14505 | Gm5084 | 3.245615 | 5.10E-34 |
| 28172 | Slitrk1 | 3.2406 | 3.90E-07 |
| 5248 | Csrp3 | 3.238569 | 0.001822 |
| 24663 | Pirt | 3.229823 | 2.11E-17 |
| 3190 | Best3 | 3.22838 | 0.000358 |
| 30739 | Trim55 | 3.207685 | 1.99E-16 |
| 3600 | Cadps | 3.198102 | 6.96E-43 |
| 5430 | Cyp19a1 | 3.177081 | 9.24E-05 |
| 21528 | Myh15 | 3.172073 | 0.000479 |
| 25072 | Ppfia2 | 3.17194 | 2.60E-177 |
| 16708 | Habp2 | 3.159957 | 9.83E-07 |
| 27545 | Sfrp5 | 3.153978 | 6.89E-37 |
| 21593 | Myot | 3.138385 | 7.53E-06 |
| 18103 | Kcnip1 | 3.137597 | 4.91E-18 |
| 22085 | Nkx3-2 | 3.134267 | 0.000626 |
| 25954 | Rapsn | 3.128369 | 4.46E-24 |
| 22270 | Nr2f1 | 3.116114 | 0 |
| 32055 | Vstm2a | 3.112882 | 1.18E-177 |
| 28625 | Spag17 | 3.093408 | 4.43E-18 |
| 2297 | Ano5 | 3.090625 | 4.07E-16 |
| 4570 | Chrna1 | 3.08551 | 1.08E-31 |
| 30331 | Tox2 | 3.085169 | 2.38E-119 |
| 23986 | Oxtr | 3.077116 | 1.46E-36 |
| 1526 | Abca8a | 3.063001 | 1.25E-106 |
| 18089 | Kcne4 | 3.041747 | 1.01E-149 |
| 4214 | Cdh6 | 3.023952 | 5.41E-283 |
| 30683 | Trhde | 3.020834 | 1.85E-24 |
| 21907 | Neb | 3.014128 | 5.72E-119 |
| 14128 | Gm43522 | 3.009179 | 0.000301 |
| 29228 | Synpo2l | 3.000652 | 6.77E-43 |
| 21182 | Mrgprf | 2.985862 | 3.30E-69 |
| 4951 | Col6a6 | 2.979179 | 2.94E-213 |
| 3584 | Cacng1 | 2.975821 | 0.000334 |
| 21531 | Myh4 | 2.975123 | 2.67E-05 |
| 21535 | Myh8 | 2.971643 | 3.85E-30 |
| 7496 | Fitm1 | 2.943247 | 2.49E-07 |
| 17070 | Hrk | 2.939941 | 2.34E-13 |
| 4884 | Cntn5 | 2.939183 | 2.04E-31 |
| 5051 | Cpa3 | 2.93791 | 3.23E-58 |
| 5007 | Corin | 2.933937 | 5.31E-259 |
| 2942 | B130024G19Rik | 2.930165 | 9.48E-101 |
| 4584 | Chrnd | 2.926234 | 3.56E-18 |
| 2616 | Arpp21 | 2.907415 | 1.78E-24 |
| 7385 | Fcrl6 | 2.891661 | 0.00206 |
| 7802 | Gabrg3 | 2.889711 | 4.88E-16 |
| 4200 | Cdh15 | 2.887255 | 4.82E-59 |
| 7117 | Fam163a | 2.875907 | 5.71E-06 |
| 4567 | Chrm3 | 2.873645 | 1.83E-08 |
| 5642 | Dab1 | 2.871514 | 5.04E-135 |
| 28180 | Sln | 2.866048 | 0.002469 |
| 31449 | Veph1 | 2.858553 | 1.08E-31 |
| 21552 | Mylk3 | 2.854574 | 3.05E-60 |
| 30273 | Tnnt2 | 2.853043 | 5.63E-33 |
| 11619 | Gm23608 | 2.847602 | 0.000748 |
| 28150 | Slco4c1 | 2.823334 | 0.002892 |
| 3736 | Casq2 | 2.814412 | 4.90E-23 |
| 30025 | Tmem196 | 2.814012 | 2.78E-12 |
| 6734 | Emx1 | 2.812406 | 0.000871 |
| 5077 | Cpne4 | 2.800579 | 6.27E-08 |
| 28609 | Sp9 | 2.794628 | 2.02E-43 |
| 26388 | Rnase10 | 2.788556 | 0.000113 |
| 1716 | Actn2 | 2.786963 | 6.52E-38 |
| 3484 | C1qtnf3 | 2.781557 | 0 |
| 16909 | Hjv | 2.770737 | 1.97E-09 |
| 29288 | Tacr1 | 2.761552 | 2.15E-20 |
| 24295 | Pdc | 2.750065 | 0.001278 |
| 6972 | Evx1 | 2.743855 | 0.013558 |
| 13350 | Gm29683 | 2.73226 | 0.000165 |
| 4798 | Clvs1 | 2.724338 | 1.54E-08 |
| 1700 | Acta2 | 2.715632 | 8.25E-257 |
| 19123 | Lrrtm4 | 2.714269 | 1.01E-66 |
| 4109 | Cd3g | 2.707627 | 0.001805 |
| 18341 | Klhl31 | 2.705152 | 7.71E-05 |
| 30272 | Tnnt1 | 2.704639 | 6.35E-138 |
| 21595 | Myoz2 | 2.697327 | 0.001802 |
| 18897 | LOC102637806 | 2.694872 | 7.19E-05 |
| 2469 | Arfgef3 | 2.690661 | 1.88E-45 |
| 5938 | Dgkb | 2.686518 | 1.93E-51 |
| 486 | 2310065F04Rik | 2.680146 | 0.015261 |
| 6973 | Evx1os | 2.677674 | 0.015358 |
| 17039 | Hoxd8 | 2.66511 | 9.55E-09 |
| 30369 | Tpsb2 | 2.659243 | 0.000906 |
| 22630 | Onecut1 | 2.645029 | 0.006202 |
| 17889 | Isl1 | 2.642943 | 6.27E-28 |
| 17696 | Il33 | 2.63993 | 1.15E-07 |
| 1430 | A730049H05Rik | 2.639667 | 3.10E-09 |
| 18843 | Lmcd1 | 2.635523 | 2.85E-194 |
| 5115 | Crb1 | 2.634958 | 0.000927 |
| 32928 | Zic4 | 2.63059 | 0.006497 |
| 32079 | Vwde | 2.630332 | 5.81E-07 |
| 24695 | Pkhd1l1 | 2.629859 | 2.69E-10 |
| 22359 | Ntn1 | 2.616343 | 9.63E-211 |
| 2116 | Alkal2 | 2.609659 | 6.32E-10 |
| 6849 | Erbb4 | 2.602532 | 1.42E-21 |
| 17066 | Hrh1 | 2.601078 | 7.78E-05 |
| 3301 | Bpifa1 | 2.599844 | 0.020212 |
| 1208 | 6430584L05Rik | 2.593376 | 0.020281 |
| 28151 | Slco5a1 | 2.590212 | 3.08E-122 |
| 22822 | Or13j1 | 2.582976 | 0.021278 |
| 5954 | Dhh | 2.578565 | 1.47E-05 |
| 30738 | Trim54 | 2.5749 | 0.008616 |
| 6694 | Elovl3 | 2.574484 | 0.001289 |
| 28312 | Smyd1 | 2.568907 | 5.88E-24 |
| 27183 | Scel | 2.567164 | 5.28E-25 |
| 21006 | Mlc1 | 2.56389 | 0.008665 |
| 25044 | Pou3f3 | 2.56158 | 8.05E-289 |
| 32669 | Zfp536 | 2.556537 | 2.50E-139 |
| 6736 | Emx2os | 2.547783 | 0.000103 |
| 4784 | Clrn1 | 2.545896 | 1.29E-34 |
| 16799 | Hdc | 2.539287 | 1.69E-32 |
| 13710 | Gm34354 | 2.527151 | 1.93E-20 |
| 21317 | Msc | 2.520888 | 3.95E-20 |
| 12697 | Gm25630 | 2.513127 | 2.52E-06 |
| 17942 | Itk | 2.500444 | 0.001967 |
| 1699 | Acta1 | 2.497438 | 2.28E-56 |
| 3589 | Cacng6 | 2.487813 | 0.004834 |
| 21538 | Myl1 | 2.486344 | 1.90E-08 |
| 21147 | Mpz | 2.483492 | 5.22E-20 |
| 2671 | Ascl1 | 2.481624 | 5.42E-13 |
| 17608 | Igsf11 | 2.476157 | 3.95E-34 |
| 27561 | Sgca | 2.472194 | 8.97E-09 |
| 29965 | Tmem132e | 2.471748 | 3.50E-44 |
| 21597 | Mypn | 2.47057 | 3.85E-07 |
| 2141 | Alpk3 | 2.469903 | 5.24E-19 |
| 2659 | Asb18 | 2.465252 | 0.00011 |
| 3684 | Car10 | 2.465169 | 0.000193 |
| 22247 | Npy1r | 2.464347 | 1.04E-09 |
| 4577 | Chrna6 | 2.454652 | 7.30E-05 |
| 28321 | Snap25 | 2.45297 | 1.29E-58 |
| 30268 | Tnni1 | 2.442798 | 3.54E-26 |
| 18080 | Kcnc4 | 2.440761 | 2.67E-09 |
| 18099 | Kcnh5 | 2.437521 | 1.54E-06 |
| 6168 | Dner | 2.433384 | 1.56E-25 |
| 18161 | Kcnv2 | 2.433038 | 0.015155 |
| 27848 | Slc22a3 | 2.431963 | 1.49E-08 |
| 2140 | Alpk2 | 2.423234 | 4.74E-06 |
| 4626 | Cilp | 2.41811 | 9.52E-19 |
| 4891 | Cntnap5b | 2.417858 | 1.89E-06 |
| 6032 | Dlgap2 | 2.416079 | 2.97E-05 |
| 1832 | Adgra1 | 2.410866 | 0.003844 |
| 32607 | Zfp385b | 2.402479 | 8.90E-94 |
| 17609 | Igsf21 | 2.397827 | 0.000151 |
| 6282 | Dscaml1 | 2.393926 | 3.94E-06 |
| 17160 | Htr2b | 2.393681 | 5.26E-09 |
| 15 | 1110002E22Rik | 2.391626 | 1.33E-18 |
| 25711 | Ptx3 | 2.389312 | 1.72E-104 |
| 29061 | Strc | 2.38337 | 0.000134 |
| 19321 | Mal | 2.380842 | 3.84E-07 |
| 18155 | Kcns2 | 2.375834 | 9.17E-39 |
| 21982 | Nfatc2 | 2.370589 | 1.04E-138 |
| 9395 | Gm15638 | 2.359778 | 0.005015 |
| 18077 | Kcnc1 | 2.350585 | 1.91E-08 |
| 29063 | Strip2 | 2.341608 | 3.67E-49 |
| 28501 | Sntg1 | 2.337757 | 4.27E-06 |
| 17226 | Idi2 | 2.335289 | 0.000943 |
| 18220 | Khdrbs2 | 2.334295 | 5.44E-72 |
| 7668 | Frmpd4 | 2.320192 | 5.75E-20 |
| 17641 | Il13 | 2.307512 | 0.000173 |
| 26032 | Rbm20 | 2.299168 | 3.40E-39 |
| 27080 | Sall3 | 2.292842 | 2.11E-09 |
| 4801 | Cma1 | 2.283495 | 4.38E-11 |
| 21590 | Myom2 | 2.280353 | 1.69E-10 |
| 7731 | Fut9 | 2.275189 | 2.95E-11 |
| 1527 | Abca8b | 2.267898 | 8.55E-82 |
| 19125 | Lrtm1 | 2.244882 | 0.014154 |
| 24277 | Pcsk1 | 2.243563 | 1.10E-09 |
| 27632 | Shc3 | 2.242139 | 2.91E-15 |
| 3630 | Caly | 2.240086 | 0.000208 |
| 18469 | Krt222 | 2.236979 | 1.99E-07 |
| 5215 | Csgalnact1 | 2.23405 | 4.24E-165 |
| 17880 | Irx4 | 2.23057 | 0.000715 |
| 6380 | Dynlt5 | 2.210893 | 5.07E-07 |
| 18717 | Lefty2 | 2.20666 | 0.000382 |
| 29256 | Sytl5 | 2.203117 | 2.32E-13 |
| 19115 | Lrrn1 | 2.201512 | 0 |
| 21554 | Mylpf | 2.200384 | 7.87E-35 |
| 21481 | Musk | 2.188953 | 7.45E-07 |
| 30266 | Tnnc1 | 2.18816 | 1.42E-24 |
| 4028 | Ccr8 | 2.185271 | 0.001011 |
| 18973 | Lox | 2.183889 | 0 |
| 1156 | 5430431A17Rik | 2.178174 | 1.26E-13 |
| 1270 | 9230105E05Rik | 2.176904 | 0.000801 |
| 17040 | Hoxd9 | 2.170226 | 0.018275 |
| 18773 | Lhx9 | 2.168043 | 4.21E-08 |
| 30013 | Tmem182 | 2.16291 | 0.019057 |
| 25836 | Rab3c | 2.161384 | 0.000106 |
| 29463 | Tbx15 | 2.16022 | 0 |
| 25479 | Prss34 | 2.154576 | 0.013245 |
| 2364 | Apba1 | 2.152021 | 1.19E-49 |
| 1881 | Adora3 | 2.149404 | 0.022102 |
| 24871 | Plxdc2 | 2.146956 | 5.46E-201 |
| 21056 | Mmp3 | 2.135151 | 0.00023 |
| 29780 | Thy1 | 2.133023 | 5.45E-111 |
| 5127 | Creb5 | 2.129213 | 2.17E-194 |
| 27919 | Slc26a7 | 2.10844 | 4.07E-193 |
| 25398 | Prox1 | 2.100567 | 1.97E-32 |
| 27899 | Slc25a41 | 2.097634 | 0.000172 |
| 17911 | Itga4 | 2.09151 | 1.47E-146 |
| 26239 | Rgs8 | 2.087681 | 2.34E-21 |
| 1887 | Adra1a | 2.080415 | 3.28E-10 |
| 27253 | Scn4a | 2.077446 | 2.62E-05 |
| 3483 | C1qtnf2 | 2.072021 | 3.84E-25 |
| 584 | 3110099E03Rik | 2.066689 | 0.000237 |
| 7801 | Gabrg2 | 2.063281 | 0.010259 |
| 8102 | Glis3 | 2.062159 | 2.28E-94 |
| 18085 | Kcne1 | 2.061525 | 0.01764 |
| 22269 | Nr2e3 | 2.055722 | 0.016543 |
| 3561 | Cabp7 | 2.054118 | 0.000328 |
| 1431 | A730056A06Rik | 2.049632 | 1.31E-05 |
| 18350 | Klhl41 | 2.04304 | 1.02E-12 |
| 1802 | Adamtsl3 | 2.032961 | 9.50E-126 |
| 22081 | Nkx2-5 | 2.024665 | 0.004798 |
| 27454 | Serpina3n | 2.012213 | 8.33E-06 |
| 19128 | Lsamp | 2.011883 | 4.78E-79 |
| 1429 | A730046J19Rik | 2.002527 | 0.005399 |
| 29320 | Tafa1 | 1.998772 | 1.94E-09 |
| 25322 | Prkcq | 1.98847 | 2.07E-30 |
| 25831 | Rab38 | 1.987482 | 7.37E-104 |
| 31259 | Unc13c | 1.981564 | 3.64E-19 |
| 6244 | Dpt | 1.977723 | 4.34E-99 |
| 17676 | Il20ra | 1.976105 | 2.84E-11 |
| 18066 | Kcna2 | 1.974718 | 1.22E-13 |
| 1893 | Adrb1 | 1.969398 | 9.43E-11 |
| 16405 | Grm5 | 1.969012 | 0.023341 |
| 28760 | Spock3 | 1.963395 | 1.04E-42 |
| 32218 | Wnt16 | 1.960273 | 3.58E-61 |
| 16319 | Gpr88 | 1.958745 | 2.88E-05 |
| 4888 | Cntnap3 | 1.956321 | 0.00045 |
| 16541 | Gzmb | 1.955187 | 4.72E-07 |
| 27387 | Sema5b | 1.953648 | 1.21E-71 |
| 767 | 4930474M22Rik | 1.949032 | 1.27E-11 |
| 5923 | Des | 1.947041 | 6.37E-83 |
| 5719 | Dcn | 1.946871 | 6.16E-248 |
| 23995 | P2ry10 | 1.946003 | 0.004173 |
| 22463 | Nxph3 | 1.945093 | 1.06E-41 |
| 513 | 2610028E06Rik | 1.914677 | 0.008073 |
| 1445 | A830082K12Rik | 1.913253 | 2.09E-91 |
| 29281 | Tac1 | 1.90518 | 3.86E-23 |
| 29707 | Tgfb2 | 1.904078 | 6.96E-175 |
| 28860 | Srpk3 | 1.903767 | 1.71E-07 |
| 18078 | Kcnc2 | 1.901223 | 5.05E-14 |
| 2136 | Alox8 | 1.898547 | 2.03E-08 |
| 27035 | Rxrg | 1.895473 | 7.83E-10 |
| 18120 | Kcnj8 | 1.89106 | 4.81E-24 |
| 14031 | Gm40909 | 1.881693 | 0.015252 |
| 20162 | Mir344-2 | 1.879844 | 0.015084 |
| 1524 | Abca6 | 1.87663 | 2.59E-28 |
| 1890 | Adra2a | 1.873566 | 9.93E-47 |
| 686 | 4930429F24Rik | 1.871311 | 2.20E-07 |
| 6207 | Dok6 | 1.864067 | 2.95E-33 |
| 21532 | Myh6 | 1.862005 | 5.36E-07 |
| 25267 | Prdm6 | 1.861623 | 1.57E-77 |
| 1514 | Abca12 | 1.860757 | 1.82E-05 |
| 27186 | Scg2 | 1.859795 | 8.41E-33 |
| 19739 | Mgp | 1.856318 | 1.59E-36 |
| 2397 | Apobec2 | 1.855925 | 2.66E-05 |
| 1000 | 4930599N23Rik | 1.8551 | 0.007203 |
| 3688 | Car14 | 1.853395 | 5.16E-33 |
| 17058 | Hpse | 1.851438 | 1.89E-79 |
| 18859 | Lmod3 | 1.845686 | 0.00328 |
| 27352 | Sel1l3 | 1.84459 | 7.96E-08 |
| 7491 | Filip1 | 1.842442 | 2.18E-61 |
| 2210 | Angpt4 | 1.838493 | 0.001177 |
| 15255 | Gm7328 | 1.838268 | 0.008744 |
| 25175 | Ppp3r2 | 1.834358 | 2.64E-07 |
| 24910 | Pnma2 | 1.832636 | 2.67E-28 |
| 771 | 4930478L05Rik | 1.83102 | 0.018066 |
| 16387 | Grin3a | 1.829597 | 9.56E-101 |
| 32262 | Xkr4 | 1.82345 | 6.62E-06 |
| 6935 | Esyt3 | 1.823142 | 9.26E-15 |
| 8919 | Gm13652 | 1.816248 | 0.014618 |
| 2967 | B3galt5 | 1.814062 | 2.06E-10 |
| 25712 | Ptx4 | 1.809628 | 0.014884 |
| 32155 | Wdr72 | 1.807022 | 3.09E-07 |
| 5902 | Dennd2d | 1.795224 | 4.33E-12 |
| 25899 | Radil | 1.795023 | 1.60E-49 |
| 27861 | Slc24a3 | 1.794339 | 4.35E-74 |
| 18083 | Kcnd3 | 1.790233 | 1.18E-25 |
| 6208 | Dok7 | 1.789562 | 2.21E-11 |
| 18171 | Kctd16 | 1.788691 | 1.85E-06 |
| 23945 | Otogl | 1.784857 | 7.77E-17 |
| 31135 | Ube2ql1 | 1.784636 | 1.28E-25 |
| 6191 | Dock10 | 1.784174 | 9.06E-126 |
| 422 | 2010016I18Rik | 1.779332 | 0.017008 |
| 30931 | Ttc22 | 1.776953 | 3.57E-08 |
| 6686 | Eln | 1.776638 | 3.73E-158 |
| 17714 | Il9r | 1.773791 | 0.023198 |
| 2443 | Aqp5 | 1.773765 | 0.023047 |
| 7466 | Fhad1 | 1.771667 | 0.006103 |
| 24438 | Pex5l | 1.76036 | 0.000904 |
| 27150 | Sbspon | 1.759232 | 1.22E-10 |
| 21591 | Myom3 | 1.756769 | 1.02E-08 |
| 28095 | Slc6a2 | 1.738762 | 0.003561 |
| 25054 | Pou6f2 | 1.737118 | 0.011091 |
| 29472 | Tbx3os2 | 1.735592 | 6.95E-07 |
| 6174 | Dnm3 | 1.731896 | 3.67E-128 |
| 5059 | Cpeb1 | 1.72995 | 2.21E-05 |
| 22271 | Nr2f2 | 1.729729 | 3.08E-152 |
| 7964 | Gdf7 | 1.729585 | 2.80E-30 |
| 14490 | Gm50470 | 1.727016 | 2.07E-13 |
| 16780 | Hcn1 | 1.720879 | 1.15E-06 |
| 27252 | Scn3b | 1.710877 | 1.03E-12 |
| 27029 | Rxfp1 | 1.696435 | 7.63E-18 |
| 7004 | Exph5 | 1.686839 | 3.15E-08 |
| 7406 | Fer1l6 | 1.684469 | 0.012707 |
| 4216 | Cdh8 | 1.682686 | 6.46E-23 |
| 7558 | Fn3k | 1.682208 | 1.98E-09 |
| 24681 | Piwil4 | 1.680789 | 0.016343 |
| 6587 | Eid3 | 1.678365 | 0.000608 |
| 4580 | Chrnb1 | 1.677837 | 5.14E-28 |
| 25045 | Pou3f4 | 1.677724 | 3.12E-06 |
| 16376 | Grik1 | 1.67703 | 1.29E-69 |
| 19741 | Mgst1 | 1.673001 | 4.91E-41 |
| 24875 | Plxna4 | 1.672726 | 3.95E-59 |
| 4681 | Cldn1 | 1.670672 | 8.23E-43 |
| 9582 | Gm16897 | 1.669973 | 8.55E-14 |
| 3578 | Cacna2d3 | 1.667259 | 4.88E-72 |
| 29110 | Sulf1 | 1.65135 | 2.62E-210 |
| 4274 | Cdr1os | 1.646183 | 4.91E-09 |
| 22293 | Nrg1 | 1.6424 | 7.80E-27 |
| 19433 | Marchf3 | 1.637895 | 1.71E-16 |
| 7451 | Fgf9 | 1.627906 | 1.15E-08 |
| 26034 | Rbm24 | 1.624055 | 5.89E-19 |
| 24747 | Platr14 | 1.623606 | 0.000332 |
| 26016 | Rbfox3 | 1.622957 | 1.15E-12 |
| 5394 | Cxcr6 | 1.619567 | 0.004975 |
| 27862 | Slc24a4 | 1.618388 | 5.49E-07 |
| 17670 | Il1rapl2 | 1.612349 | 0.001138 |
| 4950 | Col6a5 | 1.608279 | 1.36E-06 |
| 9502 | Gm16249 | 1.604417 | 0.022643 |
| 24801 | Plekhb1 | 1.601617 | 1.42E-09 |
| 523 | 2610316D01Rik | 1.601031 | 4.92E-57 |
| 1312 | 9530026P05Rik | 1.599793 | 2.06E-12 |
| 9425 | Gm15789 | 1.592047 | 0.0006 |
| 5240 | Cspg5 | 1.589243 | 9.72E-46 |
| 18343 | Klhl33 | 1.585237 | 1.86E-09 |
| 7483 | Fibin | 1.580394 | 3.73E-148 |
| 5645 | Dach1 | 1.579846 | 8.52E-98 |
| 22551 | Odam | 1.574084 | 0.01469 |
| 1438 | A830018L16Rik | 1.571526 | 1.03E-34 |
| 3458 | C030034L19Rik | 1.568516 | 7.75E-05 |
| 16369 | Gria2 | 1.568515 | 1.12E-78 |
| 9410 | Gm15737 | 1.567665 | 0.023386 |
| 7922 | Gbp8 | 1.566943 | 5.73E-05 |
| 7369 | Fcgbp | 1.565902 | 0.000135 |
| 10953 | Gm22352 | 1.56279 | 0.001523 |
| 1236 | 8430426J06Rik | 1.56262 | 0.016341 |
| 17876 | Irs4 | 1.557084 | 5.30E-10 |
| 2365 | Apba2 | 1.546525 | 3.08E-14 |
| 22060 | Nkain2 | 1.545098 | 8.51E-13 |
| 3179 | Bean1 | 1.544775 | 1.97E-30 |
| 27813 | Slc18a1 | 1.543002 | 0.003269 |
| 29774 | Thsd7a | 1.536341 | 1.63E-23 |
| 17156 | Htr1b | 1.536165 | 1.38E-25 |
| 27611 | Sh3gl2 | 1.535939 | 6.91E-22 |
| 18349 | Klhl40 | 1.533953 | 0.010463 |
| 19078 | Lrrc4c | 1.532415 | 4.77E-09 |
| 4819 | Cmtm5 | 1.53201 | 0.004618 |
| 26191 | Rflna | 1.530131 | 3.56E-12 |
| 17169 | Htra1 | 1.529949 | 2.22E-56 |
| 500 | 2410021H03Rik | 1.527287 | 0.000213 |
| 29991 | Tmem163 | 1.526928 | 0.000593 |
| 2779 | Atp1a2 | 1.526198 | 2.59E-19 |
| 8833 | Gm13302 | 1.524764 | 2.46E-09 |
| 28285 | Smoc2 | 1.524101 | 1.01E-145 |
| 27612 | Sh3gl3 | 1.522339 | 3.36E-38 |
| 4599 | Chst8 | 1.520098 | 1.09E-17 |
| 4949 | Col6a4 | 1.518585 | 0.001118 |
| 17914 | Itga7 | 1.517316 | 2.39E-13 |
| 5379 | Cxcl12 | 1.517076 | 1.99E-175 |
| 6206 | Dok5 | 1.51298 | 1.22E-24 |
| 27673 | Siah3 | 1.510804 | 1.97E-13 |
| 4217 | Cdh9 | 1.509323 | 0.000381 |
| 1799 | Adamts9 | 1.508389 | 3.21E-122 |
| 24879 | Plxnb3 | 1.506772 | 1.37E-09 |
| 5381 | Cxcl14 | 1.502089 | 2.03E-117 |
| 18982 | Lpar4 | 1.496926 | 6.89E-87 |
| 27599 | Sh3bgr | 1.491644 | 0.003897 |
| 7894 | Gata3 | 1.485466 | 2.88E-06 |
| 1680 | Acsbg1 | 1.485119 | 0.000498 |
| 28552 | Soga3 | 1.47681 | 0.002525 |
| 494 | 2410004I01Rik | 1.466446 | 0.0048 |
| 7595 | Foxd2 | 1.464752 | 0.00209 |
| 1827 | Adcyap1r1 | 1.461873 | 1.79E-06 |
| 7803 | Gabrp | 1.459068 | 0.000705 |
| 1528 | Abca9 | 1.457901 | 4.52E-53 |
| 3275 | Bnc1 | 1.453006 | 2.79E-18 |
| 30192 | Tmprss5 | 1.451604 | 0.017239 |
| 25624 | Ptgdr | 1.451429 | 0.009912 |
| 3528 | C530044C16Rik | 1.450394 | 0.000222 |
| 23944 | Otog | 1.444779 | 0.003928 |
| 25699 | Ptprt | 1.438769 | 1.19E-37 |
| 12123 | Gm24564 | 1.437285 | 0.000505 |
| 2447 | Aqp9 | 1.437071 | 3.19E-08 |
| 18750 | Lgi4 | 1.436326 | 1.62E-05 |
| 24706 | Pknox2 | 1.434609 | 1.49E-25 |
| 5711 | Dchs2 | 1.432315 | 1.82E-33 |
| 16929 | Hmga2-ps1 | 1.428611 | 2.16E-10 |
| 16783 | Hcn4 | 1.427631 | 1.86E-30 |
| 17669 | Il1rapl1 | 1.427272 | 0.00014 |
| 24708 | Pkp2 | 1.422751 | 4.13E-06 |
| 18828 | Lix1 | 1.419718 | 2.15E-23 |
| 7247 | Far1os | 1.412454 | 7.74E-08 |
| 17643 | Il13ra2 | 1.405313 | 0.008338 |
| 25519 | Psd3 | 1.403315 | 1.36E-111 |
| 19155 | Ltbp2 | 1.399827 | 6.20E-33 |
| 814 | 4930511M06Rik | 1.398262 | 0.001246 |
| 4272 | Cdon | 1.397659 | 2.74E-123 |
| 16876 | Hhip | 1.39687 | 1.12E-29 |
| 17662 | Il1a | 1.394762 | 0.003082 |
| 18082 | Kcnd2 | 1.394746 | 0.013155 |
| 6663 | Elavl2 | 1.394516 | 1.43E-08 |
| 2013 | Ajap1 | 1.394063 | 6.43E-05 |
| 6311 | Dtx4 | 1.393101 | 1.31E-79 |
| 19729 | Mgat4c | 1.391543 | 3.65E-15 |
| 19009 | Lrfn2 | 1.389833 | 6.75E-06 |
| 25628 | Ptger2 | 1.388878 | 3.59E-06 |
| 713 | 4930444P10Rik | 1.385146 | 6.93E-05 |
| 25846 | Rab44 | 1.384006 | 0.00053 |
| 17140 | Hspb6 | 1.38138 | 1.89E-33 |
| 23950 | Otor | 1.381196 | 6.95E-28 |
| 24803 | Plekhd1 | 1.379977 | 0.011288 |
| 17627 | Ikzf2 | 1.375054 | 1.26E-40 |
| 15442 | Gm7819 | 1.373957 | 1.50E-18 |
| 16922 | Hmcn1 | 1.372602 | 1.89E-88 |
| 25889 | Rad51ap2 | 1.372059 | 0.004994 |
| 1804 | Adamtsl5 | 1.370226 | 1.17E-18 |
| 7913 | Gbp10 | 1.363416 | 0.000431 |
| 19276 | Mag | 1.359381 | 0.004433 |
| 29077 | Stx19 | 1.35888 | 0.003706 |
| 28575 | Sox10 | 1.354804 | 5.58E-25 |
| 22368 | Ntrk3 | 1.349864 | 2.45E-39 |
| 18994 | Lpl | 1.348276 | 2.57E-69 |
| 15005 | Gm6418 | 1.347436 | 1.18E-07 |
| 25958 | Rarres1 | 1.342495 | 5.03E-05 |
| 1640 | Ache | 1.341522 | 1.33E-16 |
| 3615 | Calhm4 | 1.341124 | 0.000524 |
| 7458 | Fgfr4 | 1.33726 | 3.30E-19 |
| 1209 | 6430590A07Rik | 1.336503 | 0.00133 |
| 32232 | Wnt9b | 1.335733 | 0.001351 |
| 7439 | Fgf18 | 1.334807 | 2.35E-54 |
| 5706 | Dcc | 1.334763 | 6.83E-52 |
| 27375 | Sema3c | 1.334202 | 1.75E-29 |
| 7657 | Frmd3 | 1.333668 | 1.83E-10 |
| 27479 | Serpinb8 | 1.326809 | 5.36E-28 |
| 9696 | Gm17907 | 1.32621 | 0.020203 |
| 27660 | Shox2 | 1.31799 | 3.60E-110 |
| 29837 | Tktl1 | 1.317013 | 0.000254 |
| 3575 | Cacna1s | 1.314515 | 0.000891 |
| 5048 | Cp | 1.312172 | 1.11E-06 |
| 1409 | A630001G21Rik | 1.311891 | 2.35E-12 |
| 7920 | Gbp6 | 1.309971 | 0.000362 |
| 22326 | Nsg2 | 1.308018 | 4.09E-05 |
| 16737 | Has1 | 1.304863 | 2.83E-09 |
| 16866 | Hfm1 | 1.304824 | 0.004464 |
| 3058 | Bbox1 | 1.303004 | 8.03E-11 |
| 14699 | Gm5535 | 1.296745 | 0.019511 |
| 8339 | Gm11266 | 1.296052 | 3.39E-10 |
| 3269 | Bmpr1b | 1.292629 | 3.72E-12 |
| 8221 | Gm10389 | 1.292207 | 0.000942 |
| 19403 | Mapk10 | 1.287074 | 7.06E-06 |
| 17159 | Htr2a | 1.285109 | 3.21E-07 |
| 1852 | Adgrg6 | 1.284025 | 8.94E-81 |
| 16732 | Hapln3 | 1.283261 | 3.65E-14 |
| 24777 | Plcl1 | 1.278723 | 4.27E-18 |
| 28863 | Srpx | 1.278638 | 1.16E-51 |
| 3831 | Ccdc141 | 1.277445 | 5.08E-22 |
| 6952 | Etv1 | 1.27644 | 5.47E-37 |
| 7880 | Gas1 | 1.271802 | 6.58E-77 |
| 30265 | Tnn | 1.27121 | 2.94E-92 |
| 3676 | Caps2 | 1.267902 | 0.023225 |
| 3185 | Bend5 | 1.266013 | 6.44E-66 |
| 6250 | Dpyd | 1.265151 | 5.60E-70 |
| 16516 | Gucy1b2 | 1.262776 | 0.000165 |
| 28717 | Sphkap | 1.259309 | 4.78E-06 |
| 7838 | Galnt17 | 1.250574 | 2.25E-20 |
| 7834 | Galnt13 | 1.248646 | 3.78E-17 |
| 9442 | Gm15910 | 1.239818 | 0.000101 |
| 2598 | Armh1 | 1.238346 | 4.33E-13 |
| 23934 | Osr1 | 1.238152 | 1.08E-80 |
| 16275 | Gpr17 | 1.235188 | 6.06E-16 |
| 18106 | Kcnip4 | 1.231865 | 1.62E-12 |
| 7132 | Fam171b | 1.231148 | 1.22E-97 |
| 19122 | Lrrtm3 | 1.23022 | 9.00E-23 |
| 19066 | Lrrc3b | 1.22934 | 1.17E-06 |
| 19154 | Ltbp1 | 1.229205 | 5.27E-121 |
| 23966 | Otx1 | 1.227555 | 0.014024 |
| 28970 | Stambpl1 | 1.22421 | 1.61E-34 |
| 23932 | Osm | 1.222294 | 0.02197 |
| 7554 | Fmod | 1.212207 | 4.35E-42 |
| 25432 | Prr16 | 1.207949 | 7.33E-92 |
| 21534 | Myh7b | 1.206455 | 4.21E-08 |
| 7858 | Gap43 | 1.204463 | 8.99E-57 |
| 5052 | Cpa4 | 1.203293 | 1.77E-21 |
| 30863 | Tshz2 | 1.199176 | 4.83E-60 |
| 29743 | Thbs1 | 1.194046 | 5.17E-108 |
| 5605 | D430036J16Rik | 1.193122 | 6.28E-06 |
| 2134 | Alox5 | 1.190819 | 0.003558 |
| 29206 | Syn3 | 1.185945 | 7.78E-15 |
| 2137 | Aloxe3 | 1.18383 | 1.61E-05 |
| 4962 | Colgalt2 | 1.183167 | 7.82E-35 |
| 7899 | Gata6os | 1.18006 | 0.004234 |
| 24310 | Pde10a | 1.17752 | 1.08E-37 |
| 6325 | Dusp13 | 1.177241 | 0.000263 |
| 16317 | Gpr85 | 1.177126 | 1.68E-12 |
| 29842 | Tlcd3b | 1.175559 | 0.007627 |
| 26357 | Ripor2 | 1.174848 | 1.13E-09 |
| 24315 | Pde1c | 1.172835 | 2.15E-15 |
| 4931 | Col25a1 | 1.171616 | 7.08E-80 |
| 17655 | Il17rd | 1.170036 | 2.49E-63 |
| 22062 | Nkain4 | 1.168516 | 3.72E-08 |
| 22308 | Nrp1 | 1.168066 | 2.34E-78 |
| 27016 | Runx1t1 | 1.16493 | 6.08E-70 |
| 27877 | Slc25a21 | 1.164691 | 2.52E-07 |
| 27821 | Slc1a2 | 1.162822 | 1.44E-18 |
| 30755 | Trim72 | 1.159881 | 0.00501 |
| 31267 | Unc5c | 1.156031 | 5.43E-54 |
| 3922 | Ccdc80 | 1.154851 | 3.71E-67 |
| 26760 | Rprm | 1.154176 | 3.45E-19 |
| 5612 | D630023F18Rik | 1.151185 | 0.018481 |
| 26998 | Rtn4rl2 | 1.150122 | 4.36E-08 |
| 20991 | Mitf | 1.147869 | 1.22E-19 |
| 7527 | Flnc | 1.147481 | 2.21E-79 |
| 10361 | Gm19705 | 1.143741 | 0.007862 |
| 6050 | Dlx6os1 | 1.1419 | 1.13E-34 |
| 3625 | Caln1 | 1.141301 | 3.31E-08 |
| 19911 | Mir1898 | 1.137861 | 0.001004 |
| 30131 | Tmem59l | 1.137517 | 5.55E-32 |
| 27920 | Slc26a8 | 1.137185 | 0.001456 |
| 1398 | A530016L24Rik | 1.135944 | 0.001731 |
| 29964 | Tmem132d | 1.130658 | 7.27E-07 |
| 21589 | Myom1 | 1.13007 | 1.07E-16 |
| 5179 | Cryab | 1.129717 | 1.30E-09 |
| 21975 | Nexn | 1.127548 | 4.19E-24 |
| 16868 | Hgf | 1.126985 | 3.29E-15 |
| 18471 | Krt24 | 1.122463 | 0.021031 |
| 19454 | Masp1 | 1.121715 | 8.75E-37 |
| 7065 | Fads6 | 1.116366 | 0.020986 |
| 18183 | Kctd8 | 1.113588 | 0.000252 |
| 7919 | Gbp5 | 1.111574 | 0.017121 |
| 3610 | Calcrl | 1.110154 | 2.68E-35 |
| 6848 | Erbb3 | 1.109111 | 4.68E-34 |
| 24796 | Plekha4 | 1.108957 | 3.26E-12 |
| 6025 | Dleu7 | 1.108351 | 2.27E-05 |
| 18767 | Lhx2 | 1.106427 | 0.009786 |
| 439 | 2210408I21Rik | 1.105274 | 8.32E-26 |
| 6815 | Ephb3 | 1.10436 | 1.05E-73 |
| 18340 | Klhl30 | 1.100776 | 0.000931 |
| 10183 | Gm18980 | 1.100375 | 0.00016 |
| 21809 | Nckap5 | 1.098858 | 1.62E-12 |
| 14664 | Gm54215 | 1.094357 | 3.89E-07 |
| 17986 | Jam2 | 1.080335 | 4.37E-14 |
| 27006 | Rubcnl | 1.079516 | 2.31E-06 |
| 24838 | Plk5 | 1.075847 | 0.000747 |
| 29508 | Tcf4 | 1.074074 | 1.09E-73 |
| 22274 | Nr3c2 | 1.073187 | 1.12E-15 |
| 25696 | Ptprq | 1.071791 | 0.001848 |
| 27039 | Ryr1 | 1.071258 | 1.96E-26 |
| 7597 | Foxd3 | 1.065266 | 0.000137 |
| 25148 | Ppp1r3g | 1.064618 | 0.006962 |
| 5547 | Cyp7b1 | 1.064475 | 1.69E-22 |
| 2084 | Aldh1l2 | 1.063781 | 2.97E-10 |
| 6398 | E130008D07Rik | 1.062926 | 1.16E-06 |
| 13897 | Gm3695 | 1.062365 | 0.010754 |
| 29962 | Tmem132c | 1.059216 | 7.15E-81 |
| 16519 | Gucy2e | 1.058634 | 2.76E-07 |
| 1800 | Adamtsl1 | 1.056507 | 1.25E-49 |
| 1187 | 6030443J06Rik | 1.053109 | 8.17E-06 |
| 7723 | Fut1 | 1.053024 | 0.003938 |
| 29253 | Sytl2 | 1.049368 | 1.00E-16 |
| 6698 | Elovl7 | 1.048269 | 0.000768 |
| 348 | 1700109K24Rik | 1.047716 | 2.86E-16 |
| 1787 | Adamts16 | 1.042816 | 0.000135 |
| 19001 | Lratd2 | 1.041709 | 4.09E-17 |
| 26529 | Rora | 1.039333 | 1.86E-49 |
| 5441 | Cyp27a1 | 1.037051 | 3.24E-08 |
| 2792 | Atp2b3 | 1.036592 | 6.77E-05 |
| 30221 | Tnfaip8l3 | 1.031848 | 5.28E-13 |
| 3766 | Cbfa2t3 | 1.029507 | 4.12E-36 |
| 21563 | Myo18b | 1.028552 | 4.78E-06 |
| 4880 | Cntn1 | 1.028301 | 8.61E-25 |
| 28844 | Srgn | 1.022121 | 6.96E-06 |
| 28864 | Srpx2 | 1.018028 | 1.25E-10 |
| 6501 | Ednra | 1.017503 | 1.10E-71 |
| 6264 | Draxin | 1.016396 | 2.42E-12 |
| 4927 | Col20a1 | 1.014129 | 6.67E-17 |
| 28983 | Stard9 | 1.011696 | 4.05E-39 |
| 27394 | Semp2l1 | 1.009269 | 0.023522 |
| 5648 | Dact2 | 1.007402 | 9.34E-15 |
| 25944 | Rap2b | 1.007207 | 6.70E-31 |
| 8464 | Gm11827 | 1.00364 | 0.000575 |
| 26440 | Rnf152 | 0.997398 | 2.60E-19 |
| 4204 | Cdh19 | 0.997333 | 0.000608 |
| 19323 | Malat1 | 0.996967 | 7.10E-66 |
| 18064 | Kcna1 | 0.991028 | 1.65E-05 |
| 32392 | Zbtb37 | 0.988853 | 1.86E-25 |
| 8065 | Gjb6 | 0.988698 | 0.013565 |
| 24189 | Pcdh9 | 0.988599 | 5.17E-27 |
| 17990 | Jazf1 | 0.988089 | 5.68E-31 |
| 2682 | Asic1 | 0.985426 | 3.86E-17 |
| 26966 | Rspo2 | 0.984548 | 2.34E-26 |
| 7631 | Foxq1 | 0.9828 | 0.021033 |
| 18342 | Klhl32 | 0.981904 | 3.33E-05 |
| 8128 | Glt8d2 | 0.981599 | 2.37E-30 |
| 28994 | Stc1 | 0.981294 | 1.52E-20 |
| 1769 | Adam33 | 0.976706 | 3.91E-39 |
| 3774 | Cbln2 | 0.97456 | 4.36E-06 |
| 28041 | Slc44a5 | 0.973516 | 6.04E-31 |
| 32645 | Zfp469 | 0.969655 | 1.83E-24 |
| 6095 | Dnah11 | 0.969105 | 0.008634 |
| 7893 | Gata2 | 0.966069 | 1.04E-10 |
| 24317 | Pde3a | 0.964075 | 6.98E-36 |
| 30029 | Tmem200a | 0.963977 | 1.98E-39 |
| 17756 | Inka2 | 0.961961 | 0.000107 |
| 7889 | Gask1a | 0.958592 | 1.12E-06 |
| 27538 | Sfmbt2 | 0.958498 | 2.33E-22 |
| 28912 | Sstr1 | 0.95655 | 1.51E-08 |
| 2211 | Angptl1 | 0.954562 | 1.40E-49 |
| 5292 | Ctf2 | 0.954535 | 0.002313 |
| 10302 | Gm19220 | 0.950217 | 3.13E-15 |
| 28957 | St8sia6 | 0.946687 | 0.009927 |
| 29511 | Tcf7l2 | 0.945328 | 9.38E-45 |
| 26229 | Rgs2 | 0.945116 | 4.26E-27 |
| 30923 | Ttc12 | 0.942005 | 3.59E-08 |
| 26269 | Rhoj | 0.940262 | 2.25E-36 |
| 28155 | Slf1 | 0.938007 | 1.04E-41 |
| 3735 | Casq1 | 0.935871 | 0.000288 |
| 27497 | Serpini1 | 0.932885 | 1.51E-07 |
| 7835 | Galnt14 | 0.932715 | 3.48E-06 |
| 6010 | Disp1 | 0.93256 | 1.37E-43 |
| 29470 | Tbx3 | 0.930169 | 6.84E-57 |
| 21788 | Ncan | 0.929565 | 0.000778 |
| 29009 | Sting1 | 0.929271 | 1.37E-18 |
| 7996 | Gfra1 | 0.927694 | 2.71E-21 |
| 26946 | Rsad2 | 0.926241 | 0.015677 |
| 21051 | Mmp23 | 0.925835 | 1.88E-08 |
| 6561 | Egfl6 | 0.925578 | 6.90E-41 |
| 32370 | Zbed6 | 0.925406 | 8.77E-06 |
| 6234 | Dpp4 | 0.91979 | 0.001618 |
| 4372 | Cep295nl | 0.918806 | 0.014611 |
| 27448 | Serpina3h | 0.918063 | 4.37E-07 |
| 3711 | Carmil3 | 0.917618 | 1.03E-24 |
| 14091 | Gm42573 | 0.916886 | 0.017763 |
| 3583 | Cacnb4 | 0.916291 | 2.07E-08 |
| 6741 | Enc1 | 0.913867 | 4.52E-48 |
| 29961 | Tmem132b | 0.913007 | 8.84E-13 |
| 28170 | Slit2 | 0.911398 | 8.59E-58 |
| 3039 | Barx2 | 0.911045 | 3.41E-11 |
| 29513 | Tchh | 0.910097 | 0.000773 |
| 24537 | Phip | 0.907729 | 3.20E-59 |
| 3673 | Capns2 | 0.907381 | 0.015081 |
| 16823 | Hectd2 | 0.907207 | 2.76E-13 |
| 25684 | Ptprc | 0.904795 | 1.27E-10 |
| 18266 | Kif6 | 0.904322 | 9.90E-06 |
| 16238 | Gpm6a | 0.903658 | 1.74E-07 |
| 16012 | Gm9517 | 0.901901 | 3.34E-07 |
| 16302 | Gpr4 | 0.89588 | 2.80E-08 |
| 9637 | Gm17415 | 0.894905 | 2.13E-06 |
| 2178 | Ampd3 | 0.894166 | 7.71E-07 |
| 2275 | Ankrd55 | 0.891417 | 0.023745 |
| 1594 | Ablim3 | 0.891151 | 4.05E-07 |
| 18006 | Jph2 | 0.89054 | 9.76E-05 |
| 23988 | P2rx2 | 0.888405 | 0.022648 |
| 25283 | Prelp | 0.888161 | 9.01E-18 |
| 5050 | Cpa2 | 0.886803 | 1.14E-08 |
| 32733 | Zfp663 | 0.885532 | 0.001891 |
| 22061 | Nkain3 | 0.885329 | 0.002507 |
| 18283 | Kiss1r | 0.884092 | 0.002685 |
| 16377 | Grik2 | 0.882908 | 1.02E-06 |
| 19192 | Ly6h | 0.882875 | 5.46E-18 |
| 7248 | Far2 | 0.882285 | 2.34E-16 |
| 1974 | Ahrr | 0.881369 | 3.84E-11 |
| 28915 | Sstr4 | 0.881255 | 7.22E-08 |
| 17891 | Islr | 0.881139 | 5.14E-34 |
| 4320 | Celf3 | 0.878708 | 0.002246 |
| 6723 | Emilin3 | 0.878429 | 3.81E-39 |
| 29605 | Tent5a | 0.878337 | 3.96E-13 |
| 1300 | 9430037G07Rik | 0.876561 | 0.005867 |
| 17136 | Hspb1 | 0.874862 | 5.38E-05 |
| 27912 | Slc26a10 | 0.873998 | 1.66E-05 |
| 7997 | Gfra2 | 0.871881 | 1.65E-45 |
| 28935 | St3gal1 | 0.871242 | 1.40E-46 |
| 16383 | Grin2a | 0.871211 | 5.71E-06 |
| 29227 | Synpo2 | 0.869615 | 7.99E-31 |
| 19683 | Mexis | 0.868663 | 0.002238 |
| 18076 | Kcnb2 | 0.868604 | 7.54E-11 |
| 7970 | Gdpd2 | 0.865955 | 3.12E-06 |
| 4111 | Cd40 | 0.865127 | 1.97E-08 |
| 4102 | Cd34 | 0.863431 | 2.37E-42 |
| 2166 | Amigo1 | 0.86157 | 1.78E-28 |
| 27758 | Slc10a1 | 0.860496 | 9.49E-05 |
| 29642 | Tex15 | 0.858186 | 1.18E-15 |
| 28044 | Slc45a3 | 0.856768 | 1.22E-05 |
| 19620 | Mei4 | 0.8555 | 0.000214 |
| 21991 | Nfib | 0.853795 | 1.19E-64 |
| 2787 | Atp2a1 | 0.853304 | 7.26E-07 |
| 29712 | Tgfbr3 | 0.850179 | 3.46E-38 |
| 19091 | Lrrc7 | 0.849711 | 3.36E-19 |
| 14819 | Gm5860 | 0.848009 | 0.00089 |
| 4934 | Col28a1 | 0.846828 | 0.017715 |
| 30350 | Tpm1 | 0.845297 | 1.75E-51 |
| 18116 | Kcnj3 | 0.844833 | 2.53E-15 |
| 6430 | E330013P04Rik | 0.843914 | 0.018313 |
| 16384 | Grin2b | 0.843757 | 0.000628 |
| 16268 | Gpr156 | 0.843113 | 0.00212 |
| 8116 | Glrb | 0.841828 | 2.34E-12 |
| 16603 | H2-T10 | 0.840353 | 0.001281 |
| 25703 | Ptprz1 | 0.840002 | 9.17E-38 |
| 4952 | Col7a1 | 0.838126 | 5.49E-37 |
| 4290 | Ceacam1 | 0.837674 | 0.016898 |
| 28174 | Slitrk3 | 0.833744 | 0.002308 |
| 7837 | Galnt16 | 0.830535 | 2.32E-29 |
| 26254 | Rhd | 0.830302 | 0.010635 |
| 1250 | 9130008F23Rik | 0.82916 | 0.005564 |
| 5219 | Csmd1 | 0.827149 | 0.003811 |
| 29742 | Thbd | 0.826357 | 4.19E-22 |
| 3284 | Boc | 0.825108 | 2.30E-41 |
| 32718 | Zfp641 | 0.823022 | 5.62E-12 |
| 32010 | Vps13b | 0.820465 | 1.18E-33 |
| 16798 | Hdac9 | 0.82037 | 3.22E-25 |
| 6563 | Egfl8 | 0.820251 | 0.000131 |
| 3985 | Ccn6 | 0.819465 | 0.008324 |
| 25629 | Ptger3 | 0.81934 | 3.50E-05 |
| 29860 | Tlr1 | 0.816992 | 0.000102 |
| 25979 | Rasgrp4 | 0.816527 | 6.31E-10 |
| 32225 | Wnt5b | 0.816211 | 1.72E-11 |
| 2080 | Aldh1a3 | 0.81583 | 9.60E-15 |
| 5204 | Csdc2 | 0.815239 | 1.40E-13 |
| 7280 | Fbn2 | 0.814778 | 1.31E-42 |
| 18457 | Krt1 | 0.813234 | 0.000545 |
| 1379 | A330076H08Rik | 0.81317 | 2.94E-09 |
| 4566 | Chrm2 | 0.813147 | 4.07E-08 |
| 18862 | Lmx1a | 0.813017 | 0.000146 |
| 2036 | Akap6 | 0.811276 | 6.52E-05 |
| 27563 | Sgcd | 0.808329 | 8.60E-12 |
| 1550 | Abcd2 | 0.807563 | 4.13E-08 |
| 16916 | Hlf | 0.806566 | 4.78E-15 |
| 29242 | Syt16 | 0.80444 | 7.19E-10 |
| 7437 | Fgf16 | 0.803562 | 0.012969 |
| 7278 | Fbln7 | 0.802877 | 0.000795 |
| 3908 | Ccdc65 | 0.80251 | 0.011276 |
| 27257 | Scn8a | 0.801808 | 5.06E-16 |
| 19408 | Mapk15 | 0.801771 | 4.77E-09 |
| 7968 | Gdnf | 0.801767 | 1.12E-06 |
| 30274 | Tnnt3 | 0.800265 | 0.001104 |
| 30822 | Trpm2 | 0.800112 | 0.005583 |
| 9756 | Gm18030 | 0.799525 | 0.000872 |
| 18296 | Klf12 | 0.797872 | 4.73E-16 |
| 18699 | Ldb3 | 0.797706 | 0.018951 |
| 16393 | Grip2 | 0.797477 | 8.67E-10 |
| 29167 | Sv2c | 0.797271 | 3.35E-12 |
| 16514 | Gucy1a2 | 0.796901 | 4.19E-05 |
| 29173 | Svep1 | 0.796311 | 2.18E-20 |
| 2373 | Apcdd1 | 0.79584 | 1.30E-44 |
| 24089 | Paqr6 | 0.795342 | 0.001915 |
| 29091 | Stxbp5 | 0.792955 | 5.84E-36 |
| 25900 | Radx | 0.792693 | 0.011556 |
| 8984 | Gm13986 | 0.790074 | 0.011577 |
| 2783 | Atp1b2 | 0.789201 | 1.26E-16 |
| 24901 | Pnck | 0.787438 | 3.32E-13 |
| 6779 | Entrep1 | 0.78625 | 0.003534 |
| 29574 | Tec | 0.782775 | 6.25E-06 |
| 2848 | Atp8a2 | 0.781608 | 5.55E-07 |
| 26151 | Rem2 | 0.777336 | 0.000111 |
| 19625 | Meiosin | 0.775993 | 6.49E-06 |
| 21416 | Mtss1 | 0.774883 | 3.37E-41 |
| 3634 | Camk2a | 0.771341 | 3.53E-07 |
| 22536 | Obscn | 0.770993 | 0.014048 |
| 24914 | Pnma8b | 0.769466 | 1.25E-14 |
| 7010 | Eya1 | 0.764971 | 9.41E-36 |
| 27950 | Slc30a10 | 0.763802 | 2.47E-05 |
| 29601 | Tenm4 | 0.761114 | 2.86E-26 |
| 1786 | Adamts15 | 0.759572 | 3.18E-25 |
| 21548 | Myl9 | 0.758762 | 3.05E-24 |
| 2916 | AW551984 | 0.758388 | 7.04E-42 |
| 31262 | Unc45b | 0.757818 | 0.000576 |
| 16090 | Gm9918 | 0.757023 | 0.001232 |
| 28997 | Steap2 | 0.755985 | 1.21E-18 |
| 23935 | Osr2 | 0.755157 | 5.95E-33 |
| 5559 | Cys1 | 0.752179 | 2.86E-06 |
| 5063 | Cped1 | 0.749533 | 5.94E-29 |
| 31269 | Unc5d | 0.749522 | 6.79E-11 |
| 29487 | Tcea3 | 0.748819 | 0.000231 |
| 29243 | Syt17 | 0.747295 | 0.021287 |
| 26442 | Rnf165 | 0.746497 | 2.23E-12 |
| 7180 | Fam228a | 0.746209 | 0.000615 |
| 1546 | Abcc6 | 0.74613 | 0.000986 |
| 29858 | Tln2 | 0.74537 | 1.03E-29 |
| 21786 | Ncam1 | 0.743703 | 2.63E-44 |
| 28999 | Steap4 | 0.743075 | 0.002295 |
| 32358 | Zan | 0.74278 | 0.00012 |
| 8099 | Glipr2 | 0.741759 | 4.64E-23 |
| 3477 | C1ql1 | 0.741477 | 0.000171 |
| 18698 | Ldb2 | 0.741287 | 5.51E-30 |
| 22160 | Nol3 | 0.737614 | 0.010879 |
| 4196 | Cdh10 | 0.737417 | 2.82E-09 |
| 22357 | Ntm | 0.736574 | 1.58E-07 |
| 16372 | Grid1 | 0.735278 | 1.58E-06 |
| 18729 | Lepr | 0.734486 | 4.32E-29 |
| 16841 | Heph | 0.734314 | 4.34E-33 |
| 6033 | Dlgap3 | 0.732034 | 0.002959 |
| 21114 | Moxd1 | 0.729782 | 4.21E-09 |
| 18008 | Jph4 | 0.727013 | 4.81E-15 |
| 21774 | Nav3 | 0.723814 | 2.26E-15 |
| 7571 | Fndc5 | 0.72272 | 2.76E-08 |
| 17663 | Il1b | 0.719555 | 5.02E-05 |
| 8085 | Glcci1 | 0.718518 | 1.23E-05 |
| 613 | 4833422M21Rik | 0.71767 | 0.017529 |
| 1875 | Adnp | 0.71617 | 0.002392 |
| 2648 | Asap3 | 0.715446 | 1.07E-18 |
| 4789 | Clstn2 | 0.7149 | 2.44E-18 |
| 31448 | Vegfd | 0.712917 | 1.17E-11 |
| 22340 | Nsun7 | 0.711203 | 0.003008 |
| 24674 | Pitpnm3 | 0.710647 | 9.49E-21 |
| 21032 | Mmd2 | 0.709804 | 0.000188 |
| 29864 | Tlr2 | 0.705769 | 6.19E-10 |
| 7254 | Fas | 0.704112 | 0.003817 |
| 3340 | Brinp1 | 0.703465 | 7.08E-09 |
| 24765 | Plcb1 | 0.702192 | 1.94E-18 |
| 27560 | Sfxn5 | 0.701995 | 3.02E-11 |
| 7923 | Gbp9 | 0.701029 | 9.84E-09 |
| 4456 | Cfap69 | 0.700446 | 3.36E-05 |
| 2628 | Arsi | 0.69871 | 9.53E-06 |
| 6809 | Epha5 | 0.698655 | 2.33E-07 |
| 22367 | Ntrk2 | 0.69849 | 2.02E-34 |
| 27378 | Sema3f | 0.698203 | 3.66E-29 |
| 25287 | Prex2 | 0.697449 | 5.08E-17 |
| 6930 | Esrrg | 0.69622 | 0.001006 |
| 29215 | Syne3 | 0.694586 | 2.99E-07 |
| 16329 | Gprin2 | 0.692411 | 1.32E-11 |
| 9635 | Gm17396 | 0.69212 | 1.20E-05 |
| 28557 | Sorbs2 | 0.691936 | 6.28E-06 |
| 25461 | Prrt4 | 0.691314 | 0.002457 |
| 29343 | Taok3 | 0.689865 | 5.97E-15 |
| 8057 | Gja5 | 0.688999 | 3.20E-05 |
| 4199 | Cdh13 | 0.688913 | 6.02E-16 |
| 16419 | Gsap | 0.687796 | 4.13E-08 |
| 27639 | Shfl | 0.687122 | 1.19E-11 |
| 24203 | Pcdhac2 | 0.687111 | 1.85E-07 |
| 21499 | Myb | 0.686873 | 1.68E-05 |
| 31421 | Vash1 | 0.68662 | 1.86E-17 |
| 25663 | Ptpdc1 | 0.686377 | 3.67E-22 |
| 18772 | Lhx8 | 0.686274 | 1.65E-34 |
| 4048 | Cd14 | 0.682151 | 6.80E-06 |
| 28176 | Slitrk5 | 0.681779 | 8.07E-07 |
| 26517 | Robo2 | 0.681314 | 1.75E-31 |
| 4488 | Ch25h | 0.679536 | 0.001287 |
| 28173 | Slitrk2 | 0.678009 | 0.000105 |
| 2767 | Atp10b | 0.677265 | 0.022898 |
| 17075 | Hs3st1 | 0.677116 | 2.25E-09 |
| 18580 | L1cam | 0.677107 | 2.68E-07 |
| 30087 | Tmem26 | 0.675624 | 1.93E-34 |
| 19733 | Mgat5 | 0.675405 | 4.93E-21 |
| 3638 | Camk2n1 | 0.673644 | 9.56E-08 |
| 7250 | Farp2 | 0.672547 | 1.92E-10 |
| 26402 | Rnasel | 0.669045 | 9.36E-18 |
| 5058 | Cpe | 0.668565 | 2.96E-28 |
| 7181 | Fam228b | 0.668407 | 5.69E-06 |
| 24288 | Pcx | 0.667995 | 6.37E-17 |
| 6497 | Edil3 | 0.665843 | 1.46E-14 |
| 3565 | Cachd1 | 0.665075 | 1.28E-36 |
| 26232 | Rgs22 | 0.665001 | 0.006832 |
| 28634 | Sparc | 0.664444 | 3.10E-30 |
| 3570 | Cacna1e | 0.663061 | 0.000284 |
| 29248 | Syt6 | 0.662135 | 0.000188 |
| 3205 | Bgn | 0.66205 | 7.88E-40 |
| 3994 | Ccnd1 | 0.65985 | 5.91E-29 |
| 32936 | Zkscan16 | 0.659241 | 2.80E-05 |
| 28091 | Slc6a17 | 0.658701 | 3.45E-18 |
| 7898 | Gata6 | 0.657154 | 2.12E-05 |
| 7108 | Fam13c | 0.655903 | 5.79E-10 |
| 4918 | Col13a1 | 0.654907 | 1.52E-17 |
| 13 | 0610040J01Rik | 0.653305 | 0.001073 |
| 24742 | Plag1 | 0.650977 | 1.27E-14 |
| 32054 | Vsnl1 | 0.650871 | 0.004084 |
| 2406 | Apod | 0.650819 | 5.44E-09 |
| 22302 | Nrk | 0.650765 | 1.65E-19 |
| 2239 | Ankrd12 | 0.649911 | 8.19E-21 |
| 19555 | Mdga1 | 0.649589 | 8.77E-13 |
| 2052 | Akr1b8 | 0.647311 | 1.28E-06 |
| 16928 | Hmga2 | 0.647116 | 2.15E-31 |
| 3142 | Bcl11a | 0.647105 | 7.04E-32 |
| 24052 | Pak5 | 0.646966 | 8.00E-05 |
| 4945 | Col5a3 | 0.646743 | 1.02E-05 |
| 29255 | Sytl4 | 0.644924 | 1.97E-09 |
| 3756 | Cav1 | 0.644388 | 1.05E-23 |
| 7058 | Fabp7 | 0.642816 | 0.005547 |
| 29322 | Tafa3 | 0.639836 | 0.004722 |
| 27480 | Serpinb9 | 0.639509 | 3.03E-05 |
| 19014 | Lrguk | 0.638819 | 8.52E-07 |
| 2515 | Arhgdib | 0.638196 | 1.74E-07 |
| 2970 | B3gat1 | 0.635344 | 2.67E-10 |
| 17654 | Il17rc | 0.634885 | 0.006784 |
| 24798 | Plekha6 | 0.634498 | 3.95E-09 |
| 17787 | Insyn1 | 0.634226 | 6.14E-15 |
| 25686 | Ptprd | 0.633211 | 1.15E-24 |
| 25466 | Prss12 | 0.632146 | 1.74E-22 |
| 18240 | Kif1a | 0.631812 | 5.35E-19 |
| 26992 | Rtn2 | 0.631056 | 2.17E-05 |
| 15047 | Gm6566 | 0.63066 | 0.003193 |
| 26240 | Rgs9 | 0.629119 | 4.34E-05 |
| 18809 | Lipa | 0.628089 | 1.01E-20 |
| 24114 | Parp8 | 0.625329 | 4.44E-23 |
| 28952 | St8sia2 | 0.625029 | 1.19E-24 |
| 7569 | Fndc3c1 | 0.624574 | 2.08E-21 |
| 2372 | Apc2 | 0.62454 | 6.70E-15 |
| 22145 | Nnt | 0.623103 | 0.021106 |
| 3448 | Bves | 0.622953 | 0.02145 |
| 9492 | Gm16201 | 0.621549 | 0.006767 |
| 32260 | Xist | 0.620649 | 4.66E-19 |
| 4562 | Chrd | 0.620271 | 7.49E-13 |
| 16604 | H2-T22 | 0.620256 | 2.29E-10 |
| 30762 | Trio | 0.619671 | 4.74E-21 |
| 5364 | Cux2 | 0.619283 | 2.48E-11 |
| 16897 | Hint3 | 0.618872 | 0.000632 |
| 16764 | Hbegf | 0.618716 | 6.92E-07 |
| 24217 | Pcdhb21 | 0.61863 | 6.81E-05 |
| 1797 | Adamts7 | 0.618429 | 3.82E-19 |
| 32800 | Zfp811 | 0.617445 | 1.37E-10 |
| 19453 | Mas1 | 0.616822 | 0.016956 |
| 6542 | Efhc1 | 0.616475 | 0.001346 |
| 6253 | Dpysl3 | 0.616092 | 5.08E-35 |
| 19492 | Mboat1 | 0.615305 | 8.18E-10 |
| 1377 | A330074K22Rik | 0.614929 | 4.68E-06 |
| 31201 | Ucn2 | 0.613979 | 0.006748 |
| 15612 | Gm8291 | 0.612834 | 0.000103 |
| 7738 | Fxyd2 | 0.612552 | 0.023423 |
| 7887 | Gas7 | 0.611825 | 1.07E-07 |
| 2964 | B3galt1 | 0.610971 | 2.88E-06 |
| 27391 | Sema6d | 0.610056 | 6.23E-24 |
| 3334 | Brdt | 0.609226 | 2.49E-05 |
| 6568 | Egln3 | 0.60897 | 4.24E-07 |
| 19412 | Mapk4 | 0.607325 | 0.000357 |
| 8018 | Ggta1 | 0.606378 | 2.83E-15 |
| 27031 | Rxfp3 | 0.606155 | 0.000136 |
| 9841 | Gm18189 | 0.604698 | 0.002753 |
| 2585 | Armc2 | 0.604354 | 1.38E-06 |
| 29466 | Tbx2 | 0.600279 | 7.84E-20 |
| 19339 | Man2a1 | 0.600024 | 4.94E-24 |
| 21929 | Neil3 | 0.599068 | 6.40E-16 |
| 24334 | Pde8b | 0.598847 | 0.002981 |
| 19459 | Mast4 | 0.598515 | 1.52E-15 |
| 5747 | Ddah1 | 0.598335 | 3.71E-19 |
| 9428 | Gm15806 | 0.596388 | 0.000988 |
| 24324 | Pde5a | 0.595528 | 2.35E-17 |
| 6106 | Dnah8 | 0.593494 | 2.27E-05 |
| 32411 | Zbtb8b | 0.593385 | 0.023536 |
| 951 | 4930570G19Rik | 0.592402 | 0.008501 |
| 17613 | Igsf6 | 0.592103 | 0.000453 |
| 28204 | Smarca2 | 0.591704 | 7.78E-32 |
| 21418 | Mttp | 0.5917 | 5.51E-06 |
| 28561 | Sorcs2 | 0.591146 | 6.87E-15 |
| 27092 | Samd5 | 0.588623 | 8.75E-20 |
| 30330 | Tox | 0.587792 | 1.53E-15 |
| 17127 | Hspa1a | 0.587694 | 3.97E-11 |
| 24873 | Plxna2 | 0.584707 | 5.10E-15 |
| 3698 | Car9 | 0.584109 | 0.000197 |
| 30829 | Trps1 | 0.583681 | 2.62E-23 |
| 7135 | Fam174b | 0.581584 | 1.91E-09 |
| 24580 | Pid1 | 0.581561 | 5.25E-15 |
| 6728 | Eml5 | 0.581406 | 0.000306 |
| 24669 | Pitpnc1 | 0.581036 | 6.34E-21 |
| 24844 | Plp1 | 0.579344 | 3.44E-07 |
| 27379 | Sema3g | 0.579103 | 9.77E-07 |
| 2214 | Angptl4 | 0.577656 | 4.41E-08 |
| 19633 | Meox1 | 0.577303 | 4.37E-05 |
| 32224 | Wnt5a | 0.575793 | 1.02E-25 |
| 29846 | Tle1 | 0.575204 | 2.98E-23 |
| 3837 | Ccdc15 | 0.574883 | 0.013577 |
| 18736 | Lfng | 0.57419 | 5.22E-06 |
| 19117 | Lrrn3 | 0.573253 | 2.91E-05 |
| 1820 | Adcy4 | 0.573007 | 4.26E-15 |
| 24911 | Pnma3 | 0.572294 | 0.005321 |
| 18158 | Kcnt2 | 0.571949 | 6.46E-05 |
| 6818 | Ephx1 | 0.571516 | 0.000443 |
| 16432 | Gsdmd | 0.571113 | 4.37E-09 |
| 24183 | Pcdh17 | 0.567884 | 1.54E-15 |
| 18425 | Kmt2c | 0.567853 | 4.69E-16 |
| 7625 | Foxo6 | 0.567453 | 0.00251 |
| 1958 | Agtr1a | 0.567233 | 0.001175 |
| 16882 | Hic1 | 0.567043 | 1.36E-11 |
| 16449 | Gsta4 | 0.566772 | 5.92E-13 |
| 27247 | Scn1a | 0.566524 | 0.000523 |
| 29598 | Tenm1 | 0.566038 | 8.07E-07 |
| 2452 | Arap2 | 0.565722 | 0.000114 |
| 3632 | Camk1d | 0.56572 | 7.71E-06 |
| 31258 | Unc13b | 0.564362 | 1.29E-09 |
| 24018 | Pabpc4l | 0.563986 | 1.59E-09 |
| 7428 | Fgd5 | 0.56378 | 5.26E-14 |
| 19000 | Lratd1 | 0.563163 | 2.24E-09 |
| 7670 | Frrs1l | 0.562697 | 1.27E-08 |
| 28995 | Stc2 | 0.562065 | 0.010138 |
| 4537 | Chl1 | 0.559577 | 0.000604 |
| 2381 | Aph1c | 0.558928 | 0.000859 |
| 6307 | Dtx1 | 0.558709 | 3.91E-09 |
| 2049 | Akr1b10 | 0.557517 | 0.000183 |
| 25952 | Rapgefl1 | 0.556246 | 1.60E-10 |
| 2721 | Atcay | 0.554779 | 0.006035 |
| 21544 | Myl4 | 0.554342 | 0.00049 |
| 4639 | Cited1 | 0.552958 | 0.000113 |
| 1960 | Agtr2 | 0.55066 | 1.22E-16 |
| 6081 | Dmxl2 | 0.550399 | 1.30E-10 |
| 27601 | Sh3bgrl2 | 0.549962 | 8.52E-10 |
| 25518 | Psd2 | 0.549314 | 0.000107 |
| 26046 | Rbm4 | 0.547415 | 0.000563 |
| 6720 | Emid1 | 0.54735 | 5.81E-09 |
| 19005 | Lrch3 | 0.54685 | 1.49E-11 |
| 29251 | Syt9 | 0.546706 | 2.20E-09 |
| 31340 | Usp2 | 0.546653 | 1.69E-08 |
| 17128 | Hspa1b | 0.544184 | 8.59E-11 |
| 19614 | Megf10 | 0.544103 | 5.67E-06 |
| 25991 | Rassf4 | 0.543832 | 1.60E-06 |
| 17616 | Igsf9b | 0.542727 | 2.42E-06 |
| 3432 | Btn2a2 | 0.542035 | 0.015478 |
| 24814 | Plekhh2 | 0.541258 | 2.16E-13 |
| 26176 | Rev3l | 0.540994 | 3.02E-19 |
| 3685 | Car11 | 0.540695 | 1.37E-06 |
| 26527 | Ror1 | 0.540653 | 2.15E-20 |
| 22277 | Nr4a3 | 0.540547 | 1.07E-10 |
| 25642 | Ptgs2 | 0.539557 | 0.001386 |
| 25761 | Pyroxd2 | 0.53886 | 7.23E-05 |
| 1456 | A930007A09Rik | 0.537332 | 0.010287 |
| 1508 | Aass | 0.533542 | 0.000242 |
| 28339 | Snhg11 | 0.533166 | 0.009142 |
| 16594 | H2-Q1 | 0.531476 | 0.01459 |
| 4030 | Ccrl2 | 0.530227 | 2.88E-05 |
| 26464 | Rnf213 | 0.52948 | 1.70E-09 |
| 19780 | Minar2 | 0.529357 | 3.21E-05 |
| 17892 | Islr2 | 0.526903 | 0.003333 |
| 14801 | Gm5815 | 0.526494 | 0.000172 |
| 25517 | Psd | 0.526141 | 5.10E-08 |
| 32988 | Zrsr1 | 0.525685 | 2.14E-10 |
| 2512 | Arhgap8 | 0.525546 | 0.007939 |
| 25924 | Ramp3 | 0.524606 | 0.006643 |
| 3267 | Bmper | 0.522643 | 1.33E-10 |
| 17845 | Iqsec1 | 0.520165 | 3.07E-17 |
| 4114 | Cd46 | 0.519722 | 0.000917 |
| 27390 | Sema6c | 0.519644 | 2.32E-12 |
| 22299 | Nrip1 | 0.519291 | 1.04E-13 |
| 18715 | Lef1 | 0.518387 | 1.35E-16 |
| 7797 | Gabrb3 | 0.517101 | 0.018849 |
| 29573 | Tead4 | 0.516829 | 0.000522 |
| 5075 | Cpne2 | 0.516037 | 1.10E-07 |
| 30351 | Tpm2 | 0.51591 | 7.19E-14 |
| 24865 | Plscr4 | 0.514974 | 6.93E-05 |
| 25460 | Prrt3 | 0.514885 | 0.019578 |
| 26225 | Rgs16 | 0.513785 | 1.68E-06 |
| 24107 | Parp12 | 0.513579 | 3.07E-07 |
| 21852 | Ndst3 | 0.513518 | 0.00061 |
| 16907 | Hivep2 | 0.513492 | 9.73E-08 |
| 26214 | Rgma | 0.513213 | 3.82E-11 |
| 18136 | Kcnma1 | 0.513076 | 0.000787 |
| 24665 | Pisd-ps1 | 0.512855 | 5.61E-11 |
| 17607 | Igsf10 | 0.51157 | 8.18E-14 |
| 21340 | Msrb3 | 0.511259 | 3.57E-15 |
| 3103 | BC051226 | 0.51116 | 0.010417 |
| 26251 | Rhbdl3 | 0.509214 | 2.68E-05 |
| 16330 | Gprin3 | 0.506797 | 0.000137 |
| 21947 | Nell2 | 0.506253 | 2.86E-09 |
| 1986 | AI504432 | 0.505965 | 0.000757 |
| 30094 | Tmem268 | 0.505592 | 1.31E-08 |
| 30160 | Tmem8b | 0.504778 | 1.92E-16 |
| 1736 | Acvr2b | 0.504466 | 4.14E-06 |
| 7691 | Fstl1 | 0.504137 | 4.18E-26 |
| 25053 | Pou6f1 | 0.502248 | 5.81E-14 |
| 24211 | Pcdhb16 | 0.501914 | 2.79E-05 |
| 22363 | Ntng1 | 0.501691 | 3.04E-10 |
| 18074 | Kcnab3 | 0.501576 | 0.000133 |
| 18829 | Lix1l | 0.500554 | 6.24E-21 |

**Table5. Upregulated DEGs of lingual part of tooth germs at bell stage than of buccal part**

|  | SYMBOL | log2FoldChange | pvalue |
| --- | --- | --- | --- |
| 16726 | Hand2os1 | -5.16943 | 2.03E-11 |
| 30892 | Tspan8 | -4.94538 | 4.49E-107 |
| 1988 | AI593442 | -4.73867 | 1.83E-55 |
| 16725 | Hand2 | -4.61295 | 1.38E-17 |
| 30458 | Trank1 | -4.55244 | 2.75E-78 |
| 17088 | Hsd11b1 | -4.12669 | 3.64E-63 |
| 30470 | Trappc3l | -3.99565 | 1.27E-06 |
| 16261 | Gpr149 | -3.94917 | 3.14E-29 |
| 23940 | Ostn | -3.92058 | 1.98E-33 |
| 21764 | Nat8f3 | -3.90464 | 1.50E-07 |
| 16111 | Gmnc | -3.89929 | 2.68E-06 |
| 27691 | Sim2 | -3.75597 | 2.79E-15 |
| 23999 | P2ry14 | -3.71711 | 2.49E-76 |
| 25697 | Ptprr | -3.6782 | 9.83E-89 |
| 24858 | Plppr5 | -3.46632 | 1.15E-113 |
| 4889 | Cntnap4 | -3.38732 | 3.95E-81 |
| 1959 | Agtr1b | -3.37681 | 4.34E-08 |
| 24781 | Plcxd3 | -3.3404 | 1.61E-13 |
| 9498 | Gm16223 | -3.31449 | 2.38E-05 |
| 5686 | Dbx1 | -3.28593 | 3.52E-05 |
| 24857 | Plppr4 | -3.25479 | 7.73E-125 |
| 2295 | Ano3 | -3.14894 | 4.72E-45 |
| 17968 | Iyd | -3.07307 | 9.67E-10 |
| 16827 | Hecw1 | -3.06382 | 2.65E-39 |
| 25773 | Qrfprl | -3.05555 | 6.63E-07 |
| 19635 | Mep1a | -3.02198 | 0.003797 |
| 5256 | Cst8 | -3.02195 | 0.003674 |
| 2683 | Asic2 | -2.98367 | 6.37E-11 |
| 5055 | Cpb1 | -2.98103 | 9.60E-10 |
| 27456 | Serpina6 | -2.97392 | 0.001003 |
| 13958 | Gm38684 | -2.95729 | 0.000106 |
| 7081 | Fam110c | -2.94309 | 9.63E-14 |
| 1859 | Adh1 | -2.93696 | 2.50E-05 |
| 22279 | Nr5a2 | -2.9137 | 2.14E-101 |
| 28088 | Slc6a14 | -2.84367 | 1.03E-07 |
| 9053 | Gm14267 | -2.83792 | 1.65E-34 |
| 1399 | A530021J07Rik | -2.83159 | 0.008637 |
| 4958 | Colec10 | -2.79331 | 7.13E-11 |
| 27826 | Slc1a7 | -2.76253 | 0.000104 |
| 17786 | Insrr | -2.75901 | 1.11E-59 |
| 5490 | Cyp2f2 | -2.75079 | 1.71E-23 |
| 5348 | Ctxnd1 | -2.64722 | 7.09E-144 |
| 16520 | Gucy2f | -2.62322 | 6.25E-06 |
| 6068 | Dmrta1 | -2.59067 | 5.24E-09 |
| 22539 | Oca2 | -2.57719 | 6.20E-05 |
| 1956 | Agt | -2.55145 | 3.44E-46 |
| 18144 | Kcnn2 | -2.49822 | 7.96E-28 |
| 17076 | Hs3st2 | -2.46403 | 2.50E-05 |
| 24907 | Pnliprp1 | -2.43323 | 0.032429 |
| 24146 | Pax3 | -2.4014 | 9.56E-27 |
| 6737 | En1 | -2.40061 | 1.59E-26 |
| 2706 | Astn1 | -2.38511 | 8.13E-134 |
| 24787 | Pld5 | -2.37304 | 1.04E-20 |
| 24313 | Pde1a | -2.36104 | 1.68E-127 |
| 19627 | Meis2 | -2.34564 | 8.72E-132 |
| 2878 | AU015836 | -2.3426 | 0.000215 |
| 7116 | Fam162b | -2.33862 | 6.75E-08 |
| 4198 | Cdh12 | -2.30814 | 2.34E-69 |
| 1845 | Adgrf4 | -2.29418 | 1.67E-08 |
| 18174 | Kctd19 | -2.29236 | 0.004275 |
| 24279 | Pcsk2 | -2.27766 | 2.05E-14 |
| 31455 | Vgll3 | -2.27716 | 2.95E-202 |
| 16810 | Hdnr | -2.26574 | 1.13E-05 |
| 5221 | Csmd3 | -2.25608 | 1.50E-34 |
| 30079 | Tmem252 | -2.24325 | 8.07E-202 |
| 27755 | Slamf8 | -2.23968 | 0.049821 |
| 4324 | Celrr | -2.23582 | 3.52E-05 |
| 27030 | Rxfp2 | -2.23135 | 6.81E-07 |
| 4885 | Cntn6 | -2.21245 | 2.70E-207 |
| 27524 | Sez6l | -2.20791 | 4.44E-40 |
| 5105 | Cr2 | -2.20444 | 1.09E-14 |
| 3204 | Bglap3 | -2.20381 | 9.50E-05 |
| 27041 | Ryr3 | -2.1866 | 4.87E-129 |
| 26074 | Rbp4 | -2.18578 | 2.34E-54 |
| 3724 | Casp12 | -2.18483 | 5.41E-33 |
| 5387 | Cxcl5 | -2.17308 | 5.03E-83 |
| 2078 | Aldh1a1 | -2.17144 | 1.66E-20 |
| 7789 | Gabra1 | -2.1705 | 4.14E-58 |
| 28586 | Sox2ot | -2.1653 | 5.47E-09 |
| 26226 | Rgs17 | -2.14901 | 1.55E-102 |
| 21768 | Nat8f7 | -2.13215 | 0.010433 |
| 32948 | Zmat4 | -2.10723 | 5.87E-48 |
| 18482 | Krt36 | -2.09044 | 7.25E-05 |
| 18150 | Kcnq3 | -2.0801 | 1.82E-16 |
| 21787 | Ncam2 | -2.06694 | 2.95E-41 |
| 28550 | Sod3 | -2.0628 | 2.36E-14 |
| 10482 | Gm20754 | -2.05816 | 0.002915 |
| 25159 | Ppp2r2b | -2.05433 | 3.01E-56 |
| 26428 | Rnf138rt1 | -2.04862 | 0.000322 |
| 5054 | Cpa6 | -2.04287 | 1.83E-41 |
| 18747 | Lgi1 | -2.03586 | 2.90E-29 |
| 17168 | Htr7 | -2.02722 | 1.50E-25 |
| 7104 | Fam135b | -2.02325 | 4.52E-18 |
| 28781 | Sprr1a | -2.02041 | 0.002051 |
| 24487 | Pgm5 | -2.00353 | 1.13E-184 |
| 6769 | Entpd1 | -1.98542 | 3.13E-170 |
| 2081 | Aldh1a7 | -1.98343 | 6.94E-08 |
| 8117 | Glrp1 | -1.97852 | 0.007867 |
| 3868 | Ccdc187 | -1.97583 | 3.88E-12 |
| 4524 | Chgb | -1.97562 | 2.66E-06 |
| 1759 | Adam23 | -1.96011 | 4.18E-104 |
| 2629 | Arsj | -1.94516 | 9.01E-62 |
| 17080 | Hs3st5 | -1.93902 | 4.22E-21 |
| 6101 | Dnah5 | -1.93614 | 0.006398 |
| 15659 | Gm8417 | -1.92238 | 0.009222 |
| 14438 | Gm4951 | -1.91899 | 0.000434 |
| 17922 | Itgax | -1.91233 | 7.72E-06 |
| 3597 | Cadm2 | -1.89743 | 3.37E-49 |
| 17251 | Ifi27l2a | -1.89642 | 3.07E-21 |
| 18484 | Krt4 | -1.89336 | 4.76E-70 |
| 21561 | Myo16 | -1.89102 | 4.02E-44 |
| 27579 | Sgpp2 | -1.87762 | 9.59E-18 |
| 32262 | Xkr4 | -1.87495 | 2.58E-12 |
| 17935 | Itgbl1 | -1.8733 | 1.48E-130 |
| 1439 | A830019L24Rik | -1.86694 | 9.97E-06 |
| 24083 | Pappa2 | -1.86279 | 2.63E-195 |
| 27238 | Scin | -1.85273 | 3.11E-22 |
| 19503 | Mc4r | -1.84576 | 1.70E-09 |
| 29709 | Tgfbi | -1.83883 | 9.30E-170 |
| 22474 | Oas1a | -1.81867 | 8.73E-12 |
| 30224 | Tnfrsf11b | -1.81733 | 5.91E-31 |
| 27478 | Serpinb7 | -1.80329 | 1.52E-08 |
| 1734 | Acvr1c | -1.79513 | 0.001577 |
| 1824 | Adcy8 | -1.7921 | 1.07E-45 |
| 2365 | Apba2 | -1.7904 | 3.55E-21 |
| 27446 | Serpina3f | -1.78636 | 6.18E-06 |
| 104 | 1700010K23Rik | -1.78516 | 0.008521 |
| 5993 | Dio2 | -1.77438 | 1.52E-21 |
| 28134 | Slc9a7 | -1.76204 | 7.66E-19 |
| 2275 | Ankrd55 | -1.7577 | 5.27E-05 |
| 10184 | Gm18981 | -1.75529 | 4.44E-07 |
| 29039 | Stmn4 | -1.74874 | 3.77E-08 |
| 26530 | Rorb | -1.74667 | 4.01E-17 |
| 28136 | Slc9a9 | -1.72548 | 6.66E-64 |
| 22482 | Oas2 | -1.72144 | 3.41E-18 |
| 22047 | Nipal1 | -1.71844 | 9.89E-12 |
| 32378 | Zbtb16 | -1.71263 | 1.68E-26 |
| 5182 | Cryba4 | -1.70792 | 0.000321 |
| 27186 | Scg2 | -1.68196 | 1.53E-64 |
| 3961 | Ccl21a | -1.67618 | 1.05E-73 |
| 5440 | Cyp26c1 | -1.67522 | 2.35E-60 |
| 28030 | Slc40a1 | -1.67232 | 2.23E-98 |
| 22120 | Nmbr | -1.66054 | 6.00E-07 |
| 6759 | Enpep | -1.65947 | 1.94E-62 |
| 1838 | Adgrd1 | -1.65267 | 2.12E-27 |
| 21489 | Mx1 | -1.64823 | 4.31E-05 |
| 22483 | Oas3 | -1.64404 | 0.008921 |
| 7435 | Fgf14 | -1.64307 | 2.18E-30 |
| 30861 | Tshr | -1.64137 | 7.83E-07 |
| 3884 | Ccdc3 | -1.64023 | 5.79E-127 |
| 24850 | Plpp4 | -1.63759 | 0.006686 |
| 6863 | Ereg | -1.6365 | 0.009528 |
| 27833 | Slc22a14 | -1.6348 | 0.008628 |
| 19233 | Lyve1 | -1.63208 | 9.42E-57 |
| 6108 | Dnai1 | -1.63071 | 8.75E-06 |
| 29773 | Thsd4 | -1.63056 | 8.51E-151 |
| 24441 | Pf4 | -1.60252 | 2.46E-42 |
| 14669 | Gm5431 | -1.6025 | 0.003801 |
| 4122 | Cd55os | -1.6007 | 0.002244 |
| 24066 | Pamr1 | -1.596 | 1.06E-50 |
| 25183 | Ppp4r4 | -1.59223 | 2.41E-47 |
| 2146 | Alx1 | -1.59073 | 2.40E-18 |
| 27762 | Slc10a4-ps | -1.59028 | 3.16E-05 |
| 3962 | Ccl21b | -1.58917 | 1.87E-16 |
| 19735 | Mgl2 | -1.58698 | 0.000201 |
| 6095 | Dnah11 | -1.58085 | 1.92E-05 |
| 17079 | Hs3st4 | -1.57832 | 0.000937 |
| 1803 | Adamtsl4 | -1.57829 | 1.21E-40 |
| 21033 | Mme | -1.5717 | 1.30E-123 |
| 6683 | Elmod1 | -1.57092 | 1.43E-12 |
| 8258 | Gm10591 | -1.56917 | 8.89E-14 |
| 27053 | S100a7a | -1.56586 | 6.18E-07 |
| 3963 | Ccl21d | -1.55983 | 3.16E-14 |
| 4051 | Cd163 | -1.55806 | 2.05E-09 |
| 24078 | Papln | -1.55797 | 1.16E-45 |
| 30359 | Tppp | -1.55793 | 2.93E-22 |
| 6016 | Dkk2 | -1.55253 | 1.93E-146 |
| 24775 | Plch1 | -1.55154 | 1.06E-19 |
| 27057 | S100b | -1.54637 | 6.44E-47 |
| 29040 | Stmnd1 | -1.54152 | 0.000684 |
| 28605 | Sp5 | -1.5405 | 1.07E-14 |
| 22574 | Ogn | -1.53828 | 1.57E-112 |
| 4202 | Cdh17 | -1.53365 | 4.92E-33 |
| 17907 | Itga11 | -1.53152 | 5.66E-77 |
| 27316 | Sec1 | -1.52404 | 1.10E-11 |
| 21852 | Ndst3 | -1.52165 | 3.27E-40 |
| 19556 | Mdga2 | -1.5135 | 7.31E-42 |
| 27377 | Sema3e | -1.51031 | 2.82E-51 |
| 5102 | Cpxm2 | -1.50666 | 1.37E-08 |
| 22449 | Nwd2 | -1.50273 | 8.47E-06 |
| 7809 | Gad1os | -1.49972 | 0.000133 |
| 32360 | Zar1 | -1.49266 | 4.55E-07 |
| 22305 | Nrn1 | -1.49238 | 2.79E-09 |
| 1502 | Aard | -1.49184 | 8.65E-25 |
| 13561 | Gm32391 | -1.49072 | 0.006888 |
| 7601 | Foxf1 | -1.48969 | 3.42E-90 |
| 8834 | Gm13304 | -1.48769 | 5.94E-13 |
| 4953 | Col8a1 | -1.48276 | 1.66E-101 |
| 5438 | Cyp26a1 | -1.47928 | 5.33E-30 |
| 7199 | Fam43a | -1.47509 | 1.90E-57 |
| 19779 | Minar1 | -1.47276 | 3.55E-09 |
| 7446 | Fgf4 | -1.46836 | 0.000226 |
| 29775 | Thsd7b | -1.46413 | 2.80E-19 |
| 27002 | Rtp4 | -1.4579 | 4.36E-15 |
| 29621 | Tesc | -1.45027 | 2.14E-06 |
| 27040 | Ryr2 | -1.44917 | 3.07E-26 |
| 5376 | Cxcl1 | -1.44755 | 4.25E-52 |
| 2148 | Alx4 | -1.44115 | 1.33E-48 |
| 29166 | Sv2b | -1.43792 | 2.20E-13 |
| 25976 | Rasgrp1 | -1.43392 | 1.09E-12 |
| 7613 | Foxl1 | -1.43357 | 4.13E-05 |
| 7022 | F13a1 | -1.43097 | 9.26E-88 |
| 3801 | Ccbe1 | -1.42818 | 2.84E-48 |
| 19607 | Medag | -1.42607 | 2.30E-10 |
| 4121 | Cd55b | -1.42579 | 0.00535 |
| 1645 | Ackr4 | -1.42369 | 5.81E-35 |
| 22235 | Npr3 | -1.4219 | 1.03E-64 |
| 19736 | Mgll | -1.41648 | 6.46E-55 |
| 16786 | Hcrtr2 | -1.413 | 0.001321 |
| 2086 | Aldh3a1 | -1.41179 | 0.002451 |
| 25293 | Prickle1 | -1.40921 | 5.76E-100 |
| 19729 | Mgat4c | -1.4078 | 4.35E-11 |
| 28336 | Sned1 | -1.40677 | 5.60E-90 |
| 4142 | Cd86 | -1.40493 | 3.00E-11 |
| 3341 | Brinp2 | -1.39606 | 0.002319 |
| 29505 | Tcf24 | -1.39175 | 1.67E-17 |
| 27951 | Slc30a2 | -1.39 | 3.69E-05 |
| 22031 | Niban1 | -1.38918 | 7.43E-92 |
| 21769 | Nat8l | -1.38672 | 3.37E-20 |
| 22480 | Oas1g | -1.38548 | 0.001309 |
| 21909 | Necab1 | -1.38412 | 6.44E-18 |
| 4904 | Coch | -1.37456 | 9.66E-09 |
| 2782 | Atp1b1 | -1.37316 | 3.39E-49 |
| 5350 | Cubn | -1.37049 | 9.50E-27 |
| 27373 | Sema3a | -1.36761 | 1.96E-98 |
| 2442 | Aqp4 | -1.367 | 4.23E-08 |
| 29750 | Them5 | -1.36666 | 6.72E-05 |
| 3258 | Bmp2 | -1.36276 | 3.11E-38 |
| 13751 | Gm34911 | -1.35084 | 0.000223 |
| 21155 | Mrc1 | -1.35004 | 2.26E-85 |
| 18309 | Klf9 | -1.34938 | 8.29E-54 |
| 3904 | Ccdc60 | -1.34919 | 0.002439 |
| 21730 | Nalf1 | -1.3488 | 6.86E-06 |
| 7402 | Fendrr | -1.34787 | 9.80E-106 |
| 27906 | Slc25a48 | -1.34588 | 8.81E-05 |
| 4120 | Cd55 | -1.3445 | 7.06E-30 |
| 29175 | Svip | -1.34415 | 2.56E-16 |
| 6748 | Enho | -1.34124 | 1.40E-25 |
| 32885 | Zfp979 | -1.3394 | 0.000259 |
| 4233 | Cdk15 | -1.32856 | 1.33E-05 |
| 6750 | Enkur | -1.32729 | 0.004107 |
| 7652 | Frem2 | -1.32668 | 7.36E-84 |
| 25023 | Postn | -1.32595 | 4.02E-110 |
| 22485 | Oasl2 | -1.32521 | 3.03E-15 |
| 27927 | Slc27a6 | -1.32274 | 1.49E-23 |
| 21945 | Nell1 | -1.32065 | 2.11E-34 |
| 16279 | Gpr176 | -1.32038 | 6.00E-11 |
| 22181 | Nos1 | -1.31991 | 2.05E-12 |
| 18156 | Kcns3 | -1.31926 | 1.50E-12 |
| 23930 | Osgin1 | -1.31632 | 7.59E-06 |
| 32458 | Zdhhc14 | -1.31531 | 7.11E-22 |
| 1676 | Acpp | -1.31206 | 4.91E-29 |
| 19269 | Maf | -1.31101 | 1.68E-78 |
| 16403 | Grm3 | -1.30789 | 0.000252 |
| 3954 | Ccl12 | -1.29888 | 3.09E-08 |
| 17067 | Hrh2 | -1.29838 | 0.011123 |
| 17940 | Itih5 | -1.29835 | 3.64E-62 |
| 8062 | Gjb3 | -1.29818 | 2.51E-06 |
| 4799 | Clvs2 | -1.29706 | 0.006092 |
| 17239 | Ifi202b | -1.29397 | 2.21E-22 |
| 7796 | Gabrb2 | -1.29342 | 5.05E-09 |
| 1698 | Acss3 | -1.28746 | 2.86E-30 |
| 32409 | Zbtb7c | -1.27863 | 4.82E-27 |
| 4730 | Clec2g | -1.27407 | 0.009349 |
| 25614 | Ptchd1 | -1.27142 | 2.73E-05 |
| 21195 | Mro | -1.26952 | 4.97E-05 |
| 4554 | Chodl | -1.26692 | 3.75E-82 |
| 29721 | Tgm2 | -1.26365 | 1.26E-48 |
| 3965 | Ccl24 | -1.26104 | 1.46E-05 |
| 19504 | Mc5r | -1.25172 | 2.73E-23 |
| 28780 | Sprn | -1.24762 | 2.48E-06 |
| 3137 | Bche | -1.24694 | 2.64E-44 |
| 29321 | Tafa2 | -1.24431 | 6.46E-15 |
| 17321 | Igf1 | -1.2431 | 1.55E-75 |
| 16770 | Hcar1 | -1.24093 | 0.009498 |
| 17241 | Ifi204 | -1.24046 | 3.88E-06 |
| 7463 | Fgl2 | -1.23469 | 4.28E-15 |
| 16942 | Hmgcs2 | -1.23367 | 3.57E-55 |
| 7586 | Fosl2 | -1.23295 | 5.25E-76 |
| 28960 | Stac | -1.23167 | 6.36E-30 |
| 25070 | Ppef2 | -1.23153 | 0.005907 |
| 26238 | Rgs7bp | -1.22568 | 7.51E-22 |
| 8015 | Ggt5 | -1.2203 | 6.01E-18 |
| 25386 | Prokr1 | -1.2178 | 2.49E-29 |
| 17247 | Ifi211 | -1.21724 | 3.84E-05 |
| 1974 | Ahrr | -1.21494 | 8.66E-34 |
| 29130 | Sult4a1 | -1.21433 | 9.12E-08 |
| 1894 | Adrb2 | -1.21427 | 3.11E-10 |
| 3690 | Car2 | -1.21301 | 1.25E-47 |
| 32217 | Wnt11 | -1.21153 | 7.99E-37 |
| 7992 | Gfod1 | -1.2106 | 2.34E-28 |
| 17617 | Igtp | -1.20869 | 1.20E-13 |
| 30710 | Trim30a | -1.2071 | 1.37E-09 |
| 4578 | Chrna7 | -1.196 | 3.19E-09 |
| 27262 | Scnn1g | -1.19194 | 5.82E-06 |
| 2332 | Aox3 | -1.19162 | 9.60E-19 |
| 29031 | Stk38l | -1.18364 | 1.23E-32 |
| 17679 | Il21r | -1.18203 | 4.91E-08 |
| 26340 | Rims4 | -1.17955 | 0.000213 |
| 26983 | Rtl3 | -1.1767 | 2.22E-73 |
| 27348 | Sectm1b | -1.17628 | 0.007129 |
| 30188 | Tmprss15 | -1.17422 | 0.005847 |
| 7380 | Fcna | -1.17337 | 6.00E-05 |
| 16362 | Grem1 | -1.1673 | 5.29E-21 |
| 16939 | Hmgcll1 | -1.16564 | 1.36E-11 |
| 16913 | Hk3 | -1.16347 | 2.13E-13 |
| 2023 | Ak8 | -1.16305 | 0.005312 |
| 6194 | Dock3 | -1.16149 | 3.11E-08 |
| 22439 | Nupr1 | -1.15989 | 2.76E-09 |
| 2698 | Aspn | -1.14879 | 7.90E-63 |
| 19206 | Lynx1 | -1.14699 | 1.97E-15 |
| 21060 | Mmrn1 | -1.14459 | 1.24E-35 |
| 3623 | Calml3 | -1.14319 | 8.14E-08 |
| 16436 | Gsg1l | -1.14241 | 4.77E-22 |
| 30212 | Tnc | -1.1408 | 1.28E-73 |
| 25062 | Ppargc1a | -1.14059 | 2.18E-10 |
| 19544 | Mctp1 | -1.13801 | 2.25E-11 |
| 3436 | Btnl2 | -1.13393 | 0.002027 |
| 22276 | Nr4a2 | -1.1335 | 3.36E-44 |
| 17083 | Hs6st2 | -1.1328 | 1.67E-57 |
| 17244 | Ifi207 | -1.12884 | 3.80E-26 |
| 16407 | Grm7 | -1.11978 | 0.006613 |
| 22350 | Nt5e | -1.11801 | 4.95E-44 |
| 4903 | Cobll1 | -1.11541 | 3.07E-30 |
| 24736 | Plaat3 | -1.11473 | 4.28E-16 |
| 27491 | Serpine2 | -1.11332 | 2.85E-76 |
| 24276 | Pcp4l1 | -1.11325 | 7.89E-12 |
| 21702 | Naaa | -1.10827 | 6.63E-37 |
| 27166 | Scara5 | -1.10581 | 2.27E-43 |
| 18466 | Krt19 | -1.10496 | 4.87E-13 |
| 3615 | Calhm4 | -1.10077 | 1.00E-07 |
| 23997 | P2ry12 | -1.09904 | 2.60E-07 |
| 23998 | P2ry13 | -1.096 | 3.52E-06 |
| 6232 | Dpp10 | -1.09553 | 0.002681 |
| 6322 | Dusp10 | -1.09257 | 1.47E-35 |
| 24741 | Plac9 | -1.08612 | 3.13E-21 |
| 5439 | Cyp26b1 | -1.08302 | 1.27E-51 |
| 2503 | Arhgap36 | -1.08146 | 5.26E-07 |
| 19322 | Mal2 | -1.07974 | 0.003294 |
| 8061 | Gjb2 | -1.07965 | 0.00232 |
| 3975 | Ccl7 | -1.07686 | 7.17E-07 |
| 3984 | Ccn5 | -1.07607 | 0.007452 |
| 16368 | Gria1 | -1.07529 | 1.87E-49 |
| 29641 | Tex14 | -1.07323 | 2.76E-06 |
| 4598 | Chst7 | -1.07197 | 1.37E-08 |
| 7599 | Foxe1 | -1.06918 | 0.003736 |
| 26052 | Rbm46 | -1.06634 | 4.49E-06 |
| 4718 | Clec10a | -1.06515 | 4.30E-05 |
| 3723 | Casp1 | -1.06448 | 7.04E-05 |
| 3041 | Batf | -1.06411 | 1.10E-10 |
| 3982 | Ccn3 | -1.06263 | 1.82E-09 |
| 7767 | G630016G05Rik | -1.06242 | 0.002917 |
| 17089 | Hsd11b2 | -1.06025 | 1.92E-07 |
| 23754 | Or8b53 | -1.05638 | 0.008982 |
| 17092 | Hsd17b11 | -1.05435 | 5.25E-29 |
| 4218 | Cdhr1 | -1.05313 | 6.44E-07 |
| 24468 | Pgap6 | -1.05261 | 1.26E-17 |
| 24936 | Podn | -1.05024 | 9.73E-33 |
| 28598 | Sp100 | -1.04906 | 6.51E-07 |
| 6937 | Etaa1os | -1.04461 | 0.001595 |
| 16914 | Hkdc1 | -1.04372 | 0.000922 |
| 16122 | Gna15 | -1.04233 | 3.19E-05 |
| 28915 | Sstr4 | -1.0416 | 2.97E-12 |
| 27354 | Selenbp1 | -1.03972 | 1.05E-18 |
| 6760 | Enpp1 | -1.03969 | 4.06E-34 |
| 3616 | Calhm5 | -1.03869 | 4.79E-08 |
| 18817 | Lipm | -1.03564 | 0.007061 |
| 4307 | Cebpd | -1.03059 | 3.90E-12 |
| 19396 | Map6d1 | -1.02994 | 0.000899 |
| 31224 | Ugt1a7c | -1.02972 | 0.001111 |
| 24088 | Paqr5 | -1.0277 | 0.000215 |
| 32495 | Zfhx4 | -1.02577 | 7.39E-60 |
| 7594 | Foxd1 | -1.02486 | 1.26E-06 |
| 24743 | Plagl1 | -1.01996 | 4.14E-66 |
| 26321 | Ric3 | -1.01983 | 2.13E-08 |
| 24082 | Pappa | -1.01882 | 2.32E-20 |
| 29870 | Tlr8 | -1.01871 | 0.003687 |
| 14370 | Gm4841 | -1.01868 | 0.001179 |
| 5802 | Ddx60 | -1.01827 | 0.000157 |
| 27052 | S100a6 | -1.01643 | 2.77E-35 |
| 22367 | Ntrk2 | -1.01395 | 1.15E-60 |
| 17871 | Irgm2 | -1.00921 | 2.74E-14 |
| 27015 | Runx1 | -1.00684 | 1.98E-30 |
| 22625 | Omd | -1.00626 | 3.14E-43 |
| 17646 | Il16 | -1.00445 | 1.13E-15 |
| 7628 | Foxp2 | -0.99912 | 8.01E-11 |
| 24516 | Phf11d | -0.99404 | 0.001671 |
| 7388 | Fcrls | -0.9919 | 1.12E-29 |
| 19121 | Lrrtm2 | -0.99137 | 1.18E-13 |
| 21711 | Nacad | -0.99103 | 7.91E-11 |
| 28242 | Smim1 | -0.98991 | 1.15E-16 |
| 3774 | Cbln2 | -0.98947 | 2.06E-06 |
| 29871 | Tlr9 | -0.98856 | 0.004851 |
| 8100 | Glis1 | -0.98821 | 2.44E-12 |
| 25768 | Qpct | -0.98765 | 5.97E-14 |
| 17240 | Ifi203 | -0.98736 | 2.40E-17 |
| 16297 | Gpr34 | -0.98702 | 0.000698 |
| 24498 | Phactr3 | -0.98652 | 5.20E-05 |
| 21490 | Mx2 | -0.98469 | 1.14E-06 |
| 2829 | Atp6v0e2 | -0.98061 | 8.11E-13 |
| 2161 | Amer2 | -0.97596 | 4.34E-12 |
| 27280 | Scube2 | -0.97542 | 7.18E-06 |
| 18460 | Krt13 | -0.973 | 2.52E-38 |
| 32002 | Vnn1 | -0.97245 | 0.01087 |
| 29111 | Sulf2 | -0.97108 | 1.39E-62 |
| 26086 | Rcan2 | -0.96956 | 9.65E-17 |
| 5994 | Dio3 | -0.96546 | 1.09E-29 |
| 1792 | Adamts20 | -0.95951 | 1.55E-39 |
| 27633 | Shc4 | -0.95881 | 1.66E-07 |
| 21307 | Ms4a6b | -0.9555 | 2.38E-12 |
| 3959 | Ccl2 | -0.94987 | 4.71E-11 |
| 1533 | Abcb4 | -0.94543 | 0.001287 |
| 26990 | Rtl9 | -0.94376 | 2.19E-13 |
| 32219 | Wnt2 | -0.9409 | 1.90E-10 |
| 17050 | Hpgds | -0.93917 | 3.13E-15 |
| 17886 | Isg15 | -0.93615 | 0.005506 |
| 4882 | Cntn3 | -0.93611 | 3.65E-32 |
| 2142 | Alpl | -0.93228 | 3.39E-56 |
| 5109 | Cracdl | -0.93108 | 4.77E-12 |
| 4663 | Clca2 | -0.92494 | 2.36E-05 |
| 30951 | Ttc39b | -0.92378 | 1.75E-26 |
| 3032 | Bank1 | -0.92317 | 0.002625 |
| 28502 | Sntg2 | -0.92289 | 6.31E-08 |
| 3521 | C3ar1 | -0.92249 | 1.71E-14 |
| 24063 | Pam | -0.91916 | 3.19E-44 |
| 7040 | F830016B08Rik | -0.9187 | 0.003725 |
| 30813 | Trpc3 | -0.91835 | 4.16E-22 |
| 19104 | Lrrc8e | -0.91567 | 4.89E-05 |
| 4795 | Clu | -0.91493 | 3.32E-19 |
| 17269 | Ifitm5 | -0.91411 | 1.00E-05 |
| 27782 | Slc13a5 | -0.91333 | 0.002965 |
| 6286 | Dsg1a | -0.91285 | 2.99E-11 |
| 21503 | Mybpc1 | -0.91275 | 1.12E-26 |
| 21069 | Mndal | -0.91221 | 2.81E-06 |
| 27860 | Slc24a2 | -0.90875 | 0.007154 |
| 24470 | Pgbd5 | -0.90842 | 7.65E-12 |
| 21592 | Myorg | -0.90791 | 2.88E-15 |
| 26967 | Rspo3 | -0.907 | 4.11E-19 |
| 2089 | Aldh3b2 | -0.90568 | 0.002455 |
| 19053 | Lrrc25 | -0.90417 | 1.00E-05 |
| 17772 | Inpp5j | -0.90278 | 0.008015 |
| 27355 | Selenbp2 | -0.9024 | 0.000256 |
| 1865 | Adh7 | -0.90216 | 0.001235 |
| 24563 | Phyhip | -0.90177 | 0.008917 |
| 22362 | Ntn5 | -0.90001 | 2.33E-05 |
| 21729 | Nalcn | -0.89925 | 6.47E-18 |
| 3598 | Cadm3 | -0.8992 | 8.69E-36 |
| 7216 | Fam83b | -0.89896 | 2.15E-07 |
| 16349 | Gramd1c | -0.89662 | 4.57E-21 |
| 3157 | Bcl3 | -0.89592 | 4.45E-10 |
| 32252 | Xaf1 | -0.89582 | 6.02E-06 |
| 3025 | Baiap2l1 | -0.89405 | 4.78E-13 |
| 18749 | Lgi3 | -0.89058 | 3.63E-09 |
| 24358 | Pdk4 | -0.88947 | 1.39E-13 |
| 25824 | Rab32 | -0.88935 | 1.35E-14 |
| 2267 | Ankrd44 | -0.88779 | 1.17E-43 |
| 7543 | Fmn2 | -0.88616 | 2.11E-18 |
| 6344 | Dusp8 | -0.88525 | 8.88E-16 |
| 7213 | Fam81a | -0.88413 | 3.23E-08 |
| 2074 | Albfm1 | -0.884 | 0.003501 |
| 18692 | Lcp1 | -0.88096 | 6.89E-31 |
| 5643 | Dab2 | -0.87976 | 3.36E-41 |
| 26158 | Reps2 | -0.87967 | 5.77E-23 |
| 6811 | Epha7 | -0.87858 | 2.12E-34 |
| 5647 | Dact1 | -0.87782 | 3.60E-27 |
| 29291 | Tacstd2 | -0.87626 | 2.29E-09 |
| 21043 | Mmp16 | -0.87551 | 1.49E-35 |
| 25143 | Ppp1r3b | -0.87293 | 3.61E-38 |
| 1642 | Ackr1 | -0.87289 | 1.25E-10 |
| 31338 | Usp18 | -0.87249 | 0.002137 |
| 16606 | H2-T24 | -0.87227 | 0.001887 |
| 26236 | Rgs6 | -0.87203 | 5.51E-05 |
| 27619 | Sh3rf2 | -0.87124 | 5.89E-14 |
| 28165 | Slfn8 | -0.87112 | 0.000826 |
| 6807 | Epha3 | -0.87107 | 5.23E-40 |
| 29157 | Susd2 | -0.86774 | 2.00E-08 |
| 27004 | Rttn | -0.86508 | 3.42E-34 |
| 1340 | 9930111J21Rik2 | -0.86356 | 0.000537 |
| 22475 | Oas1b | -0.86346 | 0.003153 |
| 21302 | Ms4a4a | -0.86295 | 0.0012 |
| 2330 | Aox1 | -0.86277 | 1.19E-07 |
| 2322 | Aoah | -0.86207 | 0.004187 |
| 19387 | Map3k8 | -0.8614 | 7.42E-09 |
| 1914 | Aff1 | -0.86132 | 4.85E-23 |
| 18348 | Klhl4 | -0.86132 | 2.91E-21 |
| 27472 | Serpinb5 | -0.86078 | 8.69E-13 |
| 25993 | Rassf6 | -0.86053 | 0.011331 |
| 30264 | Tnmd | -0.86002 | 1.19E-20 |
| 4712 | Cldn6 | -0.85811 | 3.81E-16 |
| 7998 | Gfra3 | -0.85778 | 1.63E-11 |
| 17866 | Irf7 | -0.85754 | 1.29E-06 |
| 22224 | Npl | -0.85691 | 1.28E-14 |
| 6487 | Eda2r | -0.85642 | 0.000333 |
| 24407 | Peli2 | -0.85521 | 1.73E-26 |
| 24768 | Plcb4 | -0.8529 | 1.56E-34 |
| 28151 | Slco5a1 | -0.85285 | 3.57E-12 |
| 6315 | Duoxa1 | -0.85114 | 0.000332 |
| 2604 | Arnt2 | -0.85079 | 7.75E-32 |
| 23914 | Osbp2 | -0.84924 | 0.000253 |
| 29619 | Tes | -0.849 | 6.40E-25 |
| 6458 | Ebf1 | -0.84889 | 1.07E-36 |
| 30187 | Tmprss13 | -0.84854 | 1.76E-07 |
| 1513 | Abca1 | -0.84524 | 9.32E-37 |
| 3476 | C1qc | -0.84136 | 2.33E-22 |
| 4256 | Cdkl2 | -0.84118 | 0.000259 |
| 1839 | Adgre1 | -0.83947 | 6.26E-15 |
| 18758 | Lhfp | -0.8385 | 4.63E-29 |
| 21917 | Nectin4 | -0.83843 | 4.62E-17 |
| 25471 | Prss23 | -0.83723 | 5.08E-27 |
| 25630 | Ptger4 | -0.83683 | 1.14E-06 |
| 21908 | Nebl | -0.83462 | 1.78E-11 |
| 19545 | Mctp2 | -0.8346 | 0.001939 |
| 1915 | Aff2 | -0.8339 | 9.02E-18 |
| 1561 | Abcg5 | -0.83208 | 2.69E-05 |
| 28898 | Ssc5d | -0.83076 | 5.41E-22 |
| 28759 | Spock2 | -0.8301 | 6.71E-33 |
| 1781 | Adamts1 | -0.82974 | 1.09E-37 |
| 21703 | Naalad2 | -0.82703 | 7.53E-23 |
| 1783 | Adamts12 | -0.82393 | 1.38E-33 |
| 23992 | P2rx6 | -0.82275 | 0.001726 |
| 3253 | Bmf | -0.82263 | 9.13E-25 |
| 24880 | Plxnc1 | -0.82239 | 6.04E-19 |
| 30179 | Tmprss11a | -0.82208 | 4.23E-05 |
| 30823 | Trpm3 | -0.8182 | 3.23E-07 |
| 2485 | Arhgap20 | -0.81576 | 6.69E-21 |
| 21310 | Ms4a7 | -0.81529 | 7.30E-05 |
| 6877 | Erich2 | -0.815 | 0.001757 |
| 5664 | Dapl1 | -0.81498 | 0.000218 |
| 29770 | Thrb | -0.8138 | 1.62E-05 |
| 5374 | Cx3cr1 | -0.81257 | 4.25E-25 |
| 4195 | Cdh1 | -0.81204 | 7.03E-26 |
| 21308 | Ms4a6c | -0.81196 | 8.34E-07 |
| 6462 | Ebi3 | -0.81191 | 0.003513 |
| 7927 | Gca | -0.81076 | 0.004957 |
| 1154 | 5430421F17Rik | -0.80963 | 3.03E-09 |
| 7494 | Firre | -0.80958 | 1.11E-36 |
| 6326 | Dusp14 | -0.80911 | 3.04E-07 |
| 5943 | Dgki | -0.80649 | 0.000149 |
| 3283 | Bnipl | -0.80531 | 0.000202 |
| 18160 | Kcnv1 | -0.80285 | 0.00023 |
| 29533 | Tcp11l2 | -0.80162 | 1.52E-13 |
| 6287 | Dsg1b | -0.80104 | 0.000206 |
| 24415 | Per2 | -0.79929 | 1.60E-20 |
| 27095 | Samd9l | -0.79896 | 7.11E-10 |
| 7975 | Gem | -0.7985 | 1.45E-29 |
| 27165 | Scara3 | -0.79845 | 2.81E-29 |
| 16366 | Grhl3 | -0.79842 | 9.69E-07 |
| 25504 | Prss8 | -0.79828 | 0.000143 |
| 29625 | Tesl1 | -0.79609 | 0.000168 |
| 3819 | Ccdc122 | -0.79602 | 0.005897 |
| 3640 | Camk4 | -0.79489 | 1.10E-06 |
| 5060 | Cpeb2 | -0.79457 | 5.26E-12 |
| 7176 | Fam222a | -0.79374 | 3.16E-08 |
| 18805 | Lingo2 | -0.79268 | 4.49E-05 |
| 6840 | Epsti1 | -0.79245 | 0.006274 |
| 22314 | Nrxn1 | -0.79184 | 3.22E-08 |
| 7917 | Gbp3 | -0.79078 | 0.002279 |
| 21336 | Msr1 | -0.79015 | 6.40E-10 |
| 17255 | Ifi44 | -0.78952 | 0.000498 |
| 19172 | Luzp2 | -0.78925 | 4.56E-08 |
| 30814 | Trpc4 | -0.78769 | 5.18E-11 |
| 22084 | Nkx3-1 | -0.78718 | 0.000232 |
| 2167 | Amigo2 | -0.78625 | 4.92E-09 |
| 6486 | Eda | -0.78456 | 1.57E-18 |
| 19762 | Mid2 | -0.78162 | 8.59E-23 |
| 7371 | Fcgr2b | -0.78103 | 6.84E-06 |
| 25755 | Pygl | -0.78059 | 2.89E-22 |
| 5944 | Dgkk | -0.77784 | 3.62E-05 |
| 18163 | Kctd1 | -0.77772 | 5.78E-27 |
| 7648 | Fras1 | -0.77643 | 2.53E-31 |
| 1887 | Adra1a | -0.77526 | 0.000108 |
| 16739 | Has2os | -0.77372 | 0.002631 |
| 2792 | Atp2b3 | -0.77311 | 0.003072 |
| 4563 | Chrdl1 | -0.77279 | 3.91E-16 |
| 29112 | Sult1a1 | -0.77245 | 3.72E-07 |
| 27261 | Scnn1b | -0.77197 | 0.003864 |
| 3474 | C1qb | -0.77188 | 2.55E-17 |
| 28032 | Slc41a2 | -0.77117 | 4.95E-08 |
| 5995 | Dio3os | -0.77062 | 1.13E-09 |
| 18855 | Lmo4 | -0.76755 | 5.20E-28 |
| 3367 | Bst2 | -0.76752 | 2.13E-08 |
| 26358 | Ripor3 | -0.76718 | 4.01E-15 |
| 6290 | Dsg3 | -0.76651 | 4.76E-11 |
| 7607 | Foxi3 | -0.76582 | 2.23E-09 |
| 27364 | Selenop | -0.76519 | 2.24E-31 |
| 32769 | Zfp750 | -0.7624 | 6.02E-12 |
| 17061 | Hr | -0.76213 | 3.11E-14 |
| 27803 | Slc16a9 | -0.76212 | 6.98E-05 |
| 16828 | Hecw2 | -0.76174 | 3.20E-18 |
| 29934 | Tmem106a | -0.76126 | 8.42E-09 |
| 19016 | Lrig1 | -0.7608 | 2.06E-27 |
| 6279 | Dsc2 | -0.76068 | 9.49E-18 |
| 26412 | Rnf112 | -0.7606 | 5.64E-08 |
| 17650 | Il17d | -0.76045 | 1.03E-10 |
| 7476 | Fhl2 | -0.75739 | 2.48E-17 |
| 3173 | Bdh1 | -0.75705 | 3.11E-13 |
| 29677 | Tfap2a | -0.75609 | 1.29E-11 |
| 18333 | Klhl23 | -0.75568 | 6.18E-27 |
| 24002 | P2ry6 | -0.75567 | 1.27E-06 |
| 19204 | Lyl1 | -0.75394 | 3.09E-09 |
| 4922 | Col17a1 | -0.7523 | 4.05E-20 |
| 7538 | Flvcr2 | -0.75062 | 0.000569 |
| 16107 | Gmfg-ps | -0.74893 | 1.37E-06 |
| 16361 | Greb1l | -0.74666 | 3.68E-13 |
| 6289 | Dsg2 | -0.74594 | 3.28E-19 |
| 6530 | Efcab15 | -0.74581 | 0.000941 |
| 4917 | Col12a1 | -0.74477 | 3.05E-37 |
| 22061 | Nkain3 | -0.74357 | 0.000213 |
| 30750 | Trim67 | -0.74346 | 0.000102 |
| 21082 | Mocos | -0.74058 | 0.005791 |
| 26885 | Rps6ka5 | -0.73932 | 2.87E-21 |
| 1737 | Acvrl1 | -0.73869 | 5.03E-22 |
| 2511 | Arhgap6 | -0.73743 | 3.94E-17 |
| 16260 | Gpr146 | -0.7371 | 6.34E-06 |
| 3881 | Ccdc27 | -0.73672 | 0.010486 |
| 3687 | Car13 | -0.73648 | 2.94E-10 |
| 29863 | Tlr13 | -0.73537 | 8.35E-05 |
| 4055 | Cd180 | -0.73533 | 0.000265 |
| 2675 | Ascl5 | -0.73423 | 1.31E-06 |
| 26327 | Rigi | -0.73377 | 8.77E-13 |
| 2130 | Alox12 | -0.73327 | 1.98E-05 |
| 25982 | Rasl10b | -0.73255 | 4.98E-09 |
| 5964 | Dhrs7 | -0.73179 | 2.85E-12 |
| 18298 | Klf14 | -0.73 | 3.20E-13 |
| 16126 | Gnal | -0.72931 | 1.32E-06 |
| 18490 | Krt7 | -0.72866 | 0.000219 |
| 21496 | Mxra8 | -0.72842 | 3.32E-26 |
| 9415 | Gm15753 | -0.72608 | 0.004127 |
| 7690 | Fst | -0.72562 | 8.92E-24 |
| 2310 | Anxa1 | -0.72434 | 1.19E-30 |
| 18741 | Lgals3bp | -0.72368 | 2.31E-11 |
| 3340 | Brinp1 | -0.72147 | 1.06E-15 |
| 15446 | Gm7823 | -0.71919 | 2.74E-08 |
| 15998 | Gm9449 | -0.71888 | 3.13E-05 |
| 7923 | Gbp9 | -0.71887 | 9.73E-13 |
| 5560 | Cysltr1 | -0.71767 | 0.008252 |
| 28119 | Slc7a7 | -0.71688 | 5.92E-09 |
| 3836 | Ccdc149 | -0.71571 | 2.97E-08 |
| 24621 | Pik3cg | -0.71507 | 1.83E-10 |
| 1855 | Adgrl2 | -0.71506 | 2.94E-33 |
| 21801 | Ncf1 | -0.71373 | 3.10E-08 |
| 19383 | Map3k5 | -0.7132 | 2.89E-11 |
| 25635 | Ptgfr | -0.71168 | 2.62E-22 |
| 23993 | P2rx7 | -0.7114 | 1.48E-07 |
| 30793 | Trnp1 | -0.71128 | 0.008152 |
| 2075 | Alcam | -0.7112 | 3.76E-29 |
| 22154 | Nod2 | -0.71117 | 1.76E-06 |
| 29173 | Svep1 | -0.71054 | 2.00E-25 |
| 30258 | Tnk1 | -0.71041 | 2.59E-09 |
| 30226 | Tnfrsf13b | -0.70997 | 0.001642 |
| 27543 | Sfrp2 | -0.70747 | 2.10E-24 |
| 7372 | Fcgr3 | -0.70708 | 2.38E-09 |
| 19025 | Lrp11 | -0.7066 | 0.000122 |
| 24166 | Pbx3 | -0.70637 | 2.83E-19 |
| 27983 | Slc35e4 | -0.70609 | 9.78E-11 |
| 2569 | Arl4c | -0.70607 | 9.46E-16 |
| 3174 | Bdh2 | -0.70606 | 3.15E-06 |
| 2342 | Ap1s3 | -0.70553 | 2.04E-06 |
| 17709 | Il6ra | -0.70491 | 1.20E-06 |
| 4789 | Clstn2 | -0.70416 | 5.46E-21 |
| 18799 | Lin7a | -0.70285 | 6.51E-05 |
| 16713 | Hacd4 | -0.70233 | 3.04E-13 |
| 23919 | Osbpl3 | -0.70074 | 9.08E-17 |
| 3773 | Cbln1 | -0.69979 | 4.57E-08 |
| 19174 | Lvrn | -0.69965 | 5.81E-09 |
| 24365 | Pdp1 | -0.69951 | 1.09E-19 |
| 24543 | Phlda1 | -0.69937 | 4.85E-10 |
| 30880 | Tspan17 | -0.69891 | 2.49E-06 |
| 28546 | Socs6 | -0.6982 | 9.25E-20 |
| 25679 | Ptpn6 | -0.69788 | 1.04E-09 |
| 32468 | Zdhhc23 | -0.69776 | 4.93E-05 |
| 4485 | Cgnl1 | -0.69742 | 6.03E-27 |
| 4099 | Cd302 | -0.69648 | 3.43E-12 |
| 25922 | Ramp1 | -0.69624 | 1.35E-06 |
| 4825 | Cmya5 | -0.69599 | 8.61E-14 |
| 26451 | Rnf182 | -0.69579 | 2.50E-13 |
| 30825 | Trpm5 | -0.69559 | 1.68E-15 |
| 6293 | Dsp | -0.69468 | 3.86E-29 |
| 28163 | Slfn5 | -0.69427 | 1.45E-09 |
| 17915 | Itga8 | -0.69385 | 1.61E-21 |
| 3529 | C5ar1 | -0.69378 | 0.000276 |
| 24491 | Pgr | -0.6935 | 0.011023 |
| 4097 | Cd300lf | -0.69296 | 0.003412 |
| 25638 | Ptgis | -0.69183 | 6.41E-15 |
| 29754 | Themis2 | -0.69011 | 0.000154 |
| 7366 | Fcer1g | -0.68899 | 1.18E-12 |
| 18488 | Krt6a | -0.68888 | 5.45E-10 |
| 1459 | A930011G23Rik | -0.68887 | 4.54E-12 |
| 4400 | Cers4 | -0.68719 | 1.27E-20 |
| 2179 | Amph | -0.68534 | 1.05E-06 |
| 25294 | Prickle2 | -0.68533 | 1.09E-22 |
| 6280 | Dsc3 | -0.68438 | 6.81E-21 |
| 5296 | Ctla2a | -0.68433 | 2.48E-06 |
| 4884 | Cntn5 | -0.68353 | 0.002583 |
| 5383 | Cxcl16 | -0.68347 | 3.64E-06 |
| 27046 | S100a14 | -0.68221 | 7.37E-08 |
| 16466 | Gstt2 | -0.68219 | 9.08E-05 |
| 32204 | Wipf3 | -0.68039 | 2.27E-05 |
| 29047 | Ston2 | -0.67945 | 4.52E-16 |
| 18464 | Krt17 | -0.67905 | 7.99E-24 |
| 26054 | Rbm47 | -0.67884 | 0.000759 |
| 2220 | Ank2 | -0.67812 | 5.03E-24 |
| 27384 | Sema4f | -0.67799 | 2.69E-09 |
| 32888 | Zfp982 | -0.6775 | 0.001005 |
| 27677 | Siglec1 | -0.67686 | 0.000567 |
| 2368 | Apbb1ip | -0.67667 | 1.75E-14 |
| 32382 | Zbtb20 | -0.67653 | 1.38E-23 |
| 5715 | Dclk3 | -0.67505 | 1.14E-06 |
| 27715 | Six2 | -0.67421 | 1.64E-17 |
| 6780 | Entrep2 | -0.67419 | 6.90E-17 |
| 28958 | Stab1 | -0.67368 | 8.69E-18 |
| 30990 | Tub | -0.67228 | 1.97E-09 |
| 5557 | Cyria | -0.67057 | 2.77E-11 |
| 4288 | Cdyl2 | -0.67024 | 4.64E-06 |
| 2359 | Ap5b1 | -0.66969 | 0.002066 |
| 18252 | Kif26b | -0.66931 | 1.48E-24 |
| 4089 | Cd300c2 | -0.66917 | 0.011306 |
| 30228 | Tnfrsf14 | -0.66846 | 0.000113 |
| 4713 | Cldn7 | -0.66838 | 0.010733 |
| 6321 | Dusp1 | -0.66804 | 1.41E-25 |
| 4271 | Cdo1 | -0.66539 | 3.59E-21 |
| 28138 | Slc9b2 | -0.66506 | 0.000362 |
| 28951 | St8sia1 | -0.66469 | 3.56E-05 |
| 17870 | Irgm1 | -0.66445 | 5.90E-09 |
| 21309 | Ms4a6d | -0.66419 | 1.78E-05 |
| 21705 | Naaladl2 | -0.6632 | 9.72E-09 |
| 3276 | Bnc2 | -0.66273 | 3.76E-19 |
| 27939 | Slc2a13 | -0.66113 | 2.14E-09 |
| 17663 | Il1b | -0.66078 | 1.30E-07 |
| 18588 | Lacc1 | -0.66062 | 4.73E-10 |
| 15782 | Gm8812 | -0.66035 | 7.03E-05 |
| 21974 | Nexmif | -0.65862 | 3.38E-09 |
| 21182 | Mrgprf | -0.65784 | 8.23E-13 |
| 5208 | Csf1r | -0.65731 | 5.83E-19 |
| 28824 | Sqor | -0.65731 | 3.35E-06 |
| 22079 | Nkx2-3 | -0.65589 | 9.12E-06 |
| 29711 | Tgfbr2 | -0.65543 | 1.41E-21 |
| 19716 | Mfsd6 | -0.65508 | 3.00E-06 |
| 1789 | Adamts18 | -0.65465 | 4.24E-08 |
| 8022 | Ghr | -0.65339 | 3.02E-20 |
| 24180 | Pcdh11x | -0.65278 | 0.002098 |
| 19027 | Lrp1b | -0.65192 | 0.00179 |
| 17257 | Ifi47 | -0.65186 | 0.009873 |
| 22036 | Nid2 | -0.64844 | 1.55E-24 |
| 29704 | Tgfa | -0.64811 | 9.48E-05 |
| 24760 | Plau | -0.6481 | 1.41E-12 |
| 2400 | Apobr | -0.64808 | 0.000189 |
| 17128 | Hspa1b | -0.64784 | 3.72E-14 |
| 30265 | Tnn | -0.64731 | 1.05E-11 |
| 24497 | Phactr2 | -0.64709 | 4.49E-22 |
| 19403 | Mapk10 | -0.64636 | 0.005565 |
| 30877 | Tspan13 | -0.64508 | 5.46E-14 |
| 29691 | Tfec | -0.64481 | 0.007881 |
| 2662 | Asb4 | -0.64314 | 1.91E-12 |
| 26553 | Rph3al | -0.64218 | 0.001666 |
| 24419 | Perp | -0.64028 | 8.31E-17 |
| 2849 | Atp8b1 | -0.63986 | 2.71E-07 |
| 18753 | Lgr5 | -0.63937 | 1.05E-24 |
| 22645 | Ophn1 | -0.63937 | 2.68E-15 |
| 22016 | Ngfr | -0.63933 | 1.15E-18 |
| 30217 | Tnfaip6 | -0.63896 | 8.45E-15 |
| 7593 | Foxc2 | -0.63845 | 0.005245 |
| 4547 | Chmp4c | -0.63832 | 3.81E-06 |
| 29866 | Tlr4 | -0.63792 | 0.000122 |
| 7547 | Fmo1 | -0.63759 | 7.53E-17 |
| 7370 | Fcgr1 | -0.63758 | 0.000178 |
| 24059 | Palm3 | -0.63426 | 1.90E-08 |
| 543 | 2810032G03Rik | -0.63424 | 8.38E-06 |
| 24108 | Parp14 | -0.63341 | 1.13E-06 |
| 6005 | Diras2 | -0.63233 | 5.87E-05 |
| 7921 | Gbp7 | -0.63068 | 1.97E-06 |
| 31364 | Usp43 | -0.63046 | 0.000403 |
| 16351 | Gramd3 | -0.62891 | 1.30E-13 |
| 6926 | Esrp1 | -0.62886 | 8.84E-13 |
| 7145 | Fam184b | -0.62854 | 0.003978 |
| 24791 | Plek | -0.6281 | 4.57E-09 |
| 22587 | Olfml2a | -0.62785 | 5.27E-19 |
| 29708 | Tgfb3 | -0.62708 | 9.62E-25 |
| 29252 | Sytl1 | -0.6267 | 5.19E-05 |
| 2320 | Anxa8 | -0.62658 | 0.004074 |
| 18188 | Kdf1 | -0.6261 | 0.000949 |
| 18857 | Lmod1 | -0.626 | 0.000286 |
| 24940 | Pof1b | -0.62578 | 0.007604 |
| 17668 | Il1rap | -0.62523 | 8.71E-12 |
| 5932 | Dgat2 | -0.62466 | 7.98E-08 |
| 7886 | Gas6 | -0.62219 | 7.85E-20 |
| 19180 | Ly6d | -0.62182 | 0.000835 |
| 5207 | Csf1 | -0.62135 | 2.91E-20 |
| 19148 | Lst1 | -0.62132 | 0.007519 |
| 26449 | Rnf180 | -0.62101 | 9.38E-08 |
| 18486 | Krt42 | -0.62097 | 0.006352 |
| 18751 | Lgmn | -0.62084 | 3.07E-21 |
| 18979 | Lpar1 | -0.62059 | 2.76E-22 |
| 4930 | Col24a1 | -0.61965 | 3.32E-12 |
| 29247 | Syt5 | -0.61931 | 0.005022 |
| 18970 | Lonrf2 | -0.61845 | 2.81E-13 |
| 6234 | Dpp4 | -0.61773 | 0.001224 |
| 27903 | Slc25a45 | -0.61716 | 1.13E-06 |
| 16221 | Gpc3 | -0.61696 | 2.20E-24 |
| 21843 | Ndnf | -0.61666 | 2.79E-14 |
| 24090 | Paqr7 | -0.61598 | 1.39E-05 |
| 32408 | Zbtb7b | -0.61565 | 6.05E-07 |
| 6779 | Entrep1 | -0.61469 | 0.00189 |
| 7408 | Fermt1 | -0.61446 | 1.46E-06 |
| 4175 | Cdc42ep3 | -0.61424 | 1.54E-12 |
| 6524 | Eepd1 | -0.61311 | 2.27E-09 |
| 17127 | Hspa1a | -0.61286 | 1.63E-05 |
| 5247 | Csrp2 | -0.6125 | 5.02E-18 |
| 22143 | Nnat | -0.61184 | 3.02E-14 |
| 26991 | Rtn1 | -0.61153 | 0.000679 |
| 18462 | Krt15 | -0.61138 | 1.67E-14 |
| 28542 | Socs2 | -0.61049 | 2.51E-12 |
| 24786 | Pld4 | -0.61013 | 6.20E-09 |
| 7188 | Fam241a | -0.60986 | 9.49E-05 |
| 3473 | C1qa | -0.60961 | 8.34E-12 |
| 29188 | Sybu | -0.60769 | 0.005826 |
| 28543 | Socs3 | -0.6076 | 4.73E-16 |
| 23965 | Otulinl | -0.60578 | 5.58E-11 |
| 18495 | Krt75 | -0.60511 | 0.006131 |
| 29860 | Tlr1 | -0.60365 | 0.003243 |
| 25959 | Rarres2 | -0.60362 | 4.88E-05 |
| 6761 | Enpp2 | -0.60361 | 5.05E-21 |
| 22219 | Nphp3 | -0.60334 | 1.24E-15 |
| 7075 | Fam102b | -0.60329 | 7.12E-19 |
| 16299 | Gpr37 | -0.60219 | 0.001283 |
| 30107 | Tmem37 | -0.60218 | 0.004411 |
| 29156 | Susd1 | -0.60151 | 2.76E-05 |
| 19397 | Map7 | -0.60128 | 1.15E-05 |
| 18169 | Kctd14 | -0.60091 | 3.35E-05 |
| 6763 | Enpp4 | -0.60056 | 2.56E-08 |
| 2339 | Ap1m2 | -0.6004 | 1.44E-05 |
| 2908 | Avpr1a | -0.5992 | 0.00045 |
| 7749 | Fzd1 | -0.59635 | 2.03E-16 |
| 5186 | Crybg1 | -0.59622 | 1.02E-05 |
| 32931 | Zim1 | -0.59313 | 1.86E-21 |
| 4197 | Cdh11 | -0.59283 | 1.97E-23 |
| 4211 | Cdh3 | -0.59172 | 3.60E-13 |
| 6261 | Dram1 | -0.59141 | 2.33E-05 |
| 25670 | Ptpn18 | -0.5907 | 0.0041 |
| 7716 | Fuca2 | -0.59067 | 1.48E-12 |
| 17142 | Hspb8 | -0.58854 | 6.78E-09 |
| 24724 | Pla2g4a | -0.58776 | 2.33E-11 |
| 6104 | Dnah7b | -0.58696 | 0.00165 |
| 27132 | Satb2 | -0.58566 | 1.03E-20 |
| 9087 | Gm14410 | -0.58523 | 8.85E-12 |
| 6479 | Ecm2 | -0.58503 | 4.97E-05 |
| 22023 | Nhlrc1 | -0.58482 | 0.003315 |
| 3176 | Bdkrb2 | -0.58287 | 0.000823 |
| 5106 | Crabp1 | -0.58187 | 1.78E-13 |
| 21149 | Mpzl2 | -0.58048 | 0.001951 |
| 4956 | Col9a2 | -0.57838 | 1.36E-11 |
| 5999 | Dipk1a | -0.57739 | 1.51E-10 |
| 26355 | Ripk4 | -0.57695 | 1.46E-05 |
| 2333 | Aox4 | -0.57577 | 0.006612 |
| 19198 | Ly86 | -0.57567 | 0.0035 |
| 6188 | Doc2b | -0.57538 | 3.72E-07 |
| 7320 | Fbxo32 | -0.57372 | 5.61E-08 |
| 4140 | Cd83 | -0.57319 | 1.54E-10 |
| 5188 | Crybg3 | -0.57171 | 4.84E-16 |
| 18633 | Larp6 | -0.5717 | 1.88E-06 |
| 24709 | Pkp3 | -0.57128 | 1.12E-09 |
| 6867 | Erg | -0.57125 | 6.66E-11 |
| 25975 | Rasgrf2 | -0.57082 | 9.92E-05 |
| 18075 | Kcnb1 | -0.57064 | 1.06E-05 |
| 28715 | Sphk1 | -0.57029 | 4.21E-05 |
| 17141 | Hspb7 | -0.56851 | 0.004446 |
| 30223 | Tnfrsf11a | -0.56696 | 6.15E-06 |
| 29819 | Timp3 | -0.56668 | 3.81E-17 |
| 6927 | Esrp2 | -0.5665 | 1.61E-06 |
| 17110 | Hsdl1 | -0.5662 | 2.05E-11 |
| 30149 | Tmem79 | -0.56565 | 0.007934 |
| 21078 | Mob3b | -0.56422 | 1.13E-09 |
| 24776 | Plch2 | -0.5634 | 3.54E-09 |
| 25604 | Ptafr | -0.56321 | 0.006401 |
| 5337 | Ctss | -0.56294 | 6.34E-08 |
| 27353 | Sele | -0.56274 | 0.000782 |
| 21579 | Myo7a | -0.56259 | 1.30E-06 |
| 8118 | Glrx | -0.56233 | 4.64E-07 |
| 29720 | Tgm1 | -0.56194 | 0.008964 |
| 29849 | Tle4 | -0.56185 | 4.63E-18 |
| 4123 | Cd59a | -0.56118 | 5.92E-10 |
| 25890 | Rad51b | -0.56082 | 1.03E-05 |
| 4101 | Cd33 | -0.56003 | 0.000952 |
| 4145 | Cd9 | -0.55934 | 1.84E-10 |
| 716 | 4930447C04Rik | -0.55809 | 5.94E-05 |
| 1588 | Abi3bp | -0.55707 | 8.32E-18 |
| 6764 | Enpp5 | -0.55698 | 4.89E-11 |
| 25854 | Rab6b | -0.55688 | 5.19E-08 |
| 27573 | Sgms2 | -0.55678 | 1.29E-07 |
| 22543 | Ocln | -0.55643 | 0.011201 |
| 4846 | Cnksr3 | -0.55642 | 2.43E-08 |
| 24769 | Plcd1 | -0.55476 | 2.09E-13 |
| 32895 | Zfp991 | -0.55391 | 4.69E-05 |
| 16363 | Grem2 | -0.55375 | 1.10E-13 |
| 27272 | Scrn1 | -0.55374 | 7.75E-08 |
| 29791 | Tifab | -0.55347 | 0.003237 |
| 31020 | Tuft1 | -0.55255 | 1.56E-06 |
| 19253 | Macc1 | -0.55228 | 0.005244 |
| 24829 | Plin2 | -0.55196 | 1.02E-13 |
| 29930 | Tmem100 | -0.55183 | 4.05E-11 |
| 5085 | Cpq | -0.55136 | 1.45E-08 |
| 26078 | Rbpjl | -0.55114 | 0.004161 |
| 2029 | Akap12 | -0.5507 | 6.03E-19 |
| 5606 | D430041D05Rik | -0.55033 | 0.005677 |
| 27083 | Samd10 | -0.54941 | 0.001228 |
| 28562 | Sorcs3 | -0.54739 | 0.006158 |
| 28649 | Spata2l | -0.54739 | 0.000785 |
| 30291 | Tob1 | -0.54698 | 6.55E-12 |
| 24053 | Pak6 | -0.54668 | 0.000825 |
| 6203 | Dok2 | -0.54649 | 4.46E-06 |
| 7425 | Fgd2 | -0.54509 | 2.16E-05 |
| 23915 | Osbpl10 | -0.54446 | 2.92E-06 |
| 4997 | Coq10b | -0.54441 | 1.12E-09 |
| 2726 | Atf3 | -0.54375 | 2.90E-12 |
| 3862 | Ccdc181 | -0.54298 | 0.000415 |
| 18235 | Kif17 | -0.54261 | 0.011658 |
| 4800 | Clybl | -0.54222 | 2.86E-05 |
| 16525 | Gulp1 | -0.54203 | 9.18E-13 |
| 16835 | Helz2 | -0.5416 | 2.82E-10 |
| 5899 | Dennd1c | -0.54146 | 0.000918 |
| 5132 | Creg1 | -0.54132 | 2.40E-11 |
| 25144 | Ppp1r3c | -0.54118 | 2.47E-08 |
| 6642 | Eif4e3 | -0.5409 | 2.86E-06 |
| 3426 | Btg2 | -0.54042 | 1.08E-17 |
| 21922 | Nedd9 | -0.53965 | 1.37E-14 |
| 19417 | Mapk8ip2 | -0.53951 | 0.003906 |
| 4722 | Clec14a | -0.53928 | 5.37E-11 |
| 4192 | Cdcp1 | -0.53916 | 4.24E-07 |
| 4737 | Clec4a1 | -0.53862 | 0.002003 |
| 2622 | Arrdc3 | -0.53797 | 7.77E-15 |
| 4845 | Cnksr2 | -0.53707 | 6.06E-08 |
| 30081 | Tmem254 | -0.53698 | 1.82E-09 |
| 28933 | St14 | -0.53668 | 3.68E-08 |
| 2152 | Ambn | -0.53565 | 6.72E-11 |
| 7082 | Fam110d | -0.53528 | 0.000239 |
| 32896 | Zfp992 | -0.5346 | 1.41E-05 |
| 28988 | Stat5a | -0.53408 | 1.22E-07 |
| 22572 | Ogfrl1 | -0.53397 | 1.53E-09 |
| 7123 | Fam167b | -0.53366 | 0.001983 |
| 21988 | Nfe2l2 | -0.53341 | 3.34E-14 |
| 2526 | Arhgef19 | -0.53293 | 6.17E-11 |
| 6078 | Dmtn | -0.53238 | 2.74E-05 |
| 21491 | Mxd1 | -0.53047 | 2.54E-13 |
| 6580 | Ehhadh | -0.53046 | 0.00237 |
| 16154 | Gngt2 | -0.52999 | 0.001621 |
| 3372 | Btbd11 | -0.52985 | 8.45E-08 |
| 3029 | Bambi | -0.52853 | 6.10E-10 |
| 29060 | Strbp | -0.52838 | 1.18E-12 |
| 15852 | Gm8995 | -0.52783 | 0.01084 |
| 16284 | Gpr183 | -0.52757 | 0.000167 |
| 2423 | Apold1 | -0.52738 | 3.57E-11 |
| 8651 | Gm12543 | -0.52701 | 4.69E-06 |
| 21420 | Mtus1 | -0.5252 | 2.28E-13 |
| 5141 | Crim1 | -0.52421 | 1.27E-11 |
| 2135 | Alox5ap | -0.52395 | 0.00405 |
| 17130 | Hspa2 | -0.52393 | 0.000231 |
| 28758 | Spock1 | -0.52335 | 6.55E-10 |
| 14777 | Gm5749 | -0.52294 | 0.001485 |
| 28160 | Slfn2 | -0.52294 | 0.000544 |
| 2601 | Armh4 | -0.52247 | 1.45E-07 |
| 23996 | P2ry10b | -0.5224 | 0.003813 |
| 27637 | She | -0.52227 | 3.03E-09 |
| 16377 | Grik2 | -0.52209 | 0.007138 |
| 6808 | Epha4 | -0.522 | 9.83E-13 |
| 7013 | Eya4 | -0.52191 | 8.75E-09 |
| 27726 | Skap2 | -0.52114 | 0.000591 |
| 28718 | Spi1 | -0.52072 | 6.44E-06 |
| 7414 | Fez1 | -0.52042 | 1.91E-11 |
| 21782 | Nbl1 | -0.51925 | 1.82E-13 |
| 5506 | Cyp2w1 | -0.51862 | 0.001537 |
| 7651 | Frem1 | -0.51786 | 8.71E-15 |
| 1695 | Acss1 | -0.51722 | 0.000248 |
| 28147 | Slco2b1 | -0.51628 | 1.47E-05 |
| 7449 | Fgf7 | -0.51617 | 2.21E-06 |
| 8969 | Gm13889 | -0.51561 | 3.25E-05 |
| 28982 | Stard8 | -0.51532 | 5.27E-09 |
| 31435 | Vcam1 | -0.51474 | 2.74E-09 |
| 4487 | Cgrrf1 | -0.51434 | 2.48E-07 |
| 14741 | Gm5675 | -0.51419 | 0.004857 |
| 6525 | Efcab1 | -0.51393 | 0.003539 |
| 30832 | Trpv2 | -0.51361 | 0.000816 |
| 28519 | Snx2 | -0.51234 | 3.06E-13 |
| 5952 | Dhdh | -0.51224 | 0.000364 |
| 25462 | Prrx1 | -0.51154 | 3.20E-16 |
| 18022 | Kank1 | -0.5112 | 3.67E-08 |
| 32222 | Wnt3a | -0.51119 | 0.006358 |
| 7461 | Fggy | -0.50974 | 0.001211 |
| 5568 | Cyth4 | -0.50899 | 1.06E-08 |
| 22273 | Nr3c1 | -0.50811 | 1.31E-11 |
| 20027 | Mir22hg | -0.50751 | 6.49E-05 |
| 25842 | Rab40b | -0.50694 | 9.65E-05 |
| 16399 | Grk5 | -0.5068 | 5.09E-11 |
| 28565 | Sort1 | -0.50579 | 1.74E-12 |
| 67 | 1700001J11Rik | -0.50544 | 0.000334 |
| 6489 | Edaradd | -0.50519 | 1.95E-05 |
| 21846 | Ndrg1 | -0.5039 | 3.01E-11 |
| 2694 | Asph | -0.50196 | 1.54E-13 |
| 21808 | Nckap1l | -0.50135 | 1.03E-08 |
| 19454 | Masp1 | -0.50034 | 1.18E-09 |

**Table6. Upregulated DEGs of buccal part of tooth germs at bell stage than of lingual part**

|  | SYMBOL | log2FoldChange | pvalue |
| --- | --- | --- | --- |
| 27690 | Sim1 | 7.077194 | 6.79E-27 |
| 26253 | Rhcg | 5.450653 | 5.94E-21 |
| 21533 | Myh7 | 4.668443 | 2.48E-90 |
| 18773 | Lhx9 | 4.599285 | 7.58E-18 |
| 17889 | Isl1 | 4.441585 | 2.82E-98 |
| 32925 | Zic1 | 4.281745 | 2.33E-46 |
| 21532 | Myh6 | 4.089434 | 5.56E-49 |
| 4736 | Clec3b | 3.990821 | 2.75E-61 |
| 21530 | Myh3 | 3.971773 | 0 |
| 21523 | Myh1 | 3.927662 | 2.17E-60 |
| 18213 | Kera | 3.906359 | 2.75E-192 |
| 2236 | Ankrd1 | 3.83806 | 8.04E-20 |
| 18858 | Lmod2 | 3.772522 | 3.69E-08 |
| 17027 | Hoxc4 | 3.718248 | 0.000129 |
| 18803 | Lincmd1 | 3.665988 | 1.32E-82 |
| 6735 | Emx2 | 3.665873 | 1.65E-35 |
| 19538 | Mcpt4 | 3.637742 | 3.64E-21 |
| 32928 | Zic4 | 3.625775 | 2.08E-05 |
| 21590 | Myom2 | 3.61223 | 2.81E-107 |
| 1716 | Actn2 | 3.574426 | 6.01E-215 |
| 5248 | Csrp3 | 3.573414 | 2.13E-34 |
| 21520 | Myf5 | 3.563449 | 4.10E-39 |
| 24152 | Pax8 | 3.539641 | 5.31E-06 |
| 32242 | Wt1os | 3.53655 | 4.06E-05 |
| 2785 | Atp1b4 | 3.522336 | 1.03E-11 |
| 30369 | Tpsb2 | 3.49613 | 4.80E-171 |
| 21531 | Myh4 | 3.460183 | 1.31E-19 |
| 21553 | Mylk4 | 3.452431 | 2.73E-122 |
| 5079 | Cpne6 | 3.441391 | 6.67E-05 |
| 30983 | Ttn | 3.431282 | 0 |
| 23986 | Oxtr | 3.402439 | 1.45E-63 |
| 32258 | Xirp1 | 3.395665 | 1.06E-13 |
| 3736 | Casq2 | 3.317275 | 1.45E-95 |
| 25310 | Prkag3 | 3.312613 | 3.14E-34 |
| 30266 | Tnnc1 | 3.30492 | 7.18E-189 |
| 17847 | Iqsec3 | 3.294047 | 1.43E-25 |
| 19535 | Mcpt-ps1 | 3.284654 | 1.53E-07 |
| 1832 | Adgra1 | 3.273429 | 3.37E-05 |
| 3306 | Bpifb1 | 3.273386 | 4.01E-05 |
| 5035 | Cox6a2 | 3.242909 | 4.70E-14 |
| 3467 | C130080G10Rik | 3.241907 | 3.66E-05 |
| 4206 | Cdh20 | 3.236025 | 1.31E-79 |
| 30370 | Tpsg1 | 3.229199 | 6.85E-06 |
| 4586 | Chrng | 3.215415 | 1.05E-102 |
| 6736 | Emx2os | 3.21256 | 7.62E-11 |
| 22630 | Onecut1 | 3.211446 | 4.81E-05 |
| 3584 | Cacng1 | 3.185252 | 8.91E-15 |
| 4567 | Chrm3 | 3.169292 | 1.57E-19 |
| 30739 | Trim55 | 3.145157 | 4.16E-60 |
| 2141 | Alpk3 | 3.136324 | 2.24E-64 |
| 2616 | Arpp21 | 3.127352 | 2.15E-98 |
| 16378 | Grik3 | 3.110626 | 8.46E-125 |
| 21506 | Mybph | 3.09577 | 1.48E-94 |
| 17789 | Insyn2b | 3.085422 | 1.40E-79 |
| 1703 | Actc1 | 3.074801 | 0 |
| 9550 | Gm16551 | 3.070367 | 2.68E-05 |
| 7615 | Foxl2os | 3.042467 | 4.80E-24 |
| 5140 | Crhr2 | 3.039067 | 4.07E-18 |
| 29228 | Synpo2l | 3.037069 | 2.51E-150 |
| 3269 | Bmpr1b | 3.032683 | 2.32E-57 |
| 21907 | Neb | 3.030552 | 3.23E-268 |
| 21554 | Mylpf | 3.02471 | 5.51E-214 |
| 29472 | Tbx3os2 | 3.011011 | 4.05E-05 |
| 21529 | Myh2 | 3.000409 | 1.23E-35 |
| 8071 | Gjd4 | 2.99618 | 3.30E-09 |
| 25363 | Prlr | 2.996116 | 2.61E-27 |
| 4842 | Cnih3 | 2.989041 | 3.24E-16 |
| 5961 | Dhrs2 | 2.983979 | 7.34E-08 |
| 16563 | H1f9 | 2.98219 | 0.001185 |
| 3758 | Cav3 | 2.97901 | 6.63E-45 |
| 18349 | Klhl40 | 2.977238 | 4.72E-20 |
| 29492 | Tceal7 | 2.971581 | 1.21E-48 |
| 21555 | Mymk | 2.968232 | 3.09E-81 |
| 29464 | Tbx18 | 2.966091 | 8.75E-162 |
| 22081 | Nkx2-5 | 2.962352 | 4.78E-12 |
| 30268 | Tnni1 | 2.962324 | 1.70E-140 |
| 18859 | Lmod3 | 2.9514 | 1.35E-27 |
| 4065 | Cd200r3 | 2.946197 | 0.000326 |
| 587 | 3300002P13Rik | 2.946185 | 0.000319 |
| 17028 | Hoxc5 | 2.931058 | 3.18E-05 |
| 30368 | Tpsab1 | 2.928546 | 1.03E-67 |
| 17696 | Il33 | 2.927506 | 3.78E-38 |
| 27253 | Scn4a | 2.921944 | 5.24E-25 |
| 21593 | Myot | 2.921126 | 6.03E-25 |
| 16909 | Hjv | 2.918922 | 2.96E-41 |
| 24179 | Pcdh10 | 2.917465 | 0 |
| 19537 | Mcpt2 | 2.911558 | 5.98E-06 |
| 3374 | Btbd17 | 2.909234 | 4.08E-14 |
| 1208 | 6430584L05Rik | 2.88907 | 0.001683 |
| 30273 | Tnnt2 | 2.879581 | 3.15E-192 |
| 1827 | Adcyap1r1 | 2.865836 | 2.58E-68 |
| 193 | 1700027H10Rik | 2.857639 | 0.000159 |
| 6734 | Emx1 | 2.85572 | 0.006916 |
| 1156 | 5430431A17Rik | 2.848026 | 5.90E-56 |
| 28176 | Slitrk5 | 2.832249 | 4.68E-69 |
| 4584 | Chrnd | 2.816986 | 2.94E-74 |
| 28312 | Smyd1 | 2.808477 | 9.97E-79 |
| 14000 | Gm3985 | 2.795738 | 1.78E-14 |
| 21521 | Myf6 | 2.790888 | 8.04E-05 |
| 26223 | Rgs13 | 2.787852 | 0.00075 |
| 27862 | Slc24a4 | 2.786721 | 1.99E-13 |
| 18103 | Kcnip1 | 2.776174 | 1.44E-34 |
| 4200 | Cdh15 | 2.773144 | 2.19E-128 |
| 565 | 2900079G21Rik | 2.727347 | 0.000115 |
| 24151 | Pax7 | 2.725113 | 4.04E-78 |
| 3301 | Bpifa1 | 2.718211 | 0.011407 |
| 21586 | Myod1 | 2.711433 | 2.59E-145 |
| 21597 | Mypn | 2.710889 | 7.83E-32 |
| 17648 | Il17b | 2.707868 | 5.45E-11 |
| 32241 | Wt1 | 2.698054 | 0.000172 |
| 7458 | Fgfr4 | 2.694482 | 3.87E-213 |
| 21056 | Mmp3 | 2.690168 | 4.91E-09 |
| 17066 | Hrh1 | 2.682794 | 0.005336 |
| 19539 | Mcpt8 | 2.681686 | 0.000469 |
| 27252 | Scn3b | 2.67574 | 8.55E-125 |
| 21534 | Myh7b | 2.670222 | 2.35E-47 |
| 1699 | Acta1 | 2.661294 | 8.18E-203 |
| 2640 | Arx | 2.657141 | 4.63E-07 |
| 2297 | Ano5 | 2.653435 | 1.24E-17 |
| 32607 | Zfp385b | 2.631983 | 1.74E-74 |
| 4570 | Chrna1 | 2.627306 | 5.75E-88 |
| 25954 | Rapsn | 2.626989 | 1.50E-53 |
| 3575 | Cacna1s | 2.624457 | 1.45E-28 |
| 18717 | Lefty2 | 2.622695 | 0.007293 |
| 30272 | Tnnt1 | 2.609365 | 4.20E-208 |
| 21535 | Myh8 | 2.608813 | 1.84E-141 |
| 27561 | Sgca | 2.605772 | 1.12E-25 |
| 21544 | Myl4 | 2.599015 | 1.04E-159 |
| 4147 | Cd96 | 2.590521 | 3.14E-05 |
| 30755 | Trim72 | 2.583 | 7.47E-28 |
| 4801 | Cma1 | 2.579116 | 2.80E-149 |
| 18350 | Klhl41 | 2.569214 | 1.24E-64 |
| 7819 | Gal3st2 | 2.567908 | 0.002658 |
| 21538 | Myl1 | 2.565057 | 3.42E-39 |
| 6168 | Dner | 2.557027 | 9.63E-44 |
| 7496 | Fitm1 | 2.555505 | 4.57E-19 |
| 3267 | Bmper | 2.552443 | 1.04E-144 |
| 21591 | Myom3 | 2.545445 | 4.27E-45 |
| 7988 | Gfi1 | 2.542377 | 2.42E-05 |
| 26218 | Rgr | 2.535249 | 0.000222 |
| 24678 | Pitx3 | 2.528435 | 1.49E-42 |
| 25479 | Prss34 | 2.525348 | 5.59E-14 |
| 21595 | Myoz2 | 2.51659 | 1.81E-21 |
| 18077 | Kcnc1 | 2.506519 | 1.11E-12 |
| 18341 | Klhl31 | 2.506182 | 1.66E-17 |
| 29961 | Tmem132b | 2.50333 | 1.78E-67 |
| 9269 | Gm15128 | 2.502698 | 0.008672 |
| 27039 | Ryr1 | 2.500224 | 7.89E-176 |
| 21556 | Mymx | 2.499075 | 3.62E-32 |
| 28860 | Srpk3 | 2.49581 | 6.11E-33 |
| 7614 | Foxl2 | 2.478376 | 8.36E-09 |
| 5938 | Dgkb | 2.472529 | 1.18E-52 |
| 29463 | Tbx15 | 2.47158 | 2.53E-210 |
| 5634 | D930007P13Rik | 2.464461 | 5.34E-05 |
| 2942 | B130024G19Rik | 2.446996 | 1.64E-70 |
| 21475 | Mup5 | 2.441777 | 0.01168 |
| 5426 | Cyp11a1 | 2.44078 | 1.03E-14 |
| 25443 | Prr32 | 2.429888 | 6.93E-11 |
| 17138 | Hspb2 | 2.424113 | 1.32E-14 |
| 21589 | Myom1 | 2.411073 | 1.07E-94 |
| 6208 | Dok7 | 2.410034 | 6.11E-59 |
| 13350 | Gm29683 | 2.408655 | 0.0005 |
| 4721 | Clec12b | 2.388319 | 1.02E-10 |
| 1884 | Adprhl1 | 2.386078 | 0.001413 |
| 31272 | Unc93a | 2.382037 | 0.00121 |
| 19028 | Lrp2 | 2.375724 | 2.12E-07 |
| 5923 | Des | 2.374227 | 4.92E-161 |
| 30345 | Tph1 | 2.366701 | 7.83E-13 |
| 3190 | Best3 | 2.366595 | 3.79E-12 |
| 1858 | Adgrv1 | 2.358122 | 4.30E-31 |
| 4931 | Col25a1 | 2.355911 | 7.05E-197 |
| 18767 | Lhx2 | 2.340727 | 1.36E-08 |
| 3209 | Bhlhe22 | 2.336581 | 6.21E-35 |
| 7466 | Fhad1 | 2.333044 | 3.79E-10 |
| 30331 | Tox2 | 2.330207 | 2.09E-84 |
| 27029 | Rxfp1 | 2.327907 | 6.62E-43 |
| 21481 | Musk | 2.324947 | 3.54E-17 |
| 28300 | Smpx | 2.324567 | 4.54E-22 |
| 22270 | Nr2f1 | 2.305674 | 2.07E-131 |
| 2443 | Aqp5 | 2.305627 | 0.004684 |
| 22551 | Odam | 2.303728 | 1.77E-06 |
| 22536 | Obscn | 2.298077 | 1.28E-30 |
| 17266 | Ifitm10 | 2.280633 | 1.29E-18 |
| 30684 | Trhr | 2.266042 | 1.10E-16 |
| 21317 | Msc | 2.260137 | 5.01E-89 |
| 22223 | Nphs2 | 2.256756 | 0.005925 |
| 25971 | Rasef | 2.256559 | 1.12E-11 |
| 2246 | Ankrd2 | 2.249643 | 2.98E-13 |
| 27645 | Shisa6 | 2.24616 | 0.011201 |
| 3589 | Cacng6 | 2.239414 | 4.38E-06 |
| 19160 | Ltf | 2.235793 | 3.12E-06 |
| 22293 | Nrg1 | 2.232037 | 6.06E-71 |
| 6810 | Epha6 | 2.231764 | 0.000142 |
| 30025 | Tmem196 | 2.226991 | 1.60E-07 |
| 6694 | Elovl3 | 2.216783 | 4.13E-05 |
| 29503 | Tcf21 | 2.213618 | 1.05E-06 |
| 5051 | Cpa3 | 2.213218 | 5.28E-160 |
| 4784 | Clrn1 | 2.206453 | 1.81E-42 |
| 27565 | Sgcg | 2.182588 | 1.21E-11 |
| 30649 | Trdn | 2.180171 | 0.000188 |
| 17139 | Hspb3 | 2.162144 | 1.52E-05 |
| 27035 | Rxrg | 2.160422 | 3.57E-30 |
| 29468 | Tbx21 | 2.154273 | 0.002478 |
| 18340 | Klhl30 | 2.150026 | 1.95E-13 |
| 2397 | Apobec2 | 2.144329 | 4.56E-20 |
| 4683 | Cldn11 | 2.135726 | 5.29E-68 |
| 16406 | Grm6 | 2.134882 | 0.001449 |
| 4529 | Chil1 | 2.131639 | 7.46E-13 |
| 18155 | Kcns2 | 2.129849 | 1.58E-12 |
| 17676 | Il20ra | 2.124663 | 5.96E-15 |
| 21588 | Myog | 2.124624 | 1.49E-126 |
| 17892 | Islr2 | 2.12033 | 2.37E-16 |
| 1881 | Adora3 | 2.120248 | 1.24E-05 |
| 17925 | Itgb1bp2 | 2.115004 | 2.19E-47 |
| 32794 | Zfp804a | 2.093644 | 1.18E-11 |
| 30738 | Trim54 | 2.082359 | 2.48E-05 |
| 25283 | Prelp | 2.081116 | 8.91E-94 |
| 27394 | Semp2l1 | 2.06802 | 0.004246 |
| 21153 | Mrap2 | 2.061292 | 0.000321 |
| 3484 | C1qtnf3 | 2.060349 | 9.76E-238 |
| 25072 | Ppfia2 | 2.057004 | 4.29E-99 |
| 26239 | Rgs8 | 2.055651 | 5.29E-21 |
| 4580 | Chrnb1 | 2.051154 | 1.05E-86 |
| 30158 | Tmem88b | 2.046146 | 8.73E-18 |
| 21552 | Mylk3 | 2.034393 | 2.73E-29 |
| 16257 | Gpr141b | 2.033578 | 0.00038 |
| 18828 | Lix1 | 2.033548 | 2.86E-49 |
| 45 | 1520401A03Rik | 2.030287 | 0.000264 |
| 30013 | Tmem182 | 2.029171 | 0.000221 |
| 7448 | Fgf6 | 2.026946 | 0.005378 |
| 25043 | Pou3f2 | 2.020282 | 2.03E-09 |
| 24496 | Phactr1 | 2.019234 | 1.30E-94 |
| 4963 | Colq | 2.004294 | 1.40E-10 |
| 16319 | Gpr88 | 2.004018 | 0.000104 |
| 1597 | Abra | 1.982892 | 0.000569 |
| 25054 | Pou6f2 | 1.981976 | 0.010968 |
| 6673 | Elfn2 | 1.978329 | 0.0078 |
| 30269 | Tnni2 | 1.977423 | 2.13E-37 |
| 1700 | Acta2 | 1.972705 | 1.68E-209 |
| 4028 | Ccr8 | 1.972231 | 4.30E-08 |
| 16799 | Hdc | 1.970748 | 9.03E-112 |
| 2787 | Atp2a1 | 1.961978 | 1.32E-62 |
| 3039 | Barx2 | 1.956459 | 2.50E-45 |
| 7745 | Fyb2 | 1.945988 | 0.002065 |
| 28625 | Spag17 | 1.945641 | 2.68E-05 |
| 8055 | Gja3 | 1.942683 | 3.57E-05 |
| 10786 | Gm22027 | 1.942362 | 0.004861 |
| 21190 | Mrln | 1.936387 | 2.24E-09 |
| 18068 | Kcna4 | 1.935722 | 4.68E-32 |
| 18006 | Jph2 | 1.933861 | 2.45E-35 |
| 3625 | Caln1 | 1.927851 | 0.00116 |
| 19125 | Lrtm1 | 1.919805 | 0.00156 |
| 23944 | Otog | 1.919418 | 7.41E-09 |
| 21563 | Myo18b | 1.913756 | 7.54E-34 |
| 16541 | Gzmb | 1.902181 | 5.42E-09 |
| 22242 | Nptx2 | 1.896482 | 1.99E-18 |
| 17070 | Hrk | 1.883868 | 4.30E-06 |
| 16387 | Grin3a | 1.88053 | 2.83E-78 |
| 29253 | Sytl2 | 1.872168 | 1.75E-104 |
| 22461 | Nxph1 | 1.869643 | 1.23E-57 |
| 3684 | Car10 | 1.867728 | 0.002295 |
| 589 | 3425401B19Rik | 1.867149 | 7.42E-08 |
| 3600 | Cadps | 1.867056 | 4.87E-36 |
| 25835 | Rab3b | 1.863568 | 1.22E-18 |
| 28085 | Slc6a11 | 1.857716 | 0.009956 |
| 26170 | Retnlg | 1.857654 | 0.009205 |
| 17330 | Igfbp1 | 1.857568 | 0.008251 |
| 7949 | Gcsam | 1.851716 | 7.66E-06 |
| 29902 | Tmc5 | 1.836818 | 6.30E-05 |
| 1787 | Adamts16 | 1.835535 | 2.99E-11 |
| 686 | 4930429F24Rik | 1.833528 | 7.22E-12 |
| 6842 | Epyc | 1.809382 | 0.010202 |
| 18699 | Ldb3 | 1.808107 | 5.63E-22 |
| 22018 | Ngp | 1.80485 | 2.82E-07 |
| 27599 | Sh3bgr | 1.804724 | 2.46E-10 |
| 30173 | Tmod1 | 1.804073 | 6.93E-10 |
| 1594 | Ablim3 | 1.79756 | 7.92E-70 |
| 28172 | Slitrk1 | 1.789042 | 4.59E-07 |
| 18843 | Lmcd1 | 1.77552 | 2.19E-92 |
| 7554 | Fmod | 1.773345 | 6.06E-118 |
| 16384 | Grin2b | 1.771201 | 3.85E-14 |
| 1526 | Abca8a | 1.762683 | 2.14E-71 |
| 1958 | Agtr1a | 1.758129 | 6.91E-33 |
| 21601 | Myrip | 1.752278 | 1.91E-16 |
| 2140 | Alpk2 | 1.751304 | 1.63E-17 |
| 5430 | Cyp19a1 | 1.749191 | 0.010875 |
| 31262 | Unc45b | 1.740823 | 1.88E-32 |
| 17155 | Htr1a | 1.727553 | 2.30E-11 |
| 15 | 1110002E22Rik | 1.727505 | 3.48E-21 |
| 8057 | Gja5 | 1.727089 | 3.43E-50 |
| 26034 | Rbm24 | 1.726706 | 3.40E-59 |
| 21012 | Mlip | 1.725542 | 0.00863 |
| 32115 | Wdr17 | 1.718746 | 1.17E-14 |
| 29288 | Tacr1 | 1.716093 | 1.91E-10 |
| 25973 | Rasgef1c | 1.711873 | 0.000285 |
| 17159 | Htr2a | 1.710558 | 2.74E-19 |
| 32076 | Vwc2 | 1.70829 | 5.31E-12 |
| 7657 | Frmd3 | 1.702348 | 7.84E-24 |
| 18026 | Kank4os | 1.695955 | 0.001751 |
| 18014 | Jsrp1 | 1.695669 | 9.30E-12 |
| 18716 | Lefty1 | 1.695008 | 0.001283 |
| 28180 | Sln | 1.688404 | 3.50E-08 |
| 26760 | Rprm | 1.685123 | 2.78E-30 |
| 18082 | Kcnd2 | 1.678638 | 0.007062 |
| 6849 | Erbb4 | 1.673815 | 2.73E-09 |
| 26998 | Rtn4rl2 | 1.658308 | 1.32E-08 |
| 17942 | Itk | 1.654002 | 0.002359 |
| 22359 | Ntn1 | 1.65146 | 1.27E-120 |
| 18220 | Khdrbs2 | 1.651446 | 1.24E-20 |
| 28769 | Spp1 | 1.651187 | 9.41E-18 |
| 2631 | Art1 | 1.647561 | 2.34E-06 |
| 3831 | Ccdc141 | 1.647081 | 1.82E-49 |
| 25019 | Popdc2 | 1.643857 | 1.67E-09 |
| 17043 | Hpca | 1.640421 | 1.40E-11 |
| 6206 | Dok5 | 1.637494 | 1.23E-23 |
| 24706 | Pknox2 | 1.625923 | 2.66E-43 |
| 1214 | 6720468P15Rik | 1.625437 | 0.003001 |
| 32055 | Vstm2a | 1.624083 | 3.18E-50 |
| 24803 | Plekhd1 | 1.622691 | 5.75E-08 |
| 18862 | Lmx1a | 1.622179 | 9.50E-19 |
| 2970 | B3gat1 | 1.620276 | 5.16E-18 |
| 29094 | Styk1 | 1.616894 | 0.000105 |
| 1388 | A430078I02Rik | 1.614142 | 0.001695 |
| 4919 | Col14a1 | 1.608843 | 5.78E-135 |
| 1804 | Adamtsl5 | 1.605766 | 1.07E-51 |
| 5301 | Ctnna3 | 1.605681 | 0.011329 |
| 27952 | Slc30a3 | 1.598878 | 5.27E-05 |
| 7802 | Gabrg3 | 1.592929 | 3.59E-15 |
| 19386 | Map3k7cl | 1.586034 | 3.76E-07 |
| 5053 | Cpa5 | 1.582089 | 9.84E-05 |
| 30274 | Tnnt3 | 1.576409 | 7.31E-38 |
| 16369 | Gria2 | 1.569681 | 2.01E-94 |
| 4565 | Chrm1 | 1.568688 | 0.006006 |
| 4639 | Cited1 | 1.566424 | 3.12E-28 |
| 29071 | Stum | 1.566135 | 1.83E-05 |
| 21954 | Nes | 1.55981 | 4.20E-96 |
| 2967 | B3galt5 | 1.55962 | 6.89E-17 |
| 17911 | Itga4 | 1.54893 | 1.84E-92 |
| 19690 | Mfap5 | 1.540635 | 1.47E-11 |
| 3342 | Brinp3 | 1.533723 | 0.000183 |
| 29167 | Sv2c | 1.524447 | 1.88E-75 |
| 8339 | Gm11266 | 1.523378 | 1.56E-16 |
| 28595 | Sox8 | 1.52232 | 1.65E-35 |
| 26992 | Rtn2 | 1.521502 | 7.59E-37 |
| 2469 | Arfgef3 | 1.517487 | 1.68E-45 |
| 25846 | Rab44 | 1.516859 | 4.86E-09 |
| 4626 | Cilp | 1.513907 | 6.48E-10 |
| 32218 | Wnt16 | 1.513486 | 1.44E-44 |
| 29856 | Tll2 | 1.513374 | 3.64E-05 |
| 7442 | Fgf21 | 1.510606 | 0.009355 |
| 2073 | Alb | 1.509512 | 4.35E-12 |
| 18981 | Lpar3 | 1.509298 | 1.50E-06 |
| 454 | 2310002L09Rik | 1.507925 | 0.009747 |
| 25004 | Pom121l2 | 1.506431 | 0.007843 |
| 16207 | Gpat3 | 1.503767 | 0.011147 |
| 21924 | Nefl | 1.497487 | 1.52E-33 |
| 24747 | Platr14 | 1.497369 | 5.16E-07 |
| 2660 | Asb2 | 1.492386 | 1.43E-09 |
| 21925 | Nefm | 1.490631 | 7.18E-31 |
| 29965 | Tmem132e | 1.487976 | 1.57E-35 |
| 21344 | Mstn | 1.482294 | 0.000188 |
| 6686 | Eln | 1.478654 | 1.08E-111 |
| 4217 | Cdh9 | 1.472565 | 0.000792 |
| 31199 | Ucma | 1.471428 | 0.010775 |
| 1437 | A830011K09Rik | 1.465275 | 0.003698 |
| 25175 | Ppp3r2 | 1.463542 | 0.000803 |
| 6033 | Dlgap3 | 1.458307 | 6.22E-11 |
| 18066 | Kcna2 | 1.449789 | 1.22E-21 |
| 27387 | Sema5b | 1.448403 | 9.07E-46 |
| 1888 | Adra1b | 1.447533 | 3.44E-07 |
| 4204 | Cdh19 | 1.446269 | 6.99E-08 |
| 28996 | Steap1 | 1.444345 | 3.11E-07 |
| 7666 | Frmpd2 | 1.443128 | 0.001826 |
| 32358 | Zan | 1.440625 | 5.40E-17 |
| 25042 | Pou3f1 | 1.439632 | 0.000208 |
| 29929 | Tmeff2 | 1.435712 | 1.02E-07 |
| 27545 | Sfrp5 | 1.431856 | 3.25E-14 |
| 26032 | Rbm20 | 1.428401 | 7.56E-32 |
| 4951 | Col6a6 | 1.425704 | 2.11E-76 |
| 30800 | Trp53cor1 | 1.41968 | 0.010853 |
| 5612 | D630023F18Rik | 1.418563 | 0.003363 |
| 21526 | Myh13 | 1.414728 | 9.09E-08 |
| 30759 | Trim9 | 1.411991 | 2.06E-19 |
| 26234 | Rgs4 | 1.411977 | 3.89E-37 |
| 18280 | Kirrel2 | 1.408964 | 0.000225 |
| 25322 | Prkcq | 1.396195 | 1.72E-25 |
| 8068 | Gjc3 | 1.39379 | 1.15E-05 |
| 18650 | Lbx1 | 1.390987 | 0.000509 |
| 5179 | Cryab | 1.388754 | 6.32E-42 |
| 30097 | Tmem273 | 1.388723 | 0.011777 |
| 16275 | Gpr17 | 1.388205 | 5.25E-22 |
| 5579 | D030045P18Rik | 1.387143 | 0.003808 |
| 17627 | Ikzf2 | 1.37849 | 4.24E-35 |
| 23942 | Otoa | 1.37344 | 1.18E-06 |
| 17641 | Il13 | 1.371337 | 0.000727 |
| 27848 | Slc22a3 | 1.367435 | 1.48E-05 |
| 6380 | Dynlt5 | 1.364622 | 0.001621 |
| 24092 | Paqr9 | 1.360903 | 0.008078 |
| 17756 | Inka2 | 1.356618 | 7.46E-22 |
| 17985 | Jakmip3 | 1.354383 | 2.99E-05 |
| 18683 | Lcn2 | 1.349579 | 0.003633 |
| 27612 | Sh3gl3 | 1.346376 | 4.95E-30 |
| 1445 | A830082K12Rik | 1.337587 | 3.19E-26 |
| 27877 | Slc25a21 | 1.337533 | 5.32E-16 |
| 3479 | C1ql3 | 1.328234 | 7.10E-06 |
| 28609 | Sp9 | 1.327892 | 8.12E-26 |
| 16354 | Grap2 | 1.320997 | 9.27E-05 |
| 7569 | Fndc3c1 | 1.308026 | 9.80E-61 |
| 1893 | Adrb1 | 1.303086 | 0.0004 |
| 19867 | Mir145a | 1.300328 | 0.010666 |
| 30386 | Traf3ip3 | 1.300084 | 2.91E-16 |
| 22463 | Nxph3 | 1.297299 | 2.18E-08 |
| 27813 | Slc18a1 | 1.295492 | 0.002793 |
| 5198 | Crym | 1.294994 | 1.42E-28 |
| 6325 | Dusp13 | 1.294902 | 0.000683 |
| 32155 | Wdr72 | 1.293496 | 3.15E-07 |
| 30747 | Trim63 | 1.292602 | 5.81E-05 |
| 18136 | Kcnma1 | 1.29208 | 1.16E-25 |
| 23987 | P2rx1 | 1.290518 | 8.90E-05 |
| 1593 | Ablim2 | 1.289354 | 0.000928 |
| 21975 | Nexn | 1.288491 | 6.30E-41 |
| 1878 | Adora1 | 1.288278 | 8.40E-06 |
| 6336 | Dusp27 | 1.28614 | 5.10E-11 |
| 30477 | Trarg1 | 1.280849 | 0.000824 |
| 1409 | A630001G21Rik | 1.280371 | 1.69E-13 |
| 22144 | Nnmt | 1.275807 | 0.000109 |
| 6038 | Dll1 | 1.274494 | 1.04E-22 |
| 24102 | Parm1 | 1.269894 | 1.56E-76 |
| 21044 | Mmp17 | 1.268952 | 1.85E-27 |
| 25045 | Pou3f4 | 1.264303 | 0.002352 |
| 25817 | Rab27b | 1.258749 | 5.93E-13 |
| 6848 | Erbb3 | 1.258019 | 3.32E-54 |
| 7893 | Gata2 | 1.25769 | 1.36E-28 |
| 3578 | Cacna2d3 | 1.257089 | 6.66E-40 |
| 28552 | Soga3 | 1.256893 | 0.006413 |
| 3006 | Baalc | 1.255268 | 1.36E-20 |
| 19739 | Mgp | 1.253338 | 3.20E-39 |
| 17914 | Itga7 | 1.253213 | 7.97E-30 |
| 24315 | Pde1c | 1.252859 | 9.74E-18 |
| 1430 | A730049H05Rik | 1.252687 | 0.001598 |
| 7571 | Fndc5 | 1.251911 | 7.13E-37 |
| 28575 | Sox10 | 1.250876 | 1.97E-22 |
| 27256 | Scn7a | 1.249675 | 3.19E-08 |
| 28912 | Sstr1 | 1.247258 | 1.05E-05 |
| 2287 | Anks4b | 1.243004 | 6.05E-05 |
| 17933 | Itgb7 | 1.242267 | 1.72E-08 |
| 17038 | Hoxd4 | 1.23666 | 0.004543 |
| 7491 | Filip1 | 1.234199 | 8.80E-41 |
| 6282 | Dscaml1 | 1.228655 | 0.006425 |
| 21299 | Ms4a2 | 1.219343 | 0.010826 |
| 31454 | Vgll2 | 1.217467 | 1.21E-07 |
| 25444 | Prr33 | 1.217256 | 3.52E-17 |
| 16376 | Grik1 | 1.214836 | 4.43E-45 |
| 1801 | Adamtsl2 | 1.212297 | 1.64E-13 |
| 19741 | Mgst1 | 1.210147 | 6.88E-29 |
| 24914 | Pnma8b | 1.208065 | 1.14E-47 |
| 32225 | Wnt5b | 1.207012 | 1.09E-27 |
| 26924 | Rragd | 1.202636 | 2.18E-33 |
| 18061 | Kbtbd8 | 1.201539 | 2.36E-18 |
| 17039 | Hoxd8 | 1.201431 | 6.34E-12 |
| 3448 | Bves | 1.20089 | 1.27E-06 |
| 15073 | Gm6651 | 1.196412 | 0.01018 |
| 27282 | Scx | 1.195597 | 4.30E-10 |
| 4934 | Col28a1 | 1.194111 | 5.36E-05 |
| 2084 | Aldh1l2 | 1.19404 | 1.50E-22 |
| 24509 | Phex | 1.193261 | 6.00E-16 |
| 523 | 2610316D01Rik | 1.191958 | 1.62E-21 |
| 4599 | Chst8 | 1.188031 | 2.28E-11 |
| 17063 | Hrc | 1.18661 | 8.65E-12 |
| 19321 | Mal | 1.186058 | 6.22E-06 |
| 22368 | Ntrk3 | 1.184786 | 1.80E-59 |
| 3634 | Camk2a | 1.183298 | 1.23E-19 |
| 7563 | Fnd3c2 | 1.182593 | 0.003408 |
| 5215 | Csgalnact1 | 1.182394 | 8.35E-40 |
| 4962 | Colgalt2 | 1.182364 | 3.19E-25 |
| 16372 | Grid1 | 1.180683 | 1.61E-16 |
| 77 | 1700003F12Rik | 1.17974 | 0.001536 |
| 27454 | Serpina3n | 1.17923 | 0.00125 |
| 27632 | Shc3 | 1.174071 | 0.000238 |
| 18982 | Lpar4 | 1.169913 | 5.02E-46 |
| 30190 | Tmprss3 | 1.16658 | 0.000415 |
| 21710 | Naca | 1.164226 | 3.14E-52 |
| 5013 | Coro6 | 1.163538 | 0.000244 |
| 29470 | Tbx3 | 1.162774 | 5.65E-55 |
| 7664 | Frmpd1 | 1.162562 | 1.94E-17 |
| 2682 | Asic1 | 1.162144 | 8.50E-24 |
| 22271 | Nr2f2 | 1.161177 | 1.03E-57 |
| 17136 | Hspb1 | 1.160836 | 4.80E-17 |
| 22160 | Nol3 | 1.160375 | 7.01E-07 |
| 18897 | LOC102637806 | 1.156077 | 0.003841 |
| 6025 | Dleu7 | 1.154899 | 1.53E-06 |
| 3950 | Cckar | 1.151554 | 5.96E-14 |
| 2013 | Ajap1 | 1.151044 | 2.27E-06 |
| 28846 | Srl | 1.150935 | 6.21E-41 |
| 1643 | Ackr2 | 1.14965 | 0.001806 |
| 24027 | Pacsin1 | 1.148851 | 1.45E-07 |
| 17075 | Hs3st1 | 1.140702 | 5.95E-27 |
| 7815 | Gadl1 | 1.138267 | 4.55E-06 |
| 26386 | Rn7sk | 1.13693 | 0.007947 |
| 25641 | Ptgs1 | 1.135375 | 1.81E-32 |
| 32066 | Vtn | 1.134696 | 0.00319 |
| 4575 | Chrna4 | 1.134669 | 1.74E-09 |
| 1717 | Actn3 | 1.133486 | 6.88E-32 |
| 25387 | Prokr2 | 1.132774 | 0.00122 |
| 13710 | Gm34354 | 1.13229 | 4.41E-08 |
| 27247 | Scn1a | 1.128465 | 0.010887 |
| 18025 | Kank4 | 1.125492 | 3.90E-12 |
| 32669 | Zfp536 | 1.122745 | 1.61E-31 |
| 28970 | Stambpl1 | 1.122166 | 7.76E-32 |
| 16070 | Gm9745 | 1.12135 | 0.00756 |
| 1546 | Abcc6 | 1.117685 | 1.17E-09 |
| 4805 | Cmbl | 1.114165 | 4.94E-05 |
| 8116 | Glrb | 1.111858 | 3.65E-21 |
| 2219 | Ank1 | 1.111557 | 1.70E-19 |
| 27352 | Sel1l3 | 1.110273 | 2.39E-10 |
| 24871 | Plxdc2 | 1.107642 | 2.70E-56 |
| 7968 | Gdnf | 1.106184 | 1.99E-14 |
| 24708 | Pkp2 | 1.103696 | 0.000141 |
| 3477 | C1ql1 | 1.102357 | 3.37E-16 |
| 4212 | Cdh4 | 1.097601 | 6.28E-26 |
| 22326 | Nsg2 | 1.095685 | 2.85E-09 |
| 1802 | Adamtsl3 | 1.094759 | 1.95E-41 |
| 28995 | Stc2 | 1.094738 | 2.74E-11 |
| 22060 | Nkain2 | 1.094715 | 2.79E-05 |
| 30351 | Tpm2 | 1.094299 | 7.88E-59 |
| 17608 | Igsf11 | 1.091105 | 9.91E-06 |
| 3762 | Cavin4 | 1.090587 | 1.76E-09 |
| 29238 | Syt12 | 1.088887 | 8.07E-05 |
| 3635 | Camk2b | 1.088516 | 2.00E-07 |
| 30886 | Tspan32 | 1.087921 | 0.000301 |
| 2789 | Atp2a3 | 1.087023 | 5.21E-08 |
| 1889 | Adra1d | 1.084601 | 4.32E-06 |
| 18576 | Ky | 1.084198 | 2.86E-07 |
| 31462 | Vip | 1.082428 | 0.004955 |
| 1640 | Ache | 1.081617 | 2.54E-19 |
| 7964 | Gdf7 | 1.073685 | 1.87E-12 |
| 3275 | Bnc1 | 1.070964 | 3.46E-09 |
| 19128 | Lsamp | 1.070882 | 1.18E-13 |
| 1852 | Adgrg6 | 1.068492 | 1.20E-42 |
| 19328 | Mamdc2 | 1.067798 | 8.89E-05 |
| 1250 | 9130008F23Rik | 1.066458 | 1.06E-06 |
| 6935 | Esyt3 | 1.064904 | 0.000164 |
| 5379 | Cxcl12 | 1.06247 | 3.12E-57 |
| 27861 | Slc24a3 | 1.058671 | 8.58E-36 |
| 28962 | Stac3 | 1.054604 | 6.33E-17 |
| 25831 | Rab38 | 1.05421 | 1.57E-26 |
| 20411 | Mir6236 | 1.053895 | 1.17E-06 |
| 25464 | Prrxl1 | 1.052332 | 0.002611 |
| 1393 | A4galt | 1.051323 | 0.001902 |
| 16732 | Hapln3 | 1.048891 | 6.94E-14 |
| 25958 | Rarres1 | 1.047599 | 1.16E-05 |
| 15442 | Gm7819 | 1.043711 | 2.52E-11 |
| 25756 | Pygm | 1.043387 | 1.67E-19 |
| 30753 | Trim7 | 1.040442 | 4.93E-06 |
| 30332 | Tox3 | 1.039471 | 4.25E-16 |
| 29233 | Sypl2 | 1.037986 | 0.007655 |
| 7834 | Galnt13 | 1.037626 | 2.30E-13 |
| 5662 | Dapk2 | 1.036372 | 6.03E-12 |
| 1002 | 4931403E22Rik | 1.023413 | 0.003207 |
| 28844 | Srgn | 1.022962 | 1.51E-13 |
| 28124 | Slc8a3 | 1.017867 | 1.63E-22 |
| 29320 | Tafa1 | 1.011921 | 0.000354 |
| 21499 | Myb | 1.010791 | 2.29E-17 |
| 5240 | Cspg5 | 1.010657 | 5.11E-32 |
| 31265 | Unc5a | 1.00489 | 0.000169 |
| 24873 | Plxna2 | 1.004737 | 1.88E-46 |
| 29508 | Tcf4 | 1.002935 | 1.81E-60 |
| 5744 | Dcx | 1.000787 | 1.48E-15 |
| 30203 | Tmtc2 | 1.000623 | 4.28E-37 |
| 19625 | Meiosin | 0.997712 | 6.23E-08 |
| 6062 | Dmp1 | 0.995634 | 5.43E-05 |
| 5007 | Corin | 0.995403 | 4.80E-28 |
| 19009 | Lrfn2 | 0.993978 | 2.39E-07 |
| 2136 | Alox8 | 0.993907 | 0.00311 |
| 26442 | Rnf165 | 0.989529 | 3.78E-25 |
| 1619 | Acan | 0.986355 | 1.50E-07 |
| 17619 | Iho1 | 0.983887 | 1.88E-06 |
| 27244 | Scml4 | 0.98349 | 1.58E-05 |
| 4597 | Chst5 | 0.981346 | 0.01062 |
| 6579 | Ehf | 0.981144 | 0.000318 |
| 6469 | Ecel1 | 0.981064 | 5.47E-05 |
| 8749 | Gm12933 | 0.98059 | 0.009945 |
| 27018 | Runx3 | 0.979259 | 4.22E-21 |
| 27919 | Slc26a7 | 0.978348 | 1.40E-40 |
| 27758 | Slc10a1 | 0.977703 | 1.50E-06 |
| 4927 | Col20a1 | 0.976376 | 5.24E-19 |
| 29513 | Tchh | 0.972819 | 7.13E-07 |
| 2210 | Angpt4 | 0.970579 | 0.00228 |
| 18793 | Lims2 | 0.969977 | 0.001028 |
| 5219 | Csmd1 | 0.969918 | 6.74E-06 |
| 21032 | Mmd2 | 0.968978 | 2.02E-08 |
| 13244 | Gm2762 | 0.967292 | 0.011401 |
| 379 | 1700125H03Rik | 0.965552 | 0.008578 |
| 24382 | Pdzd2 | 0.96521 | 1.59E-37 |
| 12349 | Gm25005 | 0.965019 | 0.00544 |
| 7132 | Fam171b | 0.96378 | 2.87E-52 |
| 24879 | Plxnb3 | 0.962006 | 9.93E-07 |
| 24813 | Plekhh1 | 0.958375 | 2.96E-16 |
| 27389 | Sema6b | 0.957601 | 7.60E-19 |
| 19644 | Met | 0.956381 | 1.10E-28 |
| 18083 | Kcnd3 | 0.95631 | 0.00026 |
| 6663 | Elavl2 | 0.951292 | 0.001836 |
| 6099 | Dnah2 | 0.94939 | 2.22E-29 |
| 28529 | Snx31 | 0.948395 | 0.009393 |
| 21147 | Mpz | 0.94529 | 1.05E-06 |
| 2637 | Artn | 0.944492 | 4.66E-06 |
| 1837 | Adgrb3 | 0.94435 | 1.22E-10 |
| 6952 | Etv1 | 0.942141 | 5.47E-22 |
| 1438 | A830018L16Rik | 0.939559 | 4.68E-06 |
| 23950 | Otor | 0.939014 | 3.84E-08 |
| 4214 | Cdh6 | 0.936368 | 6.48E-26 |
| 29260 | T | 0.933509 | 0.004991 |
| 7127 | Fam169b | 0.933135 | 0.000149 |
| 27611 | Sh3gl2 | 0.927639 | 1.69E-10 |
| 16330 | Gprin3 | 0.923934 | 5.65E-10 |
| 8833 | Gm13302 | 0.922967 | 0.000318 |
| 4810 | Cmklr1 | 0.922771 | 1.63E-15 |
| 24334 | Pde8b | 0.922139 | 1.62E-07 |
| 22370 | Ntsr1 | 0.92134 | 0.004192 |
| 17716 | Ildr2 | 0.919193 | 0.000196 |
| 29573 | Tead4 | 0.918227 | 1.94E-13 |
| 9144 | Gm14634 | 0.916773 | 0.010626 |
| 22361 | Ntn4 | 0.916449 | 2.28E-07 |
| 7890 | Gask1b | 0.914501 | 8.32E-30 |
| 1816 | Adcy1 | 0.914298 | 5.25E-20 |
| 28462 | Snord73b | 0.913247 | 0.005447 |
| 4744 | Clec4e | 0.910046 | 0.001795 |
| 17058 | Hpse | 0.906959 | 3.34E-14 |
| 9397 | Gm15663 | 0.906555 | 2.08E-10 |
| 7693 | Fstl4 | 0.901538 | 0.00337 |
| 25508 | Prune2 | 0.901447 | 1.89E-11 |
| 18806 | Lingo3 | 0.900696 | 4.51E-05 |
| 27464 | Serpinb1a | 0.900285 | 8.16E-07 |
| 3858 | Ccdc177 | 0.900046 | 0.001694 |
| 19207 | Lypd1 | 0.895786 | 3.09E-13 |
| 4245 | Cdk5r1 | 0.894832 | 2.58E-14 |
| 25020 | Popdc3 | 0.894828 | 0.00491 |
| 24185 | Pcdh19 | 0.894633 | 9.17E-24 |
| 3688 | Car14 | 0.89386 | 0.000199 |
| 1986 | AI504432 | 0.892331 | 2.27E-09 |
| 29241 | Syt15 | 0.89206 | 0.001872 |
| 14490 | Gm50470 | 0.892035 | 0.000188 |
| 1707 | Actg2 | 0.890029 | 4.30E-07 |
| 27379 | Sema3g | 0.888892 | 3.16E-16 |
| 18432 | Kndc1 | 0.886714 | 5.25E-06 |
| 6254 | Dpysl4 | 0.884652 | 2.17E-25 |
| 7925 | Gbx2 | 0.881228 | 0.010542 |
| 29009 | Sting1 | 0.881166 | 6.10E-18 |
| 4872 | Cnr1 | 0.879882 | 2.91E-10 |
| 27825 | Slc1a6 | 0.879139 | 0.000487 |
| 19066 | Lrrc3b | 0.878571 | 6.29E-05 |
| 25899 | Radil | 0.876654 | 1.67E-14 |
| 26422 | Rnf128 | 0.87612 | 0.002961 |
| 7382 | Fcor | 0.874302 | 0.003445 |
| 19433 | Marchf3 | 0.872631 | 1.19E-06 |
| 24339 | Pdgfc | 0.87109 | 1.79E-24 |
| 7625 | Foxo6 | 0.870439 | 1.52E-06 |
| 13369 | Gm29773 | 0.869697 | 0.001713 |
| 23991 | P2rx5 | 0.867531 | 0.005035 |
| 2659 | Asb18 | 0.867175 | 0.003448 |
| 18051 | Kazald1 | 0.867128 | 1.25E-13 |
| 7835 | Galnt14 | 0.866543 | 0.000189 |
| 24321 | Pde4c | 0.864322 | 0.002211 |
| 29466 | Tbx2 | 0.863662 | 1.81E-25 |
| 27666 | Shroom3 | 0.863312 | 3.84E-08 |
| 19633 | Meox1 | 0.863254 | 7.85E-16 |
| 31201 | Ucn2 | 0.863203 | 4.99E-05 |
| 21774 | Nav3 | 0.862908 | 1.13E-18 |
| 26251 | Rhbdl3 | 0.861399 | 6.76E-16 |
| 13955 | Gm38576 | 0.861394 | 8.90E-05 |
| 28044 | Slc45a3 | 0.858417 | 3.98E-10 |
| 30790 | Trmt9b | 0.85768 | 1.17E-06 |
| 28477 | Snph | 0.854929 | 0.001416 |
| 7451 | Fgf9 | 0.854188 | 3.65E-08 |
| 17226 | Idi2 | 0.847436 | 0.000103 |
| 5011 | Coro2a | 0.846699 | 7.92E-06 |
| 7880 | Gas1 | 0.845753 | 1.33E-33 |
| 7731 | Fut9 | 0.844364 | 0.004387 |
| 18096 | Kcnh2 | 0.841951 | 9.11E-12 |
| 8056 | Gja4 | 0.841332 | 2.10E-13 |
| 19145 | Lsp1 | 0.839284 | 1.64E-37 |
| 4272 | Cdon | 0.837028 | 1.23E-36 |
| 18736 | Lfng | 0.836397 | 2.32E-16 |
| 19723 | Mgarp | 0.836375 | 0.000373 |
| 7596 | Foxd2os | 0.835453 | 2.85E-07 |
| 18089 | Kcne4 | 0.835425 | 8.06E-23 |
| 27912 | Slc26a10 | 0.834821 | 1.45E-05 |
| 4427 | Cfap100 | 0.833675 | 0.00685 |
| 18854 | Lmo3 | 0.833569 | 1.25E-06 |
| 2628 | Arsi | 0.830562 | 1.87E-16 |
| 29598 | Tenm1 | 0.828755 | 6.22E-20 |
| 5661 | Dapk1 | 0.828425 | 4.10E-29 |
| 5642 | Dab1 | 0.828415 | 8.79E-14 |
| 27078 | Sall1 | 0.826975 | 9.34E-06 |
| 21800 | Nceh1 | 0.826522 | 3.89E-08 |
| 3638 | Camk2n1 | 0.825558 | 1.25E-12 |
| 30234 | Tnfrsf21 | 0.82261 | 1.06E-24 |
| 22227 | Npm2 | 0.822181 | 0.006598 |
| 14664 | Gm54215 | 0.822118 | 6.52E-05 |
| 26996 | Rtn4r | 0.821187 | 1.70E-06 |
| 7437 | Fgf16 | 0.821051 | 0.002606 |
| 17655 | Il17rd | 0.820566 | 1.37E-30 |
| 21546 | Myl6b | 0.817293 | 1.07E-10 |
| 29063 | Strip2 | 0.815122 | 6.08E-09 |
| 18636 | Lars2 | 0.812063 | 0.000225 |
| 25952 | Rapgefl1 | 0.809942 | 1.92E-21 |
| 18820 | Lipo2 | 0.80976 | 0.008315 |
| 4932 | Col26a1 | 0.808484 | 4.68E-32 |
| 25517 | Psd | 0.806726 | 8.26E-06 |
| 4880 | Cntn1 | 0.8067 | 1.70E-12 |
| 17990 | Jazf1 | 0.805217 | 3.56E-25 |
| 6665 | Elavl4 | 0.80427 | 0.004596 |
| 21965 | Neurl3 | 0.803111 | 4.76E-06 |
| 18750 | Lgi4 | 0.802988 | 0.002673 |
| 18640 | Lat2 | 0.802544 | 0.001309 |
| 24462 | Pgam2 | 0.802046 | 3.15E-08 |
| 30863 | Tshz2 | 0.801796 | 1.02E-24 |
| 27375 | Sema3c | 0.799115 | 2.81E-20 |
| 17671 | Il1rl1 | 0.797886 | 2.24E-07 |
| 21786 | Ncam1 | 0.796534 | 3.21E-35 |
| 7838 | Galnt17 | 0.794702 | 4.14E-12 |
| 19466 | Matn2 | 0.793743 | 6.08E-34 |
| 29962 | Tmem132c | 0.792415 | 2.29E-32 |
| 5645 | Dach1 | 0.791678 | 4.99E-24 |
| 31269 | Unc5d | 0.789952 | 1.33E-10 |
| 24317 | Pde3a | 0.788458 | 2.95E-24 |
| 6302 | Dtna | 0.788258 | 9.10E-08 |
| 28307 | Smtnl2 | 0.786782 | 3.75E-10 |
| 2120 | Alkbh3os1 | 0.785527 | 0.00103 |
| 32582 | Zfp335os | 0.784125 | 0.001687 |
| 31421 | Vash1 | 0.783412 | 2.60E-21 |
| 2364 | Apba1 | 0.783308 | 7.42E-09 |
| 16922 | Hmcn1 | 0.783273 | 2.06E-32 |
| 4207 | Cdh22 | 0.782054 | 2.83E-09 |
| 24777 | Plcl1 | 0.780924 | 2.26E-09 |
| 25695 | Ptpro | 0.780042 | 2.04E-05 |
| 7592 | Foxc1 | 0.777209 | 2.19E-06 |
| 6809 | Epha5 | 0.776883 | 9.79E-07 |
| 7010 | Eya1 | 0.776056 | 2.07E-30 |
| 19122 | Lrrtm3 | 0.774827 | 1.00E-07 |
| 25644 | Ptgs2os2 | 0.773425 | 0.011555 |
| 21416 | Mtss1 | 0.772968 | 6.01E-28 |
| 4955 | Col9a1 | 0.772783 | 0.000168 |
| 3644 | Camkv | 0.771551 | 1.09E-12 |
| 22241 | Nptx1 | 0.771214 | 3.74E-12 |
| 4681 | Cldn1 | 0.769389 | 8.43E-19 |
| 22313 | Nrtn | 0.76686 | 0.011073 |
| 30821 | Trpm1 | 0.76635 | 0.000602 |
| 4627 | Cilp2 | 0.765843 | 0.008784 |
| 7445 | Fgf3 | 0.764986 | 4.78E-11 |
| 29469 | Tbx22 | 0.763766 | 2.90E-10 |
| 16929 | Hmga2-ps1 | 0.762105 | 0.0011 |
| 16864 | Heyl | 0.761909 | 1.08E-16 |
| 4918 | Col13a1 | 0.759578 | 6.58E-22 |
| 21114 | Moxd1 | 0.758382 | 2.60E-19 |
| 1620 | Acap1 | 0.756696 | 0.002258 |
| 27084 | Samd11 | 0.75589 | 0.002269 |
| 29948 | Tmem121 | 0.755763 | 8.11E-08 |
| 3711 | Carmil3 | 0.75553 | 8.01E-17 |
| 25924 | Ramp3 | 0.75302 | 4.63E-09 |
| 22062 | Nkain4 | 0.750531 | 0.000129 |
| 7483 | Fibin | 0.747853 | 7.53E-32 |
| 2977 | B3gnt5 | 0.747339 | 1.62E-14 |
| 6015 | Dkk1 | 0.745737 | 5.98E-11 |
| 6501 | Ednra | 0.745066 | 1.28E-26 |
| 22039 | Nim1k | 0.744705 | 0.000119 |
| 19492 | Mboat1 | 0.742368 | 1.06E-12 |
| 17335 | Igfbp6 | 0.742167 | 2.60E-05 |
| 3026 | Baiap2l2 | 0.74007 | 4.76E-07 |
| 30876 | Tspan12 | 0.738898 | 4.08E-10 |
| 3998 | Ccne1 | 0.738693 | 1.19E-25 |
| 29986 | Tmem158 | 0.737826 | 3.71E-11 |
| 28285 | Smoc2 | 0.737299 | 4.07E-33 |
| 18146 | Kcnn4 | 0.736415 | 0.000379 |
| 3185 | Bend5 | 0.736044 | 5.82E-24 |
| 28244 | Smim10l2a | 0.735538 | 1.00E-05 |
| 28560 | Sorcs1 | 0.734506 | 1.99E-09 |
| 7309 | Fbxo17 | 0.733159 | 2.85E-13 |
| 17777 | Insc | 0.732936 | 1.24E-10 |
| 5441 | Cyp27a1 | 0.732373 | 1.25E-06 |
| 7211 | Fam78a | 0.732183 | 9.73E-06 |
| 21773 | Nav2 | 0.731469 | 2.60E-24 |
| 22192 | Notum | 0.728529 | 1.94E-14 |
| 1798 | Adamts8 | 0.727493 | 2.06E-09 |
| 25703 | Ptprz1 | 0.727377 | 5.90E-30 |
| 17666 | Il1r1 | 0.726355 | 2.71E-13 |
| 28284 | Smoc1 | 0.724451 | 1.23E-18 |
| 19614 | Megf10 | 0.724415 | 2.64E-20 |
| 22582 | Olfm2 | 0.723421 | 5.62E-05 |
| 24726 | Pla2g4c | 0.723041 | 3.70E-05 |
| 7996 | Gfra1 | 0.72268 | 7.34E-14 |
| 29491 | Tceal6 | 0.722594 | 5.43E-08 |
| 29476 | Tbxa2r | 0.721409 | 0.000105 |
| 5547 | Cyp7b1 | 0.721214 | 2.47E-11 |
| 4566 | Chrm2 | 0.720888 | 7.27E-08 |
| 24189 | Pcdh9 | 0.720747 | 1.81E-09 |
| 3483 | C1qtnf2 | 0.720412 | 1.55E-07 |
| 26225 | Rgs16 | 0.720034 | 3.95E-19 |
| 24060 | Palmd | 0.719892 | 4.84E-12 |
| 17140 | Hspb6 | 0.7192 | 3.41E-13 |
| 6421 | E2f2 | 0.717913 | 2.08E-11 |
| 6207 | Dok6 | 0.717238 | 0.000256 |
| 28522 | Snx22 | 0.715914 | 0.003547 |
| 16419 | Gsap | 0.715397 | 1.09E-08 |
| 27374 | Sema3b | 0.714407 | 1.21E-10 |
| 4595 | Chst3 | 0.714318 | 5.81E-15 |
| 6311 | Dtx4 | 0.714071 | 1.04E-21 |
| 4111 | Cd40 | 0.713789 | 5.27E-06 |
| 6058 | Dmc1 | 0.713705 | 0.002885 |
| 17849 | Irag1 | 0.708476 | 3.26E-06 |
| 22656 | Optc | 0.708168 | 0.009519 |
| 3606 | Calcb | 0.707387 | 1.26E-05 |
| 5127 | Creb5 | 0.706787 | 2.97E-18 |
| 28173 | Slitrk2 | 0.705099 | 6.45E-05 |
| 18343 | Klhl33 | 0.704995 | 0.002683 |
| 4312 | Cecr2 | 0.703623 | 9.45E-09 |
| 2211 | Angptl1 | 0.702833 | 5.01E-25 |
| 26518 | Robo3 | 0.701945 | 0.000171 |
| 13187 | Gm266 | 0.700028 | 9.33E-05 |
| 28100 | Slc6a4 | 0.699666 | 0.000251 |
| 28760 | Spock3 | 0.698789 | 6.18E-10 |
| 27538 | Sfmbt2 | 0.693211 | 2.44E-12 |
| 24798 | Plekha6 | 0.692486 | 1.84E-11 |
| 29324 | Tafa5 | 0.691845 | 1.16E-11 |
| 4374 | Cep41 | 0.691085 | 1.44E-16 |
| 3639 | Camk2n2 | 0.689793 | 0.000316 |
| 27820 | Slc1a1 | 0.687502 | 0.003117 |
| 16710 | Hacd1 | 0.686569 | 0.000476 |
| 21024 | Mlxipl | 0.68607 | 0.009284 |
| 24910 | Pnma2 | 0.685142 | 3.33E-05 |
| 25968 | Rasal3 | 0.683347 | 0.01106 |
| 26166 | Ret | 0.683083 | 0.000234 |
| 27587 | Sh2b2 | 0.682557 | 2.06E-11 |
| 29780 | Thy1 | 0.678678 | 2.82E-20 |
| 6430 | E330013P04Rik | 0.678377 | 0.004933 |
| 17986 | Jam2 | 0.67759 | 1.07E-08 |
| 2911 | AW011738 | 0.676443 | 3.28E-06 |
| 19649 | Metrn | 0.676436 | 1.97E-12 |
| 16928 | Hmga2 | 0.675617 | 2.03E-22 |
| 3587 | Cacng4 | 0.674852 | 5.76E-12 |
| 19116 | Lrrn2 | 0.674604 | 3.10E-07 |
| 10183 | Gm18980 | 0.674191 | 0.002676 |
| 21106 | Morrbid | 0.674186 | 0.000955 |
| 6010 | Disp1 | 0.672271 | 3.43E-18 |
| 7743 | Fxyd7 | 0.669778 | 3.69E-05 |
| 6771 | Entpd3 | 0.668736 | 6.47E-13 |
| 2080 | Aldh1a3 | 0.667357 | 2.00E-21 |
| 7889 | Gask1a | 0.666205 | 3.56E-07 |
| 30972 | Ttll11 | 0.665939 | 0.001243 |
| 24901 | Pnck | 0.665919 | 2.30E-07 |
| 524 | 2610318N02Rik | 0.665792 | 3.82E-05 |
| 3179 | Bean1 | 0.664447 | 7.61E-06 |
| 24796 | Plekha4 | 0.663965 | 5.65E-07 |
| 27392 | Sema7a | 0.662229 | 1.52E-13 |
| 4648 | Ckb | 0.662219 | 9.26E-10 |
| 6250 | Dpyd | 0.660951 | 1.29E-14 |
| 6098 | Dnah17 | 0.65969 | 2.42E-05 |
| 5091 | Cpsf4l | 0.659272 | 0.000621 |
| 25699 | Ptprt | 0.658722 | 5.36E-06 |
| 19555 | Mdga1 | 0.657705 | 2.71E-13 |
| 29511 | Tcf7l2 | 0.657149 | 3.02E-21 |
| 6253 | Dpysl3 | 0.655736 | 2.46E-25 |
| 24801 | Plekhb1 | 0.65543 | 0.002638 |
| 27378 | Sema3f | 0.654535 | 5.59E-19 |
| 17214 | Icosl | 0.653412 | 3.07E-07 |
| 4952 | Col7a1 | 0.652352 | 5.43E-15 |
| 26985 | Rtl5 | 0.652327 | 2.57E-12 |
| 2449 | Ar | 0.652084 | 0.000327 |
| 6741 | Enc1 | 0.651662 | 1.20E-18 |
| 22229 | Npnt | 0.651493 | 3.61E-28 |
| 18715 | Lef1 | 0.651373 | 1.14E-19 |
| 16383 | Grin2a | 0.649482 | 4.19E-06 |
| 18786 | Limch1 | 0.649343 | 1.99E-24 |
| 29209 | Sync | 0.648419 | 0.002829 |
| 7636 | Foxs1 | 0.646983 | 0.004831 |
| 32678 | Zfp568 | 0.646886 | 2.30E-21 |
| 17854 | Irak3 | 0.644504 | 0.005836 |
| 8123 | Gls2 | 0.64372 | 0.002296 |
| 28999 | Steap4 | 0.643345 | 3.51E-07 |
| 30205 | Tmtc4 | 0.643276 | 4.86E-17 |
| 6813 | Ephb1 | 0.642854 | 3.21E-09 |
| 29487 | Tcea3 | 0.641451 | 2.05E-07 |
| 7488 | Fign | 0.641055 | 2.38E-08 |
| 7297 | Fbxl22 | 0.64032 | 0.008858 |
| 25944 | Rap2b | 0.6398 | 4.40E-12 |
| 30008 | Tmem178b | 0.63958 | 0.002492 |
| 19733 | Mgat5 | 0.639457 | 3.86E-17 |
| 5075 | Cpne2 | 0.637524 | 3.00E-10 |
| 2231 | Ankle1 | 0.635978 | 5.70E-11 |
| 26134 | Reep6 | 0.63434 | 0.000246 |
| 6047 | Dlx4os | 0.633001 | 7.13E-06 |
| 6563 | Egfl8 | 0.632896 | 0.000481 |
| 24403 | Peg12 | 0.630628 | 3.44E-11 |
| 32380 | Zbtb18 | 0.629675 | 2.65E-16 |
| 16854 | Hes6 | 0.629297 | 4.82E-12 |
| 29883 | Tm6sf1 | 0.62891 | 2.61E-08 |
| 3994 | Ccnd1 | 0.628672 | 9.85E-17 |
| 4915 | Col11a1 | 0.628612 | 2.12E-23 |
| 2964 | B3galt1 | 0.628586 | 2.35E-06 |
| 2307 | Antxr1 | 0.627609 | 2.94E-26 |
| 3766 | Cbfa2t3 | 0.62715 | 1.60E-13 |
| 25120 | Ppp1r14b | 0.62454 | 1.16E-12 |
| 30987 | Ttyh1 | 0.623684 | 9.96E-05 |
| 25074 | Ppfia4 | 0.623484 | 1.90E-13 |
| 24905 | Pnldc1 | 0.623023 | 0.001891 |
| 21482 | Mustn1 | 0.622654 | 0.000524 |
| 18240 | Kif1a | 0.621798 | 4.58E-15 |
| 29858 | Tln2 | 0.620125 | 3.38E-19 |
| 6001 | Dipk1c | 0.618478 | 8.25E-11 |
| 7303 | Fbxl8 | 0.618056 | 0.010511 |
| 4515 | Chd7 | 0.617764 | 1.39E-11 |
| 25792 | Rab11fip1 | 0.616567 | 0.000198 |
| 25742 | Pxdc1 | 0.615124 | 2.22E-11 |
| 2020 | Ak4 | 0.614842 | 1.24E-10 |
| 24674 | Pitpnm3 | 0.614543 | 1.98E-12 |
| 29037 | Stmn2 | 0.613602 | 0.000118 |
| 26000 | Rb1 | 0.612917 | 5.92E-13 |
| 25998 | Raver2 | 0.612588 | 5.69E-17 |
| 6549 | Efna3 | 0.610578 | 5.51E-08 |
| 28155 | Slf1 | 0.609799 | 1.46E-15 |
| 3826 | Ccdc134 | 0.609364 | 5.40E-12 |
| 28324 | Snap91 | 0.607567 | 0.000348 |
| 4349 | Cenpv | 0.607476 | 1.02E-08 |
| 7668 | Frmpd4 | 0.607262 | 0.003406 |
| 18776 | Lifr | 0.607188 | 2.44E-15 |
| 18852 | Lmo1 | 0.607054 | 4.84E-10 |
| 32263 | Xkr5 | 0.606471 | 2.37E-06 |
| 18571 | Ksr1 | 0.606438 | 4.73E-18 |
| 6754 | Eno3 | 0.604651 | 4.04E-10 |
| 28034 | Slc43a1 | 0.604476 | 5.73E-12 |
| 4328 | Cemip | 0.603995 | 0.00031 |
| 6568 | Egln3 | 0.603955 | 4.02E-07 |
| 17174 | Hunk | 0.603127 | 1.95E-19 |
| 28961 | Stac2 | 0.602748 | 6.74E-07 |
| 32240 | Wscd2 | 0.602323 | 9.95E-09 |
| 28304 | Sms | 0.60164 | 4.73E-12 |
| 9632 | Gm17366 | 0.601476 | 0.000264 |
| 29903 | Tmc6 | 0.601269 | 5.09E-10 |
| 30236 | Tnfrsf23 | 0.600909 | 0.001823 |
| 27544 | Sfrp4 | 0.60052 | 3.68E-14 |
| 18305 | Klf5 | 0.596755 | 6.43E-08 |
| 2292 | Ano1 | 0.596439 | 1.76E-13 |
| 27056 | S100a9 | 0.595882 | 0.00732 |
| 27941 | Slc2a3 | 0.595583 | 2.11E-10 |
| 24203 | Pcdhac2 | 0.594036 | 3.80E-07 |
| 2050 | Akr1b3 | 0.593871 | 3.49E-09 |
| 30361 | Tppp3 | 0.593719 | 2.83E-11 |
| 21772 | Nav1 | 0.591583 | 7.13E-21 |
| 2166 | Amigo1 | 0.590728 | 1.05E-11 |
| 22315 | Nrxn2 | 0.590276 | 0.000235 |
| 28557 | Sorbs2 | 0.588346 | 2.92E-07 |
| 22202 | Npas2 | 0.588078 | 0.00017 |
| 21926 | Negr1 | 0.586397 | 1.94E-14 |
| 31211 | Ufsp1 | 0.58603 | 0.001571 |
| 28350 | Snhg7 | 0.585524 | 1.34E-07 |
| 3133 | Bcat1 | 0.585308 | 1.29E-11 |
| 19257 | Macrod1 | 0.584161 | 8.22E-05 |
| 19001 | Lratd2 | 0.584075 | 3.56E-06 |
| 21955 | Net1 | 0.583715 | 3.30E-15 |
| 17327 | Igf2os | 0.583275 | 4.30E-08 |
| 1927 | Agap2 | 0.583048 | 2.00E-11 |
| 22189 | Notch3 | 0.58278 | 3.76E-15 |
| 2762 | Atoh8 | 0.582392 | 6.00E-06 |
| 5239 | Cspg4b | 0.582006 | 7.22E-15 |
| 27938 | Slc2a12 | 0.581817 | 0.004854 |
| 6905 | Esm1 | 0.58118 | 0.001759 |
| 17692 | Il2rg | 0.581046 | 0.000983 |
| 16532 | Gxylt2 | 0.5802 | 2.55E-19 |
| 9560 | Gm16617 | 0.579635 | 0.001348 |
| 6046 | Dlx4 | 0.579561 | 1.52E-08 |
| 21051 | Mmp23 | 0.577842 | 3.79E-06 |
| 18122 | Kcnk1 | 0.57547 | 3.46E-05 |
| 17616 | Igsf9b | 0.575009 | 2.60E-07 |
| 24153 | Pax9 | 0.574539 | 8.93E-21 |
| 6459 | Ebf2 | 0.574463 | 7.63E-06 |
| 27673 | Siah3 | 0.57425 | 0.001328 |
| 6705 | Emb | 0.57363 | 8.09E-13 |
| 16908 | Hivep3 | 0.573545 | 1.94E-05 |
| 24314 | Pde1b | 0.572616 | 4.27E-07 |
| 29561 | Tdrd1 | 0.57261 | 0.001318 |
| 28074 | Slc5a3 | 0.572012 | 1.78E-15 |
| 19760 | Mid1 | 0.571719 | 5.58E-13 |
| 512 | 2610027K06Rik | 0.57107 | 0.004049 |
| 24283 | Pcsk5 | 0.570327 | 3.04E-11 |
| 16764 | Hbegf | 0.570303 | 2.62E-05 |
| 5291 | Ctf1 | 0.569937 | 2.44E-09 |
| 30887 | Tspan33 | 0.569471 | 0.001478 |
| 4397 | Cerox1 | 0.569317 | 0.000165 |
| 3182 | Begain | 0.569296 | 1.41E-12 |
| 18966 | Lockd | 0.567849 | 0.000118 |
| 3038 | Barx1 | 0.566868 | 3.86E-13 |
| 6063 | Dmpk | 0.566166 | 2.54E-13 |
| 27814 | Slc18a2 | 0.565963 | 0.000349 |
| 7527 | Flnc | 0.565645 | 2.71E-16 |
| 5050 | Cpa2 | 0.564859 | 0.0003 |
| 7163 | Fam20c | 0.564135 | 4.55E-07 |
| 31735 | Vmn1r43 | 0.564097 | 0.002829 |
| 9435 | Gm15867 | 0.563826 | 0.009077 |
| 5204 | Csdc2 | 0.561104 | 8.39E-07 |
| 7439 | Fgf18 | 0.560744 | 1.00E-10 |
| 17876 | Irs4 | 0.560046 | 0.000833 |
| 3261 | Bmp4 | 0.559106 | 1.72E-19 |
| 19123 | Lrrtm4 | 0.558531 | 9.56E-05 |
| 29248 | Syt6 | 0.558507 | 0.000208 |
| 2373 | Apcdd1 | 0.55845 | 4.53E-17 |
| 16371 | Gria4 | 0.557925 | 7.29E-09 |
| 7391 | Fdps | 0.557858 | 0.00017 |
| 3977 | Ccl9 | 0.557156 | 0.000132 |
| 29024 | Stk32a | 0.554279 | 0.000491 |
| 26214 | Rgma | 0.55373 | 2.99E-12 |
| 26529 | Rora | 0.553481 | 4.30E-12 |
| 16730 | Hapln1 | 0.553364 | 8.40E-09 |
| 25509 | Prx | 0.552367 | 3.80E-10 |
| 26269 | Rhoj | 0.552261 | 4.60E-17 |
| 30864 | Tshz3 | 0.551149 | 4.04E-09 |
| 27950 | Slc30a10 | 0.550825 | 0.002064 |
| 19339 | Man2a1 | 0.549701 | 7.69E-17 |
| 24288 | Pcx | 0.549053 | 8.51E-10 |
| 16903 | Hipk4 | 0.549045 | 0.000613 |
| 459 | 2310009B15Rik | 0.548895 | 0.00046 |
| 5916 | Depp1 | 0.54779 | 0.000746 |
| 15944 | Gm9300 | 0.547322 | 0.006233 |
| 26473 | Rnf225 | 0.546725 | 0.011547 |
| 17611 | Igsf3 | 0.546004 | 8.12E-17 |
| 25985 | Rasl12 | 0.545917 | 1.03E-05 |
| 18849 | Lmnb2 | 0.545202 | 1.27E-13 |
| 7481 | Fhod3 | 0.54413 | 1.84E-06 |
| 5044 | Cox7c | 0.544122 | 2.73E-05 |
| 4160 | Cdc25b | 0.543732 | 3.37E-14 |
| 29901 | Tmc4 | 0.543273 | 0.000201 |
| 29054 | Stra6 | 0.543222 | 6.44E-05 |
| 27497 | Serpini1 | 0.54304 | 0.002679 |
| 17955 | Itpr3 | 0.542558 | 1.13E-12 |
| 2779 | Atp1a2 | 0.542335 | 8.40E-05 |
| 10618 | Gm21009 | 0.539885 | 0.001473 |
| 19348 | Maneal | 0.539404 | 0.000206 |
| 19362 | Map2k1 | 0.539032 | 2.60E-14 |
| 3557 | Cabp1 | 0.538462 | 0.002233 |
| 18246 | Kif21b | 0.538458 | 2.50E-10 |
| 6828 | Epop | 0.538333 | 9.65E-05 |
| 25101 | Ppm1j | 0.537209 | 0.004297 |
| 24812 | Plekhg6 | 0.536386 | 0.003719 |
| 21040 | Mmp13 | 0.536234 | 2.71E-06 |
| 32533 | Zfp202 | 0.535133 | 7.97E-08 |
| 2892 | Aunip | 0.532926 | 0.003467 |
| 27390 | Sema6c | 0.53256 | 3.38E-10 |
| 4920 | Col15a1 | 0.532099 | 1.08E-14 |
| 24123 | Pask | 0.531664 | 5.83E-12 |
| 24528 | Phf24 | 0.531212 | 1.12E-05 |
| 25977 | Rasgrp2 | 0.531117 | 8.44E-06 |
| 21337 | Msra | 0.530582 | 1.73E-05 |
| 31009 | Tubb6 | 0.530215 | 8.86E-17 |
| 3698 | Car9 | 0.527666 | 0.002515 |
| 28935 | St3gal1 | 0.527179 | 1.05E-15 |
| 16923 | Hmcn2 | 0.5271 | 0.005145 |
| 3142 | Bcl11a | 0.526595 | 5.95E-13 |
| 17237 | Iffo1 | 0.526298 | 3.50E-11 |
| 18652 | Lca5 | 0.526212 | 8.88E-09 |
| 31259 | Unc13c | 0.526069 | 2.87E-06 |
| 18783 | Lilrb4b | 0.525681 | 0.000494 |
| 28997 | Steap2 | 0.525255 | 1.57E-11 |
| 3284 | Boc | 0.524996 | 1.92E-14 |
| 5644 | Dab2ip | 0.524759 | 6.43E-15 |
| 24844 | Plp1 | 0.524684 | 3.09E-06 |
| 7218 | Fam83d | 0.523627 | 3.09E-10 |
| 6577 | Ehd3 | 0.523142 | 2.09E-05 |
| 28938 | St3gal4 | 0.523116 | 6.23E-14 |
| 24586 | Pif1 | 0.522749 | 7.92E-08 |
| 16348 | Gramd1b | 0.522349 | 5.48E-13 |
| 5058 | Cpe | 0.519137 | 3.53E-14 |
| 25657 | Ptn | 0.518645 | 1.08E-15 |
| 30796 | Troap | 0.518495 | 1.07E-09 |
| 3720 | Cask | 0.518143 | 5.14E-14 |
| 27307 | Sdk2 | 0.517585 | 4.93E-11 |
| 14826 | Gm5873 | 0.516711 | 5.53E-06 |
| 30131 | Tmem59l | 0.516639 | 3.60E-06 |
| 17336 | Igfbp7 | 0.516575 | 1.38E-09 |
| 30341 | Tpd52l1 | 0.514828 | 7.34E-06 |
| 6264 | Draxin | 0.514645 | 0.001806 |
| 8099 | Glipr2 | 0.514551 | 1.03E-12 |
| 19012 | Lrfn5 | 0.514357 | 0.006072 |
| 16239 | Gpm6b | 0.513413 | 7.51E-13 |
| 17787 | Insyn1 | 0.513269 | 5.77E-12 |
| 26966 | Rspo2 | 0.512672 | 5.36E-10 |
| 16603 | H2-T10 | 0.512404 | 0.00882 |
| 24725 | Pla2g4b | 0.511233 | 0.006957 |
| 19117 | Lrrn3 | 0.510611 | 8.28E-05 |
| 3573 | Cacna1h | 0.50904 | 3.72E-11 |
| 27080 | Sall3 | 0.508526 | 9.72E-06 |
| 18856 | Lmo7 | 0.508303 | 1.50E-11 |
| 17322 | Igf1r | 0.507442 | 4.46E-15 |
| 30215 | Tnfaip2 | 0.506645 | 0.000346 |
| 1769 | Adam33 | 0.506492 | 5.29E-12 |
| 24392 | Pdzrn4 | 0.506365 | 0.0039 |
| 25940 | Rap1gap2 | 0.50577 | 0.000397 |
| 22243 | Nptxr | 0.505614 | 9.73E-10 |
| 32150 | Wdr62 | 0.504759 | 1.04E-07 |
| 32215 | Wnt10a | 0.503895 | 4.14E-05 |
| 7595 | Foxd2 | 0.503564 | 0.005437 |
| 2500 | Arhgap33 | 0.503332 | 3.86E-13 |
| 24085 | Papss2 | 0.50278 | 2.71E-09 |
| 30893 | Tspan9 | 0.502218 | 3.78E-15 |
| 25673 | Ptpn21 | 0.501585 | 8.40E-09 |
| 27639 | Shfl | 0.501296 | 1.54E-07 |
| 3534 | C630043F03Rik | 0.500459 | 8.16E-10 |

**Table 7. Upregulated DEGs of dental mesenchyme of the lingual (Cap-L+2D) part after cultured *in vitro* for two days at the cap stage than of buccal (Cap-B+2D) part after cultured *in vitro* for two days at the cap stage**

|  | SYMBOL | log2FoldChange | pvalue |
| --- | --- | --- | --- |
| 18460 | Krt13 | -11.0151 | 2.93E-63 |
| 18484 | Krt4 | -9.82151 | 7.30E-60 |
| 28795 | Sprr3 | -7.5453 | 8.08E-19 |
| 6290 | Dsg3 | -6.55809 | 2.78E-54 |
| 18488 | Krt6a | -6.36532 | 6.56E-135 |
| 19321 | Mal | -6.33339 | 2.44E-13 |
| 5490 | Cyp2f2 | -6.2559 | 1.23E-27 |
| 6286 | Dsg1a | -6.19681 | 1.07E-37 |
| 30179 | Tmprss11a | -6.18874 | 1.23E-23 |
| 28781 | Sprr1a | -6.02519 | 4.46E-22 |
| 18489 | Krt6b | -5.99758 | 2.47E-25 |
| 18462 | Krt15 | -5.83443 | 7.47E-260 |
| 18466 | Krt19 | -5.59333 | 2.75E-39 |
| 22079 | Nkx2-3 | -5.55489 | 6.34E-16 |
| 27640 | Shh | -5.47086 | 6.70E-10 |
| 28739 | Spink5 | -5.45332 | 1.47E-12 |
| 16425 | Gsdmc | -5.22937 | 1.27E-11 |
| 30185 | Tmprss11g | -4.98737 | 2.79E-08 |
| 7116 | Fam162b | -4.93428 | 9.13E-21 |
| 30180 | Tmprss11b | -4.8801 | 1.40E-37 |
| 19180 | Ly6d | -4.84376 | 8.12E-20 |
| 27468 | Serpinb3a | -4.7965 | 1.24E-09 |
| 6287 | Dsg1b | -4.75184 | 1.65E-11 |
| 28101 | Slc6a5 | -4.59746 | 1.16E-17 |
| 32769 | Zfp750 | -4.51834 | 3.55E-44 |
| 6740 | Enam | -4.50621 | 3.21E-10 |
| 22279 | Nr5a2 | -4.48624 | 6.65E-228 |
| 4889 | Cntnap4 | -4.40994 | 1.08E-117 |
| 2878 | AU015836 | -4.39125 | 1.72E-21 |
| 2287 | Anks4b | -4.31706 | 9.12E-08 |
| 28333 | Sncb | -4.3087 | 1.48E-07 |
| 30892 | Tspan8 | -4.3011 | 1.53E-178 |
| 27030 | Rxfp2 | -4.30043 | 3.86E-81 |
| 5187 | Crybg2 | -4.27053 | 7.94E-21 |
| 18463 | Krt16 | -4.25544 | 7.46E-15 |
| 30187 | Tmprss13 | -4.23724 | 1.98E-14 |
| 4922 | Col17a1 | -4.19621 | 2.79E-129 |
| 18487 | Krt5 | -4.19356 | 2.76E-215 |
| 16942 | Hmgcs2 | -4.18095 | 4.55E-134 |
| 22580 | Olah | -4.15308 | 7.44E-17 |
| 5506 | Cyp2w1 | -4.14491 | 5.23E-23 |
| 5256 | Cst8 | -4.11769 | 5.51E-07 |
| 30708 | Trim29 | -4.0895 | 4.80E-83 |
| 7998 | Gfra3 | -4.08589 | 7.46E-107 |
| 23999 | P2ry14 | -4.07108 | 6.51E-84 |
| 27046 | S100a14 | -4.06717 | 7.14E-46 |
| 18815 | Lipi | -4.05526 | 8.49E-18 |
| 31290 | Upk3bl | -4.03485 | 1.66E-23 |
| 19324 | Mall | -4.02418 | 7.87E-17 |
| 7445 | Fgf3 | -3.99876 | 1.04E-40 |
| 18457 | Krt1 | -3.95466 | 4.36E-32 |
| 6061 | Dmkn | -3.92471 | 1.71E-07 |
| 4710 | Cldn4 | -3.91157 | 2.38E-56 |
| 10184 | Gm18981 | -3.89535 | 2.86E-40 |
| 16726 | Hand2os1 | -3.85588 | 5.23E-75 |
| 7599 | Foxe1 | -3.84043 | 2.91E-16 |
| 2441 | Aqp3 | -3.83823 | 6.88E-58 |
| 30182 | Tmprss11d | -3.83685 | 5.37E-06 |
| 7613 | Foxl1 | -3.83418 | 1.33E-24 |
| 19172 | Luzp2 | -3.83402 | 1.21E-72 |
| 17096 | Hsd17b2 | -3.82827 | 7.51E-05 |
| 18461 | Krt14 | -3.81717 | 7.29E-183 |
| 16725 | Hand2 | -3.81123 | 9.52E-69 |
| 5438 | Cyp26a1 | -3.80456 | 4.21E-43 |
| 29291 | Tacstd2 | -3.74601 | 6.02E-37 |
| 3623 | Calml3 | -3.74196 | 1.32E-09 |
| 7452 | Fgfbp1 | -3.74081 | 5.21E-16 |
| 24907 | Pnliprp1 | -3.73565 | 3.23E-26 |
| 30190 | Tmprss3 | -3.71778 | 1.08E-05 |
| 24858 | Plppr5 | -3.70634 | 4.35E-42 |
| 22254 | Nr0b1 | -3.67784 | 1.33E-07 |
| 7402 | Fendrr | -3.66192 | 7.94E-216 |
| 19635 | Mep1a | -3.65972 | 1.86E-09 |
| 7739 | Fxyd3 | -3.62808 | 1.07E-11 |
| 13958 | Gm38684 | -3.60712 | 1.88E-16 |
| 30184 | Tmprss11f | -3.5998 | 0.000248 |
| 23972 | Ovol1 | -3.58453 | 1.81E-15 |
| 25613 | Ptch2 | -3.58305 | 9.75E-123 |
| 4195 | Cdh1 | -3.56901 | 1.14E-97 |
| 3773 | Cbln1 | -3.54146 | 8.30E-62 |
| 27539 | Sfn | -3.51399 | 7.32E-88 |
| 2675 | Ascl5 | -3.50759 | 1.10E-07 |
| 2081 | Aldh1a7 | -3.4984 | 1.07E-12 |
| 25516 | Psca | -3.46003 | 6.25E-05 |
| 447 | 2300002M23Rik | -3.44062 | 1.66E-07 |
| 16520 | Gucy2f | -3.40154 | 4.26E-12 |
| 1542 | Abcc2 | -3.39223 | 1.84E-13 |
| 17088 | Hsd11b1 | -3.38064 | 8.81E-104 |
| 7607 | Foxi3 | -3.37939 | 3.43E-10 |
| 16705 | H60c | -3.36936 | 0.000804 |
| 28584 | Sox2 | -3.35868 | 3.86E-21 |
| 26152 | Ren1 | -3.35077 | 3.76E-06 |
| 6926 | Esrp1 | -3.34656 | 6.74E-56 |
| 8062 | Gjb3 | -3.34127 | 4.04E-10 |
| 1780 | Adamdec1 | -3.33493 | 6.38E-10 |
| 27478 | Serpinb7 | -3.33448 | 1.95E-13 |
| 4847 | Cnmd | -3.32069 | 4.58E-09 |
| 21427 | Muc15 | -3.31042 | 2.95E-05 |
| 6293 | Dsp | -3.29172 | 2.40E-137 |
| 3868 | Ccdc187 | -3.29007 | 9.54E-10 |
| 2795 | Atp2c2 | -3.28282 | 0.001128 |
| 28053 | Slc4a1 | -3.27962 | 1.07E-06 |
| 27461 | Serpinb11 | -3.27727 | 0.000184 |
| 18464 | Krt17 | -3.27584 | 1.04E-125 |
| 8606 | Gm12371 | -3.2551 | 3.17E-07 |
| 27348 | Sectm1b | -3.25483 | 2.78E-10 |
| 16366 | Grhl3 | -3.24694 | 1.63E-27 |
| 30861 | Tshr | -3.24281 | 3.07E-51 |
| 17202 | Ibsp | -3.2293 | 2.59E-192 |
| 18486 | Krt42 | -3.20383 | 1.78E-06 |
| 4712 | Cldn6 | -3.18554 | 2.91E-19 |
| 19189 | Ly6g6e | -3.17612 | 1.25E-07 |
| 25263 | Prdm16os | -3.16563 | 8.17E-09 |
| 28817 | Sptlc3 | -3.15299 | 2.90E-08 |
| 18359 | Klk11 | -3.14386 | 0.000478 |
| 6288 | Dsg1c | -3.11499 | 0.002596 |
| 19621 | Meig1 | -3.1109 | 2.29E-12 |
| 32219 | Wnt2 | -3.10899 | 4.05E-72 |
| 7850 | Galr1 | -3.09466 | 0.000116 |
| 31306 | Urah | -3.08763 | 0.000676 |
| 28262 | Smim31 | -3.08247 | 0.002849 |
| 1792 | Adamts20 | -3.07692 | 3.39E-136 |
| 21917 | Nectin4 | -3.07535 | 1.61E-54 |
| 1256 | 9130024F11Rik | -3.07411 | 7.56E-101 |
| 8414 | Gm11597 | -3.07383 | 0.000597 |
| 27995 | Slc36a2 | -3.06478 | 1.30E-55 |
| 18597 | Lama1 | -3.0443 | 1.21E-135 |
| 27462 | Serpinb12 | -3.04127 | 1.34E-07 |
| 4714 | Cldn8 | -3.03356 | 0.000184 |
| 24709 | Pkp3 | -3.02491 | 3.64E-40 |
| 25262 | Prdm16 | -2.99326 | 1.93E-80 |
| 8063 | Gjb4 | -2.99244 | 0.00119 |
| 7081 | Fam110c | -2.9898 | 4.43E-12 |
| 5687 | Dbx2 | -2.98345 | 2.15E-15 |
| 21434 | Muc4 | -2.96304 | 0.000355 |
| 24412 | Penk | -2.94231 | 1.45E-117 |
| 1562 | Abcg8 | -2.93912 | 2.41E-06 |
| 2301 | Ano9 | -2.93786 | 2.30E-07 |
| 21195 | Mro | -2.93765 | 6.41E-15 |
| 29750 | Them5 | -2.93018 | 9.20E-05 |
| 16261 | Gpr149 | -2.92191 | 6.27E-11 |
| 4524 | Chgb | -2.90974 | 6.57E-36 |
| 18416 | Klrg1 | -2.89634 | 3.99E-07 |
| 28590 | Sox5 | -2.8946 | 2.64E-40 |
| 28605 | Sp5 | -2.89451 | 3.30E-32 |
| 19211 | Lypd3 | -2.89252 | 7.12E-28 |
| 27057 | S100b | -2.89231 | 6.90E-44 |
| 5372 | Cwh43 | -2.88503 | 0.000472 |
| 7263 | Fat2 | -2.8813 | 9.34E-19 |
| 9482 | Gm16136 | -2.87924 | 9.09E-09 |
| 28088 | Slc6a14 | -2.86577 | 1.89E-06 |
| 9053 | Gm14267 | -2.85509 | 5.45E-11 |
| 19252 | Mab21l4 | -2.85445 | 0.000566 |
| 19210 | Lypd2 | -2.84424 | 4.36E-05 |
| 4757 | Clic6 | -2.83329 | 1.27E-20 |
| 24498 | Phactr3 | -2.82158 | 1.14E-48 |
| 3884 | Ccdc3 | -2.81573 | 1.86E-131 |
| 25514 | Psapl1 | -2.80836 | 2.07E-05 |
| 2706 | Astn1 | -2.80707 | 2.88E-122 |
| 19627 | Meis2 | -2.80405 | 3.15E-122 |
| 2339 | Ap1m2 | -2.79476 | 8.19E-15 |
| 4218 | Cdhr1 | -2.79241 | 1.60E-26 |
| 31467 | Vit | -2.78675 | 2.36E-26 |
| 138 | 1700018A04Rik | -2.78666 | 1.35E-13 |
| 5348 | Ctxnd1 | -2.78117 | 4.59E-89 |
| 18138 | Kcnmb2 | -2.781 | 7.71E-16 |
| 5847 | Defb1 | -2.77708 | 0.000118 |
| 17257 | Ifi47 | -2.7763 | 1.00E-47 |
| 17757 | Inmt | -2.77567 | 0.000884 |
| 17656 | Il17re | -2.77301 | 1.70E-08 |
| 7547 | Fmo1 | -2.76642 | 8.70E-139 |
| 3175 | Bdkrb1 | -2.75184 | 3.25E-15 |
| 2089 | Aldh3b2 | -2.75141 | 4.73E-13 |
| 19569 | Me3 | -2.74993 | 5.18E-53 |
| 2713 | Asz1 | -2.74815 | 0.000326 |
| 31462 | Vip | -2.74762 | 0.010387 |
| 2292 | Ano1 | -2.74445 | 5.31E-83 |
| 21347 | Msx2 | -2.73728 | 5.43E-47 |
| 1824 | Adcy8 | -2.73546 | 1.92E-50 |
| 7063 | Fads2b | -2.73335 | 4.61E-08 |
| 6671 | Elf5 | -2.7327 | 6.96E-08 |
| 8092 | Gli1 | -2.71273 | 1.16E-103 |
| 6765 | Enpp6 | -2.71036 | 1.02E-08 |
| 26355 | Ripk4 | -2.70877 | 6.96E-23 |
| 25390 | Prom2 | -2.70705 | 2.54E-12 |
| 17269 | Ifitm5 | -2.69896 | 3.48E-71 |
| 27081 | Sall4 | -2.69514 | 0.000215 |
| 3974 | Ccl6 | -2.69178 | 1.19E-48 |
| 30971 | Ttll10 | -2.69113 | 0.000169 |
| 24781 | Plcxd3 | -2.68938 | 1.16E-06 |
| 24088 | Paqr5 | -2.68907 | 6.89E-11 |
| 27281 | Scube3 | -2.67843 | 2.66E-103 |
| 22276 | Nr4a2 | -2.67777 | 1.75E-133 |
| 7601 | Foxf1 | -2.66972 | 1.31E-150 |
| 19322 | Mal2 | -2.66367 | 9.48E-08 |
| 3204 | Bglap3 | -2.66061 | 6.95E-46 |
| 4591 | Chst13 | -2.65996 | 3.67E-16 |
| 27782 | Slc13a5 | -2.6507 | 2.07E-101 |
| 18747 | Lgi1 | -2.64573 | 3.70E-13 |
| 17094 | Hsd17b13 | -2.6451 | 0.005622 |
| 28942 | St6gal2 | -2.6398 | 1.28E-71 |
| 19027 | Lrp1b | -2.63737 | 4.14E-28 |
| 3202 | Bglap | -2.60996 | 2.71E-82 |
| 3697 | Car8 | -2.58986 | 2.10E-57 |
| 4833 | Cnfn | -2.58644 | 6.06E-05 |
| 27132 | Satb2 | -2.58435 | 4.78E-127 |
| 16318 | Gpr87 | -2.58415 | 0.001017 |
| 6927 | Esrp2 | -2.58187 | 1.76E-23 |
| 1759 | Adam23 | -2.57951 | 1.62E-106 |
| 26216 | Rgn | -2.57123 | 0.002655 |
| 4198 | Cdh12 | -2.56891 | 4.07E-19 |
| 7602 | Foxf2 | -2.56311 | 6.22E-80 |
| 26074 | Rbp4 | -2.56049 | 9.17E-46 |
| 16402 | Grm2 | -2.55279 | 0.002722 |
| 27573 | Sgms2 | -2.55154 | 8.32E-105 |
| 16810 | Hdnr | -2.54731 | 9.89E-15 |
| 23940 | Ostn | -2.5421 | 6.58E-51 |
| 3588 | Cacng5 | -2.53551 | 5.46E-08 |
| 5539 | Cyp4f39 | -2.53449 | 1.54E-06 |
| 22084 | Nkx3-1 | -2.53366 | 3.21E-07 |
| 23943 | Otof | -2.51913 | 1.20E-12 |
| 7593 | Foxc2 | -2.51405 | 7.73E-30 |
| 28081 | Slc5a9 | -2.51276 | 4.30E-06 |
| 6068 | Dmrta1 | -2.51199 | 5.21E-09 |
| 1991 | AI661453 | -2.51002 | 1.15E-16 |
| 1737 | Acvrl1 | -2.50555 | 3.28E-96 |
| 2132 | Alox12e | -2.50339 | 0.003693 |
| 25476 | Prss30 | -2.49369 | 0.00392 |
| 22653 | Oprk1 | -2.4868 | 0.003976 |
| 4417 | Ces2g | -2.48264 | 6.16E-09 |
| 19178 | Ly6c1 | -2.47999 | 2.53E-30 |
| 8077 | Gkn1 | -2.47705 | 9.20E-08 |
| 16365 | Grhl2 | -2.47169 | 9.45E-17 |
| 3953 | Ccl11 | -2.47136 | 6.48E-19 |
| 30288 | Tns4 | -2.46784 | 5.50E-09 |
| 6356 | Dynap | -2.46497 | 0.010312 |
| 4192 | Cdcp1 | -2.46406 | 5.56E-20 |
| 24776 | Plch2 | -2.45146 | 4.15E-30 |
| 27166 | Scara5 | -2.45115 | 6.41E-78 |
| 27446 | Serpina3f | -2.44806 | 9.57E-18 |
| 2130 | Alox12 | -2.44492 | 1.30E-27 |
| 18174 | Kctd19 | -2.43644 | 0.005491 |
| 24275 | Pcp4 | -2.43208 | 0.00091 |
| 1522 | Abca4 | -2.42842 | 1.31E-45 |
| 18604 | Lamb3 | -2.42293 | 6.97E-39 |
| 6800 | Epcam | -2.42075 | 9.14E-38 |
| 7282 | Fbp2 | -2.42008 | 0.00098 |
| 7412 | Fetub | -2.40902 | 2.45E-10 |
| 16235 | Gpihbp1 | -2.39772 | 1.98E-28 |
| 4928 | Col22a1 | -2.39204 | 1.61E-30 |
| 4532 | Chil5 | -2.39197 | 0.014321 |
| 30458 | Trank1 | -2.39066 | 3.80E-68 |
| 7594 | Foxd1 | -2.39018 | 1.14E-13 |
| 32191 | Wfdc6a | -2.3861 | 0.006185 |
| 5440 | Cyp26c1 | -2.38494 | 1.79E-06 |
| 4393 | Cer1 | -2.38422 | 0.002786 |
| 24854 | Plppr1 | -2.38354 | 5.21E-48 |
| 28136 | Slc9a9 | -2.3814 | 1.50E-85 |
| 4663 | Clca2 | -2.3732 | 1.37E-20 |
| 6683 | Elmod1 | -2.37304 | 1.45E-09 |
| 18978 | Loxl4 | -2.37071 | 5.09E-46 |
| 5916 | Depp1 | -2.34401 | 2.07E-15 |
| 18490 | Krt7 | -2.33742 | 1.40E-12 |
| 29498 | Tcerg1l | -2.33542 | 9.13E-22 |
| 25159 | Ppp2r2b | -2.33448 | 2.36E-43 |
| 14370 | Gm4841 | -2.33349 | 1.14E-27 |
| 3525 | C4b | -2.33075 | 8.03E-24 |
| 29289 | Tacr2 | -2.32673 | 0.000121 |
| 3724 | Casp12 | -2.31478 | 9.49E-42 |
| 4730 | Clec2g | -2.30745 | 4.43E-11 |
| 21060 | Mmrn1 | -2.30541 | 1.63E-35 |
| 24419 | Perp | -2.30361 | 4.47E-60 |
| 24551 | Phospho1 | -2.30327 | 5.75E-22 |
| 27619 | Sh3rf2 | -2.30187 | 6.41E-20 |
| 29704 | Tgfa | -2.30144 | 3.68E-28 |
| 5606 | D430041D05Rik | -2.30114 | 3.13E-13 |
| 4916 | Col11a2 | -2.29744 | 7.41E-75 |
| 27051 | S100a5 | -2.29493 | 0.00114 |
| 17840 | Iqcn | -2.2891 | 2.69E-06 |
| 28933 | St14 | -2.28794 | 1.91E-24 |
| 28569 | Sostdc1 | -2.28703 | 6.75E-91 |
| 16339 | Gpx2 | -2.27748 | 0.010424 |
| 3691 | Car3 | -2.27106 | 1.08E-79 |
| 1541 | Abcc12 | -2.27092 | 2.17E-05 |
| 19183 | Ly6g | -2.2675 | 0.010744 |
| 8015 | Ggt5 | -2.26473 | 2.91E-23 |
| 25773 | Qrfprl | -2.25846 | 0.000344 |
| 21764 | Nat8f3 | -2.25696 | 0.001106 |
| 2662 | Asb4 | -2.25604 | 3.85E-73 |
| 8564 | Gm12185 | -2.25495 | 0.000411 |
| 19179 | Ly6c2 | -2.25372 | 4.79E-06 |
| 6289 | Dsg2 | -2.2524 | 1.04E-28 |
| 26250 | Rhbdl2 | -2.25167 | 4.72E-20 |
| 27637 | She | -2.24993 | 2.24E-62 |
| 14661 | Gm5414 | -2.24751 | 0.011719 |
| 30470 | Trappc3l | -2.24702 | 0.000475 |
| 7408 | Fermt1 | -2.24325 | 7.32E-12 |
| 24940 | Pof1b | -2.23985 | 1.15E-08 |
| 24389 | Pdzk1ip1 | -2.23624 | 0.000117 |
| 1789 | Adamts18 | -2.23619 | 2.99E-39 |
| 3265 | Bmp8a | -2.22878 | 3.17E-07 |
| 18587 | L3mbtl4 | -2.22864 | 0.006962 |
| 29621 | Tesc | -2.2128 | 1.27E-14 |
| 16289 | Gpr25 | -2.21013 | 0.013106 |
| 7841 | Galnt3 | -2.19468 | 2.02E-20 |
| 21527 | Myh14 | -2.191 | 1.74E-22 |
| 18805 | Lingo2 | -2.18553 | 1.90E-25 |
| 24730 | Pla2g5 | -2.1805 | 0.000367 |
| 3203 | Bglap2 | -2.17083 | 1.30E-49 |
| 25065 | Ppbp | -2.16944 | 8.65E-06 |
| 2152 | Ambn | -2.16376 | 9.44E-69 |
| 6669 | Elf3 | -2.16215 | 9.63E-14 |
| 8065 | Gjb6 | -2.15972 | 0.009815 |
| 27524 | Sez6l | -2.15608 | 7.51E-49 |
| 6962 | Eva1c | -2.15301 | 6.17E-13 |
| 27579 | Sgpp2 | -2.15047 | 8.25E-33 |
| 3258 | Bmp2 | -2.1464 | 7.76E-79 |
| 3770 | Cblc | -2.14474 | 6.61E-05 |
| 6499 | Edn2 | -2.14443 | 4.50E-06 |
| 22652 | Oprd1 | -2.14125 | 5.14E-07 |
| 7022 | F13a1 | -2.13651 | 1.79E-15 |
| 6108 | Dnai1 | -2.12973 | 1.76E-07 |
| 4568 | Chrm4 | -2.12839 | 4.32E-22 |
| 22543 | Ocln | -2.12566 | 3.74E-08 |
| 30258 | Tnk1 | -2.11904 | 2.71E-12 |
| 24279 | Pcsk2 | -2.11827 | 9.03E-30 |
| 29746 | Thbs4 | -2.11623 | 2.42E-64 |
| 3029 | Bambi | -2.11095 | 8.12E-79 |
| 1439 | A830019L24Rik | -2.11012 | 2.14E-05 |
| 25835 | Rab3b | -2.1055 | 1.09E-27 |
| 26470 | Rnf222 | -2.1043 | 0.006067 |
| 4290 | Ceacam1 | -2.10395 | 1.20E-14 |
| 19215 | Lypd6b | -2.10346 | 1.19E-09 |
| 17161 | Htr2c | -2.09695 | 0.000391 |
| 5151 | Crispld2 | -2.09289 | 1.32E-77 |
| 1676 | Acpp | -2.0903 | 2.83E-31 |
| 6826 | Epn3 | -2.08638 | 7.41E-17 |
| 24850 | Plpp4 | -2.08408 | 0.008569 |
| 16876 | Hhip | -2.07921 | 2.70E-48 |
| 25122 | Ppp1r14c | -2.07886 | 3.86E-24 |
| 24313 | Pde1a | -2.07563 | 6.75E-51 |
| 21358 | Mtarc1 | -2.07165 | 3.95E-05 |
| 5266 | Cstdc5 | -2.06864 | 4.25E-05 |
| 7463 | Fgl2 | -2.0654 | 2.16E-43 |
| 6877 | Erich2 | -2.06276 | 1.83E-07 |
| 24707 | Pkp1 | -2.05801 | 3.45E-50 |
| 3109 | BC055402 | -2.05756 | 0.008419 |
| 3910 | Ccdc68 | -2.05706 | 1.59E-06 |
| 2320 | Anxa8 | -2.05563 | 4.61E-19 |
| 4054 | Cd177 | -2.04941 | 0.009022 |
| 4203 | Cdh18 | -2.04937 | 3.01E-18 |
| 6760 | Enpp1 | -2.0481 | 8.11E-64 |
| 30007 | Tmem178 | -2.04385 | 1.04E-21 |
| 2078 | Aldh1a1 | -2.04359 | 6.06E-10 |
| 25471 | Prss23 | -2.03696 | 5.97E-65 |
| 27494 | Serpinf2 | -2.03685 | 0.010043 |
| 4557 | Chp2 | -2.03538 | 0.009264 |
| 7767 | G630016G05Rik | -2.03533 | 7.31E-14 |
| 2086 | Aldh3a1 | -2.03171 | 5.57E-32 |
| 7651 | Frem1 | -2.02844 | 3.49E-73 |
| 7690 | Fst | -2.02297 | 1.03E-72 |
| 6279 | Dsc2 | -2.01666 | 6.59E-40 |
| 7992 | Gfod1 | -2.01485 | 1.00E-53 |
| 6315 | Duoxa1 | -2.01378 | 5.82E-08 |
| 26235 | Rgs5 | -2.0091 | 2.67E-44 |
| 16992 | Hopx | -2.00763 | 1.16E-09 |
| 18465 | Krt18 | -2.00756 | 5.58E-22 |
| 69 | 1700001K23Rik | -2.00336 | 0.000774 |
| 1635 | Ace2 | -2.0029 | 0.010436 |
| 27279 | Scube1 | -2.0021 | 1.25E-67 |
| 5221 | Csmd3 | -2.00016 | 6.80E-16 |
| 8969 | Gm13889 | -1.99778 | 2.05E-50 |
| 21149 | Mpzl2 | -1.99461 | 2.60E-12 |
| 25988 | Rassf10 | -1.99421 | 5.62E-10 |
| 2147 | Alx3 | -1.99357 | 3.31E-13 |
| 24076 | Panx3 | -1.99328 | 1.66E-66 |
| 3695 | Car6 | -1.99179 | 0.002124 |
| 28744 | Spint1 | -1.99029 | 5.41E-18 |
| 2079 | Aldh1a2 | -1.98554 | 9.90E-79 |
| 31286 | Upk1b | -1.98044 | 0.000298 |
| 6489 | Edaradd | -1.97996 | 1.47E-28 |
| 7040 | F830016B08Rik | -1.97827 | 9.80E-08 |
| 30243 | Tnfsf11 | -1.97785 | 1.06E-49 |
| 18592 | Lad1 | -1.97715 | 4.34E-25 |
| 4929 | Col23a1 | -1.97704 | 1.02E-66 |
| 18810 | Lipc | -1.97468 | 0.001876 |
| 29862 | Tlr12 | -1.9742 | 9.39E-05 |
| 24824 | Plet1 | -1.97049 | 0.004284 |
| 16358 | Grb7 | -1.9665 | 1.55E-07 |
| 16827 | Hecw1 | -1.96595 | 9.03E-10 |
| 2629 | Arsj | -1.96529 | 4.44E-09 |
| 25932 | Ranbp3l | -1.96467 | 2.09E-07 |
| 32228 | Wnt7b | -1.96281 | 2.20E-23 |
| 24739 | Plac8 | -1.9624 | 7.24E-10 |
| 29634 | Tex11 | -1.96163 | 0.0051 |
| 30874 | Tspan10 | -1.95838 | 0.00012 |
| 18817 | Lipm | -1.94961 | 0.008281 |
| 610 | 4833419F23Rik | -1.94839 | 0.007252 |
| 3095 | BC048671 | -1.94701 | 0.013803 |
| 32222 | Wnt3a | -1.94678 | 0.014274 |
| 25551 | Psmb11 | -1.9449 | 0.000441 |
| 2823 | Atp6v0a4 | -1.9384 | 0.003678 |
| 17620 | Iigp1 | -1.93081 | 1.44E-59 |
| 16304 | Gpr50 | -1.92659 | 2.86E-45 |
| 29730 | Tgtp2 | -1.92301 | 4.60E-16 |
| 17715 | Ildr1 | -1.91799 | 0.009236 |
| 3976 | Ccl8 | -1.91768 | 0.009531 |
| 7104 | Fam135b | -1.91581 | 1.16E-08 |
| 24919 | Pnoc | -1.9135 | 0.000138 |
| 9498 | Gm16223 | -1.91234 | 0.007564 |
| 24792 | Plek2 | -1.91116 | 5.55E-09 |
| 16361 | Greb1l | -1.90988 | 1.34E-43 |
| 5664 | Dapl1 | -1.90578 | 6.15E-06 |
| 18188 | Kdf1 | -1.904 | 1.68E-06 |
| 27633 | Shc4 | -1.89963 | 1.35E-30 |
| 1630 | Acbd7 | -1.89955 | 0.004177 |
| 32649 | Zfp488 | -1.8966 | 2.41E-13 |
| 19206 | Lynx1 | -1.8923 | 2.09E-42 |
| 6948 | Etnk2 | -1.89203 | 2.84E-32 |
| 6046 | Dlx4 | -1.89154 | 1.00E-07 |
| 18743 | Lgals7 | -1.88986 | 6.79E-18 |
| 2055 | Akr1c14 | -1.88237 | 2.50E-08 |
| 18606 | Lamc2 | -1.87998 | 7.30E-35 |
| 26968 | Rspo4 | -1.87826 | 2.50E-30 |
| 5501 | Cyp2j9 | -1.87452 | 3.53E-26 |
| 18088 | Kcne3 | -1.87104 | 1.52E-23 |
| 23035 | Or2v1 | -1.87081 | 0.000668 |
| 5361 | Cutal | -1.87038 | 0.011849 |
| 22314 | Nrxn1 | -1.8682 | 6.67E-32 |
| 6769 | Entpd1 | -1.868 | 4.79E-57 |
| 6759 | Enpep | -1.86671 | 1.23E-48 |
| 17646 | Il16 | -1.86641 | 3.78E-15 |
| 32239 | Wscd1 | -1.86273 | 2.38E-19 |
| 2075 | Alcam | -1.86165 | 4.59E-58 |
| 3041 | Batf | -1.86067 | 7.78E-34 |
| 7915 | Gbp2 | -1.8603 | 5.65E-37 |
| 30224 | Tnfrsf11b | -1.85813 | 2.38E-37 |
| 3440 | Btnl9 | -1.8545 | 1.96E-09 |
| 21702 | Naaa | -1.84778 | 2.67E-36 |
| 4953 | Col8a1 | -1.84522 | 2.12E-57 |
| 7817 | Gal | -1.83674 | 3.49E-05 |
| 1838 | Adgrd1 | -1.83636 | 1.11E-39 |
| 19383 | Map3k5 | -1.83551 | 1.24E-38 |
| 25612 | Ptch1 | -1.83034 | 5.80E-67 |
| 30746 | Trim62 | -1.83033 | 3.74E-58 |
| 7550 | Fmo4 | -1.82971 | 0.000244 |
| 30833 | Trpv3 | -1.82937 | 0.010759 |
| 18150 | Kcnq3 | -1.81788 | 9.67E-18 |
| 3881 | Ccdc27 | -1.81778 | 0.00367 |
| 6543 | Efhc2 | -1.81645 | 0.00703 |
| 2473 | Arg1 | -1.8153 | 1.80E-05 |
| 10419 | Gm20203 | -1.81497 | 0.008365 |
| 5386 | Cxcl3 | -1.8142 | 0.000228 |
| 2990 | B4galnt3 | -1.81301 | 9.30E-13 |
| 30079 | Tmem252 | -1.81169 | 1.81E-57 |
| 3950 | Cckar | -1.80936 | 0.000475 |
| 26553 | Rph3al | -1.80435 | 2.46E-18 |
| 6045 | Dlx3 | -1.80294 | 2.31E-31 |
| 2849 | Atp8b1 | -1.80188 | 5.51E-15 |
| 27186 | Scg2 | -1.80163 | 2.31E-22 |
| 26990 | Rtl9 | -1.8002 | 1.02E-36 |
| 22183 | Nos2 | -1.79999 | 8.25E-17 |
| 30713 | Trim30d | -1.79872 | 0.004521 |
| 1547 | Abcc8 | -1.79704 | 3.27E-19 |
| 27837 | Slc22a18 | -1.79037 | 1.84E-07 |
| 2421 | Apol9a | -1.79 | 0.012326 |
| 19269 | Maf | -1.78847 | 7.05E-60 |
| 27056 | S100a9 | -1.78623 | 1.24E-09 |
| 30894 | Tspear | -1.77879 | 0.00062 |
| 14561 | Gm5200 | -1.7781 | 7.27E-11 |
| 22623 | Olr1 | -1.77752 | 0.000951 |
| 7676 | Frzb | -1.77098 | 7.37E-54 |
| 29177 | Svopl | -1.7686 | 1.24E-06 |
| 7216 | Fam83b | -1.768 | 0.0005 |
| 27803 | Slc16a9 | -1.76423 | 7.74E-19 |
| 28944 | St6galnac2 | -1.7599 | 4.51E-31 |
| 30809 | Trp73 | -1.75832 | 6.35E-08 |
| 26704 | Rpl39l | -1.75038 | 0.000411 |
| 3871 | Ccdc190 | -1.75017 | 0.002099 |
| 27380 | Sema4a | -1.74894 | 5.75E-24 |
| 5396 | Cxxc4 | -1.747 | 1.34E-30 |
| 4882 | Cntn3 | -1.73786 | 7.87E-39 |
| 20003 | Mir212 | -1.7363 | 5.11E-05 |
| 4149 | Cda | -1.73494 | 4.84E-06 |
| 28200 | Smagp | -1.73447 | 9.28E-11 |
| 19181 | Ly6e | -1.72936 | 8.88E-48 |
| 30129 | Tmem54 | -1.7256 | 0.005804 |
| 1399 | A530021J07Rik | -1.72494 | 0.005601 |
| 26983 | Rtl3 | -1.72343 | 5.90E-51 |
| 18072 | Kcnab1 | -1.72291 | 4.66E-14 |
| 29729 | Tgtp1 | -1.71156 | 2.94E-09 |
| 18757 | Lhcgr | -1.7109 | 2.81E-06 |
| 25956 | Rarb | -1.70984 | 6.08E-33 |
| 4653 | Ckmt1 | -1.70865 | 8.29E-17 |
| 4399 | Cers3 | -1.70634 | 5.82E-06 |
| 28295 | Smpd3 | -1.70426 | 1.40E-47 |
| 19556 | Mdga2 | -1.69901 | 5.66E-19 |
| 17045 | Hpcal4 | -1.69852 | 9.48E-13 |
| 31364 | Usp43 | -1.69778 | 9.87E-11 |
| 19736 | Mgll | -1.69745 | 1.85E-44 |
| 29619 | Tes | -1.69726 | 1.22E-55 |
| 27951 | Slc30a2 | -1.69654 | 1.21E-05 |
| 30821 | Trpm1 | -1.69607 | 9.26E-09 |
| 28502 | Sntg2 | -1.69406 | 1.13E-14 |
| 24078 | Papln | -1.69279 | 3.47E-09 |
| 6003 | Dipk2b | -1.69183 | 0.000419 |
| 19104 | Lrrc8e | -1.69138 | 1.32E-08 |
| 6047 | Dlx4os | -1.69086 | 0.013114 |
| 2209 | Angpt2 | -1.69058 | 4.10E-35 |
| 7020 | F11r | -1.68988 | 5.77E-34 |
| 19573 | Mecom | -1.68866 | 3.10E-35 |
| 27829 | Slc22a1 | -1.68597 | 0.003773 |
| 26242 | Rgsl1 | -1.68191 | 0.000342 |
| 18749 | Lgi3 | -1.68179 | 1.67E-23 |
| 18759 | Lhfpl1 | -1.67812 | 4.05E-06 |
| 3341 | Brinp2 | -1.67436 | 1.20E-08 |
| 21974 | Nexmif | -1.66903 | 2.14E-29 |
| 7608 | Foxj1 | -1.66359 | 8.72E-15 |
| 2148 | Alx4 | -1.66319 | 6.35E-49 |
| 24735 | Plaat1 | -1.65922 | 2.27E-06 |
| 5071 | Cpm | -1.65797 | 6.81E-43 |
| 3176 | Bdkrb2 | -1.65676 | 7.68E-43 |
| 3923 | Ccdc81 | -1.65639 | 0.000109 |
| 26332 | Rimbp2 | -1.65348 | 5.98E-07 |
| 4583 | Chrnb4 | -1.6524 | 0.000822 |
| 25642 | Ptgs2 | -1.65054 | 5.63E-45 |
| 29951 | Tmem125 | -1.64554 | 0.006943 |
| 2423 | Apold1 | -1.64443 | 3.59E-42 |
| 18361 | Klk13 | -1.64402 | 0.009846 |
| 1356 | A230077H06Rik | -1.64147 | 1.57E-12 |
| 29709 | Tgfbi | -1.64096 | 9.59E-47 |
| 25697 | Ptprr | -1.64065 | 1.61E-19 |
| 13270 | Gm28286 | -1.6405 | 0.002794 |
| 6040 | Dll4 | -1.63785 | 1.11E-36 |
| 6748 | Enho | -1.63754 | 4.92E-24 |
| 17749 | Inhba | -1.63722 | 3.17E-49 |
| 27472 | Serpinb5 | -1.63542 | 1.22E-15 |
| 17907 | Itga11 | -1.63496 | 2.31E-44 |
| 27355 | Selenbp2 | -1.63175 | 1.18E-06 |
| 7245 | Fap | -1.63144 | 5.32E-36 |
| 3865 | Ccdc184 | -1.62892 | 0.000288 |
| 5647 | Dact1 | -1.62678 | 4.66E-46 |
| 21743 | Nap1l5 | -1.62649 | 1.07E-15 |
| 4747 | Clec4n | -1.62483 | 1.52E-20 |
| 29321 | Tafa2 | -1.62336 | 1.18E-23 |
| 25813 | Rab25 | -1.62202 | 4.38E-09 |
| 7058 | Fabp7 | -1.61951 | 4.22E-07 |
| 22295 | Nrg3 | -1.61572 | 2.03E-06 |
| 2146 | Alx1 | -1.61426 | 1.05E-16 |
| 24692 | Pkdcc | -1.61188 | 8.76E-45 |
| 21040 | Mmp13 | -1.60759 | 5.85E-45 |
| 6737 | En1 | -1.6075 | 4.12E-19 |
| 28079 | Slc5a7 | -1.60557 | 3.56E-23 |
| 3961 | Ccl21a | -1.60514 | 0.011482 |
| 4711 | Cldn5 | -1.60383 | 1.45E-29 |
| 14438 | Gm4951 | -1.60063 | 0.00022 |
| 32217 | Wnt11 | -1.59988 | 2.49E-36 |
| 526 | 2610528A11Rik | -1.59933 | 0.000959 |
| 24616 | Pik3c2g | -1.59582 | 8.30E-24 |
| 2332 | Aox3 | -1.59558 | 2.47E-28 |
| 7970 | Gdpd2 | -1.59445 | 1.13E-17 |
| 22439 | Nupr1 | -1.59384 | 6.16E-34 |
| 27068 | Saa3 | -1.59322 | 9.06E-11 |
| 27979 | Slc35d3 | -1.59232 | 7.45E-15 |
| 32231 | Wnt9a | -1.59101 | 1.11E-44 |
| 26984 | Rtl4 | -1.58911 | 1.13E-07 |
| 28100 | Slc6a4 | -1.58797 | 1.10E-07 |
| 6785 | Eomes | -1.58304 | 0.0012 |
| 28242 | Smim1 | -1.58239 | 3.96E-23 |
| 7848 | Galntl6 | -1.58166 | 0.007377 |
| 2767 | Atp10b | -1.58124 | 0.004649 |
| 21601 | Myrip | -1.57982 | 1.33E-10 |
| 16175 | Golga7b | -1.57424 | 0.0101 |
| 16737 | Has1 | -1.57376 | 8.06E-23 |
| 4844 | Cnksr1 | -1.57351 | 4.25E-06 |
| 26086 | Rcan2 | -1.57106 | 1.32E-30 |
| 5054 | Cpa6 | -1.57006 | 3.71E-17 |
| 29077 | Stx19 | -1.56621 | 0.00779 |
| 24053 | Pak6 | -1.56494 | 1.17E-11 |
| 4594 | Chst2 | -1.56313 | 3.43E-45 |
| 21957 | Neto2 | -1.56157 | 1.72E-41 |
| 2401 | Apoc1 | -1.55701 | 1.45E-07 |
| 3173 | Bdh1 | -1.55563 | 3.48E-30 |
| 1748 | Adam12 | -1.55231 | 4.80E-45 |
| 22233 | Npr1 | -1.54311 | 4.94E-17 |
| 32378 | Zbtb16 | -1.54262 | 6.84E-12 |
| 17930 | Itgb4 | -1.5408 | 2.71E-21 |
| 24066 | Pamr1 | -1.53977 | 6.39E-29 |
| 22625 | Omd | -1.5366 | 3.26E-38 |
| 27860 | Slc24a2 | -1.53592 | 3.04E-07 |
| 5078 | Cpne5 | -1.53591 | 8.81E-32 |
| 17671 | Il1rl1 | -1.53471 | 5.61E-24 |
| 1845 | Adgrf4 | -1.52837 | 0.006031 |
| 3690 | Car2 | -1.52598 | 1.53E-29 |
| 5993 | Dio2 | -1.52547 | 1.32E-29 |
| 4120 | Cd55 | -1.52034 | 5.01E-17 |
| 6819 | Ephx2 | -1.51715 | 4.49E-12 |
| 4208 | Cdh23 | -1.51504 | 1.05E-14 |
| 21033 | Mme | -1.51222 | 3.36E-40 |
| 18146 | Kcnn4 | -1.51185 | 6.77E-11 |
| 27354 | Selenbp1 | -1.5109 | 3.30E-17 |
| 22028 | Nhs | -1.51026 | 2.85E-30 |
| 5625 | D7Ertd443e | -1.50774 | 0.001376 |
| 32002 | Vnn1 | -1.50655 | 2.91E-11 |
| 29625 | Tesl1 | -1.50599 | 3.61E-13 |
| 4281 | Cdsn | -1.5054 | 1.38E-06 |
| 19634 | Meox2 | -1.50348 | 3.31E-14 |
| 2142 | Alpl | -1.50021 | 2.60E-39 |
| 24186 | Pcdh20 | -1.49981 | 3.70E-24 |
| 25976 | Rasgrp1 | -1.49833 | 5.01E-06 |
| 5503 | Cyp2s1 | -1.49664 | 2.29E-19 |
| 16740 | Has3 | -1.49623 | 7.91E-24 |
| 32220 | Wnt2b | -1.49561 | 4.77E-19 |
| 18239 | Kif19b | -1.49532 | 0.000843 |
| 28026 | Slc39a8 | -1.49529 | 3.83E-19 |
| 17239 | Ifi202b | -1.49345 | 4.69E-21 |
| 22283 | Nrarp | -1.49269 | 2.34E-08 |
| 28550 | Sod3 | -1.49267 | 9.09E-15 |
| 6525 | Efcab1 | -1.491 | 2.49E-08 |
| 27985 | Slc35f2 | -1.49047 | 2.32E-22 |
| 22298 | Nrgn | -1.48882 | 1.12E-22 |
| 28607 | Sp7 | -1.48831 | 8.16E-38 |
| 25466 | Prss12 | -1.48557 | 5.72E-41 |
| 7963 | Gdf6 | -1.48457 | 6.21E-22 |
| 17906 | Itga10 | -1.4808 | 1.28E-33 |
| 5296 | Ctla2a | -1.48007 | 2.19E-12 |
| 7543 | Fmn2 | -1.47952 | 3.22E-23 |
| 6812 | Epha8 | -1.4786 | 7.22E-07 |
| 27055 | S100a8 | -1.4782 | 0.000154 |
| 7468 | Fhdc1 | -1.47816 | 9.13E-17 |
| 27165 | Scara3 | -1.47569 | 3.12E-38 |
| 7221 | Fam83g | -1.47417 | 3.25E-10 |
| 6062 | Dmp1 | -1.47272 | 6.78E-36 |
| 29720 | Tgm1 | -1.47208 | 5.07E-06 |
| 30666 | Trf | -1.46917 | 2.86E-23 |
| 29166 | Sv2b | -1.46816 | 4.23E-08 |
| 27392 | Sema7a | -1.46634 | 4.24E-36 |
| 17811 | Ipcef1 | -1.46625 | 0.003215 |
| 30379 | Trabd2b | -1.46508 | 4.20E-34 |
| 28594 | Sox7 | -1.46458 | 6.69E-24 |
| 4795 | Clu | -1.46397 | 6.37E-34 |
| 6331 | Dusp2 | -1.46356 | 1.59E-24 |
| 22018 | Ngp | -1.4634 | 0.00016 |
| 6015 | Dkk1 | -1.46049 | 6.98E-20 |
| 29721 | Tgm2 | -1.4582 | 5.19E-29 |
| 6530 | Efcab15 | -1.45076 | 0.000405 |
| 529 | 2700046A07Rik | -1.44914 | 0.010236 |
| 28754 | Spns2 | -1.44891 | 4.72E-26 |
| 1790 | Adamts19 | -1.4475 | 1.41E-22 |
| 7962 | Gdf5 | -1.44674 | 5.29E-05 |
| 19121 | Lrrtm2 | -1.44552 | 3.33E-22 |
| 7031 | F5 | -1.44383 | 0.001297 |
| 3159 | Bcl6b | -1.44177 | 4.76E-27 |
| 6750 | Enkur | -1.44086 | 3.92E-05 |
| 6188 | Doc2b | -1.44025 | 5.32E-33 |
| 21020 | Mlph | -1.4315 | 1.33E-08 |
| 17750 | Inhbb | -1.43069 | 9.51E-19 |
| 27088 | Samd15 | -1.42785 | 1.76E-05 |
| 17207 | Icam2 | -1.42503 | 1.04E-20 |
| 18169 | Kctd14 | -1.42099 | 6.73E-11 |
| 24724 | Pla2g4a | -1.41702 | 7.30E-31 |
| 2328 | Aoc3 | -1.41654 | 2.48E-19 |
| 25381 | Procr | -1.41597 | 2.00E-14 |
| 8110 | Glp1r | -1.41259 | 0.000679 |
| 19253 | Macc1 | -1.4123 | 0.000282 |
| 2268 | Ankrd45 | -1.40946 | 1.74E-08 |
| 29708 | Tgfb3 | -1.40925 | 1.54E-36 |
| 4578 | Chrna7 | -1.40777 | 1.27E-08 |
| 19249 | Mab21l1 | -1.40075 | 7.82E-30 |
| 3874 | Ccdc194 | -1.40071 | 4.62E-09 |
| 4307 | Cebpd | -1.39901 | 1.40E-37 |
| 7027 | F2rl2 | -1.39672 | 0.000455 |
| 4598 | Chst7 | -1.396 | 7.21E-10 |
| 4211 | Cdh3 | -1.39551 | 1.05E-23 |
| 6321 | Dusp1 | -1.39489 | 1.06E-37 |
| 17663 | Il1b | -1.39308 | 0.009178 |
| 17079 | Hs3st4 | -1.3917 | 9.74E-05 |
| 6715 | Emcn | -1.39035 | 2.73E-26 |
| 18287 | Kl | -1.39024 | 9.91E-05 |
| 30682 | Trh | -1.38984 | 8.56E-05 |
| 21138 | Mpped1 | -1.3885 | 3.28E-15 |
| 29531 | Tcp11 | -1.38818 | 1.10E-10 |
| 4486 | Cgref1 | -1.38736 | 1.62E-19 |
| 6771 | Entpd3 | -1.38375 | 3.77E-32 |
| 27251 | Scn3a | -1.38368 | 2.69E-17 |
| 2782 | Atp1b1 | -1.38353 | 4.38E-31 |
| 6541 | Efhb | -1.38344 | 0.000116 |
| 22362 | Ntn5 | -1.38322 | 7.52E-12 |
| 24743 | Plagl1 | -1.38296 | 2.17E-41 |
| 5387 | Cxcl5 | -1.3789 | 1.42E-32 |
| 22190 | Notch4 | -1.37816 | 4.07E-31 |
| 7923 | Gbp9 | -1.37539 | 3.79E-21 |
| 16256 | Gpr141 | -1.37473 | 0.003946 |
| 4213 | Cdh5 | -1.37416 | 1.13E-36 |
| 5433 | Cyp1b1 | -1.3734 | 8.85E-31 |
| 23992 | P2rx6 | -1.37054 | 1.62E-08 |
| 17935 | Itgbl1 | -1.36882 | 1.41E-33 |
| 5994 | Dio3 | -1.36843 | 3.59E-22 |
| 29509 | Tcf7 | -1.36779 | 2.90E-30 |
| 3752 | Catspere2 | -1.36758 | 0.002426 |
| 1892 | Adra2c | -1.36674 | 0.005867 |
| 29031 | Stk38l | -1.36565 | 1.73E-26 |
| 3262 | Bmp5 | -1.36533 | 1.12E-18 |
| 3954 | Ccl12 | -1.36359 | 0.00577 |
| 18628 | Large2 | -1.36153 | 3.89E-18 |
| 22277 | Nr4a3 | -1.35883 | 1.35E-35 |
| 28624 | Spag16 | -1.35794 | 0.005657 |
| 5995 | Dio3os | -1.35281 | 3.57E-22 |
| 27364 | Selenop | -1.35121 | 5.55E-34 |
| 7522 | Fli1 | -1.35087 | 6.47E-17 |
| 26226 | Rgs17 | -1.34839 | 2.72E-21 |
| 6280 | Dsc3 | -1.34503 | 1.51E-25 |
| 19442 | Marco | -1.34102 | 1.61E-10 |
| 17867 | Irf8 | -1.33912 | 3.62E-23 |
| 25950 | Rapgef5 | -1.33903 | 1.08E-21 |
| 6104 | Dnah7b | -1.33801 | 2.36E-08 |
| 29605 | Tent5a | -1.33617 | 6.99E-30 |
| 396 | 1810019D21Rik | -1.32794 | 0.00214 |
| 27820 | Slc1a1 | -1.32261 | 0.000905 |
| 8061 | Gjb2 | -1.31969 | 6.58E-07 |
| 18775 | Lif | -1.31858 | 1.44E-16 |
| 6780 | Entrep2 | -1.31658 | 9.03E-30 |
| 6811 | Epha7 | -1.31626 | 3.52E-30 |
| 3596 | Cadm1 | -1.31415 | 9.91E-39 |
| 3253 | Bmf | -1.31259 | 1.91E-32 |
| 29789 | Tie1 | -1.31017 | 5.85E-28 |
| 27042 | S100a1 | -1.3096 | 7.23E-19 |
| 29323 | Tafa4 | -1.30832 | 0.001591 |
| 16048 | Gm9661 | -1.30729 | 0.012085 |
| 16368 | Gria1 | -1.30708 | 3.63E-35 |
| 4457 | Cfap70 | -1.30667 | 0.000656 |
| 7490 | Fignl2 | -1.30432 | 2.25E-15 |
| 3122 | Bcan | -1.30374 | 1.70E-06 |
| 32200 | Wif1 | -1.30086 | 2.99E-28 |
| 2322 | Aoah | -1.30055 | 0.000122 |
| 7327 | Fbxo40 | -1.29889 | 0.000979 |
| 18120 | Kcnj8 | -1.29863 | 5.21E-20 |
| 24398 | Pecam1 | -1.29624 | 6.12E-31 |
| 16939 | Hmgcll1 | -1.29532 | 1.73E-09 |
| 24083 | Pappa2 | -1.29449 | 2.69E-33 |
| 18579 | Kynu | -1.29314 | 0.004762 |
| 7836 | Galnt15 | -1.2928 | 0.00182 |
| 17067 | Hrh2 | -1.29267 | 0.013114 |
| 28129 | Slc9a3r1 | -1.2925 | 8.16E-20 |
| 26518 | Robo3 | -1.29246 | 2.51E-06 |
| 8083 | Glb1l2 | -1.29187 | 1.90E-08 |
| 4485 | Cgnl1 | -1.2918 | 2.43E-28 |
| 27622 | Sh3tc2 | -1.29003 | 7.12E-06 |
| 21908 | Nebl | -1.28853 | 7.15E-15 |
| 29773 | Thsd4 | -1.28826 | 2.99E-31 |
| 16707 | Haao | -1.28707 | 0.002933 |
| 7281 | Fbp1 | -1.2863 | 3.80E-05 |
| 24857 | Plppr4 | -1.28389 | 6.09E-13 |
| 28762 | Spon2 | -1.28233 | 1.08E-07 |
| 24090 | Paqr7 | -1.28231 | 2.15E-18 |
| 30879 | Tspan15 | -1.28101 | 1.50E-19 |
| 5615 | D630033O11Rik | -1.2802 | 0.000157 |
| 16066 | Gm973 | -1.27889 | 0.012334 |
| 3276 | Bnc2 | -1.27706 | 7.54E-26 |
| 24441 | Pf4 | -1.27682 | 1.27E-07 |
| 19051 | Lrrc23 | -1.27636 | 3.90E-05 |
| 18281 | Kirrel3 | -1.27594 | 3.93E-16 |
| 16088 | Gm9899 | -1.27576 | 1.75E-08 |
| 24487 | Pgm5 | -1.27569 | 6.94E-31 |
| 26460 | Rnf207 | -1.27456 | 0.001438 |
| 17061 | Hr | -1.27356 | 2.36E-24 |
| 5168 | Crtac1 | -1.26931 | 5.01E-14 |
| 28030 | Slc40a1 | -1.26926 | 9.11E-23 |
| 7792 | Gabra4 | -1.26711 | 6.33E-20 |
| 7914 | Gbp11 | -1.26657 | 1.03E-05 |
| 19160 | Ltf | -1.2665 | 0.001423 |
| 8037 | Gimap8 | -1.26618 | 4.93E-07 |
| 29705 | Tgfb1 | -1.2659 | 9.24E-28 |
| 27762 | Slc10a4-ps | -1.26414 | 0.008111 |
| 32360 | Zar1 | -1.26322 | 0.002181 |
| 26236 | Rgs6 | -1.2629 | 4.67E-12 |
| 17644 | Il15 | -1.26259 | 0.000297 |
| 4330 | Cend1 | -1.26175 | 4.05E-10 |
| 17236 | Ier5l | -1.26137 | 1.87E-27 |
| 7435 | Fgf14 | -1.26075 | 2.15E-11 |
| 28940 | St3gal6 | -1.26059 | 1.61E-18 |
| 32213 | Wnk4 | -1.26023 | 7.03E-20 |
| 17894 | Ism2 | -1.25957 | 4.19E-07 |
| 4719 | Clec11a | -1.2595 | 9.72E-26 |
| 2474 | Arg2 | -1.25649 | 0.000424 |
| 18499 | Krt79 | -1.25608 | 1.77E-06 |
| 4480 | Cftr | -1.25454 | 0.010525 |
| 17092 | Hsd17b11 | -1.25419 | 5.59E-24 |
| 17128 | Hspa1b | -1.25417 | 1.86E-23 |
| 2480 | Arhgap15 | -1.25413 | 0.00108 |
| 21963 | Neurl1b | -1.25171 | 5.31E-12 |
| 21420 | Mtus1 | -1.25107 | 1.20E-23 |
| 28887 | Srxn1 | -1.25067 | 6.61E-27 |
| 19504 | Mc5r | -1.24863 | 1.31E-12 |
| 8056 | Gja4 | -1.24715 | 1.03E-18 |
| 29679 | Tfap2c | -1.24641 | 1.16E-24 |
| 8035 | Gimap6 | -1.24545 | 4.22E-19 |
| 30264 | Tnmd | -1.24504 | 2.94E-22 |
| 26210 | Rgcc | -1.24436 | 7.01E-24 |
| 17241 | Ifi204 | -1.24406 | 4.95E-06 |
| 27474 | Serpinb6b | -1.24286 | 1.96E-10 |
| 4099 | Cd302 | -1.24264 | 5.54E-24 |
| 17617 | Igtp | -1.23879 | 9.30E-15 |
| 1652 | Acod1 | -1.23754 | 8.46E-05 |
| 16292 | Gpr3 | -1.23672 | 1.27E-10 |
| 17321 | Igf1 | -1.23644 | 8.05E-28 |
| 2072 | Alas2 | -1.23631 | 0.002407 |
| 31323 | Ushbp1 | -1.23256 | 2.72E-21 |
| 32495 | Zfhx4 | -1.22964 | 1.74E-27 |
| 30877 | Tspan13 | -1.22905 | 1.49E-24 |
| 30335 | Tpbgl | -1.22706 | 0.001015 |
| 8095 | Glipr1 | -1.22646 | 3.82E-17 |
| 25548 | Psma8 | -1.22547 | 0.010164 |
| 6313 | Duox1 | -1.22486 | 4.00E-07 |
| 19099 | Lrrc75b | -1.22383 | 2.19E-13 |
| 24181 | Pcdh12 | -1.2238 | 1.29E-16 |
| 5560 | Cysltr1 | -1.22357 | 2.71E-05 |
| 28582 | Sox18 | -1.21968 | 2.43E-14 |
| 6036 | Dlk1 | -1.21798 | 1.99E-25 |
| 32268 | Xkrx | -1.21696 | 0.008726 |
| 6867 | Erg | -1.21552 | 1.13E-17 |
| 18144 | Kcnn2 | -1.21368 | 7.37E-08 |
| 1879 | Adora2a | -1.21364 | 2.28E-08 |
| 5247 | Csrp2 | -1.21179 | 1.15E-26 |
| 28972 | Stap2 | -1.21084 | 4.39E-10 |
| 5603 | D430019H16Rik | -1.2097 | 2.23E-26 |
| 22184 | Nos3 | -1.20868 | 3.18E-07 |
| 26420 | Rnf125 | -1.20833 | 5.51E-06 |
| 7188 | Fam241a | -1.20706 | 1.96E-12 |
| 19176 | Ly6a | -1.20687 | 0.000809 |
| 24736 | Plaat3 | -1.20639 | 6.48E-07 |
| 25504 | Prss8 | -1.20638 | 0.000379 |
| 25648 | Pth2r | -1.20626 | 0.010758 |
| 25959 | Rarres2 | -1.20604 | 6.97E-07 |
| 7919 | Gbp5 | -1.20603 | 6.76E-07 |
| 5186 | Crybg1 | -1.20564 | 3.41E-08 |
| 17141 | Hspb7 | -1.20514 | 7.44E-09 |
| 4446 | Cfap45 | -1.20307 | 0.000185 |
| 25975 | Rasgrf2 | -1.2028 | 2.69E-06 |
| 2526 | Arhgef19 | -1.20233 | 1.71E-16 |
| 28336 | Sned1 | -1.20173 | 1.85E-28 |
| 16448 | Gsta3 | -1.20096 | 0.002719 |
| 31291 | Upp1 | -1.19936 | 3.57E-09 |
| 17863 | Irf4 | -1.19749 | 0.000524 |
| 26428 | Rnf138rt1 | -1.19678 | 0.008819 |
| 5388 | Cxcl9 | -1.19616 | 0.010502 |
| 3043 | Batf3 | -1.19606 | 7.43E-13 |
| 1956 | Agt | -1.19548 | 1.10E-15 |
| 26340 | Rims4 | -1.1951 | 0.000251 |
| 3624 | Calml4 | -1.19459 | 1.84E-14 |
| 19738 | Mgmt | -1.19258 | 2.41E-06 |
| 3709 | Carmil1 | -1.19107 | 2.79E-25 |
| 3493 | C1s1 | -1.1898 | 9.94E-12 |
| 17333 | Igfbp4 | -1.18965 | 1.26E-24 |
| 3801 | Ccbe1 | -1.18951 | 4.36E-21 |
| 19380 | Map3k21 | -1.18943 | 0.002913 |
| 28083 | Slc66a2 | -1.18867 | 7.58E-20 |
| 5110 | Cracr2a | -1.1866 | 1.73E-13 |
| 3283 | Bnipl | -1.18629 | 0.00104 |
| 2456 | Areg | -1.18526 | 0.001388 |
| 3570 | Cacna1e | -1.18459 | 2.82E-11 |
| 18570 | Krtdap | -1.18446 | 0.000689 |
| 24333 | Pde8a | -1.18417 | 4.86E-22 |
| 32196 | Wfikkn2 | -1.18305 | 6.56E-23 |
| 30381 | Traf1 | -1.18293 | 0.000604 |
| 17247 | Ifi211 | -1.18236 | 0.001565 |
| 27523 | Sez6 | -1.1782 | 0.000376 |
| 17080 | Hs3st5 | -1.17778 | 1.54E-08 |
| 27078 | Sall1 | -1.17608 | 4.10E-05 |
| 29982 | Tmem151a | -1.176 | 1.08E-09 |
| 4105 | Cd38 | -1.17541 | 3.97E-16 |
| 27017 | Runx2 | -1.17319 | 6.20E-28 |
| 25101 | Ppm1j | -1.17271 | 1.63E-10 |
| 17984 | Jakmip2 | -1.17244 | 9.41E-07 |
| 29702 | Tg | -1.17105 | 0.000296 |
| 1857 | Adgrl4 | -1.1701 | 4.31E-10 |
| 29329 | Tagln2 | -1.16984 | 1.29E-24 |
| 5431 | Cyp1a1 | -1.16785 | 4.61E-15 |
| 32608 | Zfp385c | -1.16742 | 2.16E-06 |
| 17127 | Hspa1a | -1.16624 | 1.54E-21 |
| 24880 | Plxnc1 | -1.16615 | 1.46E-19 |
| 26171 | Retreg1 | -1.16568 | 1.34E-17 |
| 19328 | Mamdc2 | -1.16523 | 5.40E-18 |
| 6899 | Esam | -1.16386 | 5.96E-17 |
| 27379 | Sema3g | -1.16344 | 8.94E-15 |
| 24166 | Pbx3 | -1.16164 | 1.32E-20 |
| 7975 | Gem | -1.16154 | 3.59E-23 |
| 5943 | Dgki | -1.16124 | 4.62E-05 |
| 24761 | Plaur | -1.16076 | 5.91E-17 |
| 29984 | Tmem154 | -1.16006 | 7.85E-05 |
| 24803 | Plekhd1 | -1.15991 | 0.000378 |
| 21945 | Nell1 | -1.15971 | 2.09E-17 |
| 19151 | Ltb | -1.15759 | 0.010403 |
| 18607 | Lamc3 | -1.15588 | 6.05E-20 |
| 7176 | Fam222a | -1.1547 | 1.15E-14 |
| 27796 | Slc16a2 | -1.1537 | 7.44E-26 |
| 25330 | Prkg2 | -1.14997 | 2.74E-14 |
| 19197 | Ly75 | -1.14991 | 2.58E-14 |
| 29252 | Sytl1 | -1.14785 | 4.92E-07 |
| 7644 | Fpr1 | -1.14385 | 0.012772 |
| 27052 | S100a6 | -1.14379 | 9.07E-21 |
| 7584 | Fosb | -1.14228 | 1.22E-25 |
| 3224 | Bik | -1.14197 | 0.001232 |
| 29691 | Tfec | -1.14173 | 3.14E-06 |
| 3779 | Cbr3 | -1.14158 | 3.00E-15 |
| 18693 | Lcp2 | -1.13987 | 2.20E-12 |
| 26158 | Reps2 | -1.13758 | 5.42E-18 |
| 16466 | Gstt2 | -1.13589 | 1.50E-05 |
| 21769 | Nat8l | -1.13345 | 7.20E-12 |
| 2083 | Aldh1l1 | -1.13307 | 2.06E-07 |
| 25447 | Prr5l | -1.13252 | 1.04E-15 |
| 32199 | Whrn | -1.13204 | 1.05E-23 |
| 29770 | Thrb | -1.1315 | 2.38E-10 |
| 7947 | Gcnt4 | -1.13098 | 3.69E-10 |
| 28086 | Slc6a12 | -1.1299 | 7.49E-06 |
| 19664 | Mettl24 | -1.12936 | 0.003664 |
| 25803 | Rab17 | -1.12847 | 0.007624 |
| 2493 | Arhgap27 | -1.12844 | 1.20E-14 |
| 5163 | Crocc2 | -1.12759 | 0.000251 |
| 7536 | Flt4 | -1.12678 | 4.80E-17 |
| 21139 | Mpped2 | -1.12663 | 7.69E-26 |
| 17168 | Htr7 | -1.12656 | 8.56E-11 |
| 21061 | Mmrn2 | -1.12478 | 4.05E-13 |
| 3984 | Ccn5 | -1.12429 | 7.92E-08 |
| 18748 | Lgi2 | -1.12385 | 9.47E-23 |
| 4306 | Cebpb | -1.12268 | 1.45E-15 |
| 4749 | Clec7a | -1.12267 | 1.36E-07 |
| 3824 | Ccdc13 | -1.12262 | 0.001088 |
| 28761 | Spon1 | -1.12242 | 2.09E-24 |
| 31444 | Vdr | -1.12196 | 7.10E-14 |
| 18758 | Lhfp | -1.11942 | 7.12E-24 |
| 8219 | Gm10382 | -1.11862 | 2.03E-06 |
| 30687 | Trib1 | -1.11797 | 1.33E-25 |
| 28149 | Slco4a1 | -1.1154 | 2.69E-12 |
| 5323 | Ctsc | -1.1134 | 3.37E-21 |
| 22363 | Ntng1 | -1.11198 | 5.52E-18 |
| 31022 | Tulp1 | -1.11173 | 2.47E-07 |
| 4075 | Cd22 | -1.11134 | 0.001723 |
| 16323 | Gprc5a | -1.11117 | 4.74E-12 |
| 6807 | Epha3 | -1.11089 | 4.40E-21 |
| 30165 | Tmem95 | -1.11063 | 2.50E-07 |
| 13561 | Gm32391 | -1.10774 | 0.004704 |
| 16363 | Grem2 | -1.10769 | 9.13E-20 |
| 22480 | Oas1g | -1.10741 | 0.001922 |
| 3144 | Bcl2 | -1.10666 | 1.36E-21 |
| 1874 | Adm2 | -1.10421 | 0.000395 |
| 3778 | Cbr2 | -1.10321 | 5.77E-05 |
| 31436 | Vcan | -1.10132 | 1.14E-23 |
| 23919 | Osbpl3 | -1.10035 | 4.24E-17 |
| 18741 | Lgals3bp | -1.10026 | 1.18E-18 |
| 17216 | Id2 | -1.09966 | 4.36E-24 |
| 29106 | Sugct | -1.09954 | 1.66E-05 |
| 18113 | Kcnj15 | -1.09635 | 0.001372 |
| 29859 | Tlnrd1 | -1.09597 | 3.10E-24 |
| 5377 | Cxcl10 | -1.0954 | 4.59E-10 |
| 22574 | Ogn | -1.09428 | 7.80E-24 |
| 26201 | Rfx2 | -1.09413 | 5.48E-17 |
| 21059 | Mmp9 | -1.09316 | 2.21E-20 |
| 30221 | Tnfaip8l3 | -1.09142 | 8.03E-14 |
| 27169 | Scarf1 | -1.09015 | 3.96E-11 |
| 30958 | Ttc7 | -1.08906 | 7.64E-08 |
| 6539 | Efemp1 | -1.08849 | 9.30E-20 |
| 5243 | Csrnp1 | -1.08682 | 2.85E-22 |
| 24870 | Plxdc1 | -1.08417 | 1.15E-09 |
| 28897 | Ssc4d | -1.08357 | 4.77E-10 |
| 4722 | Clec14a | -1.08307 | 6.46E-19 |
| 6344 | Dusp8 | -1.08302 | 1.51E-17 |
| 7749 | Fzd1 | -1.08266 | 1.53E-24 |
| 6905 | Esm1 | -1.08264 | 1.04E-12 |
| 27868 | Slc25a13 | -1.08152 | 6.61E-13 |
| 3729 | Casp4 | -1.08123 | 3.06E-05 |
| 21846 | Ndrg1 | -1.08022 | 2.01E-18 |
| 25389 | Prom1 | -1.07974 | 3.56E-19 |
| 25373 | Prmt8 | -1.07967 | 0.005992 |
| 27676 | Sigirr | -1.07909 | 0.000711 |
| 515 | 2610035D17Rik | -1.07861 | 1.85E-06 |
| 3229 | Birc3 | -1.07732 | 1.42E-19 |
| 28745 | Spint2 | -1.07386 | 1.39E-09 |
| 30750 | Trim67 | -1.0732 | 0.010495 |
| 32216 | Wnt10b | -1.07127 | 2.17E-15 |
| 18069 | Kcna5 | -1.06968 | 2.41E-08 |
| 25692 | Ptprm | -1.06539 | 1.74E-21 |
| 18130 | Kcnk3 | -1.06435 | 0.000179 |
| 28959 | Stab2 | -1.06431 | 0.00019 |
| 27903 | Slc25a45 | -1.06355 | 7.15E-10 |
| 24732 | Pla2g7 | -1.06313 | 3.85E-18 |
| 1783 | Adamts12 | -1.06307 | 2.53E-22 |
| 25076 | Ppfibp2 | -1.06224 | 1.67E-17 |
| 28007 | Slc38a3 | -1.06178 | 9.62E-09 |
| 5804 | Decr1 | -1.0617 | 8.54E-17 |
| 416 | 1810073O08Rik | -1.06112 | 0.011539 |
| 3977 | Ccl9 | -1.0604 | 2.89E-15 |
| 29231 | Syp | -1.05971 | 0.000347 |
| 4955 | Col9a1 | -1.05954 | 9.14E-06 |
| 27797 | Slc16a3 | -1.05946 | 1.78E-11 |
| 6693 | Elovl2 | -1.0592 | 8.42E-08 |
| 16733 | Hapln4 | -1.05762 | 0.001914 |
| 6579 | Ehf | -1.05739 | 0.011172 |
| 16828 | Hecw2 | -1.05732 | 4.68E-16 |
| 22154 | Nod2 | -1.05694 | 1.83E-09 |
| 22364 | Ntng2 | -1.05679 | 5.94E-07 |
| 25140 | Ppp1r36 | -1.056 | 0.014372 |
| 30217 | Tnfaip6 | -1.05453 | 4.51E-21 |
| 32056 | Vstm2b | -1.054 | 2.55E-18 |
| 22102 | Nlrp1b | -1.05328 | 0.009292 |
| 6971 | Evpl | -1.05295 | 1.25E-12 |
| 22584 | Olfm4 | -1.05267 | 0.009971 |
| 21731 | Nalf2 | -1.04969 | 2.04E-07 |
| 21425 | Muc1 | -1.04829 | 2.56E-12 |
| 18333 | Klhl23 | -1.04822 | 2.41E-20 |
| 3188 | Best1 | -1.04703 | 0.000313 |
| 2569 | Arl4c | -1.04553 | 1.40E-17 |
| 7372 | Fcgr3 | -1.0454 | 2.27E-12 |
| 3174 | Bdh2 | -1.04392 | 4.21E-12 |
| 5718 | Dclre1c | -1.0436 | 2.42E-16 |
| 31435 | Vcam1 | -1.03989 | 1.79E-19 |
| 19615 | Megf11 | -1.03973 | 4.20E-15 |
| 29111 | Sulf2 | -1.03935 | 9.59E-20 |
| 3143 | Bcl11b | -1.03786 | 4.83E-18 |
| 6326 | Dusp14 | -1.03541 | 1.50E-17 |
| 6984 | Exoc3l | -1.03359 | 1.93E-12 |
| 17233 | Ier3 | -1.0332 | 1.76E-17 |
| 6806 | Epha2 | -1.03295 | 1.25E-13 |
| 4146 | Cd93 | -1.03226 | 3.72E-19 |
| 24178 | Pcdh1 | -1.03221 | 7.38E-16 |
| 7886 | Gas6 | -1.03045 | 1.36E-17 |
| 25291 | Prg4 | -1.02978 | 0.000535 |
| 7235 | Fancc | -1.02935 | 1.31E-14 |
| 1698 | Acss3 | -1.02904 | 1.41E-09 |
| 28960 | Stac | -1.02777 | 5.26E-10 |
| 18163 | Kctd1 | -1.0255 | 9.49E-20 |
| 4956 | Col9a2 | -1.02535 | 1.19E-15 |
| 32053 | Vsir | -1.02341 | 2.77E-10 |
| 29817 | Timp1 | -1.02125 | 5.90E-19 |
| 28299 | Smpdl3b | -1.02105 | 1.86E-10 |
| 16436 | Gsg1l | -1.01924 | 1.15E-11 |
| 1561 | Abcg5 | -1.01916 | 0.001048 |
| 6897 | Errfi1 | -1.01788 | 2.91E-21 |
| 19696 | Mfng | -1.01771 | 2.01E-10 |
| 32593 | Zfp36 | -1.01767 | 6.49E-20 |
| 4587 | Chst1 | -1.01556 | 1.00E-20 |
| 3220 | Bicdl2 | -1.01482 | 0.008978 |
| 25755 | Pygl | -1.0147 | 6.66E-12 |
| 27238 | Scin | -1.01422 | 1.51E-16 |
| 6502 | Ednrb | -1.00962 | 4.23E-11 |
| 16122 | Gna15 | -1.00916 | 0.000355 |
| 2643 | As3mt | -1.00768 | 1.43E-08 |
| 5557 | Cyria | -1.00523 | 1.43E-13 |
| 7113 | Fam161a | -1.00507 | 2.48E-06 |
| 18739 | Lgals2 | -1.00464 | 0.009874 |
| 21131 | Mpo | -1.00294 | 3.09E-05 |
| 4491 | Chad | -1.00208 | 6.05E-05 |
| 18016 | Jun | -1.00012 | 2.44E-18 |
| 17089 | Hsd11b2 | -0.99824 | 0.001684 |
| 1634 | Ace | -0.99753 | 3.75E-06 |
| 32187 | Wfdc2 | -0.99517 | 0.002661 |
| 24260 | Pclo | -0.99487 | 0.002207 |
| 25890 | Rad51b | -0.99428 | 8.52E-08 |
| 19207 | Lypd1 | -0.99425 | 0.000159 |
| 18166 | Kctd12 | -0.99285 | 9.81E-19 |
| 4145 | Cd9 | -0.99267 | 5.62E-16 |
| 5328 | Ctsh | -0.99265 | 1.27E-11 |
| 28759 | Spock2 | -0.99208 | 1.09E-19 |
| 21515 | Myct1 | -0.99186 | 2.43E-09 |
| 13991 | Gm39529 | -0.9915 | 5.15E-10 |
| 28864 | Srpx2 | -0.98999 | 5.52E-15 |
| 18051 | Kazald1 | -0.9897 | 3.72E-15 |
| 19068 | Lrrc4 | -0.98948 | 1.60E-09 |
| 22031 | Niban1 | -0.98902 | 3.40E-18 |
| 30212 | Tnc | -0.98811 | 1.92E-18 |
| 19234 | Lyz1 | -0.98799 | 4.05E-11 |
| 21843 | Ndnf | -0.98749 | 1.70E-18 |
| 7586 | Fosl2 | -0.98646 | 3.39E-19 |
| 29533 | Tcp11l2 | -0.98569 | 1.13E-13 |
| 3148 | Bcl2a1d | -0.98551 | 0.010733 |
| 25293 | Prickle1 | -0.98503 | 1.03E-18 |
| 21058 | Mmp8 | -0.98484 | 0.000374 |
| 7755 | Fzd5 | -0.98464 | 3.62E-12 |
| 17618 | Ihh | -0.98457 | 0.000966 |
| 16730 | Hapln1 | -0.98443 | 2.61E-13 |
| 21004 | Mkx | -0.98261 | 3.27E-12 |
| 21568 | Myo1d | -0.98206 | 6.17E-15 |
| 16525 | Gulp1 | -0.97962 | 6.95E-18 |
| 6329 | Dusp18 | -0.97945 | 2.22E-18 |
| 1785 | Adamts14 | -0.97876 | 1.91E-18 |
| 30891 | Tspan7 | -0.97858 | 1.86E-18 |
| 30290 | Tnxb | -0.97712 | 1.58E-07 |
| 4079 | Cd248 | -0.97657 | 4.68E-17 |
| 2085 | Aldh2 | -0.97643 | 1.87E-16 |
| 2491 | Arhgap25 | -0.97535 | 3.29E-08 |
| 21039 | Mmp12 | -0.97473 | 3.44E-12 |
| 27491 | Serpine2 | -0.97444 | 1.54E-18 |
| 28585 | Sox21 | -0.97339 | 0.002387 |
| 26321 | Ric3 | -0.97236 | 2.52E-09 |
| 4825 | Cmya5 | -0.97222 | 6.17E-10 |
| 8132 | Glul | -0.9708 | 1.41E-19 |
| 1846 | Adgrf5 | -0.97035 | 2.77E-15 |
| 25691 | Ptprk | -0.96928 | 1.46E-16 |
| 18981 | Lpar3 | -0.96886 | 3.02E-08 |
| 26519 | Robo4 | -0.96722 | 1.71E-13 |
| 1830 | Add3 | -0.96492 | 9.41E-18 |
| 1992 | AI662270 | -0.96447 | 0.011795 |
| 28260 | Smim3 | -0.96238 | 8.97E-16 |
| 14436 | Gm4949 | -0.96144 | 9.23E-12 |
| 1803 | Adamtsl4 | -0.96111 | 3.72E-10 |
| 18018 | Jund | -0.96052 | 7.17E-19 |
| 27002 | Rtp4 | -0.96049 | 2.25E-07 |
| 18976 | Loxl2 | -0.96044 | 1.01E-16 |
| 8105 | Glns-ps1 | -0.95904 | 4.59E-08 |
| 28638 | Spata13 | -0.95872 | 1.79E-14 |
| 26449 | Rnf180 | -0.95799 | 5.59E-09 |
| 7929 | Gcc1 | -0.95778 | 6.62E-16 |
| 24563 | Phyhip | -0.95598 | 0.014429 |
| 17171 | Htra3 | -0.9558 | 1.61E-16 |
| 4935 | Col2a1 | -0.95275 | 4.29E-14 |
| 28317 | Snai1 | -0.95262 | 7.24E-17 |
| 19779 | Minar1 | -0.95203 | 0.00362 |
| 18112 | Kcnj14 | -0.9514 | 0.000796 |
| 27621 | Sh3tc1 | -0.95056 | 1.63E-07 |
| 2453 | Arap3 | -0.95013 | 1.24E-14 |
| 25661 | Ptp4a3 | -0.9497 | 1.09E-16 |
| 6976 | Exd1 | -0.94929 | 0.004385 |
| 3211 | Bhlhe40 | -0.94922 | 3.01E-17 |
| 28127 | Slc9a2 | -0.94767 | 1.32E-09 |
| 7918 | Gbp4 | -0.94713 | 0.005425 |
| 7917 | Gbp3 | -0.9471 | 2.93E-07 |
| 26486 | Rnf43 | -0.94562 | 8.98E-06 |
| 25319 | Prkcg | -0.94457 | 1.81E-08 |
| 25448 | Prr7 | -0.94434 | 2.38E-10 |
| 7743 | Fxyd7 | -0.94392 | 7.69E-06 |
| 3687 | Car13 | -0.94348 | 2.63E-12 |
| 7596 | Foxd2os | -0.94156 | 0.000791 |
| 18165 | Kctd11 | -0.9411 | 5.37E-15 |
| 18076 | Kcnb2 | -0.94105 | 1.82E-10 |
| 27490 | Serpine1 | -0.94095 | 4.65E-17 |
| 2699 | Asprv1 | -0.94014 | 0.00134 |
| 25480 | Prss35 | -0.9394 | 6.91E-13 |
| 28199 | Smad9 | -0.93827 | 8.83E-15 |
| 30175 | Tmod3 | -0.93769 | 1.49E-16 |
| 25386 | Prokr1 | -0.93685 | 1.31E-08 |
| 6524 | Eepd1 | -0.93354 | 4.61E-14 |
| 17788 | Insyn2a | -0.93268 | 2.42E-06 |
| 18304 | Klf4 | -0.93247 | 3.14E-17 |
| 30005 | Tmem176b | -0.93205 | 1.05E-14 |
| 22192 | Notum | -0.93193 | 5.93E-10 |
| 5376 | Cxcl1 | -0.93173 | 2.42E-15 |
| 5498 | Cyp2j6 | -0.93173 | 1.20E-10 |
| 27373 | Sema3a | -0.9316 | 9.98E-17 |
| 7935 | Gch1 | -0.93116 | 6.38E-07 |
| 27374 | Sema3b | -0.9293 | 1.62E-11 |
| 2698 | Aspn | -0.92891 | 4.71E-17 |
| 30329 | Tor4a | -0.9289 | 0.000686 |
| 31349 | Usp29 | -0.92844 | 3.31E-13 |
| 16374 | Grid2ip | -0.92835 | 0.000527 |
| 145 | 1700019D03Rik | -0.92834 | 0.000288 |
| 18294 | Klf10 | -0.92808 | 6.22E-18 |
| 6044 | Dlx2 | -0.92783 | 5.59E-18 |
| 24415 | Per2 | -0.9276 | 4.40E-15 |
| 7698 | Fth1 | -0.92447 | 1.95E-19 |
| 17908 | Itga2 | -0.92221 | 3.50E-11 |
| 19743 | Mgst3 | -0.9222 | 6.85E-10 |
| 26651 | Rpl26-ps6 | -0.92131 | 0.00796 |
| 9987 | Gm18555 | -0.92032 | 0.005966 |
| 24395 | Pear1 | -0.91965 | 1.29E-15 |
| 16835 | Helz2 | -0.91951 | 1.44E-15 |
| 4197 | Cdh11 | -0.91801 | 7.84E-17 |
| 16143 | Gng11 | -0.91496 | 3.74E-10 |
| 16397 | Grk3 | -0.91413 | 3.68E-16 |
| 31224 | Ugt1a7c | -0.91406 | 1.45E-05 |
| 7456 | Fgfr2 | -0.91354 | 1.02E-15 |
| 13371 | Gm29778 | -0.91312 | 9.60E-08 |
| 19158 | Ltbr | -0.91276 | 3.47E-15 |
| 30291 | Tob1 | -0.91149 | 1.68E-16 |
| 28541 | Socs1 | -0.91146 | 1.41E-13 |
| 27004 | Rttn | -0.91048 | 1.43E-15 |
| 27236 | Schip1 | -0.91034 | 0.003503 |
| 25824 | Rab32 | -0.91033 | 1.88E-12 |
| 5085 | Cpq | -0.9083 | 7.01E-13 |
| 7716 | Fuca2 | -0.90749 | 1.40E-15 |
| 19552 | Mdfi | -0.90724 | 8.06E-15 |
| 24621 | Pik3cg | -0.90722 | 6.77E-07 |
| 6960 | Eva1a | -0.90506 | 3.60E-08 |
| 3606 | Calcb | -0.90486 | 4.75E-07 |
| 7920 | Gbp6 | -0.90482 | 8.38E-06 |
| 25014 | Pon3 | -0.90474 | 2.44E-07 |
| 24787 | Pld5 | -0.90417 | 0.000238 |
| 22310 | Nrros | -0.90411 | 3.19E-10 |
| 30265 | Tnn | -0.90376 | 1.80E-13 |
| 27311 | Sdr42e1 | -0.90212 | 0.000299 |
| 6667 | Elf1 | -0.90164 | 4.14E-13 |
| 16420 | Gsc | -0.9014 | 4.51E-13 |
| 19235 | Lyz2 | -0.90098 | 1.08E-16 |
| 17708 | Il6 | -0.90073 | 5.20E-06 |
| 2726 | Atf3 | -0.89995 | 2.22E-15 |
| 25969 | Rasd1 | -0.89907 | 0.000596 |
| 16632 | H2aj | -0.8987 | 6.29E-14 |
| 6742 | Endod1 | -0.89867 | 2.29E-12 |
| 4997 | Coq10b | -0.89858 | 1.68E-12 |
| 4484 | Cgn | -0.8972 | 4.25E-05 |
| 24000 | P2ry2 | -0.89678 | 7.05E-06 |
| 16370 | Gria3 | -0.89652 | 3.17E-15 |
| 6066 | Dmrt2 | -0.89575 | 0.001788 |
| 24276 | Pcp4l1 | -0.89412 | 3.48E-08 |
| 28122 | Slc8a1 | -0.89309 | 4.79E-14 |
| 5411 | Cyba | -0.89197 | 2.81E-11 |
| 3372 | Btbd11 | -0.89123 | 1.85E-12 |
| 19426 | Mapre2 | -0.89095 | 7.15E-16 |
| 22354 | Ntf3 | -0.88936 | 1.99E-11 |
| 32530 | Zfp185 | -0.88918 | 3.10E-09 |
| 24938 | Podxl | -0.88764 | 1.46E-12 |
| 6553 | Efnb2 | -0.88657 | 9.55E-16 |
| 32050 | Vsig2 | -0.88622 | 4.09E-05 |
| 5373 | Cx3cl1 | -0.88599 | 2.79E-13 |
| 24622 | Pik3ip1 | -0.88544 | 1.10E-10 |
| 22275 | Nr4a1 | -0.88503 | 8.52E-15 |
| 18979 | Lpar1 | -0.88455 | 4.93E-17 |
| 28147 | Slco2b1 | -0.88453 | 0.001739 |
| 17777 | Insc | -0.88113 | 4.03E-11 |
| 6791 | Epb41l2 | -0.8799 | 5.84E-15 |
| 7628 | Foxp2 | -0.87917 | 7.23E-10 |
| 5504 | Cyp2t4 | -0.87913 | 0.00035 |
| 17673 | Il1rn | -0.87911 | 1.88E-07 |
| 21302 | Ms4a4a | -0.87849 | 0.002099 |
| 17977 | Jag1 | -0.87842 | 1.57E-15 |
| 27095 | Samd9l | -0.87812 | 9.53E-09 |
| 30082 | Tmem255a | -0.87801 | 4.10E-13 |
| 27939 | Slc2a13 | -0.87788 | 1.54E-11 |
| 16778 | Hck | -0.87652 | 1.92E-05 |
| 18852 | Lmo1 | -0.87639 | 1.99E-05 |
| 21787 | Ncam2 | -0.876 | 1.81E-05 |
| 16460 | Gsto1 | -0.8755 | 4.65E-12 |
| 30149 | Tmem79 | -0.87435 | 0.001032 |
| 5125 | Creb3l3 | -0.87331 | 0.003598 |
| 17857 | Irf1 | -0.87331 | 7.01E-13 |
| 5392 | Cxcr4 | -0.87327 | 6.85E-14 |
| 3972 | Ccl4 | -0.87168 | 0.001506 |
| 4471 | Cfh | -0.87105 | 3.01E-15 |
| 7017 | Ezr | -0.87018 | 2.22E-11 |
| 3426 | Btg2 | -0.86804 | 1.24E-15 |
| 17206 | Icam1 | -0.86724 | 1.51E-13 |
| 24658 | Pira2 | -0.86656 | 0.009152 |
| 2006 | Aim2 | -0.86531 | 0.000209 |
| 25394 | Pros1 | -0.86486 | 1.83E-14 |
| 3146 | Bcl2a1b | -0.86414 | 0.011915 |
| 18993 | Lpin3 | -0.86412 | 7.87E-05 |
| 27571 | Sgk3 | -0.8636 | 5.98E-09 |
| 2376 | Apela | -0.86335 | 0.000787 |
| 16465 | Gstt1 | -0.86322 | 1.78E-09 |
| 17905 | Itga1 | -0.86221 | 3.94E-12 |
| 18348 | Klhl4 | -0.86203 | 2.39E-08 |
| 14774 | Gm5739 | -0.86112 | 1.28E-06 |
| 4950 | Col6a5 | -0.86089 | 5.23E-05 |
| 3491 | C1rb | -0.86045 | 8.65E-06 |
| 9457 | Gm16011 | -0.86029 | 0.002134 |
| 6864 | Erf | -0.85992 | 1.20E-13 |
| 25295 | Prickle3 | -0.8574 | 2.94E-09 |
| 18156 | Kcns3 | -0.85696 | 4.91E-05 |
| 6500 | Edn3 | -0.85425 | 1.64E-12 |
| 1855 | Adgrl2 | -0.85417 | 3.54E-15 |
| 7213 | Fam81a | -0.854 | 0.003527 |
| 28958 | Stab1 | -0.85369 | 2.69E-11 |
| 26412 | Rnf112 | -0.8531 | 4.70E-06 |
| 6482 | Ecscr | -0.85204 | 1.15E-08 |
| 29477 | Tbxas1 | -0.85135 | 0.000524 |
| 3658 | Capg | -0.85007 | 1.06E-10 |
| 18222 | Khk | -0.84969 | 1.02E-07 |
| 18300 | Klf16 | -0.84961 | 4.93E-12 |
| 29237 | Syt11 | -0.84908 | 3.17E-12 |
| 28758 | Spock1 | -0.84898 | 4.06E-13 |
| 6486 | Eda | -0.84851 | 1.53E-11 |
| 22016 | Ngfr | -0.84781 | 4.34E-14 |
| 4097 | Cd300lf | -0.84773 | 3.09E-05 |
| 22309 | Nrp2 | -0.84747 | 3.32E-14 |
| 3569 | Cacna1d | -0.84662 | 3.08E-13 |
| 4878 | Cntfr | -0.84595 | 4.01E-09 |
| 1167 | 5730416F02Rik | -0.84491 | 0.001244 |
| 29790 | Tifa | -0.84473 | 3.34E-07 |
| 15175 | Gm7049 | -0.844 | 1.36E-09 |
| 15782 | Gm8812 | -0.84378 | 1.51E-06 |
| 24768 | Plcb4 | -0.84375 | 2.01E-12 |
| 27812 | Slc17a9 | -0.84346 | 6.64E-05 |
| 3490 | C1ra | -0.84345 | 5.45E-09 |
| 18284 | Kit | -0.84332 | 2.74E-11 |
| 21569 | Myo1e | -0.84321 | 4.87E-13 |
| 31340 | Usp2 | -0.84214 | 3.87E-13 |
| 10711 | Gm21451 | -0.84205 | 1.02E-10 |
| 21308 | Ms4a6c | -0.84191 | 4.81E-05 |
| 17074 | Hs2st1 | -0.84189 | 1.40E-13 |
| 3982 | Ccn3 | -0.84133 | 2.67E-07 |
| 25984 | Rasl11b | -0.8404 | 2.33E-13 |
| 24628 | Pik3r6 | -0.84029 | 0.000336 |
| 3474 | C1qb | -0.84 | 7.20E-10 |
| 29745 | Thbs3 | -0.83933 | 2.09E-12 |
| 21151 | Mr1 | -0.83933 | 1.81E-11 |
| 24862 | Plscr1 | -0.83783 | 9.29E-07 |
| 5571 | Cyyr1 | -0.83728 | 0.000693 |
| 15789 | Gm8834 | -0.83652 | 0.003372 |
| 3822 | Ccdc126 | -0.8356 | 3.62E-09 |
| 5300 | Ctnna2 | -0.83473 | 3.06E-09 |
| 29700 | Tfr2 | -0.83444 | 0.010264 |
| 28581 | Sox17 | -0.83417 | 8.21E-06 |
| 26451 | Rnf182 | -0.83282 | 8.61E-12 |
| 16353 | Grap | -0.83279 | 5.85E-06 |
| 5136 | Crem | -0.83237 | 1.32E-10 |
| 32409 | Zbtb7c | -0.83232 | 4.12E-10 |
| 24763 | Plbd1 | -0.83227 | 0.013778 |
| 30881 | Tspan18 | -0.83217 | 1.90E-13 |
| 22204 | Npas4 | -0.83142 | 2.47E-11 |
| 7473 | Fhit | -0.83097 | 0.011191 |
| 6986 | Exoc3l4 | -0.8305 | 6.41E-07 |
| 30915 | Tst | -0.83001 | 2.48E-06 |
| 24769 | Plcd1 | -0.82962 | 1.38E-12 |
| 24318 | Pde3b | -0.82954 | 2.73E-10 |
| 27983 | Slc35e4 | -0.82936 | 8.07E-10 |
| 16107 | Gmfg-ps | -0.82913 | 0.008478 |
| 7122 | Fam167a | -0.82913 | 4.12E-08 |
| 4915 | Col11a1 | -0.82837 | 2.72E-13 |
| 4142 | Cd86 | -0.82788 | 0.00981 |
| 21310 | Ms4a7 | -0.82721 | 3.99E-08 |
| 6547 | Efna1 | -0.8258 | 3.36E-10 |
| 19273 | Maff | -0.82565 | 9.93E-12 |
| 16764 | Hbegf | -0.82535 | 9.68E-10 |
| 10055 | Gm18711 | -0.82474 | 0.000342 |
| 28804 | Spsb1 | -0.82345 | 5.42E-12 |
| 25978 | Rasgrp3 | -0.82177 | 7.26E-10 |
| 30102 | Tmem30b | -0.82161 | 4.52E-11 |
| 6788 | Epas1 | -0.82134 | 3.54E-10 |
| 16373 | Grid2 | -0.82085 | 9.12E-09 |
| 6078 | Dmtn | -0.81998 | 5.83E-06 |
| 18208 | Kdr | -0.81839 | 5.10E-12 |
| 25983 | Rasl11a | -0.81793 | 8.46E-11 |
| 27098 | Samsn1 | -0.81681 | 0.014225 |
| 4589 | Chst11 | -0.81673 | 1.19E-12 |
| 21703 | Naalad2 | -0.81647 | 1.96E-08 |
| 29516 | Tcim | -0.8157 | 1.42E-13 |
| 24633 | Pim1 | -0.81543 | 3.15E-11 |
| 18833 | Llgl2 | -0.81493 | 0.00019 |
| 21508 | Myc | -0.81475 | 2.30E-11 |
| 22132 | Nmnat2 | -0.81446 | 4.31E-10 |
| 6002 | Dipk2a | -0.81413 | 5.77E-13 |
| 931 | 4930562C15Rik | -0.81356 | 1.05E-06 |
| 7944 | Gcnt1 | -0.81339 | 4.72E-08 |
| 30174 | Tmod2 | -0.81308 | 8.45E-12 |
| 19567 | Me1 | -0.81124 | 8.64E-11 |
| 22589 | Olfml3 | -0.81108 | 9.10E-12 |
| 2601 | Armh4 | -0.80901 | 1.74E-10 |
| 6048 | Dlx5 | -0.8086 | 8.27E-12 |
| 17226 | Idi2 | -0.80799 | 0.000868 |
| 29945 | Tmem119 | -0.80779 | 3.45E-11 |
| 5385 | Cxcl2 | -0.80743 | 0.000313 |
| 30875 | Tspan11 | -0.80688 | 3.31E-12 |
| 25683 | Ptprb | -0.80619 | 2.38E-09 |
| 17779 | Insig2 | -0.80586 | 2.20E-10 |
| 2407 | Apoe | -0.80585 | 1.57E-12 |
| 19397 | Map7 | -0.80577 | 3.80E-05 |
| 6234 | Dpp4 | -0.80544 | 0.000905 |
| 15565 | Gm8174 | -0.80508 | 0.0021 |
| 27061 | S1pr1 | -0.80417 | 5.46E-14 |
| 4116 | Cd48 | -0.804 | 0.006857 |
| 30825 | Trpm5 | -0.80355 | 1.47E-11 |
| 29860 | Tlr1 | -0.80329 | 0.000541 |
| 8467 | Gm11837 | -0.80307 | 0.007562 |
| 21038 | Mmp11 | -0.8022 | 4.32E-12 |
| 25458 | Prrt1 | -0.80197 | 3.54E-05 |
| 3473 | C1qa | -0.80158 | 5.80E-10 |
| 7199 | Fam43a | -0.80116 | 6.49E-12 |
| 7583 | Fos | -0.80089 | 9.99E-13 |
| 26885 | Rps6ka5 | -0.80078 | 3.31E-11 |
| 17268 | Ifitm3 | -0.7996 | 3.70E-14 |
| 32252 | Xaf1 | -0.79886 | 3.16E-05 |
| 16882 | Hic1 | -0.79834 | 4.57E-13 |
| 3971 | Ccl3 | -0.79666 | 0.000982 |
| 4848 | Cnn1 | -0.7963 | 8.76E-08 |
| 24215 | Pcdhb2 | -0.7962 | 0.003511 |
| 7414 | Fez1 | -0.79311 | 5.72E-10 |
| 18648 | Lbp | -0.79281 | 4.54E-05 |
| 28523 | Snx24 | -0.79245 | 2.17E-09 |
| 4271 | Cdo1 | -0.79206 | 7.41E-12 |
| 29678 | Tfap2b | -0.79114 | 1.43E-12 |
| 5185 | Crybb3 | -0.78978 | 0.002452 |
| 2429 | App | -0.78915 | 2.79E-12 |
| 19533 | Mcoln3 | -0.78857 | 0.000475 |
| 4563 | Chrdl1 | -0.78692 | 1.24E-08 |
| 30153 | Tmem86a | -0.78687 | 3.82E-09 |
| 50 | 1600014C10Rik | -0.78672 | 2.17E-09 |
| 5111 | Cracr2b | -0.78671 | 9.00E-05 |
| 1587 | Abi3 | -0.78537 | 0.002144 |
| 18740 | Lgals3 | -0.78496 | 1.26E-11 |
| 24320 | Pde4b | -0.78466 | 1.75E-09 |
| 29980 | Tmem150c | -0.78465 | 0.000356 |
| 25649 | Pthlh | -0.78441 | 3.96E-07 |
| 16758 | Hbb-b1 | -0.78436 | 6.01E-07 |
| 22572 | Ogfrl1 | -0.78424 | 3.09E-09 |
| 1793 | Adamts3 | -0.78403 | 1.77E-07 |
| 7426 | Fgd3 | -0.78384 | 5.72E-08 |
| 27715 | Six2 | -0.78335 | 8.40E-12 |
| 27083 | Samd10 | -0.78325 | 0.00106 |
| 30117 | Tmem44 | -0.78317 | 2.25E-08 |
| 2608 | Arpc1b | -0.78291 | 9.03E-11 |
| 17991 | Jcad | -0.78258 | 8.41E-11 |
| 26054 | Rbm47 | -0.78233 | 0.000249 |
| 5294 | Cthrc1 | -0.78142 | 2.17E-11 |
| 5112 | Cradd | -0.78128 | 2.89E-06 |
| 14186 | Gm4544 | -0.78083 | 0.01116 |
| 29130 | Sult4a1 | -0.78057 | 0.000105 |
| 25320 | Prkch | -0.78 | 4.76E-09 |
| 5142 | Crip1 | -0.77997 | 5.00E-08 |
| 5337 | Ctss | -0.77908 | 1.55E-11 |
| 30951 | Ttc39b | -0.77814 | 7.65E-12 |
| 24670 | Pitpnm1 | -0.77787 | 7.51E-10 |
| 18855 | Lmo4 | -0.77697 | 3.13E-13 |
| 28055 | Slc4a11 | -0.77498 | 3.89E-08 |
| 24775 | Plch1 | -0.77478 | 0.008128 |
| 30124 | Tmem51 | -0.774 | 6.67E-07 |
| 32931 | Zim1 | -0.7739 | 6.25E-12 |
| 6572 | Egr4 | -0.77307 | 2.07E-08 |
| 19168 | Lum | -0.7721 | 1.04E-11 |
| 27544 | Sfrp4 | -0.7719 | 2.97E-11 |
| 7068 | Fah | -0.77176 | 7.24E-07 |
| 18285 | Kitl | -0.77156 | 3.10E-12 |
| 7727 | Fut4 | -0.77155 | 0.003551 |
| 32256 | Xdh | -0.77151 | 0.000464 |
| 17922 | Itgax | -0.77117 | 0.000943 |
| 29775 | Thsd7b | -0.77101 | 5.82E-05 |
| 25949 | Rapgef4 | -0.77068 | 4.31E-05 |
| 27015 | Runx1 | -0.77022 | 1.82E-12 |
| 23915 | Osbpl10 | -0.76907 | 2.43E-08 |
| 25630 | Ptger4 | -0.76888 | 1.41E-08 |
| 21309 | Ms4a6d | -0.76841 | 1.00E-08 |
| 2511 | Arhgap6 | -0.7676 | 8.86E-11 |
| 4499 | Chchd10 | -0.76639 | 0.000394 |
| 7957 | Gdf10 | -0.76573 | 6.22E-12 |
| 24829 | Plin2 | -0.76538 | 1.67E-11 |
| 18753 | Lgr5 | -0.76534 | 2.67E-12 |
| 14658 | Gm5408 | -0.76512 | 0.000445 |
| 4737 | Clec4a1 | -0.76455 | 0.002337 |
| 29569 | Tdrp | -0.7645 | 0.00408 |
| 29866 | Tlr4 | -0.76424 | 0.000126 |
| 7631 | Foxq1 | -0.76421 | 0.001207 |
| 21988 | Nfe2l2 | -0.7635 | 1.60E-11 |
| 16351 | Gramd3 | -0.76326 | 1.73E-11 |
| 2604 | Arnt2 | -0.762 | 8.07E-12 |
| 24738 | Plac1 | -0.76176 | 0.001841 |
| 26052 | Rbm46 | -0.75926 | 0.001286 |
| 21078 | Mob3b | -0.75888 | 5.83E-10 |
| 1548 | Abcc9 | -0.75861 | 1.98E-10 |
| 18588 | Lacc1 | -0.75814 | 2.31E-09 |
| 2573 | Arl5c | -0.75803 | 1.97E-05 |
| 31376 | Usp6nl | -0.75718 | 1.06E-10 |
| 28045 | Slc45a4 | -0.75713 | 5.63E-12 |
| 23965 | Otulinl | -0.75697 | 1.36E-07 |
| 21566 | Myo1b | -0.75665 | 7.95E-13 |
| 1557 | Abcg1 | -0.75552 | 0.000187 |
| 1781 | Adamts1 | -0.75477 | 1.19E-11 |
| 2342 | Ap1s3 | -0.75443 | 0.002413 |
| 4101 | Cd33 | -0.75441 | 0.000162 |
| 17865 | Irf6 | -0.75426 | 1.29E-09 |
| 7660 | Frmd5 | -0.75284 | 7.56E-07 |
| 7738 | Fxyd2 | -0.75221 | 3.35E-05 |
| 19650 | Metrnl | -0.75211 | 1.25E-09 |
| 22153 | Nod1 | -0.75148 | 1.30E-08 |
| 19076 | Lrrc49 | -0.7513 | 1.55E-09 |
| 26274 | Rhov | -0.75115 | 0.00356 |
| 7100 | Fam131b | -0.75104 | 1.91E-05 |
| 26264 | Rhoc | -0.75093 | 6.10E-11 |
| 27729 | Skil | -0.75074 | 1.02E-10 |
| 29244 | Syt2 | -0.75054 | 4.45E-05 |
| 27473 | Serpinb6a | -0.75026 | 4.29E-12 |
| 17871 | Irgm2 | -0.75002 | 8.35E-08 |
| 4743 | Clec4d | -0.74938 | 2.05E-07 |
| 19199 | Ly9 | -0.74938 | 1.75E-05 |
| 17083 | Hs6st2 | -0.74932 | 2.89E-10 |
| 2973 | B3glct | -0.74897 | 8.39E-11 |
| 27927 | Slc27a6 | -0.74886 | 1.51E-07 |
| 4202 | Cdh17 | -0.74885 | 0.003257 |
| 24497 | Phactr2 | -0.74852 | 8.94E-11 |
| 7007 | Extl1 | -0.74839 | 0.002062 |
| 30846 | Tsc22d1 | -0.74753 | 2.63E-12 |
| 25701 | Ptpru | -0.74678 | 5.33E-09 |
| 24881 | Plxnd1 | -0.746 | 7.38E-11 |
| 31266 | Unc5b | -0.74514 | 2.05E-10 |
| 29855 | Tll1 | -0.74511 | 3.98E-07 |
| 4845 | Cnksr2 | -0.74478 | 4.35E-08 |
| 7812 | Gadd45b | -0.7447 | 1.50E-11 |
| 25173 | Ppp3cc | -0.74422 | 6.33E-08 |
| 15167 | Gm7019 | -0.74397 | 0.000208 |
| 24358 | Pdk4 | -0.74359 | 6.48E-07 |
| 28769 | Spp1 | -0.74279 | 2.69E-11 |
| 28120 | Slc7a8 | -0.74254 | 2.39E-09 |
| 26238 | Rgs7bp | -0.74198 | 7.07E-06 |
| 21730 | Nalf1 | -0.74147 | 5.83E-07 |
| 2538 | Arhgef5 | -0.74055 | 3.67E-10 |
| 16912 | Hk2 | -0.74038 | 1.20E-09 |
| 4279 | Cds1 | -0.73994 | 6.55E-07 |
| 6732 | Emp3 | -0.73993 | 2.15E-10 |
| 29869 | Tlr7 | -0.73899 | 5.45E-06 |
| 26967 | Rspo3 | -0.73847 | 1.05E-08 |
| 2029 | Akap12 | -0.73794 | 1.59E-10 |
| 25124 | Ppp1r15a | -0.73777 | 5.08E-10 |
| 2365 | Apba2 | -0.73759 | 2.69E-05 |
| 23998 | P2ry13 | -0.73731 | 0.010465 |
| 17870 | Irgm1 | -0.73651 | 3.03E-07 |
| 4119 | Cd53 | -0.73529 | 1.47E-07 |
| 7921 | Gbp7 | -0.7352 | 3.01E-06 |
| 3466 | C130074G19Rik | -0.73516 | 4.15E-09 |
| 30888 | Tspan4 | -0.73513 | 3.63E-10 |
| 8033 | Gimap4 | -0.73288 | 1.68E-05 |
| 29226 | Synpo | -0.73269 | 7.41E-11 |
| 3263 | Bmp6 | -0.73268 | 3.93E-10 |
| 30710 | Trim30a | -0.73205 | 0.000507 |
| 8022 | Ghr | -0.73138 | 3.47E-10 |
| 32340 | Ypel4 | -0.73137 | 3.49E-11 |
| 5507 | Cyp39a1 | -0.73113 | 6.25E-06 |
| 2522 | Arhgef15 | -0.73088 | 3.18E-07 |
| 32468 | Zdhhc23 | -0.73031 | 0.011723 |
| 24937 | Podnl1 | -0.72936 | 3.16E-06 |
| 4886 | Cntnap1 | -0.72839 | 8.49E-05 |
| 3934 | Ccdc88c | -0.72768 | 1.92E-08 |
| 18627 | Large1 | -0.72661 | 2.68E-11 |
| 4887 | Cntnap2 | -0.72645 | 9.18E-05 |
| 28543 | Socs3 | -0.7261 | 2.39E-10 |
| 3732 | Casp8 | -0.72529 | 3.03E-09 |
| 30371 | Tpst1 | -0.72454 | 1.04E-11 |
| 6479 | Ecm2 | -0.72422 | 0.000137 |
| 18017 | Junb | -0.72354 | 3.76E-11 |
| 16362 | Grem1 | -0.72318 | 1.76E-09 |
| 17259 | Ifit1 | -0.72265 | 0.000536 |
| 7737 | Fxyd1 | -0.72225 | 1.12E-09 |
| 22587 | Olfml2a | -0.72098 | 1.24E-08 |
| 3740 | Castor1 | -0.72067 | 0.001526 |
| 26432 | Rnf144a | -0.72026 | 2.00E-11 |
| 27786 | Slc15a2 | -0.7198 | 4.59E-09 |
| 8080 | Gla | -0.71936 | 4.04E-09 |
| 29159 | Susd4 | -0.71904 | 1.41E-05 |
| 29175 | Svip | -0.71899 | 0.00095 |
| 19751 | Mical2 | -0.71899 | 1.61E-10 |
| 24057 | Palld | -0.71896 | 3.77E-11 |
| 3476 | C1qc | -0.71866 | 3.10E-08 |
| 30167 | Tmem98 | -0.71865 | 6.95E-10 |
| 3481 | C1qtnf1 | -0.71831 | 1.60E-09 |
| 4233 | Cdk15 | -0.71801 | 0.004296 |
| 15668 | Gm8436 | -0.71526 | 3.01E-07 |
| 8093 | Gli2 | -0.71442 | 2.28E-09 |
| 27162 | Scand1 | -0.71383 | 0.012084 |
| 24883 | Pm20d2 | -0.71333 | 0.004274 |
| 19354 | Maob | -0.71323 | 9.80E-06 |
| 21801 | Ncf1 | -0.71252 | 5.54E-07 |
| 24115 | Parp9 | -0.7121 | 1.73E-06 |
| 17167 | Htr6 | -0.71157 | 0.006996 |
| 2010 | Aipl1 | -0.71153 | 0.007502 |
| 5101 | Cpxm1 | -0.71127 | 1.60E-10 |
| 2454 | Arc | -0.71038 | 5.88E-09 |
| 19175 | Lxn | -0.71038 | 1.57E-10 |
| 25013 | Pon2 | -0.70903 | 5.75E-08 |
| 4123 | Cd59a | -0.70897 | 1.74E-07 |
| 5322 | Ctsb | -0.7088 | 1.86E-11 |
| 18729 | Lepr | -0.70791 | 2.40E-09 |
| 16221 | Gpc3 | -0.70766 | 2.58E-10 |
| 18091 | Kcng1 | -0.70744 | 4.55E-06 |
| 24921 | Pnp2 | -0.70716 | 0.000204 |
| 16126 | Gnal | -0.70656 | 1.62E-06 |
| 28314 | Smyd3 | -0.7065 | 8.79E-10 |
| 4205 | Cdh2 | -0.70595 | 1.87E-09 |
| 28148 | Slco3a1 | -0.7056 | 6.64E-08 |
| 25640 | Ptgr2 | -0.70546 | 7.17E-07 |
| 26233 | Rgs3 | -0.7048 | 4.97E-09 |
| 7055 | Fabp4 | -0.70249 | 0.000125 |
| 6675 | Elk3 | -0.70158 | 8.96E-09 |
| 6845 | Erap1 | -0.70102 | 2.18E-07 |
| 27772 | Slc12a5 | -0.70043 | 1.17E-07 |
| 19607 | Medag | -0.69848 | 0.003055 |
| 25093 | Ppl | -0.69777 | 8.66E-06 |
| 32594 | Zfp362 | -0.69761 | 1.74E-09 |
| 30984 | Ttpa | -0.69722 | 0.006955 |
| 2556 | Arl11 | -0.6969 | 0.00037 |
| 30813 | Trpc3 | -0.69668 | 0.002625 |
| 25749 | Pxylp1 | -0.69571 | 4.21E-08 |
| 6050 | Dlx6os1 | -0.6957 | 5.66E-07 |
| 28982 | Stard8 | -0.6948 | 1.08E-05 |
| 25702 | Ptprv | -0.69474 | 5.56E-08 |
| 30880 | Tspan17 | -0.69463 | 2.28E-05 |
| 17130 | Hspa2 | -0.69393 | 4.20E-06 |
| 2414 | Apol6 | -0.69316 | 0.001525 |
| 24047 | Pak1 | -0.69273 | 1.20E-09 |
| 17633 | Il10rb | -0.69259 | 2.80E-07 |
| 28701 | Spef1 | -0.69227 | 4.62E-07 |
| 18602 | Lamb1 | -0.69142 | 5.22E-10 |
| 24219 | Pcdhb3 | -0.69068 | 1.60E-06 |
| 16712 | Hacd3 | -0.6901 | 2.92E-09 |
| 25505 | Prtg | -0.69003 | 0.000537 |
| 25829 | Rab36 | -0.68997 | 1.67E-08 |
| 28160 | Slfn2 | -0.68988 | 2.47E-05 |
| 24887 | Pmepa1 | -0.68986 | 7.28E-10 |
| 4944 | Col5a2 | -0.68969 | 6.95E-09 |
| 30004 | Tmem176a | -0.6891 | 4.42E-09 |
| 24360 | Pdlim2 | -0.68788 | 4.45E-06 |
| 16513 | Gucy1a1 | -0.6873 | 9.36E-09 |
| 6310 | Dtx3l | -0.6872 | 8.36E-06 |
| 24450 | Pfkfb3 | -0.68718 | 1.18E-08 |
| 23993 | P2rx7 | -0.68683 | 0.000749 |
| 29157 | Susd2 | -0.68678 | 0.000182 |
| 1725 | Actr3b | -0.68647 | 0.000351 |
| 27978 | Slc35d2 | -0.68643 | 0.004989 |
| 8992 | Gm14032 | -0.68597 | 3.89E-08 |
| 21155 | Mrc1 | -0.68517 | 4.48E-08 |
| 18291 | Klc3 | -0.68502 | 0.007702 |
| 17629 | Ikzf4 | -0.68439 | 6.28E-09 |
| 26473 | Rnf225 | -0.68363 | 0.000515 |
| 7479 | Fhl5 | -0.6825 | 0.002046 |
| 17255 | Ifi44 | -0.68157 | 0.000193 |
| 2523 | Arhgef16 | -0.68011 | 0.003939 |
| 3511 | C3 | -0.67972 | 0.006794 |
| 19306 | Magee2 | -0.67875 | 0.000428 |
| 29983 | Tmem151b | -0.67828 | 2.36E-07 |
| 1019 | 4931440P22Rik | -0.67665 | 0.010801 |
| 3627 | Calr3 | -0.67594 | 0.006878 |
| 14728 | Gm5637 | -0.67572 | 2.25E-07 |
| 4063 | Cd200r1 | -0.67554 | 0.004223 |
| 4250 | Cdk6 | -0.67548 | 1.00E-08 |
| 24869 | Plvap | -0.67517 | 1.66E-07 |
| 30107 | Tmem37 | -0.67407 | 0.000277 |
| 5439 | Cyp26b1 | -0.67404 | 1.76E-09 |
| 32407 | Zbtb7a | -0.67299 | 1.26E-08 |
| 27047 | S100a16 | -0.67211 | 3.88E-08 |
| 18352 | Klhl5 | -0.66997 | 1.57E-08 |
| 5339 | Ctsz | -0.66968 | 8.07E-08 |
| 27525 | Sez6l2 | -0.66964 | 0.011463 |
| 21496 | Mxra8 | -0.66917 | 1.59E-09 |
| 6121 | Dnajb2 | -0.66888 | 2.48E-08 |
| 25462 | Prrx1 | -0.66761 | 6.64E-10 |
| 6761 | Enpp2 | -0.66742 | 2.60E-09 |
| 30784 | Trmt2b | -0.66668 | 2.61E-07 |
| 7844 | Galnt6 | -0.66658 | 0.000165 |
| 16666 | H2bc27 | -0.66621 | 0.000511 |
| 3267 | Bmper | -0.66609 | 3.33E-07 |
| 28635 | Sparcl1 | -0.66578 | 3.75E-10 |
| 558 | 2900026A02Rik | -0.66453 | 2.11E-07 |
| 6584 | Eid1 | -0.66386 | 5.06E-09 |
| 27606 | Sh3bp5 | -0.66283 | 8.34E-09 |
| 1513 | Abca1 | -0.66233 | 1.17E-08 |
| 16139 | Gnb4 | -0.66186 | 5.85E-09 |
| 22472 | Oaf | -0.66185 | 2.43E-08 |
| 17910 | Itga3 | -0.66168 | 1.36E-06 |
| 8247 | Gm10516 | -0.66162 | 0.003298 |
| 5937 | Dgka | -0.66125 | 1.94E-07 |
| 4960 | Colec12 | -0.66075 | 3.11E-09 |
| 17850 | Irag2 | -0.66069 | 0.000631 |
| 21307 | Ms4a6b | -0.66048 | 0.000421 |
| 19204 | Lyl1 | -0.66023 | 0.008505 |
| 17263 | Ifit3 | -0.65927 | 0.00463 |
| 3024 | Baiap2 | -0.65842 | 4.60E-08 |
| 18344 | Klhl34 | -0.65742 | 0.002271 |
| 25840 | Rab3il1 | -0.65694 | 2.96E-08 |
| 7366 | Fcer1g | -0.65674 | 3.02E-06 |
| 6478 | Ecm1 | -0.65623 | 3.77E-06 |
| 7825 | Gale | -0.65541 | 1.61E-07 |
| 3503 | C2cd2l | -0.65513 | 9.29E-09 |
| 25143 | Ppp1r3b | -0.65492 | 1.24E-08 |
| 652 | 4930404I05Rik | -0.65389 | 0.010316 |
| 24222 | Pcdhb6 | -0.65297 | 0.000189 |
| 5918 | Dera | -0.65132 | 8.52E-06 |
| 28203 | Smarca1 | -0.65083 | 3.08E-08 |
| 17215 | Id1 | -0.6498 | 4.86E-08 |
| 5952 | Dhdh | -0.64852 | 0.001362 |
| 24143 | Pawr | -0.64804 | 3.75E-06 |
| 26530 | Rorb | -0.64768 | 0.00232 |
| 4930 | Col24a1 | -0.64755 | 8.53E-08 |
| 7025 | F2r | -0.6459 | 1.18E-09 |
| 6730 | Emp1 | -0.64553 | 1.63E-09 |
| 6049 | Dlx6 | -0.64425 | 8.54E-07 |
| 3116 | Bc1 | -0.64424 | 0.008151 |
| 21421 | Mtus2 | -0.64421 | 0.000137 |
| 26331 | Rilpl2 | -0.64372 | 3.25E-07 |
| 7082 | Fam110d | -0.64357 | 0.000853 |
| 19077 | Lrrc4b | -0.64343 | 1.64E-06 |
| 25923 | Ramp2 | -0.64341 | 2.05E-07 |
| 22482 | Oas2 | -0.64337 | 0.010531 |
| 1362 | A2m | -0.64317 | 9.15E-05 |
| 29016 | Stk17b | -0.64287 | 6.79E-07 |
| 3259 | Bmp2k | -0.64161 | 6.55E-07 |
[truncated: 101,277 more chars]
